# Safety and Efficacy of the Addition of Lapatinib to Perioperative Chemotherapy for Resectable HER2-Positive Gastroesophageal Adenocarcinoma: A Randomized Phase 2 Clinical Trial

**DOI:** 10.1001/jamaoncol.2019.1179

**Published:** 2019-06-20

**Authors:** Elizabeth C. Smyth, Samuel Rowley, Fay H. Cafferty, William Allum, Heike I. Grabsch, Sally Stenning, Andrew Wotherspoon, Derek Alderson, Tom Crosby, Was Mansoor, Justin S. Waters, Helen Neville-Webbe, Suzanne Darby, Jo Dent, Matthew Seymour, Joyce Thompson, Sharmila Sothi, Jane Blazeby, Ruth E. Langley, David Cunningham

**Affiliations:** 1 Department of Gastrointestinal Oncology and Lymphoma, Royal Marsden Hospital, London and Surrey, United Kingdom; 2 Department of Oncology,Cambridge University Hospitals, NHS Foundation Trust, Hill’s Road, Cambridge, United Kingdom; 3 Medical Research Council, Clinical Trials Unit, University College London, United Kingdom; 4 Department of Surgery, Royal Marsden Hospital, London and Surrey, United Kingdom; 5 Department of Pathology, GROW School for Oncology and Developmental Biology, Maastricht University Medical Center+, Maastricht, the Netherlands; 6 Division of Pathology and Data Analytics, Leeds Institute of Medical Research at St James’s, University of Leeds, Leeds, United Kingdom; 7 Department of Pathology, Royal Marsden Hospital, London and Surrey, United Kingdom; 8 Department of Surgery, Queen Elizabeth Hospital, Birmingham, United Kingdom; 9 Velindre Cancer Centre, Cardiff, United Kingdom; 10 Department of Medical Oncology, Christie Hospital, Manchester, United Kingdom; 11 Kent Oncology Centre, Maidstone Hospital, Kent, United Kingdom; 12 Clatterbridge Cancer Centre, Wirral, United Kingdom; 13 Department of Oncology, Weston Park Hospital, Sheffield, United Kingdom; 14 Department of Oncology, Huddersfield Royal Infirmary, Huddersfield, United Kingdom; 15 Leeds Institute of Cancer and Pathology, University of Leeds, Leeds, United Kingdom; 16 Heart of England NHS Trust, Birmingham, United Kingdom; 17 Department of Oncology,University Hospitals, Coventry, United Kingdom; 18 Bristol Centre for Surgical Research, Population Health Sciences, University of Bristol, Bristol, United Kingdom

## Abstract

**Importance:**

Perioperative chemotherapy and surgery are a standard of care for operable gastroesophageal adenocarcinoma. Anti-HER2 therapy improves survival in patients with advanced HER2-positive disease. The safety and feasibility of adding lapatinib to perioperative chemotherapy should be assessed.

**Objectives:**

To assess the safety of adding lapatinib to epirubicin, cisplatin, and capecitabine (ECX) chemotherapy and to establish a recommended dose regimen for a phase 3 trial.

**Design, Setting, and Participants:**

Phase 2 randomized, open-label trial comparing standard ECX (sECX: 3 preoperative and 3 postoperative cycles of ECX with modified ECX plus lapatinib (mECX+L). This multicenter national trial was conducted in 29 centers in the United Kingdom in patients with histologically proven, HER2-positive, operable gastroesophageal adenocarcinoma. Registration for *ERBB/HER2* testing took place from February 25, 2013, to April 19, 2016, and randomization took place between May 24, 2013, and April 21, 2016. Data were analyzed May 10, 2017, to May 25, 2017.

**Interventions:**

Patients were randomized 1:1 open-label to sECX (3 preoperative and 3 postoperative cycles of 50 mg/m^2^ of intravenous epirubicin on day 1, 60 mg/m^2^ intravenous cisplatin on day 1, 1250 mg/m^2 ^of oral capecitabine on days 1 through 21) or mECX+L (ECX plus lapatinib days 1 through 21 in each cycle and as 6 maintenance doses). The first 10 patients in the mECX+L arm were treated with 1000 mg/m^2^ of capecitabine and 1250 mg of lapatinib per day, after which preoperative toxic effects were reviewed according to predefined criteria to determine doses for subsequent patients.

**Main Outcomes and Measures:**

Proportion of patients experiencing grade 3 or 4 diarrhea with mECX+L. A rate of 20% or less was considered acceptable. No formal comparison between arms was planned.

**Results:**

Between February 2013, and April 2016, 441 patients underwent central HER2 testing and 63 (14%) were classified as HER2 positive. Forty-six patients were randomized; 44 (24 sECX, 20 mECX+L) are included in this analysis. Two of the first 10 patients in the mECX+L arm reported preoperative grade 3 diarrhea; thus, no dose increase was made. The primary endpoint of preoperative grade 3 or 4 diarrhea rates were 0 of 24 in the sECX arm (0%) and 4 of 20 in the mECX+L arm (21%). One of 24 in the sECX arm and 3 of 20 in the mECX+L arm stopped preoperative treatment early, and for 4 of 19 in the mECX+L arm, lapatinib dose was reduced. Postoperative complication rates were similar in each arm.

**Conclusions and Relevance:**

Administration of 1250 mg of lapatinib per day in combination with ECX chemotherapy was feasible with some increase in toxic effects, which did not compromise operative management.

**Trial Registration:**

ISRCTN.org identifier: 46020948; clinicaltrialsregister.eu identifier: 2006-000811-12

## Key Points

**Question:**

Therapy targeting HER2 increases overall survival in patients with advanced HER2-positive gastroesophageal cancer but has not yet been evaluated in patients with potentially curable disease.

**Findings:**

This randomized phase 2 study investigated standard perioperative chemotherapy with and without lapatinib in patients with resectable gastroesophageal cancer. Lapatinib at 1250 mg/d in combination with modified epirubicin, cisplatin and capecitabine chemotherapy (capecitabine dose, 1000 mg/mg^2^/d) was feasible and did not compromise operative management; however, toxic effects exceeded predefined acceptable parameters.

**Meaning:**

Biomarker-selected trials in HER2-positive patients with operable gastroesophageal cancer are feasible, but drug combinations with lower toxic effects than epirubicin, cisplatin, and capecitabine plus lapatinib should be evaluated.

## Teaser

This phase 2 randomized clinical trial investigates the effects of adding lapatinib to perioperative epirubicin, cisplatin, and capecitabine chemotherapy in patients with potentially curable HER2-positive gastroesophageal adenocarcinoma.

## Introduction

Perioperative chemotherapy and surgery improve overall survival compared with surgery alone in patients with operable gastroesophageal cancer, and the combination is a treatment approach recommended by current international guidelines.^1,2^ However, because 5-year overall survival for patients treated with contemporary perioperative chemotherapy is less than 50%, improvements in currently available regimens are urgently needed.^3^ Overexpression of the human epidermal growth factor receptor 2 (HER2) protein is found in up to 22% of gastric and gastroesophageal adenocarcinomas.^4^ In the Trastuzumab for Gastric Cancer (ToGA) trial, addition of the HER2-targeting monoclonal antibody trastuzumab to platinum-fluoropyrimidine chemotherapy in advanced HER2-positive gastric cancer improved radiologic response rates, progression-free survival, and overall survival compared with chemotherapy (hazard ratio, 0.74; 95% CI, 0.60-0.91; *P* = .005).^5^ The rationale for the current trial was to increase the pathologic response rate to neoadjuvant chemotherapy by the addition of anti-HER2 treatment, and thereby to increase complete (R0) resection rates and overall survival. At the time of the trial design, trastuzumab was not provided by the manufacturer; therefore, lapatinib was selected. We chose lapatinib, a dual inhibitor of epidermal growth factor receptor and HER2 available in an oral preparation, because it had shown activity in patients with HER2-positive advanced gastric cancer treated with chemotherapy.^6^ The primary objective of this ST03 lapatinib substudy was to assess the feasibility and safety of combining lapatinib with epirubicin, cisplatin, and capecitabine (ECX) chemotherapy in patients with resectable esophagogastric adenocarcinoma. If the pilot study was successful, the aim was to proceed to a registration trial using the same combination. This substudy was embedded within the MRC ST03 trial, Chemotherapy With or Without Bevacizumab or Lapatinib to Treat Operable Oesophagogastric Cancer, the full details of which are reported elsewhere.^7^

## Methods

### Study Design and Participants

This study followed the Consolidated Standards of Reporting Trials (CONSORT) reporting guideline. Eligibility for the lapatinib substudy was considered at 2 time points: before screening to establish *ERBB2/HER2* status, and before randomization of HER2-positive patients. Registration for *ERBB/HER2* testing took place from February 25, 2013, to April 19, 2016, and randomization took place between May 24, 2013, and April 21, 2016. Data were analyzed between May 10, 2017, and May 25, 2017. The study was part of the ST03 trial protocol (Supplement 1) and was approved by a national ethics committee and the United Kingdom (UK) Medicines and Healthcare Products Regulatory Agency (MHRA). Local approval was obtained at all participating centers. Written informed consent was provided by all participants before randomization.

Positivity for HER2 was assessed at a central location (Royal Marsden Hospital histopathology department) as a score of ≥3 on immunohistochemical staining or a score of ≥2 using the validated Ventana 4B5 antibody system with confirmation of *ERBB2/HER2* gene amplification by dual-color in situ hybridization and scored according to ToGA trial criteria.^6^ For in situ hybridization assessments, a HER2:CEP17 ratio of 2 or greater was defined as amplification. Local testing of *ERBB2/HER2* status was permitted if the local pathology laboratory met prespecified criteria (Supplement 1). After HER2 testing at participating centers , all cell blocks were recut and restained for *ERBB2/HER2* status confirmation at Royal Marsden Hospital.

After confirmation of HER2 positivity, full eligibility criteria for the study were evaluated before randomization. Eligible patients were aged 18 years or older with a diagnosis of treatment-naive, histologically confirmed, lower esophageal, gastric, or Siewert type 1, 2, or 3 esophagogastric junction adenocarcinoma. Patients with gastric and type III esophagogastric junction tumors staged as Ib (T1 N1, T2a/b N0), II, III, or stage IV (T4, N1 or N2 M0) by *AJCC Cancer Staging Manual*, sixth edition TNM criteria were eligible. Lower esophageal and type I or II esophagogastric junction tumors were staged according to the *AJCC Cancer Staging Manual*, seventh edition: 0 II*-IVa (*T2N1 not T2N0, T3N0-1, T4N0-1 M0-M1a). Full details can be found in the Trial Protocol (Supplement 1). The only major change made to the protocol after study commencement was to include normal QT duration as an inclusion criterion, owing to the effect of lapatinib on QT interval.

Patients were randomly assigned in a parallel design using 1:1 allocation and using minimization based on chemotherapy center, tumor site, tumor stage, and a random element (Figure). The randomization was performed centrally using a computerized algorithm developed and maintained by the Medical Research Council Clinical Trials Unit. The randomization was performed by research staff calling a central phone line.

**Figure.**
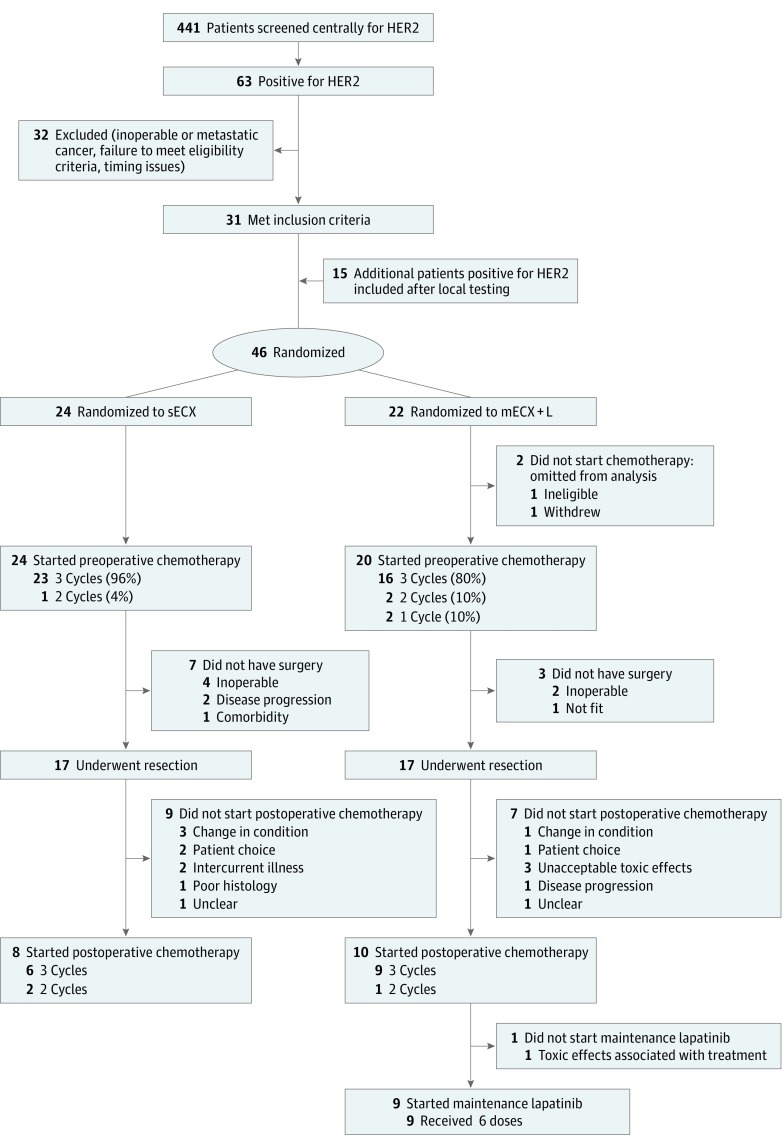
CONSORT Diagram ECX indicates epirubicin, cisplatin, and capecitabine; mECX+L, modified ECX plus lapatinib; sECX, standard ECX.

### Interventions

In the standard ECX (sECX) arm, ECX chemotherapy was administered in 3 preoperative and 3 postoperative 21-day cycles consisting of 50 mg/m^2^ epirubicin and 60 mg/m^2^ cisplatin, both intravenous, on day 1 and 1250 mg/m^2^ oral capecitabine on days 1 through 21. In the modified ECX (mECX) plus lapatinib (mECX + L) arm, patients were treated with ECX chemotherapy in 3 preoperative and 3 postoperative 21-day cycles, consisting of 50 mg/m^2^ intravenous epirubicin and 60 mg/m^2^ cisplatin on day 1 and 1000 mg/m^2^ oral capecitabine on days 1 through 21 plus 1250 mg of lapatinib taken orally on day 1 through 21 of each cycle, followed by maintenance dose of 1500 mg of lapatinib at taken orally daily for six 21-day cycles.

Postoperative sECX or mECX+L was commenced 6 to 10 weeks after surgery. Patients were followed up every 6 months after surgery for the first 3 years and every year thereafter until death, or at comparable time points if treatment was discontinued early. Chemotherapy was delivered by trained oncologists, research nurses, oncology nurses, and surgeons at UK hospitals.

### Outcomes

The primary objectives were to assess the safety of adding lapatinib to ECX chemotherapy and to establish a recommended dose of capecitabine and lapatinib for a subsequent phase 3 trial. Safety was chosen as an end point because increasing pathologic response rate would have required an excessive number of patients for the feasibility study. Secondary objectives included determination of the feasibility of centralized HER2 testing and confirmation of the proportion of HER2-positive cancers in patients with esophagogastric cancer. No changes to assessment of outcomes occurred after the feasibility trial commenced.

### Statistical Analysis

The sample size was determined pragmatically, considering what was likely to be feasible and would provide a reasonable level of evidence for this initial feasibility assessment. Randomization of 40 patients was expected to require screening of approximately 400 patients, which would allow the HER2-positive rate to be estimated with a reasonable level of accuracy. Twenty patients randomized to the mECX+L arm would facilitate an initial safety assessment. Twenty percent or less of the participants experiencing grade 3 or 4 diarrhea was deemed to be acceptable to patients and oncologists and, with the planned sample size, a 95% CI would exclude a 44% incidence of this adverse event, which was at the upper boundary of acceptability. The sample size was subsequently increased to ensure that 20 patients started treatment in the mECX+L arm and could contribute to the safety analysis.

To determine a recommended dose of capecitabine plus lapatinib in the mECX+L regimen, formal safety reviews were preplanned after 10 and 20 patients completed preoperative mECX+L. The following dose modification strategy was prespecified to be performed after review of the first 10 patients:

- If 1 patient or no patients had grade 3 or 4 diarrhea, the dose of capecitabine would be increased to 1250 mg/m^2^ for the next 10 patients.
- If 2 patients had grade 3 or 4 diarrhea, a further 10 patients would be treated at the same dose level; if no more than 4 of 20 had grade 3 or 4 diarrhea, this would be the recommended final dose
- If 3 or more patients had grade 3 or 4 diarrhea, the dose of capecitabine would be maintained at 1000 mg/m^2^ but the lapatinib dose would be reduced to 1000 mg/m^2^ for the next 10 patients.

The current analysis was performed from May 10, 2017, to May 25, 2017, approximately 1 year after the last patient entered the study after all patients had finished trial treatment (allowing a 30-day period after treatment for safety reporting).

Both the interim and final analyses were based on standard descriptive statistics, with 95% CIs where appropriate, but no formal hypothesis testing was performed. Baseline data are presented on an intention-to-treat basis; safety analyses are presented by randomized arm but are restricted to those patients who started treatment in the study. In addition, as detailed in the Results, there are some exclusions from the analysis owing to the availability of data: for example, those not undergoing surgery cannot be included in the assessment of postoperative complications and pathologic findings. At each of the formal reviews, an independent data monitoring committee considered the totality of evidence, including both descriptive statistics and detailed information on adverse events before making a recommendation. No formal hypothesis testing was performed. All analyses were carried out in Stata version 14 (StataCorp LLC).

## Results

Between February 25, 2013, and April 19, 2016, 441 patients from 29 UK centers underwent testing at the Royal Marsden Hospital for *ERBB/HER2* status (eFigure 1 in Supplement 2). The median (range) time between registration for HER2 testing and result reporting was 6 (1-31) days. The proportion of HER2-positive esophagogastric cancers based on central testing was 63 of 441 (14.3%; 95% CI, 11.2%-17.9%). This proportion does not include patients tested for HER2 at local centers, because these patients were only registered when identified as HER2-positive by the central laboratory. Of the 63 centrally tested HER2-positive patients, 31 were randomized. Reasons for HER2-positive patients not being randomized included inoperable or metastatic cancer, failure to meet full eligibility criteria, and timing of investigations or treatment. A further 15 patients were randomized after HER2 testing at 7 local centers; among these patients, 8 (53%) cancers were confirmed as HER2 positive after central testing.

Forty-six patients were enrolled; 24 were randomly assigned to receive sECX, and 22 were randomized to receive mECX+L. Baseline characteristics were similar between the 2 groups (Table). Median (IQR) age was 64 (56-69) years, 35 of 46 (76%) patients were male and 36 of the 46 (78%) had clinical stage III disease or greater at study entry.

**Table.** Baseline Patient Characteristics

Characteristic	No. (%)		
	sECX	mECX+L	Total
Male sex	21 (88)	14 (64)	35 (76)
Age, median (IQR) [range], y	65 (57-68) [45-78]	63 (54-71) [38-74]	64 (56-69) [38-78]
WHO performance status			
Normal activity	17 (71)	14 (64)	31 (67)
Restricted in physical activity	7 (29)	8 (36)	15 (33)
Site of tumor			
Lower esophageal	8 (33)	7 (32)	15 (33)
EGJ (type I)	4 (17)	5 (23)	9 (20)
EGJ (type II)	4 (17)	4 (18)	8 (17)
EGJ (type III)	4 (17)	2 (9)	6 (13)
Stomach	4 (17)	4 (18)	8 (17)
**Pretreatment Tumor Staging TNM6**			
Gastric and type III EGJ			
No.	8	6	14
T3 N0 M0 (stage II)	0 (0)	1 (17)	1 (7)
T3 N1 M0 (stage IIIa)	1 (13)	4 (67)	5 (36)
T3 N2 M0 (stage IIIb)	3 (38)	1 (17)	4 (29)
T4 N1-N2 M0 (stage IV)	4 (50)	0 (0)	4 (29)
Lower esophageal/type I or II EGJ			
No.	16	16	32
T3 N0 M0 (stage IIa)	0 (0)	4 (25)	4 (13)
T1 N1 M0 (stage IIb)	1 (6)	0 (0)	1 (3)
T2 N1 M0 (stage IIb)	4 (25)	0 (0)	4 (13)
T3 N1 M0 (stage III)	10 (63)	12 (75)	22 (69)
T4 N0-N1 M0 (stage III)	1 (6)	0 (0)	1 (3)
Total, No.	24	22	46

Abbreviations: ECX, epirubicin, cisplatin, and capecitabine; mECX+L, modified ECX plus lapatinib; EGJ, esophagogastric junction; sECX, standard ECX; WHO, World Health Organization.

The planned safety review, after 10 patients had completed preoperative treatment in the mECX+L arm, was performed in November 2014. No dose modifications were made as a result of this review (further details below). The final safety analysis, reported herein, once all patients had finished trial treatment and allowing a 30-day period after treatment for safety reporting.

Preoperative dose delays occurred in 12 of 71 (17%) sECX cycles involving 10 of 24 (42%) patients and in 8 of 54 (15%) mECX+L cycles involving 7 of 20 (35%) patients. Preoperative dose reductions occurred in 11 of 71 (15%) sECX cycles involving 9 of 24 (38%) patients and in 12 of 54 (22%) mECX+L cycles involving 9 of 20 (45%) patients. The leading cause of cycle delays and dose reductions was toxic effects (toxic effects details are provided in eTable 1 in Supplement 2). Four patients treated with preoperative mECX+L had their lapatinib dose reduced from 1250 mg/d to 1000 mg/d, with 1 patient having a further reduction to 750 mg/d. All dose reductions were because of diarrhea.

These data were not available for 1 patient receiving mECX+L who withdrew on day 1 of cycle 1 and was, therefore, excluded from this analysis. The most common preoperative toxic effect in both study arms was neutropenia, occurring in 10 of 19 patients (53%) in the mECX+L arm and 13 of 24 patients (54%) in the sECX arm; febrile neutropenia occurred in 1 patient treated with mECX+L. Grade 3 or 4 diarrhea occurred in 4 of 19 (21%; 95% CI, 6%-46%) patients treated with mECX+L but in none of the patients treated with sECX. Toxic effects more common in patients treated with preoperative mECX+L were grade 1 or 2 stomatitis, which occurred in 11 of 19 (58%) in the mECX+L arm vs 7 of 24 (29%) in the sECX arm, and vomiting of any grade, which occurred in 11 of 19 (58%) in the mECX+L arm vs 7 of 24 (29%) in the sECX arm. No patient died as a result of study treatment.

Ancillary analyses, which would not inform a definitive trial, included pathologic assessment and postoperative treatment tolerability. Pathology data were available for 16 patients in the sECX arm and 17 patients in the mECX+L arm. Local pathologic assessment of resection specimens showed a complete resection (R0) for 11 of 16 (69%) patients treated with sECX and 12 of 17 (71%) patients treated with mECX+L (eTable 2 in Supplement 2). At least 15 lymph nodes were evaluated by a pathologist in 14 of 16 (88%) patients treated with sECX and 14 of 17 (82%) patients treated with mECX+L.

One patient treated with sECX (6%) was found to have distant metastases at resection; this was not the case for any patient treated with mECX+L. Of 23 patients for whom a Mandard tumor regression grade was reported, 1 patient of 11 (9%) treated with sECX had a tumor regression grade of 1 to 2; this was true of 3 of 12 (25%) patients treated with mECX+L.

Postoperative data are available for all 34 patients (17 in each arm) who underwent surgical resection (eTable 3 in Supplement 2). Eight of the 17 (47%) of patients in the sECX arm and 10 of the 17 (59%) in the mECX+L arm experienced at least 1 complication. The incidence of anastomotic leak was similar in patients treated with mECX+L (2 of 17 [12%]) and sECX (3 of 17 [18%]). Two of 17 patients in the mECX+L arm (12%), required revision surgery (1 for ischemic bowel, 1 for chyle leak); no patient in the sECX arm had a second operative procedure.

Postoperative chemotherapy commenced after 8 weeks or later after surgery in 8 of 17 (47%) patients receiving sECX and 10 of 17 (59%) receiving mECX+L. Postoperative chemotherapy was delayed in at least 1 cycle for 5 of 8 (63%) patients receiving sECX and 6 of 10 (60%) receiving mECX+L. Five of 8 (63%) of patients in the sECX arm and 7 of 10 (70%) the mECX+L arm had at least 1 chemotherapy dose reduction postoperatively. Five of the 10 patients in the mECX+L arm (50%) had their lapatinib dose reduced during the postoperative phase.

The rate of toxic effects in the postoperative setting was similar to that recorded preoperatively (eTable 2 in Supplement 2). None of the patients had a clinically relevant drop in ejection fraction while receiving study treatment.

Five cases of grade 3 diarrhea occurred in patients treated with mECX+L. Four of these cases occurred preoperatively and were potentially related to treatment (4 of 19 [21%]; 95% CI, 6%-46%). One patient had grade 3 diarrhea postoperatively, which was deemed unrelated to treatment. At the first planned dose escalation review (based on the first 10 patients completing preoperative mECX+L), all feasibility data for the trial were reviewed by the independent data monitoring committee, and, although 3 of 10 patients (30%; 95% CI, 7%-65%) had experienced diarrhea events, the events in 2 patients were felt to be related to treatment. Because rates of other toxic effects such as neutropenia were also higher in the mECX+L arm, a decision was made not to escalate the dose of capecitabine at that point. Therefore, based on the prespecified dose escalation strategy, no escalation of mECX+L dose to 1250 mg/m^2^ of capecitabine was recommended during the study. The results presented herein conclude the second planned safety review.

Twelve patients treated in the study (6 per arm) died. All deaths were disease related.

## Discussion

In this analysis, we present the results of a randomized feasibility study of standard perioperative ECX chemotherapy vs dose-modified ECX plus lapatinib in patients with operable HER2-positive gastroesophageal adenocarcinoma. This is, to the best of our knowledge, the first report on a completed phase II randomized trial of HER2-directed therapy in operable gastroesophageal cancer. We initiated a screening program for *ERBB/HER2* status in gastroesophageal cancer at 29 UK centers and centrally screened 441 patients and confirmed a HER2-positive rate of 14% in this UK cohort. Forty-six patients were treated in the study. We found that, even with a modification of the capecitabine dose, the addition of lapatinib to mECX resulted in clinically significant diarrhea in 5 of 22 (21%) of patients treated, which was higher than the rate we considered acceptable for patient safety. For this reason, it is suggested that any future trials combining these compounds should not escalate the capecitabine dose beyond 1000 mg/m^2^/d.

The primary aim of this study was to establish a safe and tolerable regimen for lapatinib combined with ECX chemotherapy in patients with gastroesophageal cancer to inform the design of future studies. In addition to the gastrointestinal toxic effects observed in our trial and lack of observed increase in efficacy with respect to pathologic response rates, the recent results from the Fluorouracil plus leucovorin, oxaliplatin and docetaxel 4 Arbeitsgemeinschaft Internistische Onkologie (FLOT4 AIO) trial^3,7^ demonstrating better outcomes with FLOT (fluorouracil, oxaliplatin, leucovorin, and docetaxel chemotherapy compared with previous trials with ECX, and the negative results of the TRIO-013 (Translational Research in Oncology)/LOGiC (Lapatinib Optimization Study in the HER2-Positive Gastric Cancer) trial, in which lapatinib was combined with capecitabine and oxaliplatin, and the Lapatinib [Tykerb] with Paclitaxel [Taxol] in Asian ErbB2+[HER2+] Gastric Cancer Study, (TyTAN ) trial, in which lapatinib was combined with paclitaxel in the metastatic setting, mean that it is unlikely to be feasible to develop a lapatinib plus ECX combination in this patient group.^8,9^

The secondary objectives of this trial were to assess the feasibility of central biomarker assessment for a national trial in the perioperative setting. We observed 14.3% of patients to have HER2-positive cancers according to study criteria, which is consistent with other series and may inform future trial design.^10,11^ Central testing of *ERBB/HER2* status was felt to be necessary owing to the special laboratory requirements for HER2 testing in gastroesophageal cancer, which were not available in many UK centers at the time of study design. The median turnaround time for centrally tested HER2 results was 6 days, which did not introduce any undue delay into the treatment pathway and was satisfactory for patients and investigators. During the course of the study, several major centers achieved nationally recognized certification for HER2 testing and were therefore given permission to perform local HER2 testing. Although local testing for study entry can increase recruitment, central confirmation of biomarker status is likely to be required for any registration trial. This is particularly important in view of the heterogeneity of *ERBB/HER2* status observed in gastroesophageal cancer, and highlighted by the discrepancies between local and central testing results in our data set.^4^ Half of patients who had HER2-positive tumors were not randomized, predominantly owing to ineligibility. To permit screening of the largest proportion of patients within the short perioperative time frame, a permissive approach to prescreening eligibility was used, followed by full eligibility assessment after HER2 positivity had been established. Higher rates of screen failure might be expected when following such an approach.

Neither the feasibility of surgery nor surgical morbidity were compromised by the addition of lapatinib to mECX chemotherapy. A previous trial using lapatinib in conjunction with capecitabine and oxaliplatin chemotherapy was halted because of anastomotic leaks in 2 patients; no increase in anastomotic leaks was observed in lapatinib-treated patients in our study.^12^ The rate of R0 resection was comparable in both arms of this study and consistent with that demonstrated in the phase 3 randomized ST03 ECX plus or minus bevacizumab comparison.^13^ After local pathologic review, complete tumor regression rates (25%) were encouraging in patients who received mECX+L; however, this assessment is based on 23 patients.

### Limitations

One limitation of the current study is its relatively small size. However, in the context of a national biomarker selected trial performed in the perioperative setting, which is subject to time constraints, recruitment appears more impressive. The use of what is now considered a less-optimal chemotherapy regimen and HER2-targeted treatment in this patient group may be potentially subject to criticism. At the time of trial design, ECX was a recommended standard of care; and recruitment was completed before emerging data about the FLOT regimen. Adoption of FLOT as the new perioperative standard offers an excellent opportunity to combine HER2-directed therapy with a regimen that contains no potentially cardiotoxic agents, unlike ECX. This is currently being investigated in the ongoing Neoadjuvant Study Using Trastuzumab or Trastuzumab With Pertuzumab in Gastric or Gastroesophageal Junction Adenocarcinoma (EORTC INNOVATION) and FLOT vs FLOT/Herceptin/Pertuzumab for Perioperative Therapy of HER-2 Expressing Gastric or GEJ Cancer (AIO PETRARCA) perioperative studies. These trials are important, because although the addition of pertuzumab to trastuzumab plus chemotherapy did not improve overall survival in the JACOB trial (A Study of Pertuzumab in Combination With Trastuzumab and Chemotherapy in Participants With Human Epidermal Growth Factor Receptor 2 [HER2]-Positive Metastatic Gastroesophageal Junction or Gastric Cancer in HER2-Positive Advanced Gastric Cancer), an improvement in radiologic response rates was observed.^14^ Other limitations include potential bias in patient selection, remaining uncertainty surrounding feasibility, and, potentially, multiplicity.

## Conclusions

Although the external validity of the study may be limited by the use of lapatinib when more effective HER2-targeting drugs have now been developed, the trial design and structures were successful and could be used in the future for other similar trials in this setting. To capitalize on the work conducted in the current study, a translational protocol, which will include genomic, transcriptomic, and proteomic analysis of HER2-associated signaling pathways is planned. It is hoped that the results of this program will help to elucidate further opportunities for targeting HER2 in this challenging heterogenous disease.

## Notes

Supplement 1. Trial Protocol.

Supplement 2. **e Figure 1.** HER2 Testing Flowchart **eTable 1.** Preoperative Toxicity **eTable 2.** Pathological Assessment **eTable 3.** Postoperative Complications **eTable 4.** Postoperative Toxicity

Supplement 3. Data Sharing Statement
