# Supplementary material for: Safety and Efficacy of the Addition of Lapatinib to Perioperative Chemotherapy for Resectable HER2-Positive Gastroesophageal Adenocarcinoma: A Randomized Phase 2 Clinical Trial
Source: JAMA Oncol. 2019 Jun 20;5(8):1181–7. doi: 10.1001/jamaoncol.2019.1179 (PMC6587151; doi:10.1001/jamaoncol.2019.1179)
Supplement: Supplement 1. — Trial Protocol. [file jamaoncol-5-1181-s001.pdf]

Developed on behalf of the NCRI Upper GI Clinical Studies Group

Part of the National Cancer Research Network Portfolio

ST03

A Randomised Phase II/III trial of Peri-operative Chemotherapy with or without Bevacizumab in Operable Oesophagogastric Adenocarcinoma
and
A Feasibility Study Evaluating Lapatinib in HER-2 Positive Oesophagogastric Adenocarcinomas
And (in selected centres)
MRI and PET/CT Sub-Studies

Protocol Version 7, 17th February 2014

ISRCTN: 46020948

EUDRACT number: 2006-000811-12

CTA: 00316/0221/001

Authorised by:

Chief Investigator
Professor David Cunningham

Signature……………………………
Date: 17/02/2014

MRC CTU Lead
Dr Ruth Langley

Signature……………………………
Date: 17/02/2014

This protocol describes the ST03 lapatinib feasibility study, MRI sub-study and PET/CT sub-study.

Appendix T of this protocol describes the original bevacizumab comparison of the ST03 trial which is now closed to recruitment. For treatment and assessment of patients within the bevacizumab comparison only please refer to Appendix T.

GENERAL INFORMATION

This document describes the ST03 trial coordinated by the Medical Research Council Clinical Trials Unit at University College London (MRC CTU) and provides information about procedures for entering patients into the trial. The protocol should not be used as an aide-memoir or guide for the treatment of other patients. Every care has been taken in drafting this protocol, but corrections or amendments may be necessary. These will be circulated to the registered Principal Investigators (PI) in the trial, but centres entering patients for the first time are advised to contact the ST03 Trial Manager at the, MRC CTU to confirm they have the most up to date version.

Compliance

The trial will be conducted in compliance with the protocol, the Data Protection Act (DPA number: Z5886415), National Health Service (NHS) research governance and other regulatory requirements, as appropriate. MRC CTU shall be responsible for the operational management of ST03 at the participating Clinical Sites in UK and shall do so in compliance with the principles of Good Clinical Practice (GCP) as laid down by Commission Directive 2005/28/EC and by Statutory Instrument 2004/1031 [Amendments; 2006 No. 1928]. International sites shall be responsible for the operational management of ST03 at their participating Clinical Sites and shall do so in compliance with the principles of Good Clinical Practice (GCP) as laid down by the ICH topic E6 (Note for Guidance on GCP), or Commission Directive 2005/28/EC (where applicable) and in accordance with applicable local laws or regulations.

Sponsor

the trial is sponsored by the MRC and organised by the MRC Clinical Trials Unit (MRC CTU) at University College London. The managing organisation is the University College London (UCL). For any issues relating to Sponsorship, please contact Professor Max Parmar, Director, MRC CTU, Aviation House, 125 Kingsway, London, WC2B 6NH.
Email: m.parmar@ucl.ac.uk

Funding

The trial is funded by Cancer Research UK, Clinical Trials Awards Advisory Committee (CTAAC) Grant No: C1504/A6410, CRUK Trial No: CRUK/06/025 and supported by the MRC CTU.

F Hoffmann-La Roche Limited, Basel support this trial by providing free bevacizumab, an educational grant to cover the costs of the additional cardiac investigations for the first 200 patients and provide centres with per patient payments for all patients participating in the bevacizumab comparison of the trial.

GlaxoSmithKline have agreed to support the lapatinib feasibility study by providing free lapatinib, an educational grant to cover the costs of HER-2 testing for all patients screened for the feasibility study, the additional cardiac investigations for 40 patients with HER-2 positive tumours entered into the feasibility study and provide centres with per patient payments for all HER-2 positive patients participating in the lapatinib feasibility study.
Please see section 29 for further details on funding.

REGISTRATIONS AND RANDOMISATIONS
To randomise call MRC CTU, Monday to Friday 0900-1700
Tel: 020 7670 4777

SAE REPORTING
Fax: 020 7670 4818

ST03 Trial Summary

The original ST03 trial was a phase II/III trial evaluating the addition of bevacizumab to peri-operative ECX chemotherapy in operable oesophagogastric adenocarcinomas.

In addition to the Bevacizumab comparison, the trial encompasses 3 sub-studies:

1. **ST03 Lapatinib Feasibility Study**
2. **ST03 MRI Sub-study**
3. **ST03 PET/CT Sub-study**

Recruitment to the bevacizumab comparison is now complete.
Recruitment into all 3 sub-studies remains ongoing.

<u>ST03 Protocol Version 7.0 should be used:</u>

1. **For the treatment and follow up of all patients randomised to the bevacizumab comparison.**

   For these patients please refer to:

   - Appendix T for instruction on treatment and assessments
   - Section 16 onwards of the main body of the protocol for instruction on surgery, safety procedures, pathology and follow up.

And

2. **For the registration, randomisation, treatment and follow up of the ST03 lapatinib feasibility study, MRI and PET/CT sub-studies.**

   Please refer to the main body (section 1 onwards) of the protocol for instruction on all procedures relating to these studies.

Trial Administration

Chief Investigator

Professor David Cunningham
Dept of Oncology
Royal Marsden Hospital
Downs Road, Sutton
Surrey SM2 5PT

Tel: 020 8661 3156
Fax: 020 8643 9414
Email: David.Cunningham@rmh.nhs.uk

Coordinating Centre

MRC CTU
Institute of Clinical Trials & Methodology
Aviation House
125 Kingsway
London WC2B 6NH

Switchboard: 020 7670 4700
Fax: 020 7670 4818
Email: ST03@ctu.mrc.ac.uk

MRC CTU Staff

**Trial Managers:**	**Claire Robb**<br>Tel: 0207 670 4804<br>Email: c.robb@ucl.ac.uk	**Rahela Choudhury**<br>Tel: 0207 670 4801<br>Email: rahela.choudhury@ucl.ac.uk
**Data Managers:**	**Linda Ly**<br>Tel: 0207 670 4922<br>Email: linda.ly@ucl.ac.uk	**Leah Meaden**<br>Tel: 0207 670 4776<br>Email: l.meaden@ucl.ac.uk
Clinical Trial Project Manager:	*Lizzie Armstrong*	*Tel: 0207 670 4812*
Senior Statistician:	*Prof Sally Stenning*	*Tel: 0207 670 4707*
Statistician:	*Sam Rowley*	*Tel: 0207 670 4854*
MRC CTU Lead:	*Dr Ruth Langley*	*Tel: 0207 670 4718*

For general ST03 queries please contact the ST03 Trial Manager. All Clinical queries will be referred to the Chief Investigator via the Trial Manager.

Co-Investigators:

Professor Derek Alderson
Surgeon: Queen Elizabeth Hospital

Mr William Allum
Surgeon: Royal Marsden Hospital

Professor Jane Blazeby
Surgeon and QoL Advisor: Bristol Royal Infirmary

Dr Tom Crosby
Oncologist: Velindre Hospital

Mr Robert Mason
Surgeon: St Thomas' Hospital

Dr Kate Sumpter
Oncologist: Freeman Hospital, Newcastle

Dr Heike Grabsch
Pathologist: St James's University Hospital

Dr Angela Riddell
Radiologist: Royal Marsden Hospital

Professor Michael Griffin
Surgeon: Royal Victoria Infirmary

Dr Sue Chua
Radiologist: Royal Marsden Hospital

Advisors:

Professor Mark Sculpher
Health Economics Advisor: York

Dr Elizabeth Smyth
Trial Physician: Royal Marsden Hospital

Dr Chris Plummer
Cardiologist: Freeman Hospital, Newcastle

For full details of all trial committees please see Appendix O.

CONTENTS

1. ST03 Trial Summary 2

2. Background and Rationale 6

2.1 Introduction 7
2.2 Final Results of the MAGIC Trial 7
2.3 Evidence for the Use of Capecitabine 7
2.4 Evidence for the Use of Bevacizumab 8
2.5 Evidence for the use of lapatinib 8
2.6 Evidence for the use of lapatinib maintenance therapy 10
2.7 Evidence for use of MRI as a predictive biomarker for resectability 10
2.8 Evidence for use of PET/CT as a surrogate biomarker for predication of treatment response 11

3. Trial Design and Objectives 13

3.1 Trial Design 13
3.2 Trial Objectives 14
3.3 Endpoints 14

4. Centre Enrolment and Accreditation Process 15

4.1 Selection of Trial Centres/Clinicians 15
4.2 Accreditation Process 16
4.3 MHRA CTA Approval for Investigators Participation in ST03 16
4.4 Centres Participating in the Lapatinib Feasibility Study 17
4.5 Centres participating in the MRI sub-study 17
4.6 Centres participating in the PET/CT sub-study 18

5. Registration and HER-2 Testing for the Lapatinib Feasibility Study 21

5.1 Registration Eligibility Requirements 21
5.2 Consent for HER-2 Sample testing 21
5.3 Registration 22
5.4 Block Request and Dispatch Procedure 22
5.5 HER-2 Testing at the RMH laboratory 24
5.6 Local HER-2 Testing 24
5.7 Patient Screening Whilst Awaiting HER-2 Test Result 25
5.8 HER-2 Results 25

6. Selection of Patients for the Lapatinib Comparison 26

6.1 Inclusion Criteria for Patients with HER-2 Positive Tumours Entering the Lapatinib comparison 26
6.2 Exclusion Criteria for Patients with HER-2 Postive Tumours Entering the Lapatinib Comparison 29
6.3 Guidance on Concomitant Medication for Patients with HER-2 Positive Tumours Entering the Lapatinib Comparison 30
6.4 Pre-Randomisation Investigations and Screening Procedures 31

7. Randomisation of Patients with HER-2 Positive Tumours 33

7.1 Written Informed Consent 33
7.3 Randomisation Procedure for Patients Entering the Lapatinib Comparison 34
7.4 Patient Screening and Enrolment Log 35
7.5 Co-enrolment Guidelines 35

7.6 Trans-ST03: Blood and Tissue Collection for Translational Research ....**Error! Bookmark not defined.**

8. Treatment of Patients 35
8.1 General Principles 35
8.1.1 Nadir Blood Test... 36
8.1.2 Cardiac Monitoring and treatment modifications 37
8.1.3 Measures of Compliance/Adherence... 37
8.1.4 Capecitabine & Lapatinib Diary Cards.. 38
8.2 Administration of Control Arm C: Standard sECX.. 38
8.3 Toxicities and Dose Modifications for Standard sECX..................................... 39
8.4. Administration of the Investigational Arm D: mECX+L 45
8.4.1 Safety assessment and criteria for dose level escalation or reduction 45
8.4.2. mECX Capecitabine dose level 0... 46
8.4.3. Administration of Lapatinib 46
8.4.4. Problems Administering Capecitabine or lapatinib 46
8.4.5. Intervals between peri-operative treatment and surgery 47
8.4.6. Lapatinib maintenance therapy 47
8.5. Toxicities and dose modifications for lapatinib.. 47
8.5.3 Specific toxicities requiring discontinuation of lapatinib 52

9. Assessments and Procedures within the lapatinib Comparison............... 54
9.1 Assessments Required Prior to Each Cycle of Treatment with sECX or mECX+L 54
9.2. Assessments Prior to Surgery within the lapatinib Comparison...................... 54
9.3. Assessments after Completion of Post-Operative Chemotherapy within the Lapatinib Comparison... 56
9.4 Time Point for CRF Return Schedule 60

10. Selection of Patients for the MRI and/or PET/CT Substudies................ 62
10.1. Inclusion Criteria For Patients to be Registered into the MRI and/or PET-CT Sub-studies ONLY... 62
10.2. Exclusion Criteria for Patients to be Registered into the MRI and/or PET-CT Sub-studies ONLY... **Error! Bookmark not defined.**
10.3 Pre-Registration Investigations and Screening Procedures for Patients Registering for the MRI and/or PET/CT Sub-studies ONLY 63

11. Registration into the MRI and/or PET/CT Sub-studies ONLY 64
11.1 Written Informed Consent 65
11.2 Procedure for Registration into the MRI and PET/CT sub-studies.................. 66

12. Treatment of Patients Registered to MRI and/or PET/CT Sub-studies Only 67

13. Assessments and Procedures within the MRI and/or PET/CT Sub-studies Only 67
13.1 Baseline MRI and PET/CT Scans Prior to Commencing Treatment 67
13.2 Assessments Required Prior to Each Cycle of Treatment 67
13.3 Post Cycle 1 PET/CT Scan... 68
13.4 Assessments Prior to Surgery 68

14. MRI Sub-study Procedures... 73
14.1 Timing of the MRI Scans 73
14.2 MRI Sub-study Trial Schema.. 74
14.3 MRI Sequence.. 75
14.4 Data Collection & Storage... 76

14.5 Finance ... 77

15. PET/CT Sub-study Procedures ... 78
15.2 Timing of Scans ... 78
15.3.PET/CT Sub-study Trial Schema ... 79
15.4 Data Collection & Storage ... 79
15.5 Finance ... 80
15.6 Publication Policy for the PET/CT Sub-study ... 80

16. Surgery – FOR ALL PATIENTS ... 81
16.1 Surgical Procedures ... 81
16.2 Complications with surgery ... 84
17.1 Definitions of Adverse Events/Reactions ... 86
17.2 Severity/grading of adverse events ... 86
17.3 SAE Events Excluded from Expedited Notification ... 87
17.4 ST03 Additional Notable Events that Require Expedited Reporting for Patients within the sECX+/-B Comparison (Arms A and B) ... 87
17.5 ST03 Additional Notable Events that Require Expedited Reporting for Patients within the sECX Vs mECX + L Comparison (Arms C and D) ... 88
17.6 Thromboembolic events and ECX chemotherapy ... 88
17.8 SAE Notification Procedures By Investigator ... 89
17.9 MRC CTU Responsibilities ... 90
17.10 Follow-up after adverse events ... 91

18. Patient Follow-Up ... 93

19. Stopping Trial Treatment ... 93

20. Patient Moves to New Centre ... 93

21. Trial Closure ... 94
21.1 Site closure procedures ... 94

22. Archiving ... 94

23. Withdrawal of Patients ... 94

23.1 Withdrawal of Consent ... 95

24. Statistical Considerations ... 96
24.1 Method of Randomisation ... 96
24.2 Outcome Measures ... 96
24.3 Sample Size ... 98
24.4 Interim Monitoring and Analyses ... 99

25. Trial Monitoring ... 102
25.1 Risk Assessment ... 102
25.2 Monitoring at the MRC CTU ... 102
25.3 Clinical Site Monitoring ... 102
25.4 Confidentiality of Trial Documents and Patient Records ... 102
25.5 Criteria for Stopping the Trial ... 103

26. Ethical Considerations and Approval ... 104
26.1 Ethical considerations ... 104
26.2 Ethical approval ... 105

27. Regulatory Approval 105
29. Finance 106

30. Trial Committees 107
30.1 Trial Management Group (TMG) 107
30.2 Trial Steering Committee (TSC) 107
30.3 Independent Data Monitoring Committee (IDMC) 107

31. Ancillary Studies 108
31.1 Rationale for QoL Study 109
31.2 Quality of Life Measures 109
31.3 Administration of QoL Questionnaires 109
31.4 Radiology Review 110
31.5 Health Economics 110
31.6 MRI Sub-Study 110
31.7 FDG-PET Sub-study 111

32. ST03 Pathology Research 112
32.1 ST03 Central Pathology Review 112
32.2 Trans-ST03: Tissue and Blood Collection for Translational Research 114
32.3 Trans-ST03 Funding 117
32.4 Centres participating in the MRI sub-study 117
32.5 117
Summary of the Destination Pathology Research Samples in the ST03 Trial: 117
33. Publication Policy 118

34. Protocol Amendments 118

35. References 123

Appendices 131

Appendix A: Additional Definitions 131
A1: WHO Performance Status 131
A2a: TNM Staging (6th ed.): Gastric/type III OGJ Tumours 131
A2b: TNM Staging (6th ed.): Lower oesophageal/type I-II OGJ Tumours 131
A3: New York Heart Association (NYHA) Classification 132

Appendix B: Patient Information Sheets 133

Appendix C: Consent Forms 134

Appendix D: GP Letters 135

Appendix E: Guidance on Treating Hypertension 136

Appendix E(i): SAMPLE GP Letter – For Patients that Develop Hypertension 137

Appendix E (ii) ST03 Trial Blood Pressure Monitoring Form 139

Appendix F: Modified RECIST Version 1.0 Response Definitions 140

Appendix G: CTCAE Toxicity Criteria 142

Appendix H: Surface area Nomogram 146

Appendix I: Cockcroft and Gault Formula 147

Appendix J: Drug Supply Information..148

Appendix K: How to calculate the number of capecitabine tablets to dispense following a dose reduction..151

Appendix L: Summary of Products Characteristics –Undesirable Effects...153

Appendix M: TNM 7th edition staging criteria 154

Appendix N: Algorithm for cardiac monitoring requirements for patients randomised to the lapatinib comparison (Arms C and D) only.....155

Appendix O: ST03 Trial Committees 157

Appendix P - Health Economics..159

Appendix Q: ST03 HER-2 Testing and Sample Handling Procedure 161

APPENDIX R – Capectabine and Lapatinib Modification and Dose Levels ..163

Appendix S: PET/CT Sub-study Scanning Procedures..............................164

Appendix T – ST03 Original Trial Bevacizumab Comparison Now Complete..182

1. ST03 Trial Summary 182
2. Background and Rationale for the use of Bevacizumab 184
3. Trial Design and Objectives for the Bevacizumab Comparison.........................187
4. Selection of Patients...190
5. Randomisation...195
6. Treatment of Patients...197
7. General Information on Adverse Events Associated with Bevacizumab.............207
8. Assessments and Procedures required within the sECX+/-B Comparison..........216
9. Statistical Considerations for the Bevaciuzmab Comparison............................222

ABBREVIATIONS AND GLOSSARY

**ADC**	Apparent Diffusion Coefficient
**AE**	Adverse Event
**AR**	Adverse Reaction
**ANC**	Absolute Neutrophil Count
**APTT**	Activated Partial Thromboplastin Time
**ASCO**	American Society of Clinical Oncology
**AUC**	Area Under Curve
**B**	Bevacizumab
bid	Twice a day *(bis in die)*
**BP**	Blood pressure
**BSA**	Body surface area
**CERES**	Consumers for Ethics in Research
**CF**	Consent Form
**CI**	Chief Investigator
**CI**	Confidence interval
**CRF**	Case Report Form
**CRM**	Circumferential Resection Margin
**CRUK**	Cancer Research UK
**CT**	Computerised tomography
**CTA**	Clinical Trials Authorisation
**CTAAC**	Clinical Trials Advisory and Awards Committee
**CTC**	Common Toxicity Criteria
**CTU**	Clinical Trials Unit
**CXR**	Chest X-ray
**DCF**	Data Clarification Form
**DDISH**	Dual colour Dual hapten In Situ Hybridisation
**DNA**	Deoxyribonucleic Acid
**DPA**	Data Protection Act
**DPD**	Dihydropyrimidine dehydrogenase
**DWI**	Diffusion Weighted Imaging
**ECF**	Epirubicin, Cisplatin and 5-Fluorouracil
**ECG**	Electrocardiogram
**ECHO**	Echocardiogram
**ECX**	Epirubicin, Cisplatin, Xeloda (capecitabine)
**ECX+B**	Epirubicin, Cisplatin, Xeloda (capecitabine) and Bevacizumab
**ECX+L**	Epirubicin, Cisplatin, Xeloda (capecitabine) and Lapatinib
**EDTA**	Ethylenediaminetetraacetic acid
**EMEA**	European Medicines Agency
**ESMO**	European Society for Medical Oncology
**EGFR**	Epidermal Growth Factor Receptor
**EOX**	Epirubicin, Oxaliplatin, Xeloda (capecitabine)
**EUS**	Endoscopic Ultrasound
**FBC**	Full Blood Count
**FEV1**	Forced expiratory volume in one second
**FFPE**	Formalin Fixed Paraffin Embedded
**FISH**	Fluorescent In Situ Hybridisation
**G-CSF**	Granulocyte-colony stimulating factor (G-CSF)
**GCP**	Good Clinical Practice
**GFR**	Glomerular Filtration Rate
**GP**	General Practitioner
**GRO**	General Register Office
**HE**	Health Economics

**hr**	Hour
**HER-2**	Human Epidermal Growth Factor Receptor-2
**HR**	Hazard Ratio
**ICH**	International Conference on Harmonization
**IDMC**	Independent Data Monitoring Committee
**IHC**	Immunohistochemistry
**IM**	Intramuscular
**INR**	International Normalised Ratio
**ISRCTN**	International Standard Randomised Controlled Trial Number
**IU**	International Units
**IV**	Intravenous
**L**	Lapatinib
**LD**	Longest diameter
**LFT's**	Liver Function Tests
**LLN**	Lower Limit of Normal
**LREC**	Local Research Ethics Committee
**LVEF**	Left Ventricular Ejection Fraction
**mCRC**	Metastatic Colorectal Cancer
**mECX**	Modified ECX
**MDT**	Multi Disciplinary Team
**MHRA**	Medicine and Healthcare Products Regulatory Agency
**min**	Minutes
**MRC**	Medical Research Council
**MRC CTU**	Medical Research Council Clinical Trials Unit at University College London
**MREC**	Multi-Centre Research Ethics Committee
**MRI**	Magnetic resonance imaging
**MUGA**	Multiple Gated Acquisition scan
**NCI**	National Cancer Institute (USA)
**NCRI**	National Cancer Research Institute
**NCRN**	National Cancer Research Network
**NHS**	National Health Service
**NICE**	National Institute of Clinical Excellence
**NSAID**	Non-Steroidal Anti-inflammatory Drugs
**NYHA**	New York Heart Association
**ONS**	Office for National Statistics
**OGJ**	Oesophagogastric Junction
**OS**	Overall Survival
**PET**	Positron Emission Tomography
**PFS**	Progression Free Survival
**PI**	Principal Investigator
**PIS**	Patient Information Sheet
**PPI**	Proton Pump Inhibitor
po	*per orum* (orally)
**QALY**	Quality-adjusted Life Years
qds	*quater die sumendus* (4 times each day)
**QoL**	Quality of Life
**R and D**	Research and Development
**RECIST**	Response Evaluation Criteria In Solid Tumours
**RMH**	Royal Marsden Hospital
**SAC**	Sample Access Committee
**SAE**	Serious Adverse Event
**SAR**	Serious Adverse Reaction
sc	*Sub-cutaneous* (under skin)
**SCC**	Squamous Cell Carcinoma
**sECX**	Standard ECX
**SPC**	Summary of Product Characteristics
**SSA**	Site Specific Approval

**SUSAR**	Suspected Unexpected Serious Adverse Reactions
**SUV**	Standardised Uptake Value
tds	Three times a day
**TIA**	Transient Ischemic Attack
**TMG**	Trial Management Group
**TNM**	Tumour, Nodes, Metastases
**TSC**	Trial Steering Committee
**ULN**	Upper Limit of Normal
**UPCR**	Spot urine protein:creatinine ratio
**U+E**	Urea and Electrolytes
**UCL**	University College London
**VEGF**	Vascular Endothelial Growth Factor
**VEGFR-2**	Vascular Endothelial Growth Factor Receptor-2
**WHO**	World Health Organisation
**X**	*Xeloda* (Capecitabine)
**5-FU**	5-Fluorouracil

Figure 1. Overview of Current ST03 Trial Design

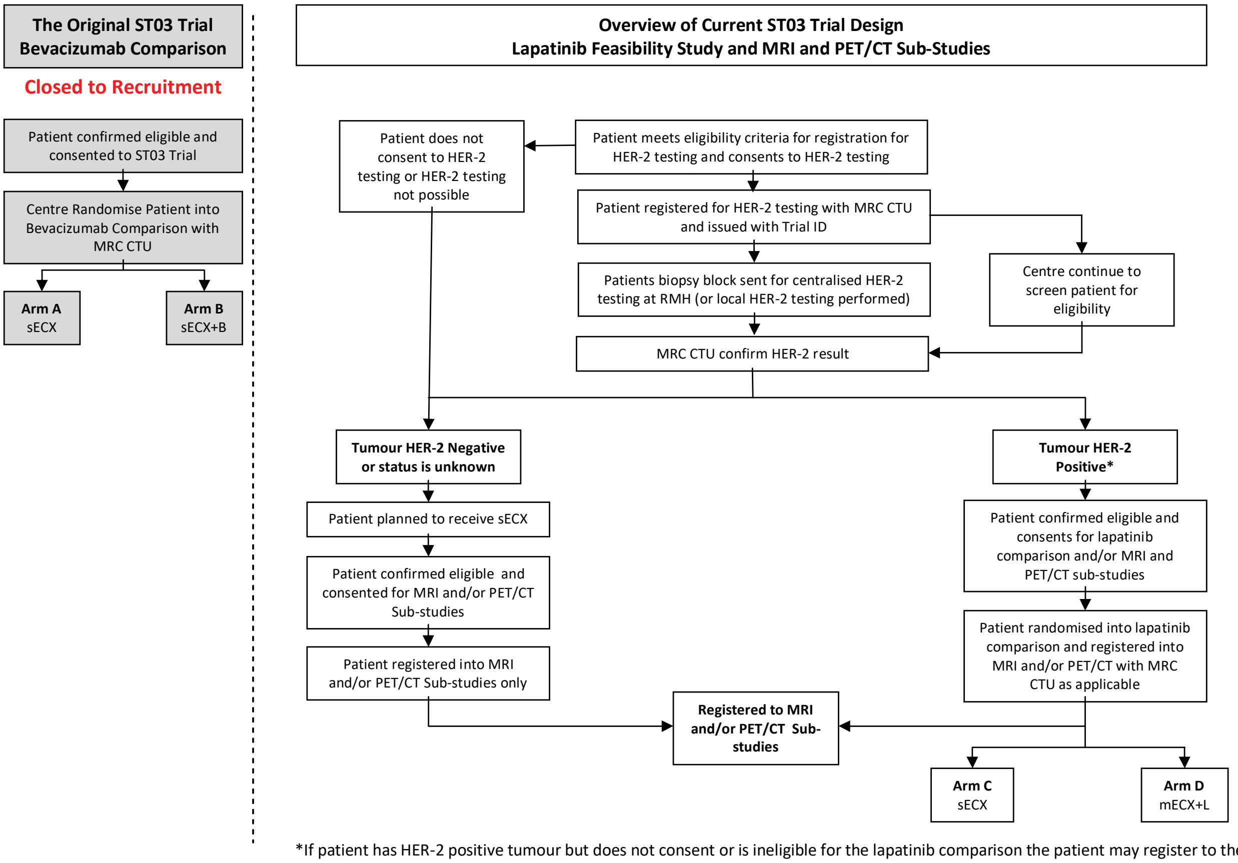

*If patient has HER-2 positive tumour but does not consent or is ineligible for the lapatinib comparison the patient may register to the MRI and/or PET/CT sub-studies only if eligible.

1. ST03 Trial Summary

1.1 Type of design

The original ST03 trial was a randomised, multi-centre, open-label phase II/III trial designed to evaluate the safety and efficacy of adding bevacizumab (B), a humanised monoclonal antibody against vascular endothelial growth factor (VEGF), to peri-operative standard ECX (sECX) chemotherapy for patients with Human Epidermal Growth Factor Receptor-2 (HER-2) negative or unknown operable oesophagogastric adenocarcinoma.

In October 2013, based on a review of cumulative data from the ST03 trial bevacizumab comparison by the Independent Data Monitoring Committee (IDMC), an Urgent Safety Amendment was implemented to amend the inclusion criteria to exclude patients with lower oesophageal, Siewert Type I, II or III Oesophagogastric junction (OGJ) adenocarcinomas from entering the bevacizumab comparison. The comparison remained unchanged for patients with gastric adenocarcinomas who did not require an oesophagogastrectomy. This Urgent Safety Amendment also mandated that patients with lower oesophageal, Siewert Type I, II or III OGJ adenocarcinomas who were already entered into the sECX+B arm (Arm B) must not receive any further pre-operative bevacizumab. Following surgery patients may resume treatment with bevacizumab provided that there are no other contraindications.

Further details of the reasons for this change to eligibility criteria are given in Section 2.4 of Appendix T.

Recruitment to the bevacizumab comparison is now complete, for further details on the original ST03 trial design, background information and for the treatment of patients in the bevacizumab comparison please refer to Appendix T. Information on surgery, safety, pathology and follow up can be found in section 16 onwards.

The ST03 trial encompasses 3 sub-studies which are still ongoing. These sub-studies are summarised below and detailed within the main body of the protocol (from this section onwards).

1.1.1 ST03 Lapatinib Feasibility Study

The ST03 lapatinib feasibility study is a randomised, multi-centre, open label phase II trial designed to evaluate the safety and feasibility of adding lapatinib (L), a small molecule tyrosine kinase inhibitor of HER-2 and epidermal growth factor receptor (EGFR), to peri-operative modified ECX (mECX) chemotherapy for patients with HER-2 positive operable oesophagogastric adenocarcinoma.

This feasibility study will evaluate the feasibility of timely HER-2 testing and the safety and feasibility of adding lapatinib to mECX chemotherapy in patients with HER-2 positive tumours. It will also assess the rates of HER-2 positivity in this study population which may be used to inform the design of a larger phase III study.

1.1.2 ST03 MRI Sub-study

The ST03 MRI sub-study is an observational registration only sub-study designed to assess the potential benefit of MRI in the selection of patients for surgery based on the ability of MRI to predict patients who are likely to have a positive circumferential resection margin (CRM). The sub-study will use T2 weighted high resolution MRI as a predictive biomarker for resectability in patients with operable lower oesophageal, Type I and II oesophagogastric junction (OGJ) adenocarcinoma.

The MR imaging data will be collected prospectively but **not** used to inform patient management.

1.1.3 ST03 PET/CT Sub-study

The ST03 PET/CT sub-study is an observational registration only sub-study designed to evaluate the role of ^{18}FDG-PET/CT as a surrogate biomarker for prediction of treatment response and prognosis, following the first cycle of neoadjuvant chemotherapy in patients with operable oesophagogastric adenocarcinoma.

The PET imaging data will be collected prospectively but **not** used to inform patient management.

See Figure 1 for an overview of the current ST03 Trial design.

1.2 Eligible Patients

1.2.1 ST03 Lapatinib Feasibility Study

Patients with histologically confirmed lower oesophageal, gastric, Siewart Type I, II or III OGJ adenocarcinoma who have not received any previous treatment for their cancer, have no metastasis (confirmed by CT) and in whom it is believed an R0 resection can be achieved, will be eligible for registration for HER-2 testing provided that they give consent for HER-2 testing, they meet the registration eligibility criteria in Section 5.1 and are assessed as potentially eligible for the lapatinib comparison using the eligibility criteria provided in Sections 6.1 and 6.2 and guidance on excluded concomitant medications in section 6.3.

Eligible patients who consent to HER-2 testing will be registered with the MRC CTU and have their tumour biopsy sample tested for HER-2 status. Patients with **HER-2 positive tumours** will be eligible for the lapatinib comparison provided they meet the full eligibility criteria in Sections 6.1 and 6.2 and are not taking any of the excluded concomitant medications listed in section 6.3.

Irrespective of HER-2 status, patients (including those patients who did not have their tumour tested for HER-2 status) may be eligible for registration in to the MRI and/or PET/CT sub-studies provided they meet the eligibility criteria in Sections 10.1 and 10.2. See Sections 10.3 and 11 for detail on pre-registration investigations and registering a patient into the MRI and/or PET/CT sub-studies only.

1.2.2 ST03 MRI Sub-study

Patients with lower oesophageal, Siewart Type I and II OGJ adenocarcinoma, either randomised to the lapatinib comparison or planned to receive sECX chemotherapy as defined in Sections 8.1-8.3 are eligible for registration into the ST03 MRI sub-study provided that they meet all eligibility criteria in Sections 10.1 and 10.2 and they do not have a contraindication to MRI.

1.2.3 ST03 PET/CT Sub-study

Patients with lower oesophageal, gastric, Siewert Type I, II and III OGJ adenocarcinoma, either randomised to the lapatinib comparison or planned to receive sECX chemotherapy as defined in Sections 8.1-8.3 are eligible for registration into the ST03 PET/CT sub-study provided that they meet all eligibility criteria in Sections 10.1 and 10.2.

1.3 Protocol Therapy

1.3.1 Patients with HER-2 Positive Tumours Randomised to the Lapatinib Comparison (Arms C and D)

Control Arm C: sECX

Patients randomised to control arm C will receive sECX consisting of 3 cycles of Epirubicin 50 mg/m^2 IV day 1, Cisplatin 60 mg/m^2 IV day 1 and Capecitabine 1250 mg/m^2 po daily in 2 divided doses Day 1-21 pre-operatively, surgery as detailed in Section 16 followed by 3 post-operative cycles of sECX at the same doses.

Investigational Arm D: mECX+L (dose levels will be confirmed at randomisation)
Patients randomised to the investigational arm D (mECX - dose level 0 initially + lapatinib) will receive mECX treatment with epirubicin and cisplatin as specified above for arm C but with a lower starting dose of capecitabine (1000mg/m^2/day), see Section 8.4 for further details of the dose levels within the mECX+L arm. In addition, on day 1-21 of every cycle they will receive lapatinib dose level 0, initially **1250mg/day**. **Doses of capecitabine and lapatinib may be modified during the feasibility study. At randomisation dose levels of both capecitabine and lapatinib will be confirmed**. Following completion of post-operative mECX+L patients will receive 18 weeks treatment with maintenance lapatinib at **1500mg/day unless modifications have taken place (please refer to Section 8.4 for instruction).**

At randomisation the current trial dose levels of capecitabine and lapatinib will be provided. If subsequent dose changes are recommended sites will be informed immediately.

1.3.2 Patients registered in MRI and/or PET/CT sub-studies ONLY
Patients who are registered in the MRI and/or PET/CT sub-studies only must be planned to receive sECX chemotherapy as defined in Sections 8.1-8.3.

1.4 Duration of Treatment

1.4.1 Patients with HER-2 Positive Tumours Randomised to the Lapatinib Comparison (Arms C and D)

Control Arm C: sECX
Pre-operative chemotherapy is expected to take 9 weeks and surgery should take place 5-6 weeks after this. Post-operative chemotherapy should recommence 6-10 weeks after surgery and should last for another 9 weeks. Therefore the duration of treatment in the control arm is expected to be 30-34 weeks.

Investigational Arm D: mECX+L
Treatment intervals will be the same as for the control arm C. Post-operative chemotherapy should recommence 6-10 weeks after surgery and should last for another 9 weeks. Patients in this arm will also receive 18 weeks of maintenance lapatinib, so that the total duration of therapy on the investigational arm will be 52 weeks.

1.4.2 Patients registered in MRI and/or PET/CT sub-studies ONLY
Patients who are registered in the MRI and/or PET/CT sub-studies **only** must be planned to receive sECX chemotherapy as defined in Sections 8.1-8.3. Therefore the duration of treatment for patients participating in the MRI and PET/CT sub-studies **only** is expected to be 30-34 weeks.

1.5 Outcome measures

1.5.1 ST03 Lapatinib Feasibility Study
The feasibility study is designed to:
a) assess the safety of adding lapatinib to ECX chemotherapy in patients with HER-2 positive operable oesophagogastric adenocarcinoma and to recommend a dose which

could be used in a future phase III trial, b) assess the feasibility of timely centralised HER-2 testing and c) estimate the level of HER-2 positivity in this study population.

1.5.2 ST03 MRI Sub-study

The MRI sub-study is designed to assess the sensitivity of MRI i.e. the proportion of patients in whom a complete resection is **not** possible who are correctly identified by the pre-operative MRI.

1.5.3 ST03 PET/CT Sub-study

The PET/CT sub-study is designed to a) assess Progression Free Survival (PFS), Overall Survival (OS), pathological response and clinical response in metabolic responders (>20% reduction in metabolic uptake) Vs non-responders (<20% reduction in metabolic uptake) and b) determine whether the response can be quantitatively assessed with PET/CT.

1.6 Assessments

1.6.1 Patients with HER-2 Positive Tumours Randomised to the Lapatinib Comparison (Arms C and D)

Patients with HER-2 positive tumours randomised to arms C and D will be assessed 3 weekly while they are receiving pre- and post-operative chemotherapy. Patients will then commence 9 weekly follow-up assessments at 9, 18 and 27 weeks from the start of cycle 4. Patients will receive follow up 1 year from surgery, 6 monthly for years 2 and 3 and then annually until death. Patients will also be asked to complete questionnaires aimed at assessing the effects of the investigational treatments on their quality of life (QoL) and on their use of health care resources (Health Economics (HE) study).

1.6.2 Patients Registered in to the MRI and/or PET/CT Sub-studies ONLY

It is recommended that patients are assessed 3 weekly while they are receiving pre- and post-operative chemotherapy. Patients will then commence 9 weekly follow-up assessments at 9, 18 and 27 weeks from the start of cycle 4. Patients will receive follow up 1 year from surgery, 6 monthly for years 2 and 3 and then annually until death.

1.7 Sample Size

1.7.1 ST03 Lapatinib Feasibility Study

For the lapatinib feasibility study, it is estimated that 200-400 patients will be screened for HER-2 status to result in 40 patients with HER-2 positive tumours (assuming a 10-20% positivity rate). It is expected that with a HER-2 positive rate of 20% it will take 18 months to complete randomisation to this comparison.

1.7.2 ST03 MRI Sub-study

For the MRI sub-study approximately 150 – 200 patients will be required to be registered.

1.7.3 ST03 PET/CT Sub-study

For the PET/CT sub-study approximately 130 patients will be required to be registered.

1.8 Data Maturity

1.8.1 ST03 Lapatinib Feasibility Study

A provisional decision about the recommended mECX+L doses for a future phase III trial will be made when the 20th lapatinib patient has completed their pre-operative

treatment. The primary analysis of the lapatinib feasibility study will take place once 20 patients have completed treatment with lapatinib.

1.8.2 ST03 MRI Sub-study

The primary analysis will take place when surgery and pathological assessments have been completed on all registered patients.

1.8.3 ST03 PET/CT Sub-study

The primary analysis will take place when 87 progression-free survival events have been reported and all patients have completed at least 12 months follow up from registration.

2. Background and Rationale

2.1 Introduction

Cancer of the stomach is the 6th most common cancer in the UK, causing over 5000 deaths each year. Lower oesophageal and gastroesophageal junctional adenocarcinoma are increasing in frequency, and rates for oesophageal adenocarcinoma for white men in the UK are amongst the highest in the world. All treatments aimed at cure involve surgery, but when it is used alone only around 20% of patients are alive after 5 years. The MRC ST02 trial (also known as MAGIC) (further details in Section 2.2) has shown that PFS and OS are significantly improved by the administration of peri-operative chemotherapy with ECF (epirubicin, cisplatin and 5-fluorouracil (5-FU)). Even so, 5-year survival rates of less than 40% are the best that can be expected, and thus there is an urgent need to build on this advance and develop new treatment approaches that can improve current survival rates.

2.2 Final Results of the MAGIC Trial

The MRC ST02 (MAGIC) (1) trial was a multi-centre, randomised controlled trial designed to assess the effect of peri-operative chemotherapy on patients with resectable adenocarcinoma of the stomach, oesophagogastric junction and lower oesophagus. Patients eligible for potentially curative resection were randomised to receive either peri-operative chemotherapy and surgery (CSC arm) or surgery alone (S arm). In the CSC arm, chemotherapy consisted of 3 pre-operative and 3 post-operative cycles of ECF (epirubicin 50mg/m^2 IV, cisplatin 60mg/m^2 IV and 5-FU 200mg/m^2/day continuous infusion) administered 3 weekly.

Between 1994 and 2002, 503 patients were randomised (250 CSC, 253 S), 74% had gastric carcinomas. Post-operative complications rates were similar between the 2 groups (CSC 46% S 45%), as were deaths within 30 days of surgery. The maximum diameter of the resected tumour was smaller in the CSC arm: median diameter (interquartile range) was 3cm (2-6cm) for CSC patients and 5cm (3-8cm) for S patients ($p<0.001$). Resection was considered curative in 79% of the CSC patients compared with 70% of the S patients who underwent surgery.

The final analysis of survival was planned for when approximately 320 deaths had occurred. This target was met in December 2004, with a median follow-up exceeding 3 years. The survival rates were significantly different (a 5 year rate of 36% for the CSC arm versus 23% in the S arm) with a hazard ratio of 0.75 (95% CI: 0.60, 0.93), $p=0.009$. The progression-free survival was also significantly better in the CSC arm (HR 0.66 [95%CI: 0.53, 0.81] $p=0.0001$), these results are now published (1). Similar results have also been reported from the FNLCC ACCORD07-FFCD 9703 trial (2).

2.3 Evidence for the Use of Capecitabine

Since the inception of the MAGIC trial, capecitabine, an oral tumour-selective fluoropyrimidine has become available. It can be used to provide prolonged 5-FU exposure at lower peak concentrations than those observed with bolus intravenous 5-FU schedules, and thus simulates continuous infusion of 5-FU (3). The advantages of capecitabine compared to infusional 5-FU are that it avoids the use of cumbersome delivery systems which compromise patient's independence and avoids the complications associated with long-term intravenous catheters. Reducing toxicity may have additional benefits in this group of patients as only 42% of the patients in MAGIC randomised to receive peri-operative chemotherapy completed all 6 cycles. Strategies that increase the number of cycles patients can tolerate may result in further survival

benefits. Reducing toxicity also results in fewer in-patient stays/out patient visits which may translate into a cost-saving measure for the health service (4).

Randomised evidence to justify the use of capecitabine instead of infused 5FU includes a trial in metastatic colorectal cancer (mCRC) where capecitabine produced a similar survival result but less toxicity than IV 5-FU/leucovorin (5). More immediately relevant to this trial are the results of the REAL2 trial. This is a multicentre randomised trial in advanced oesophagogastric tumours where the substitution of capecitabine for 5-FU was assessed. With nearly a thousand patients included in the comparison between fluoropyrimidines, the non-inferiority of capecitabine to infused 5-FU in this triplet regimen was confirmed. There was also no significant difference in response rates between arms. These results confirm that it is appropriate to use ECX in this study instead of the ECF regimen (6). Furthermore, the bioavailability of capecitabine has been shown in another study not to be impaired in patients with upper gastrointestinal pathology and/or gastrectomy (7). Therefore ECX has been chosen as the control arm regimen for this study.

2.4 Evidence for the Use of Bevacizumab

Recruitment to the bevacizumab comparison is now complete, for further details on the original ST03 trial design, background information and for the treatment of patients in the bevacizumab comparison please refer to Appendix T. Information on surgery, safety, pathology and follow up can be found in section 16 onwards.

2.5 Evidence for the use of lapatinib

Lapatinib is an oral small molecule tyrosine kinase inhibitor targeting human epidermal growth factor receptor-1 (HER-1, most commonly called EGFR) and the human epidermal growth factor receptor-2 (HER-2, also known as c-erb B2/neu and ERBB2).

2.5.1 EGFR

EGFR over-expression is a marker of poor prognosis in gastric and oesophageal cancers.(8-10) Cetuximab and panitumumab, monoclonal antibodies targeting EGFR have efficacy in combination with chemotherapy in the first (11,12) and second line treatment (13,14) of mCRC and additionally as monotherapy in chemotherapy-refractory mCRC.(15-17) The benefit in mCRC is limited to patients without mutations in *KRAS*, *BRAF*, *NRAS* and *PIK3CA* exon 20.(18) Cetuximab additionally reverses irinotecan resistance in mCRC (19) and improves survival when added to radiotherapy for head and neck cancer. (20) The small molecule tyrosine kinase inhibitors of EGFR, erlotinib and gefitinib, are active in patients with non-small cell lung cancer, but the activity is limited to patients with activating mutations of *EGFR*.(21,22) Erlotinib additionally confers a modest survival benefit when added to gemcitabine chemotherapy for unselected patients with metastatic pancreatic cancer. (23)

Research into the role of EGFR in oesophagogastric cancer and the potential value of EGFR inhibition has recently escalated, with several large studies of EGFR inhibitors now completed. Phase II studies of cetuximab in advanced gastric cancer have reported promising results in patients not selected by mutation status. (24-26). Results of a phase III randomised study of cisplatin and capecitabine in advanced gastric cancer (EXPAND), are awaited. A second anti-EGFR monoclonal antibody, panitumumab, was evaluated in combination with epirubicin, oxaliplatin and capecitabine (EOX) chemotherapy in advanced oesophago-gastric cancer (REAL3) (Waddell, ASCO 2012). This study was stopped following an IDMC analysis which demonstrated an inferior outcome in the experimental arm. However, it should be noted that the dose of conventional chemotherapy in the EOX-P arm was lower than

that in the EOX arm as modifications to this had been made based on excess toxicity seen in the combination arm during a safety run in phase. A study of gefitinib monotherapy in patients with advanced oesophageal cancer who have relapsed following first line therapy has completed recruitment, based upon the modest efficacy reported in three small phase II studies. (27-29)

2.5.2 HER-2

Amplification of the gene encoding HER-2 (also known as c-*erb B2/neu*) was first described in a gastric cancer cell line (30) and in resected human gastric cancers (31) in 1986. Administration of the anti-HER-2 monoclonal antibody, trastuzumab, to HER-2 positive gastric cancer cell lines *in vitro* and *in vivo* suppresses proliferation of the cell line and prolongs survival in the animal models. (32,33) Synergy with capecitabine and cisplatin has been reported in pre-clinical studies. (34) Activity of lapatinib has also been reported in gastric cancer cell lines, where *HER-2* amplification was the most predictive factor for lapatinib sensitivity, although none of the cell lines tested exhibited *EGFR* gene amplification. (35) Synergy was observed when lapatinib was administered after cisplatin dosing. (35)
Unlike breast cancer, where a clear negative prognostic effect from HER-2 over-expression or gene amplification was demonstrated, the effect on gastric cancer prognosis remains unclear, (36) with conflicting results reported. (33, 37-46)
Trastuzumab improves response rate, time to disease progression and overall survival (HR 0.80, 95%CI 0.64–1.00, p=0.046) when added to chemotherapy for advanced breast cancer (47) and also improves survival in early disease, when delivered after adjuvant chemotherapy (HR 0·66, 95%CI 0·47–0·91; p=0·0115). (48) Similarly, in advanced gastric and oesophagogastric junction cancer, trastuzumab improves response rate, PFS and overall survival (HR 0·74; 95% CI 0·60–0·91; p=0·0046) when added to a cisplatin/capecitabine doublet for patients with HER-2 over-expressing or *HER-2* amplified disease. (49) The greatest benefit was seen in the exploratory subgroup of patients with the strongest HER-2 positivity (HR 0.65, 95% CI 0.51-0.83), defined as 3+ on immunohistochemistry (IHC) or 2+ by IHC and positive fluorescent in situ hybridisation (FISH), (49) in whom the European Medicines Agency (EMEA) has granted a licence. Trastuzumab has been recommended by NICE in patients with HER-2 IHC 3+ oesophagogastric cancer only on the basis of greatest cost efficacy in that subgroup. Trastuzumab has not yet been evaluated in localised gastric cancer, in the adjuvant or peri-operative setting.

In advanced breast cancer, lapatinib has been evaluated in combination with capecitabine in patients with HER-2 positive disease that have previously progressed on trastuzumab, and significantly prolongs median time to progression, (50) with a trend towards improved overall survival (HR: 0.78, 95% CI: 0.55-1.12, p= 0.177), reported in the updated results.(51) The addition of lapatinib to trastuzumab in a similar patient population is also beneficial, with a greater benefit from the combination than lapatinib alone. (52) In the first line treatment of hormone receptor and HER-2 positive metastatic breast cancer, the addition of lapatinib to letrozole significantly prolongs PFS. (53) Selection of patients with HER-2 positive cancers is essential, as a study of paclitaxel plus lapatinib or placebo reported no benefit from lapatinib in an unselected population of patients with metastatic breast cancer, but improved response rate and time to progression in the subgroup of HER-2 positive patients. (54)

There are currently no data supporting a role for lapatinib in the adjuvant setting, however, evidence has accumulated for the use of this agent in the neoadjuvant setting. A phase II study in the neoadjuvant treatment for inflammatory breast cancer reported a 78% pathological response rate in combination with paclitaxel in HER-2 positive patients. Interestingly, there was no benefit reported in the cohort of patients that were EGFR positive (measured by IHC) but HER-2 negative. (55) A second neo-adjuvant study safely combined lapatinib with epirubicin, cyclophosphamide and docetaxel (EC-D) chemotherapy (four 3-week cycles of epirubicin 90 mg/m^2,

cyclophosphamide 600 mg/m^2 then four 3-week cycles docetaxel 100 mg/m^2) in 29 patients with HER-2 positive primary breast cancer. (56) The largest and most successful neoadjuvant study published to date is the NeoALLTO study, which examined the use of lapatinib both alone and in conjunction with trastuzumab and paclitaxel for HER2 overexpressing early breast cancer. Lapatinib based therapy demonstrated a pathological complete response rate comparable to that of trastuzumab, however the highest rates were seen with dual HER2 inhibition therapy. Grade 3 diarrhoea and grade 3 liver dysfunction were both more common in the lapatinib containing arms. However, this toxicity could be attributed to the fact that therapy with lapatinib and paclitaxel causes a 20% increase in systemic exposure to both drugs. After enrolment had commenced on this study the results of a pilot study showing excess diarrhoea with all three drugs at a dose of 1000 mg were reported and a protocol amendment with a dose reduction to 750 mg of lapatinib in the combination group was made. This and the inclusion of a diarrhoea management algorithm for all patients decreased the incidence of this toxicity. (57)

In advanced gastric cancer, a small phase II study of first line lapatinib monotherapy (1500mg/day) reported little efficacy in an unselected population. (58) However in a small second line study in HER-2 positive patients, although no objective responses were reported, two patients had prolonged disease stabilisation of 5 months or more. (59) More encouraging results have been seen with lapatinib in conjunction with capecitabine in the first line HER-2 positive metastatic gastric cancer setting; in a preliminary report of 67 patients treated with this combination response and stable disease rates were 22% and 45% respectively. (60) A phase III study of lapatinib (1250mg/day) in combination with capecitabine and oxaliplatin (LOGiC) is in progress, with no unexpected toxicities reported in the first 21 assessable patients. (61) Similarly, the combination with weekly paclitaxel as second line therapy appears to be very well tolerated, (62) with efficacy results awaited.

2.6 Evidence for the use of lapatinib maintenance therapy

Trastuzumab monotherapy, delivered for one year after adjuvant chemotherapy, improves survival in resected early breast cancer (HR 0·66; 95% CI 0·47–0·91, p=0·0115). (48) A further study reported a comparable magnitude of benefit (HR 0.42; 95%CI, 0.21 to 0.83; p=0.01) from nine weeks of adjuvant trastuzumab in the sub-group of 232 women with HER-2 positive disease randomised to trastuzumab or not. (63) The optimal duration of adjuvant trastuzumab is currently under evaluation in large phase III studies. Lapatinib monotherapy was compared to trastuzumab monotherapy and in combination with trastuzumab in the ALLTO trial. In September 2011 the IDMC for this study closed the lapatinib alone maintenance arm as lapatinib alone arm was unlikely to meet the prespecified criteria to demonstrate noninferiority to trastuzumab alone with respect to disease-free survival. These data have not yet been published. This study however, did not evaluate the efficacy of lapatinib initially in combination with chemotherapy as is being undertaken in ST03. Furthermore, oesophagogastric cancer differs substantially from breast cancer in that the EGFR pathway which is also inhibited by lapatinib is activated in a substantial proportion of tumours. The duration of 18 weeks monotherapy was chosen for this study to complete approximately one year of treatment from the commencement of neo-adjuvant therapy, with approximately 6 months of continuous lapatinib delivered adjuvantly

2.7 Evidence for use of MRI as a predictive biomarker for resectability

In addition to the pathological TNM stage, it is now recognised that the completeness of the surgical resection is an important factor in post operative survival, with those patients who undergo resection leaving either microscopic (R1 resection) or macroscopic (R2 resection) residual disease having a significantly poorer prognosis than those patients with a negative resection margin (R0 resection) (64, 65). The long

term results from the Kelsen study showed 32% of R0 patients were alive and disease free at 5 years compared with only 5% for patients with an R1 resection (65). Allum et al confirmed this; demonstrating a 42% 3 year survival for patients with an R0 resection compared with 18% & 8.6% for an R1 and R2 resection respectively (64). Frozen sections performed at the time of surgery reduce the risk of tumour at the longitudinal resection margins. It is the radial or CRM that remains at risk and currently the rate of R1/R2 resection due to a positive CRM is high at 39% (65, 66). One of the contributing factors to the high rate of R1/R2 resections is considered to be poor patient selection, with current staging techniques (–CT andEUS) unable to accurately identify patients who are at risk of having residual tumour at the CRM. The lack of soft tissue contrast afforded with CT and EUS prevents differentiation of post neoadjuvant therapy fibrosis from residual tumour. As a consequence the accuracy for staging and assessment of the CRM falls after neoadjuvant therapy. Since it has been shown that neoadjuvant therapy confers a survival advantage (1), the majority of patients in the UK considered for surgical resection are given pre-operative chemotherapy. Therefore there is a need to develop a more accurate non-invasive predictive biomarker for identifying tumour at the CRM in patients with oesophagogastric cancer. A previous strategic workshop on oesophageal cancer held by CRUK identified a need to incorporate novel imaging into clinical trials as a method of validating such techniques and to standardise their use prior to the roll out of the techniques into general clinical practice (67).

2.8 Evidence for use of PET/CT as a surrogate biomarker for predication of treatment response

The long term prognosis for patients with OGJ cancer remains poor, with surgical resection the only available curative therapy. Neoadjuvant chemotherapy aimed at suppressing locoregional infiltration by the tumour has been shown to significantly improve long-term survival in resectable adenocarcinoma and now forms part of routine therapy for locally advanced disease (68). Patients with a complete pathological response after neoadjuvant therapy have a consistent and impressive survival benefit, with 5-year survival rates of 60 to 100% (69). Unfortunately, neoadjuvant therapy is ineffective in up to 50-60% of patients (70). These patients are therefore subject to therapy related morbidity including nausea and vomiting, alopecia, increased susceptibility to infections through bone marrow suppression, and delay in performing surgery, without receiving any therapeutic benefit. A means of identifying this patient subgroup as early as possible during therapy is urgently required to enable avoidance of unnecessary and potentially toxic chemotherapy and offer the opportunity to switch as early as possible to different therapeutic modalities to improve survival.

The answer to this critical clinical question has proved elusive, as monitoring therapeutic response has, to date, been based on anatomical imaging modalities, principally CT and EUS, which rely on detection of structural changes such as tumour volume shrinkage (71). These morphological changes may not become evident for several weeks post therapy due to the time lag between cell death and resorption and interpretation is further complicated by the difficulty of distinguishing residual viable tumour from necrotic tissue or fibrosis by conventional imaging methods. Overall, EUS and CT have poor sensitivity for the assessment of response post therapy, with rates of 50 to 100% and 33 to 55% respectively being quoted in a recent meta-analysis (72). These figures refer to end of therapy assessment, with earlier detection of response not being feasible with these techniques.

^{18}F-FDG PET/CT is a functional imaging modality which can detect metabolic changes in tumours responding to therapy before corresponding structural changes are visible with conventional imaging techniques such as CT or EUS. This assessment of early response to therapy has within the past few years been successfully trialled in lymphoma and breast carcinoma (73, 74). In these settings FDG PET/CT can identify

subgroups showing a clear lack of response, who will benefit from immediate changeover to a new therapeutic regimen or in contrast, patients with highly chemosensitive tumours in whom treatment morbidity may be reduced by safely discontinuing therapy once complete remission is accurately confirmed. This can enable more individually tailored, response-adapted, therapy to be offered to patients. Rapid assessment of treatment effect and rapid cessation of ineffective therapy, is already creating new paradigms of individualised oncological therapy and the use of so-called response-adaptive treatment approaches is rapidly entering routine clinical practice (75). A further important benefit is that accurate and early assessment of tumour response can greatly enhance and accelerate the research evaluation of new therapies.

A number of studies have investigated the potential role of ^{18}F-FDG PET/CT as a surrogate marker predictive of treatment response and prognosis in oesophageal carcinoma (76). The MUNICON study of 119 patients with adenocarcinoma of the distal oesophagus and gastric cardia, although limited by a non-randomised trial design, has already confirmed prospectively the usefulness of early metabolic response evaluation, and demonstrated the feasibility of a PET-guided treatment algorithm (77). Most further studies to date, although tending in the main to support the value of PET in this role, have suffered from significant methodological weaknesses. A recent meta-analysis of these studies concluded that drawing large-scale inferences based on their data has been hindered by small study numbers, differing neoadjuvant therapies and the lack of defined treatment response metrics (76). A particular issue is that the vast majority have examined heterogenous patient cohorts of Squamous Cell Carcinoma (SCC) and adenocarcinoma, with only 3 of a total of 22 focusing exclusively on adenocarcinoma. These diseases exhibit fundamentally different pathobiology, with response rates to neoadjuvant chemotherapy being far lower in SCC than adenocarcinoma. The ongoing Dutch multicentre randomized NEOPEC-trial of FDG-PET for response-monitoring in oesophageal cancer similarly does not make this important distinction (78). A large prospective multicentre trial involving a homogeneous group of patients is therefore needed as a matter of urgency to validate the potential role of PET to influence clinical management in this setting. The ST03 PET/CT Sub-study will therefore be the first large scale study of a homogenous group of patients and will use standardised patient preparation, uniform PET timing in relation to neoadjuvant therapy, data acquisition and processing, and data interpretation. The EORTC 1999 definition of ^{18}F-FDG tumour response (79) for metabolic response criteria will be used in this sub-study.

The rationale for the ST03 PET/CT sub-study is that this study aims to confirm the prognostic value of FDG-PET/CT after 1 cycle of neoadjuvant chemotherapy patients with oesophagogastric adenocarcinomas where patients are treated uniformly according to the same treatment protocol, which depends on CT & EUS for response evaluation. Clinicians will be blinded to the result of PET/CT after 1 cycle and treatment decisions will be based on the agreed conventional response criteria at the end of pre-operative chemotherapy regardless of the PET/CT (after 1 cycle) scan outcome. In this way the metabolic response rate can be related to the outcome measures and the integrity of the chemotherapy trial is maintained.

The results will provide the strongest evidence to date on the prognostic value of PET/CT after 1 cycle of neoadjuvant therapy. If on this evidence an FDG-PET scan proves an accurate predictor of lack of treatment response, it can be used in the future in order to identify non-responding patients early in their course of treatment, who will benefit from immediate changeover to a second-line therapeutic regimen.

This sub-study will also offer a unique opportunity to evaluate a quantitative response system using percentage reduction in Standardised Uptake Value (SUV).

3. Trial Design and Objectives

3.1 Trial Design

The original ST03 trial was a randomised controlled, multi-centre, open-label phase II/III trial designed to evaluate the safety and efficacy sECX chemotherapy with or without bevacizumab in patients with operable oesophagogastric adenocarcinomas.

Recruitment to the bevacizumab comparison is now complete, for further details on the original ST03 trial design, background information and for the treatment of patients in the bevacizumab comparison please refer to Appendix T. Information on surgery, safety, pathology and follow up can be found in section 16 onwards.

In addition to the bevacizumab comparison, the ST03 trial encompasses 3 sub-studies which are still ongoing. These sub-studies are summarised below and detailed within the main body of the protocol (Sections 1 onwards).

3.1.1 ST03 Lapatinib Feasibility Study

The lapatinib feasibility study incorporates HER-2 testing and the subsequent randomisation of patients with HER-2 positive tumours into the lapatinib comparison (sECX vs mECX+L). Approximately 40 patients with HER-2 positive tumours will be recruited over 18 months.

Diagnostic biopsies from patients who are eligible for registration will be centrally tested for HER-2 status. Patients with HER-2 positive tumours, defined by 3+ IHC or 2+ IHC with confirmation of *HER-2* amplification by Dual colour Dual hapten In Situ Hybridisation (DDISH), will be randomised to receive either 6 cycles of peri-operative sECX chemotherapy or 6 cycles of peri-operative mECX chemotherapy and lapatinib, plus an additional 18 weeks of lapatinib as maintenance therapy post surgery, in a 1:1 ratio.

The feasibility study was initially limited to 16 selected UK centres but further centres have now been invited to participate.

3.1.2 ST03 MRI Sub-study

In participating centres, patients with lower oesophageal and type I and II OGJ operable adenocarcinomas may register for the MRI observational sub-study evaluating pre-operative MRI as a predictor of R0 resection. Patients either randomised into the lapatinib comparison or that are planned to receive sECX as defined in Sections 8.1-8.3 of this protocol are eligible for the MRI sub-study provided they meet all the eligibility criteria within Sections 10.1 and 10.2.

3.1.3 ST03 PET/CT Sub-study

In participating centres, patients with gastric, lower oesophageal and OGJ operable adendocarcinomas may register for the PET/CT observational sub-study evaluating the role of FDG-PET/CT after 1 cycle of neoadjuvant chemotherapy, for the early prediction of non-response to neoadjuvant chemotherapy in patients with operable oesophagogastric adenocarcinoma. Patients either randomised into the lapatinib comparison or that are planned to receive sECX as defined in Sections 8.1-8.3 of this protocol are eligible for the PET/CT sub-study provided they meet all the eligibility criteria within Sections 10.1 and 10.2.

3.2 Trial Objectives

3.2.1 ST03 Lapatinib Feasibility study

The primary objective of the lapatinib feasibility study is to assess the safety of adding the small molecule tyrosine kinase inhibitor lapatinib, to ECX chemotherapy administered peri-operatively in patients with HER-2 positive operable oesophagogastric adenocarcinoma.

3.2.2 ST03 MRI Sub-study

The primary objective of the MRI sub-study is to assess whether the additional use of high resolution T2 weighted MRI as a predictive biomarker improves patient selection for surgery by more accurately identifying patients at risk of an R1/R2 resection than conventional staging alone.

3.2.3 ST03 PET/CT Sub-study

The primary objective of the PET/CT sub-study is to evaluate the role of ^{18}FDG-PET/CT after 1 cycle of neoadjuvant chemotherapy, for the early prediction of non-response (defined as >20% reduction in metabolic activity) to neoadjuvant chemotherapy in patients with operable oesophagogastric adenocarcinoma. This will be assessed by reviewing a) whether an early response on FDG-PET after 1 cycle of chemotherapy predict better prognosis with respect to PFS, OS, pathological response and clinical response, and b) whether response can be quantitatively assessed with FDG-PET.

3.3 Endpoints

3.3.1 ST03 Lapatinib Feasibility Study

Primary Endpoint

The primary outcome measure for the lapatinib feasibility study is the safety of the addition of lapatinib to mECX chemotherapy.

Formal safety reviews will occur after 10 and 20 patients have completed one cycle of mECX+lapatinib. Additionally, a further safety review will be conducted once 10 patients have completed 1 cycle of maintenance lapatinib. We intend to publish the results of the safety of the addition of lapatinib to mECX chemotherapy once the recommended dose is established.

Toxicity will continue to be monitored throughout post-op treatment and dose reductions will be made accordingly on an individual basis. **The primary endpoint will be the establishment of a recommended dose for a future phase III trial.**

Secondary Endpoints

1. The aim would be for >90% of samples to have a HER-2 result within 10 working days. This target is ambitious, and given that this is a neoadjuvant study with patients undergoing a series of investigations prior to commencing neoadjuvant chemotherapy it is unlikely to delay treatment if the HER-2 testing takes longer. Results available from the FOCUS-3 study suggest 15 working days may be a more realistic target. The key measure will be to assess feasibility and to ensure that HER-2 testing does not delay treatment.

2. To determine the proportion of samples that are HER-2 positive. Feasibility of a national HER-2 trial will depend on the HER-2 positivity rate in the study population.

3.3.2 ST03 MRI Sub-study

Primary Endpoint

The MRI sub-study is designed to assess whether the additional use of high resolution T2 weighted MRI as a predictive biomarker improves patient selection for surgery by more accurately identifying patients at risk of an R1/R2 resection. The primary outcome for this sub-study will be sensitivity of MRI, i.e the prorportion of patients in whom a complete resection is **not** possible are correctly identified by the pre-operative MRI.

Secondary Endpoint

The association between apparent diffusion co-efficient (ADC) values and pathological response (Mandard grade) will be assessed as a secondary endpoint.

3.3.3 ST03 PET/CT Sub-study

Primary Endpoint

The primary outcome will be PFS in metabolic responders (>20% reduction in metabolic activity) vs non-responders.

Secondary Endpoint

The association between metabolic response and the following will also be assessed as secondary outcome measures: pathological response, radiological response prior to surgery, R0 resection rate and OS.

4. Centre Enrolment and Accreditation Process

4.1 Selection of Trial Centres/Clinicians

To participate in the ST03 trial, Investigators and clinical trial centres must be registered with the ST03 Trial Manager at the MRC CTU, and must fulfil a set of basic criteria which have been agreed by the ST03 Trial Management Group (TMG).

ST03 Centre Selection Criteria are:

- The Principal Investigator (PI) has appropriate experience to conduct the trial according to the principles of Good Clinical Practice (GCP) and the protocol.
- The PI should have sufficient time to properly conduct and complete the trial within the agreed trial period.
- The clinical trial centre has trained oncologists to deliver chemotherapy and who are integrated into the gastro-oncology multi-disciplinary team and experienced in the care of patients receiving intravenous chemotherapy.
- The clinical trial centre has an adequate number of qualified staff and adequate facilities, for the foreseen duration of the trial, to conduct the trial properly and safely and in accordance to GCP and the protocol.
- The clinical trial centre has appropriate pathologists who are experienced in the reporting of upper gastrointestinal cancer and able to support the ST03 pathology review and the Trans-ST03 sub-study tissue sample collection.
- If participating in the MRI and/or PET/CT sub-studies, the clinical trial centre has experienced radiologists who are able to report tumour measurements according to the RECIST Version 1.0 criteria and support the sub-studies where applicable.

- The clinical trial centre has experienced surgeons who specialise in the management of upper gastrointestinal cancer or have appropriate referral patterns to a specialised oesophagogastric surgery centre.

- The clinical trial centre has sufficient data management resources to allow prompt data return to the MRC CTU. Centres who have previously participated in MRC trials must have a good track record of data return.

- The clinical trial centre should permit monitoring and auditing by the MRC as Sponsor, and inspection by the appropriate regulatory authorities.

Those centres that meet the ST03 criteria will be issued with the ST03 master file documentation to apply for their Site Specific Approval (SSA) and MRC CTU accreditation documents. Centres must complete the ST03 accreditation form **at the same time as** applying for their SSA.

4.2 Accreditation Process

Each selected clinical trial site must complete the ST03 accreditation form which includes the Investigator Statement, Signature and Delegation of Responsibilities Log, and Staff contact details. The Investigator Statement verifies that the site is willing, and able to comply with the requirements of the trial. This will be signed by the PI at the site. In addition, and in compliance with GCP, all site staff participating in the trial must complete the signature and delegation of responsibilities log and forward this to the MRC CTU. The MRC CTU must be notified of any changes to trial personnel and/or their responsibilities. An up-to-date copy of this log must be stored in the Trial Master File at the site and also at the MRC CTU.

Accreditation Checklist

Sites must complete the MRC accreditation process before a site can be activated for recruitment into the ST03 trial. The MRC CTU must receive:

- ✓ A completed Investigator Statement, signed by the PI
- ✓ Completed staff Signature and Delegation log of Responsibilities
- ✓ Full contact details for all site personnel
- ✓ Confirmation of local Research and Development (R&D) approval (includes SSA)
- ✓ Completed local trial master file self assessment form
- ✓ ST03 Non-commercial Research Agreement between the MRC and Hospital Trust
- ✓ Completed the Pharmacy Initiation Checklist

On receipt of the above documents at the MRC CTU, written confirmation will be sent to the PI and an ST03 Randomisation Pack (which includes ST03 CRFs) will be sent to the first point of contact to confirm that the centre has been approved to randomise patients into the ST03 trial.

4.3 MHRA CTA Approval for Investigators Participation in ST03

The Clinical Trial Authorisation (CTA) for the ST03 trial requires that the Medicines and Healthcare Products Regulatory Agency (MHRA) be supplied with the names and addresses of all participating investigators/centres. Trial staff at the MRC CTUwill perform this task; hence it is **vital to receive full contact details for all investigators prior to their entering patients.**

4.4 Centres Participating in the Lapatinib Feasibility Study

16 centres participating in the original component of the ST03 Trial were selected to run the lapatinib feasibility study initially. The centres were chosen based on high recruitment figures, good data return and timely sample submission for the central pathology review and Trans-ST03 sub-study. The number of centres participating has been reviewed and we are in the process of opening further centres to the the lapatinib feasibility study.

All centres participating in the lapatinic feasibility study will be contacted individually and details of the initial lapatinib drug order will be discussed with the pharmacist once local R&D approval has been obtained.

Additional Accreditation Checklist for the Lapatinib Feasibility Study

All sites selected and invited to participate in the ST03 lapatinib feasibility study have already been opened to the ST03 bevacizumab comparison and therefore have already fulfilled the requirements for accreditation set out in Section 4.2 above.

In order for the site to be accredited for the lapatinib feasibility study the site must complete and return the following documentation to the MRC CTU before the site can be activated for recruitment into the lapatinib feasibility study:

- ✓ Confirmation of local R&D approval for the lapatinib feasibility study
- ✓ ST03 Lapatinib Feasibility Study Variation to Non-commercial Research Agreement between the MRC and Hospital Trust
- ✓ Template pharmacy prescriptions for the trial arms returned and approved by the ST03 TMG
- ✓ Completed local and pharmacy master file self assessment form

On receipt of the above documents at the MRC CTU, written confirmation will be sent to the PI and first point of contact to confirm that the centre has been approved to register and randomise patients into the ST03 lapatinib feasibility study. The pharmacist will also be informed of the centre's activation and an initial lapatinib drug order will be dispatched to the ST03 pharmacist (see **Appendix J** for Drug Supply Information).

If the site wishes to conduct local HER-2 testing the requirements set out in section 5.6 must be met.

4.5 Centres participating in the MRI sub-study

A small number of selected centres already participating in the original component of the ST03 Trial were initially invited to participate in the MRI sub-study. The number of centres participating has been reviewed and we are in the process of opening further centres to the MRI sub-study.

Protocol development and training in the interpretation of oesophageal MRI will be carried out in all centres prior to the commencement of the study.

A detailed description of the requirements for surgeons with regard to specimen handling is given in Section 16.1.4 of the protocol. This will allow for orientation of the specimen by the pathologist and enable the MR images to be matched with the pathology. The requirements for pathologists are given in Section 32 of the protocol and more detail can be found in the guidance booklet for pathologists. These include the provision of high resolution macroscopic images of individual cross-sections taken

at time of cut up and submitted, together with the HE stained slides for the Central Pathology Review. If your centre is unable to fully comply with the pathology requirements as described above, you may still recruit patients to the MRI sub-study, please contact the ST03 trial manager for advice if this is the case.

The requirements will be discussed with the relevant clinicians at participating centres prior to commencement of the MRI study at each centre. Further to the practical requirements set out above the site will also be required to provide confirmation of local R&D approval for the MRI sub-study and the ST03 MRI Sub-study Variation to Non-commercial Research Agreement must be in place between the MRC and the Hospital Trust.

Once the site has completed the accreditation procedures for the MRI sub-study, written confirmation will be sent to the PI and first point of contact to confirm that the centre has been approved to register patients for the MRI sub-study.

4.6 Centres participating in the PET/CT sub-study

A small number of selected centres already participating in the original component of ST03 were initially invited to participate in the PET/CT sub-study. The number of centres pariticpating has been reviewed and we are in the process of opening further centres to the PET/CT sub-study.

Before registering patients to the PET/CT sub-study the following documentation must be in place:

- ✓ ARSAC licence to include PET/CT scan for the ST03 sub-study
- ✓ Local R&D approval for the PET/CT sub-study
- ✓ Accreditation from the NCRI Core Lab

Once these documents have been returned and the centre has received written approval from the MRC CTU they may register patients into the PET/CT sub-study.

Figure 5.1 Schema for HER-2 testing, Registration and Randomisation including Registration for MRI and PET Sub-studies

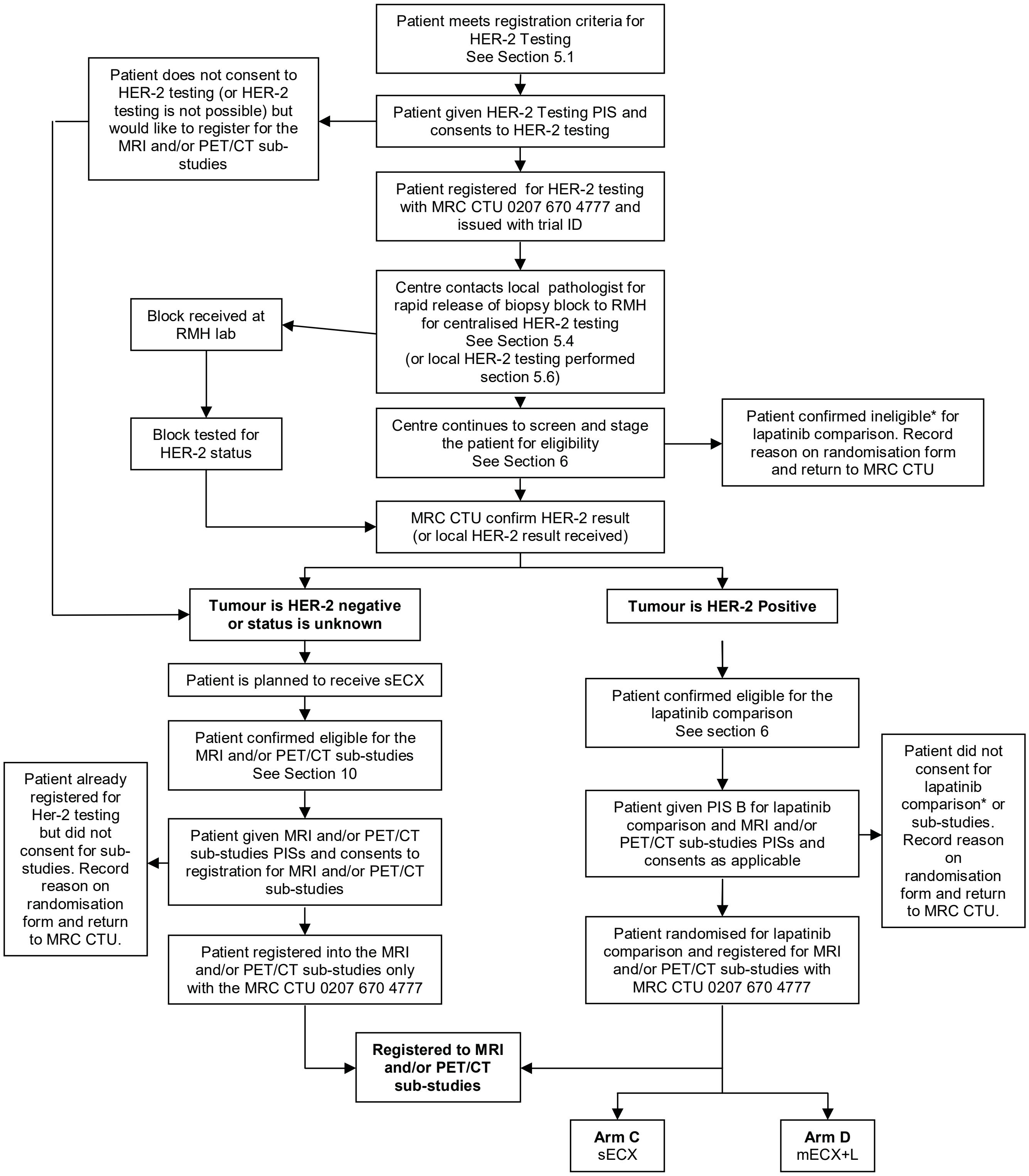

* If patient has HER-2 positive tumour but does not consent or is ineligible for lapatinib comparison the patient may register for the MRI and/or PET/CT only if eligible.

Figure 5.2 Trial Schema for Patients with HER-2 Positive Tumours Entering Lapatinib Comparison

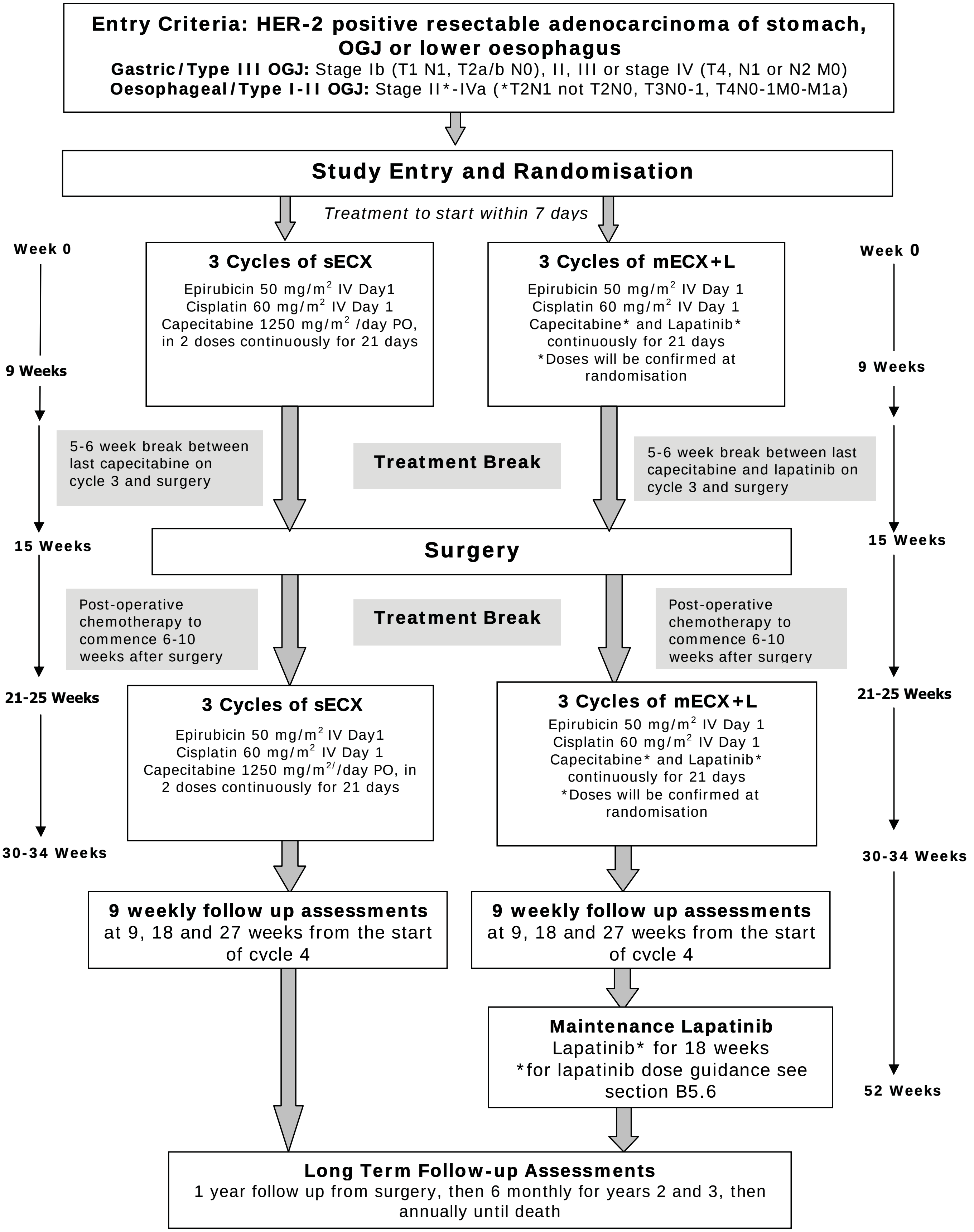

5. Registration and HER-2 Testing for the Lapatinib Feasibility Study

5.1 Registration Eligibility Requirements

In order for patients to be eligible for registration for HER-2 testing the patient must meet the below registration inclusion criteria <u>and</u> be potentially eligible for the lapatinib comparison as assessed by the investigator using the eligibility criteria provided in Sections 6.1 and 6.2 and guidance on excluded concomitant medications in Section 6.3.

Potential patients will need to have the HER-2 status of their biopsy samples determined in order to establish whether they are eligible for the lapatinib comparison.

Only patients with HER-2 positive tumours can be randomised into the lapatinib comparison provided they meet <u>**all**</u> eligibility criteria within Sections 6.1 and 6.2 and are not taking any of the excluded concomitant medications listed in Section 6.3. These patients may also enter the MRI and/or PET/CT sub-studies if recruited at a participating centre, provided all relevant eligibility criteria described in section 6.1 and 6.2 are met.

Patients who are found to have HER-2 negative or unknown/undefined tumours may be eligible for registration into the MRI and/or PET/CT sub-studies providing the centre is participating in these sub-studies and all eligbility criteria in Sections 10.1 and 10.2 are met.

Inclusion criteria for Registration for HER-2 Testing:

a) Patients must have a histologically verified lower oesophageal, Siewert Type I, II or III OGJ or gastric adenocarcinoma, and must not have received any previous treatment for their cancer. All patients should have had a CT of chest and abdomen (pelvis is optional) to confirm no metastasis and the surgeon believes that an R0 resection can be achieved.

b) Patient must have given consent to provide a tumour sample for HER-2 testing before registration for HER-2 testing.

c) Patient assessed as potentially eligible for the lapatinib comparison using the eligibility criteria provided in Sections 6.1 and 6.2 and guidance on excluded concomitant medications in Section 6.3.

A full evaluation of the eligibility criteria provided in Sections 6.1 and 6.2 with reference to guidance on excluded concomitant medications listed in Section 6.3 must be confirmed prior to randomisation in to the lapatinib comparison following HER-2 testing.

5.2 Consent for HER-2 Sample testing

Patients who meet the inclusion criteria for registration detailed above in Section 5.1 are eligible for registration. Once they have had adequate time to read the **Patient Information Sheet (PIS) for HER-2 Testing**, the patient will need to provide consent for the release of their tumour block on the **ST03 HER-2 Testing Consent Form**. See Figure 5.1 for the process for patients participating in the HER-2 testing.

If HER-2 testing is not possible patients may still be eligible for registration in to the MRI and/or PET/CT sub-studies providing they meet the eligibility criteria in Sections 10.1 and 10.2.

Please go to Section 10 for further details on the eligibility criteria and instructions for patients to be registered into the MRI and/or PET/CT sub-studies only.

5.3 Registration

It is requested that the following CRF is completed before calling the registration line:

- HER-2 Testing Registration Form (ST03/0-HER)

To register a patient telephone the MRC CTU:

+44 (0)207 670 4777 (9am - 5pm, Mon-Fri)

During registration the patient will be issued with a trial number.

This trial number will be used to identify the tumour blocks sent to the central testing laboratory at the Royal Marsden Hospital (RMH). The patient's date of birth should also be used to cross-reference the sample.

No treatment allocation will be performed at this point. However, the trial number issued at registration will continue to be the patient's unique identifier should they subsequently be randomised into the lapatinib comparison or registered for the MRI and/or PET/CT sub-studies.

If your centre has approval to conduct HER-2 testing locally (see Section 5.6 for criteria) you are still required to register the patient with the MRC CTU on the telephone number above as soon as they have consented to HER-2 testing.

5.4 Block Request and Dispatch Procedure

Once the patient has been registered with the MRC CTU the Research Nurse (RN) should immediately contact the local pathology department where the biopsy was performed, faxing a copy of the patient's consent form and a completed HER-2 Sample Request and Report Form (ST03/HER-2Test1).

The RN should also fax a copy of the completed HER-2 Sample Request and Report Form (ST03/HER-2Test1) to the MRC CTU to confirm that the sample has been requested from the local pathology laboratory. This confirmation should be received at the MRC CTU on the **same day** as registration.

The Sample Request and Report Form (ST03/HER-2Test1) requests that the pathologist identifies and releases a tumour bearing Formalin Fixed Paraffin Embedded (FFPE) block and pathology report to the central testing laboratory at RMH within 3

days by first class post. The pathologist must also confirm dispatch of the FFPE block by faxing a copy of the updated Sample Request and Reporting Form to the MRC CTU.

See Figure 5.3 for more information on the sample collection and testing procedure. For more details on the sample collection and testing process please see Appendix Q: ST03 HER-2 Testing and Sample Handling Procedure.

Figure 5.3: Overview of Randomisation and Pathology Block Request

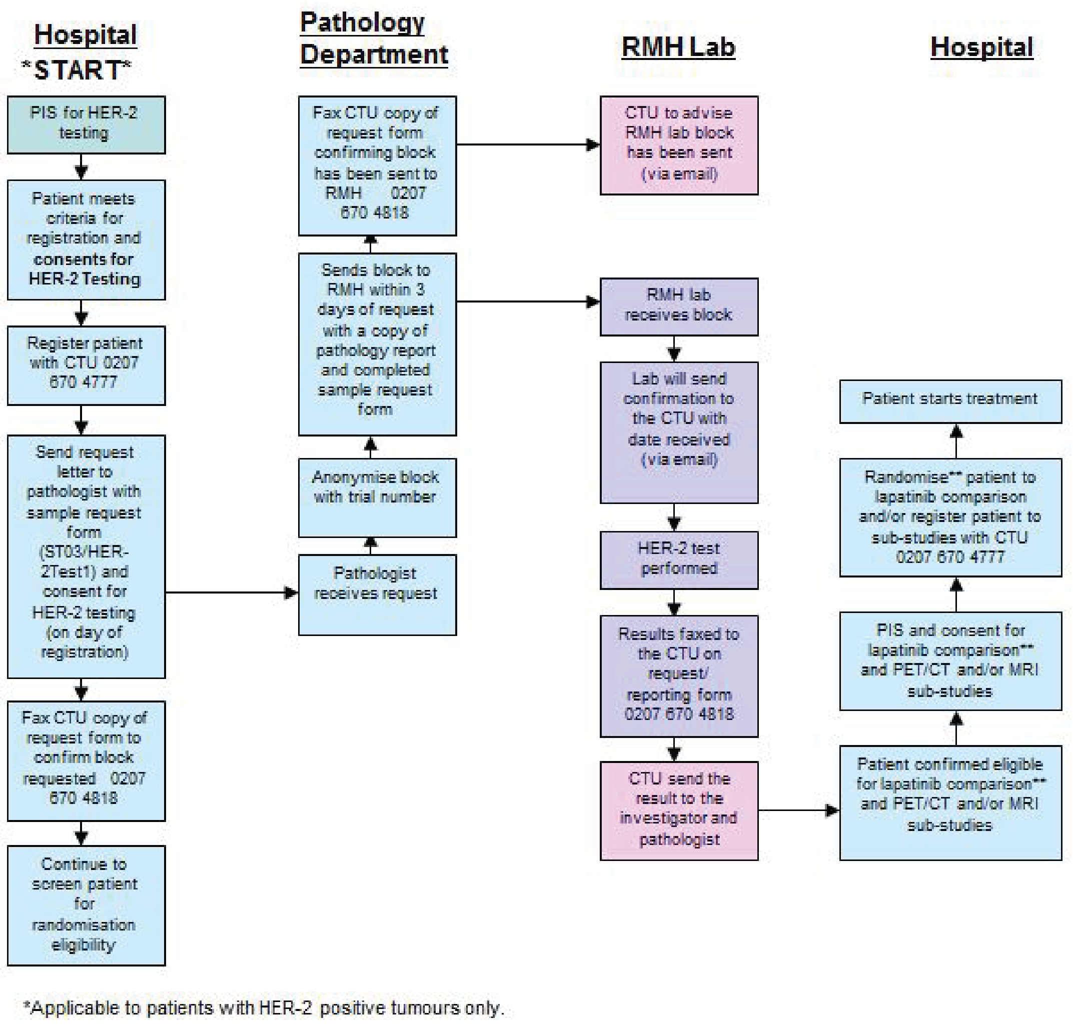

5.4.1 Tumour Block Tracking

Tumour blocks will be tracked from time of patient registration for HER-2 testing to time that the HER-2 result is available. The sample tracking process is shown in Figure 5.3 and is detailed below. Please see section 5.6 for details on local HER-2 testing.

- When a patient is registered for HER-2 testing staff at the MRC CTU will inform the RMH laboratory so that they are aware that a sample is expected.

- The RN will request the block from the local pathology department where the biopsy was performed by sending the completed pathology request cover letter

with the completed Sample Request and Report Form (ST03/HER-2Test1) and the patient's consent for HER-2 testing to the local pathology department. Once requested the RN will confirm with the MRC CTU that the request has been placed for the release of the block by faxing a copy of the completed Sample Request and Report Form (ST03/HER-2Test1) to the MRC CTU (Fax No: 0207 670 4818).

The blocks should be requested, and the MRC CTU notified of this, on the day of registration.

- The pathologist will confirm dispatch of the patient's block to the RMH laboratory with the MRC CTU by faxing an updated Sample Request and Report Form (ST03/HER-2Test1) to the MRC CTU (Fax No: 0207 670 4818).
  Please note: Samples should be anonymised, care should be taken to ensure samples are not damaged in the process of anonymisation.

  The blocks should be sent to the RMH laboratory within 3 days of receiving the request from the RN.

- Staff at the MRC CTU will confirm with the RMH laboratory that the sample has been released and should be received shortly.

- RMH laboratory staff will inform the MRC CTU of receipt of the block.

  - If the block has not arrived at the RMH laboratory within one week after registration, MRC CTU staff will contact the hospital pathology laboratory to reconfirm that the block was sent.

- Staff at the RMH laboratory will inform the MRC CTU as soon as the HER-2 result is available.

- A member of the ST03 team at MRC CTU will notify the PI, RN and local pathologist by fax of the patient's HER-2 status.

(Centres will be informed should there be any minor changes to these procedures.)

5.5 HER-2 Testing at the RMH laboratory (Central HER-2 Testing)

Central testing of HER-2 status is defined for the purpose of this study by either 3+ IHC or 2+ IHC with confirmation of HER-2 gene amplification by DDISH. HER-2 testing will be performed using the validated Ventana and 4B5 antibody system in an accredited laboratory which participates in UK NEQAS assessments. IHC will be scored according to the gastric cancer scoring devised for the ToGA study and confirmed by Ruschoff et al (80).

For ISH assessments, a HER-2:CEP17 ratio ≥2 is defined as amplification.

5.6 Local HER-2 Testing

Local HER-2 testing may be permitted following approval from the ST03 TMG. Local HER-2 testing approval for the ST03 lapatinib feasibility study is subject to the local laboratory meeting the following criteria:

a) Laboratory is CLIA/CPA accredited

b) Laboratory participated in the UK NEQAS gastric HER2 test pilot and received a satisfactory result

c) Local GI oncology pathologist

d) Prior written consent for local HER-2 testing received from MRC CTU

Please note that central verification by the RMH laboratory for all blocks will be required but that local HER-2 results will be used to inform randomisation.

Further details of the process for local HER-2 testing and central verification will be provided to centres following approval.

Patients who will have their tumour tested locally must be registered for HER-2 testing with the MRC CTU as instructed in Section 5.3 so that a patient ID can be allocated.

5.7 Patient Screening Whilst Awaiting HER-2 Test Result

Whilst awaiting the results of the HER-2 test, the patient can continue with the screening and staging investigations to confirm eligibility for randomisation into the lapatinib comparison and registration into the MRI and/or PET/CT sub-studies, if applicable.

In order to proceed to randomisation following a HER-2 positive result, patients must meet all eligbility criteria for randomisation into the lapatinib comparison and, if applicable, registration in to the MRI and/or PET-CT sub-studies as detailed in Sections 6.1 and 6.2 and 6.3.

In order to register the patient for the MRI and/or PET/CT sub-studies only following a HER-2 negative result patients must meet all the eligibility criteria as detailed in Sections 10.1 and 10.2. See Sections 10.3 and 11 for detail on pre-registration investigations and registering a patient into the MRI and/or PET/CT sub-studies only.

5.8 HER-2 Results

MRC CTU will inform the PI, RN and local pathologist of the HER-2 result for each patient's tumour by fax as soon as the result is received from the central laboratory at RMH.

If the HER-2 test is conducted locally the site should inform the MRC CTU result as soon as possible by sending the anonymised pathology report either by fax to 020 7670 4818 or by email to ST03@ctu.mrc.ac.uk. The result must be sent prior to randomisation as it is the local result that will be used for randomisation (although central verification is required for all blocks). Please refer the local HER-2 testing and central verification procedure for further information (sent to site once approval for local HER-2 testing has been obtained).

Patients must be informed of the HER-2 status of their tumour.

5.8.1 Patients with HER-2 Positive tumours

Patients with confirmed HER-2 positive tumours may be eligible for randomisation into the lapatinib comparison provided that all eligibility criteria listed within Sections 6.1 and 6.2 are met and the patient is not receiving any of the excluded concomitant medications listed within Section 6.3.

Patients with HER-2 positive tumours may also register for the MRI and/or PET-CT sub-studies provided that the patient all meets the eligibility criteria listed for the MRI and/or PET/CT sub-studies in Section 6.1 and 6.2.

If the patient does not meet all the eligibility criteria for the lapatinib comparison they may meet the eligibility criteria for registration in to the MRI and/or PET/CT sub-studies only listed within Sections 10.1 and 10.2 and if so may register for these sub-studies only. See Sections 10.3 and 11 for detail on pre-registration investigations and registering a patient into the MRI and/or PET/CT sub-studies only.

Please go to section 6 for eligibility criteria and instruction for patients to be randomised to the lapatinib comparison.

5.8.2 Patients with HER-2 Negative/Undefined tumours or Failed Molecular Tests

Patients with confirmed HER-2 negative tumours are not eligible for the lapatinib comparison but may be eligible for registration in to the MRI and/or PET/CT sub-studies only provided that all the eligibility criteria listed in Sections 10.1 and 10.2 are met.

This also applies if the RMH laboratory fails to determine the HER-2 status of the patient's tumour (HER-2 undefined) or a result is not obtained within suitable time limits (HER-2 unknown).

Please go to Section 10 and 11 for further details on eligibility criteria and procedures for registering a patient into the MRI and/or PET/CT sub-studies only.

5.8.3 Patients Ineligible or do not Consent for the Lapatinib Comparison

Some patients who are registered for HER-2 testing may subsequently be found to be ineligible for, or may decline, randomisation into the lapatinib comparison. The MRC CTU are interested in finding out the reasons why patients do not continue to randomisation after registration and therefore request that the relevant question on the Randomisation Form (ST03/1-HER) be completed and returned to the MRC CTU to detail these reasons.

6. Selection of Patients for the Lapatinib Comparison

6.1 Inclusion Criteria for Patients with HER-2 Positive Tumours Entering the Lapatinib comparison

In order to proceed to randomisation for the lapatinib comparison (and registration into the MRI and/or PET/CT sub-studies if applicable) following HER-2 testing; patients

must meet all the inclusion criteria listed below in addition to the eligibility criteria met for HER-2 testing registration:

a) Patients with histologically verified lower oesophageal, Siewert Type I, II or III OGJ or gastric adenocarcinoma, who have not received any treatment for their cancer.

Please note the eligible tumour types for the MRI and PET/CT Sub-studies listed below:

MRI Sub-study: Patients with histologically verified lower oesophageal, Siewert Type I or II OGJ adenocarcinoma, who have not received any treatment for their cancer.

PET/CT Sub-study: Patients with histologically verified lower oesophageal, Siewert Type I, II or III OGJ or gastric adenocarcinoma, who have not received any treatment for their cancer.

b) Confirmed HER-2 Positive Tumour either by RMH centralised testing lab confirmation via the ST03 Trial Team at the MRC CTU or by ST03 TMG approved local HER-2 testing performed at site.

The inclusion criteria described below are based on the TNM classification 6th edition for gastric cancer (gastric cancer and Siewert type III OGJ tumours), and oesophageal cancer (lower oesophageal cancer and Siewert type I/II OGJ tumours). Should you use TNM7 for staging of a patient's disease, please see Appendix M for guidance or speak to the ST03 team at the MRC CTU.

c) i) Siewert type III OGJ or Gastric Adenocarcinomas (using gastric cancer staging system)
Tumours should be Stage Ib (T1 N1, T2a/b N0), II, III or stage IV (T4 N1 or N2) with no evidence of distant metastases (M0) where the surgeon believes that an R0 resection can be achieved by excision of a contiguous structure.
Patients with linitis plastica should not be randomised.

ii) Lower Oesophageal or Siewert type I/II OGJ Adenocarcinomas (using oesophageal cancer staging system)
Tumours should be Stage II to Stage IVa (T1 N1, T2 N1, T3 N0-1, but not T2N0). T4 (N0 or N1) tumours are also eligible providing that they involve only the crura OR invade only the mediastinal pleura, where the surgeon believes that an R0 resection can be achieved by excision of a contiguous structure.
Patients with nodal disease affecting the origin of the left gastric and splenic artery or coeliac axis (hitherto staged as M1a) are also eligible.

d) All patients should have a CT of chest and abdomen (pelvis is optional) prior to study entry. Patients with gastric and Siewert type II and III OGJ adenocarcinomas should also have a laparoscopy prior to study entry. Endoscopic ultrasound (EUS) should be performed for all **lower oesophageal and OGJ** adenocarcinomas and according to local practice for other tumours.

The following assessments should normally be performed within 6 weeks prior to randomisation (unless otherwise specified below). If assessments are > 6 weeks or results are borderline please contact the ST03 Trial manager who will refer your query to the Chief Investigator.

e) WHO performance status 0 or 1

f) Adequate respiratory function: FEV1 $\geq$ 1.5 litres (mandatory for patients with **lower oesophageal and OGJ** tumours only)

g) Adequate cardiac ejection fraction (as determined by ECHO or MUGA scan) $\geq$ 50% or $\geq$ your centres LLN for MUGA, BP controlled and normal ECG within 4 weeks prior to randomisation.

The following assessment should normally be performed within 4 weeks prior to randomisation.

h) Normal corrected QTc (corrected for heart rate).
(This is because caution is recommended if lapatinib is administered to patients who have or may develop prolongation of QTc.)

The following assessments should normally be performed within 1 week prior to randomisation and be as defined. If assessments are > 1 week or results are borderline please contact the ST03 Trial manager who will refer your query to the Chief Investigator.

i) Adequate bone marrow function
- Absolute neutrophil count (ANC) $\geq 1.5 \times 10^9$/l
- White blood cell count $\geq 3 \times 10^9$/l
- Platelets $\geq 100 \times 10^9$/l
- Haemoglobin (Hb) $\geq$ 9g/dl (can be post-transfusion)

j) Adequate renal function: glomerular filtration rate (GFR) $\geq$60ml/min calculated or measured. If the calculated GFR is <60ml/min then a measured GFR is required (see appendix I). The measured GFR should always take precedence over the calculated GFR.

k) Adequate liver function
- serum billirubin ≤1.5x ULN
- ALT/AST ≤2.5x ULN
- ALP ≤3x ULN

l) Adequate Coagulation profile:
- International normalised ratio (INR) $\leq$ 1.5
- Activated ProThrombin Time (APTT) $\leq$ 1.5xULN

m) Patients on oral anticoagulation are **advised to** change to low molecular weight heparin prior to randomisation, to be eligible. **(See 6.3.2 Medication to be used with caution)**

n) **Patients with high frequency hearing loss are eligible for ST03**. They should be treated with cisplatin but changed to carboplatin if there is any evidence of deterioration (see Section 8.3.7).

o) Patient is fit to receive all protocol treatment

p) Completion of baseline quality of life questionnaire

q) Women of childbearing potential should have a negative pregnancy test within 7 days prior to commencing treatment, or have had amenorrhea for more than 2 years. Fertile men and women must agree to take adequate contraceptive precautions.

r) Male/female patients aged ≥18 years

s) Written informed consent obtained before randomisation

6.2 Exclusion Criteria for Patients with HER-2 Postive Tumours Entering the Lapatinib Comparison

Patients should not be randomised if any of the exclusion criteria below are met:

Significant Co-Existing or Previous Medical Conditions

a) Cerebrovascular disease (including transient ischaemic attacks (TIA) and strokes) within 1 year before registration.

b) Cardiovascular diseases as follows:
- Myocardial infarction (≤ 1 year prior to registration)
- Uncontrolled hypertension while receiving chronic medication
- Angina requiring nitrate therapy within 1 year prior to randomisation
- New York Heart Association (NYHA) Grade II or greater congestive heart failure
- Serious cardiac arrhythmia requiring medication (for example, ventricular tachycardia, supraventricular tachycardia or atrial fibrillation with a resting heart rate > 110bpm)

c) Major surgery, major trauma or open biopsy within 28 days prior to registration (not including staging laparoscopy)

d) Serious non-healing wound, ulcer or bone fracture

e) Evidence of bleeding diathesis or coagulopathy

f) Recent history of any active gastrointestinal inflammatory condition such as peptic ulcer disease, diverticulitis or inflammatory bowel disease. If patients have a known diagnosis of any of the above, evidence of disease control is required by negative endoscopy within the past 28 days.

g) Patients with mild/intermittent tinnitus can be randomised to ST03 but **patients with more severe tinnitus should not be randomised.**

h) Patients who have received chemotherapy or radiotherapy for a previous malignancy.

Other Exclusion Factors

i) Lack of physical integrity of the upper gastro-intestinal tract, malabsorption syndrome, or inability to take oral medication. Patients who require placement of enteral feeding tubes for nutrition due to inability to swallow are not eligible for randomisation. Patients who have prophylactic feeding jejunostomy tubes placed at the time of staging laparoscopy would not be excluded but this is not recommended.

j) Positive serology for HIV, Hepatitis C or active Hepatitis B

k) Patients who have previously received anthracycline treatment

l) Known peripheral neuropathy ≥ Grade 1 (absence of deep tendon reflexes as the sole neurological abnormality does not render the patient ineligible)

m) Known dihydropyrimidine dehydrogenase (DPD) deficiency

n) Patients with a history of interstitial lung disease or radiological evidence of lung fibrosis are ineligible for this study.

o) Patients requiring ongoing treatment with contraindicated concomitant medication provided in Section 6.3. Please ensure that the patient will be able to comply should they wish to proceed to randomisation.

p) Patients who have received amiodarone 6 months prior to randomisation are ineligible. (See Section 6.3.1 for medications excluded within lapatinib comparison)

6.2.1 Additional Exclusion Criteria for the MRI Sub-Study only

Patients meeting the following exclusion criteria **must not** be registered into the MRI sub-study:

- Patients with a contraindication to MRI
- Patients with gastric or Siewart Type III OGJ tumours

6.2.2 Additional Exclusion Criteria for the PET/CT Sub-Study only

Patients meeting the following exclusion criteria **must not** be registered into the PET/CT sub-study:

- Poorly controlled diabetes mellitus
- Non-FDG-avid and structurally non-measurable lesions on pre-treatment ^{18}F-FDG PET/CT and contrast enhanced CT respectively
- Unable to carry out PET/CT scan

6.3 Guidance on Concomitant Medication for Patients with HER-2 Positive Tumours Entering the Lapatinib Comparison

6.3.1 Excluded Concomitant Medications

Any patient receiving the following concomitant medication should **not** be considered for ST03 randomisation to the lapatinib comparison:

a) Dipyridamole use should be avoided and concomitant administration of capecitabine and sorivudine (or sorivudine analogues e.g. brivudine) is contraindicated. Patients receiving phenytoin concomitantly with capecitabine should be regularly monitored for increase phenytoin plasma concentrations and associated symptoms.

b) Lapatinib is a substrate for cytochrome P450 (CYP) 3A4, therefore inducers or inhibitors of CYP3A4 may alter the metabolism of lapatinib and are therefore not permitted for HER-2 positive patients during the study, or for 7 days prior to study entry for CYP3A4 inhibitors (14 days for CYP3A4 inducers). A list of these drugs is provided below:

CYP3A4 inhibitors (7-day washout required) include:

- Antibiotics clarithromycin, erythromycin and flucloxacillin
- Anti-fungals ketoconazole, itraconazole, voriconazole and fluconazole (>150mg/day)

- Anti-retrovirals delaviridine, nelfinavir, amprenavir, ritonavir, indinavir, saquinavir, lopinivir, atazanavir;
- Calcium channel blockers verapamil and diltiazem
- Anti-depressants nefazodone and fluvoxamine; cimetidine
- Grapefruits, grapefruit juice star fruit or papaw are not permitted during treatment with lapatinib or for 7 days prior to entry

CYP3A4 inducers (14-day washout required) include:
- Anti-TB drugs rifampicin, rifabutin and rifapentine
- Anticonvulsants phenytoin, carbamazepine and barbiturates (e.g. phenobarbital)
- Antiretrovirals efavirenz, nevirapine, tipranivir and etravirine
- Glucocorticoids cortisone (>50 mg), hydrocortisone (>40 mg), prednisone (>10 mg), methylprednisolone (>8 mg) or dexamethasone (>1.5 mg other than for 3-5 days after ECX chemotherapy with each cycle to prevent nausea and vomiting);

Additionally, patients that have received amiodarone within 6 months of study entry are ineligible for randomisation to the lapatinib comparison and St. John's wort, modafinil, ginkgo biloba, kava, grape seed, valerian, ginseng, echinacea and evening primrose oil should not be taken by patients during treatment with lapatinib or for 14 days prior to study entry.

6.3.2 Medications to be used with caution

- Patients receiving concomitant capecitabine and oral anticoagulants should have their anticoagulant response (INR or prothrombin time) monitored frequently in order to adjust the anticoagulant dose accordingly. Altered coagulation parameters and/or bleeding, including death, have been reported in patients taking capecitabine concomitantly with coumarin-derived anticoagulants such as warfarin and phenprocoumon. A pharmacokinetic interaction has been observed. The use of low molecular weight heparin instead of coumarin is advised but at the discretion of the Investigator.

Other cytotoxic agents or investigational drugs and radiotherapy are prohibited in this study unless there is evidence of disease progression. Any additional treatment that the responsible physician feels is appropriate is permitted.

6.3.3 Data on concomitant medication

All concomitant medication will be recorded on any subsequent Serious Adverse Event (SAE) forms.

6.4 Pre-Randomisation Investigations and Screening Procedures

Pre-randomisation investigations and screening procedures should be performed whilst awaiting the results of the HER-2 test.

6.4.1 Screening Assessments

The following screening investigations should normally be performed **within 4 weeks** prior to randomisation**:**

- Clinical history and examination, including blood pressure.
  If BP <u>is uncontrolled</u> patient should be commenced on an ACE inhibitor or other antihypertensive agent until controlled prior to randomisation. (Appendix E for Guidance on Treating Hypertension).

- ECG

- Baseline Echocardiogram (ECHO) or MUGA scan. Left Ventricular Ejection Fraction (LVEF) must be ≥50% or ≥ your centre's LLN for MUGA if this is less than 50%. There should also be an absence of other abnormal findings, such as abnormal wall motion.

- Assessment of hearing and associated interpretation of results and administration of chemotherapy can be according to local practice but please note that patients with severe tinnitus **should not** be randomised.

<u>If any of the above investigations cannot be performed within 4 weeks prior to randomisation, then the case should be discussed with the MRC CTU.</u>

The following assessments should normally be performed **1 week before randomisation:**

- Renal function assessment. Calculated GFR as per local guidelines. If the calculated GFR < 60ml/min, or the serum creatinine is above the ULN for the local centre then a measured GFR is required. **(see Appendix I Cockroft Gault formula).**
- Baseline FBC, serum urea and electrolytes, creatinine and liver function tests and coagulation profile to include INR and APTT. (see inclusion criteria section 6.1 for more details)

<u>If one or more of the above investigations cannot be completed within 1 week prior to randomisation then the case should be discussed with the MRC CTU.</u>

The above is summarised, along with the other assessments required throughout the trial in Table 9.1 ST03 Trial Assessments (**see Section 9**)

6.4.2 Tumour Staging Investigations

A CT scan must be performed prior to registration to confirm no metastasis, however all further staging investigations can be performed whilst awaiting the HER-2 test result.

Each of the tumour staging investigations outlined below should be aimed at being performed within 4 weeks prior to randomisation. However if this is not possible, the last staging investigation, which may include CT, EUS, PET/CT or laparoscopy, **should normally be performed within 4 weeks prior to randomisation.** If the last staging investigation is >4 weeks please contact the ST03 Trial Manager who will refer the query to the Chief Investigator. The results of an EUS should be used to stage **local disease** (depth of tumour invasion and nodal status), whereas CT scan should take precedence when staging distant disease spread.

1. Histological confirmation of diagnosis and baseline staging investigations must be reviewed by a Multidisciplinary Team (MDT) to confirm patient's eligibility for the trial.
2. Staging investigations should include:
    - Laparoscopy for all patients with gastric and Siewert type II and III OGJ, but **not** Siewert type I OGJ/lower oesophageal cancers
    - Spiral/multi-slice CT with oral contrast or water including chest, abdomen (pelvis is optional). Maximum slice width 5mm. IV contrast/venous phase.

- Endoscopic ultrasound (EUS) should be performed for all lower oesophageal, Siewert type I, II or III gastro-oesophageal adenocarcinomas and according to local practice for all other tumours.

PET scans, MRI or bone scans may be used where clinically indicated according to local practice.

6.4.3 Baseline MRI Scan (only applicable for the MRI Sub-study)

For those centres who are participating in the MRI sub-study the baseline MRI scan must only be performed once the staging and screening investigations have confirmed eligibility for the MRI sub-study, listed in Sections 6.1 and 6.2.

The site must ensure that informed consent is obtained from the patient prior to the baseline MRI scan being performed.

The baseline MRI scan should be performed as close to the staging investigations as possible once the patient has been confirmed to be eligible for registration into the MRI sub-study and **must** be performed prior to the patient commencing treatment. To assist with this, the baseline MRI scan may be performed prior to randomisation provided the patient has been confirmed eligible and consented.

Please see section 14 for details on the MRI sub-study procedures.

6.4.4 Baseline PET/CT Scan (only applicable for the PET/CT Sub-study)

For those centres who are participating in the PET/CT sub-study the baseline PET/CT can be performed prior to consent and randomisation as part of the standard clinical care. The baseline PET/CT scan should be performed as close to the staging investigations as possible and **must** be performed prior to the patient commencing treatment.

Please note that the cycle 1 (day 14 – 21) FDG PET/CT can only be performed after consent for the sub-study has been obtained from the patient.

Please see section 15 for details on the PET/CT sub-study procedures.

7. Randomisation of Patients with HER-2 Positive Tumours

Patients with HER-2 Positive tumours must meet all of the inclusion criteria and none of the exclusion criteria listed in Sections 6.1 and 6.2 and the patient must not be receiving any of the excluded concomitant medications listed within Section 6.3.

7.1 Written Informed Consent

Before a patient is randomised to the lapatinib comparison, written informed consent for entry into the trial must be obtained.

Written confirmation that the patient has given their consent to participate in the trial should be recorded by a qualified, experienced nurse or a clinician according to local practice.

7.1.1 Lapatinib Comparison Consent

Eligible patients with HER-2 positive tumours should be given **PIS B for patients with HER-2 positive tumours.** Once the patient has had adequate time to read the information they will be required to sign **Consent Form B for patients with HER-2 positive tumours** prior to randomisation to the lapatinib comparison.

If the patient also wishes to participate in the MRI and/or PET-CT sub-studies then they should be given the relevant PIS and asked to sign the appropriate consent form.

Patients who give their written informed consent to participate in the trial can only be randomised to the trial if they fulfil all the eligibility criteria stated in Section 6.1 and 6.2 and are not taking any excluded medications listed in Section 6.3.

7.1.2 MRI Sub-Study Consent

Centres participating in the MRI sub-study must ensure that the patient is introduced to the **MRI sub-study PIS** prior to obtaining consent within the **MRI Sub-study Consent Form.** It will be explained to the patient that they will only receive the baseline MRI scan once they have been confirmed eligible for the MRI sub-study. The site must ensure that written informed consent has been obtained prior to the baseline MRI scan being performed.

7.1.3 PET/CT Sub-Study Consent

Centres participating in the PET/CT sub-study must ensure that patients eligible for the PET/CT sub-study are introduced to the **PET/CT Sub-study PIS** prior to obtaining consent within the **PET/CT Sub-study Consent Form.**

Please note that the cycle 1 (day 14 – 21) FDG PET/CT can only be performed after consent for the sub-study has been obtained from the patient.

7.1.4 ST03-Trans Sub-Study Consent

Participation in the blood and tissue collection for the translational research study is open to all sites and strongly encouraged. In order to collect tissue and blood samples for future translational research, patients **must** sign the **Trans-ST03 Consent Form**. Although most patients are expected to consent to participation in the translational study, the wishes of patients who do not want to be involved in the translational research part of the ST03 trial will be respected and they will be allowed to enter the clinical trial only.

20ml EDTA blood should be collected from the patients that consent to Trans-ST03 before commencing ST03 treatment.

For more information on the tissue and blood collection, please see Section 32 Pathology Research.

7.3 Randomisation Procedure for Patients Entering the Lapatinib Comparison

Once the eligibility criteria for randomisation have been confirmed and informed consent has been obtained, it is recommended that the following CRFs are completed before calling the randomisation line as questions will be asked from each of these forms:

- Randomisation Form (ST03/1-HER)
- Pre-treatment Screening Form (ST03/2-HER)

- Baseline QoL questionnaire including EQ5D (must be completed before the patient is informed which treatment they have been allocated).

**To enter a patient on the trial telephone the MRC CTU randomisation line:**<br>**+44 (0)207 670 4777 (9am - 5pm, Mon-Fri)**

AT RANDOMISATION THE CORRECT DOSE LEVEL FOR ARM D THE MODIFIED ECX + LAPATINIB REGIMEN WILL BE CONFIRMED

When randomising the patient **please ensure that the MRC CTU is aware that the patient has been registered previously** and state the trial number provided at registration. You will be asked to confirm the patient initials and date of birth to verify that the correct patient is being randomised. The trial number will remain the same and a treatment will be allocated and given over the phone. In addition, a letter confirming these details and the patient's treatment schedule will be sent to the randomising centre. The trial number will be a unique identifier and the primary way in which the patient will be identified and should be used in all correspondence.

If the patient has also consented to the MRI and/or PET/CT sub-studies you will be asked to confirm this during the randomisation call and the patient will be registered for the sub-studies as applicable.

7.4 Patient Screening and Enrolment Log

The investigator should keep a patient screening and enrolment log of all patients being considered for the ST03 trial. This should include patients tested for HER-2 status who failed screening for the ST03 lapatinib comparison.

The Randomisation Form (ST03/1-HER) should also be completed to detail patients who are registered for HER-2 testing but are subsequently be found to be ineligible for, or may decline, randomisation into the lapatinib comparison.

7.5 Co-enrolment Guidelines

Ideally, patients should not be participating in any other clinical trials unless this has been discussed with, and agreed to, by the ST03 TMG previously. The primary endpoint for ST03 as a whole is overall survival and follow up should continue until this endpoint is met. The ST03 TMG should be contacted if co-enrolment is being considered.

8. Treatment of Patients

8.1 General Principles

- Treatment should normally commence **within 1 week** of randomisation (or registration into the MRI and/or PET/CT sub-studies). Patients that cannot be treated within 1 week of randomisation/registration should be discussed with the MRC CTU.
- The PI is responsible for ensuring that the protocol is followed.
- A Full Blood Count (FBC) should be done within 5 days prior to the start of cycle 1 and either on day 1 or up to 3 days before for all subsequent treatment cycles.

- Dose modifications should only be made after consulting the protocol (if in doubt please discuss with the MRC CTU).
- Body Surface Area (BSA) should be re-calculated before each cycle of chemotherapy. Treatment doses should be based on the re-calculated BSA value or according to local practice. BSA should be calculated using the Dubois and Dubois formula or nomogram. **(see Appendix H).**
- For obese patients with a Body Mass Index (BMI) > 30, BSA should be capped at $2.2m^2$.
- Granulocyte-Colony Stimulating Factor (G-CSF) may be used if it is local practice.
- Dose banding is allowed if it is local practice. If you need to use your local dose banding for the capecitabine, the dose banding schedule should be supplied to the MRC CTU.

The worst toxicity grade observed during the cycle should be recorded in the Toxicity Assessment section of the cycle CRFs (ST03/3-HER and ST03/8-HER).

8.1.1 Nadir Blood Test

All patients must have a nadir blood count during their cycle 1 treatment (at approximately day 10 (+/- 2 days) of treatment).

Any patients who experience Grade 4 (<0.5 x 109/L) neutropenia must start Granulocyte Colony Stimulating Factor (G-CSF). This could be Filgrastim 300µg once daily sub-cut injection for 3-5 days, or lengograstim according to local practice. Pegylated Filgrastim (Neulasta) may also be used but should be administered at least 24 hours after intravenous chemotherapy to reduce the risk of potentiation of myelosupression. It is recommended that patients who experience Grade 3 (<1 – 0.5 x 10^9/L) neutropenia should also be treated with G-CSF, but this is at the treating investigator's discretion.

The cycle 1 nadir count result should be emailed to the ST03 Trial Team at MRC CTU as soon as they have been performed unless your centre has been informed that this is not required.

It is recommended those patients registered into the MRI and/or PET/CT sub-studies **only** should have a nadir blood count performed during cycle 1 treatment as directed above. However the result of this nadir count does not need to be sent to the ST03 trial team. For further details on the required and recommended assessments for patients registered to the MRI and/or PET/CT sub-studies only please refer to Section 13.

All patients who are treated for Grade 3-4 neutropenia in cycle 1 must also receive G-CSF during all further cycles of sECX or mECX+Lapatinib. If the patient subsequently receives E-Carbo-X the G-CSF must be continued. The Investigator may wish to check the patients' nadir blood counts during future cycles, for example if the patient experiences Grade 2 neutropenia during cycle 1, however this is at the discretion of the PI.

For those patients who experience Grade 3 or 4 neutropenia on day 1 of subsequent cycles the dose reduction recommendations within Sections 8.3.3 and 8.5.1 should be followed and GCSF can be administered as per local practice.

For those patients who are hospitalised prior to day 10, centres should follow local standard procedure to monitor neutrophil count, but if grade 4 neutropenia documented then G-CSF is required in subsequent cycles.

8.1.2 Cardiac Monitoring and treatment modifications

Patients randomised into the lapatinib comparison (Arm C: sECX or Arm D: mECX+L) are required to have at least two additional cardiac assessments, one before surgery and the other at the end of post-operative chemotherapy. These additional cardiac assessments are not required for patients who register in to the MRI and/or PET/CT sub-studies **only** and are **not** randomised into the lapatinib comparison. For further details on the required assessments for patients registered to the MRI and/or PET/CT sub-studies only please refer to Section 13.

Please note that a reduction in LVEF at the pre-surgery cardiac assessment from the baseline assessment may require epirubicin and lapatinib to be omitted from the post-surgery chemotherapy (see **Section 9.2.2** for full details). Similarly the results of the post chemotherapy cardiac assessment may require lapatinib to be omitted during maintenance therapy (see **Section 9.3.2** for full details).

All patients (including those registered for the MRI and/or PET/CT sub-studies only) with symptoms consistent with heart failure should undergo physical examination, ECG, cardiac troponin measurement, chest x-ray and ECHO/MUGA as soon as possible. Those with left ventricular impairment, LVEF <50% for ECHO (or <LLN for MUGA), should be started on an ACE-inhibitor unless contraindicated and referred to a cardiologist. **Lapatinib and epirubicin should be permanently discontinued for these patients.**

Asymptomatic patients, in whom a ≥10% fall in LVEF to <50% for ECHO (or <LLN for MUGA) is noted at one of the planned cardiac assessments within the lapatinib comparison arms (Arms C and D), should undergo reassessment with physical examination, ECG, cardiac troponin measurement and chest x-ray. **Lapatinib and epirubicin should be permanently discontinued.** The patient should be started on an ACE-inhibitor unless contraindicated. Patients should be reassessed including repeat ECHO/MUGA 4-6 weeks later. If they have developed symptoms consistent with heart failure or there is no improvement in LVEF the patient should be referred to a cardiologist.

Patients in whom a <10% fall in LVEF to <50% for ECHO (or <LLN for MUGA) is noted at one of the planned cardiac assessments within the lapatinib comparison arms (Arms C and D), should be started on an ACE-inhibitor unless contraindicated. Lapatinib and epirubicin should be withheld. A further cardiac assessment should be taken 4-6 weeks later, if there is no improvement in LVEF or LVEF has decreased the patient should be referred to a cardiologist and lapatinib and epirubicin should be permanently discontinued. If the LVEF recovers to >50% (or >LLN for MUGA) then lapatinib and epirubicin may be continued.

Please see Sections 9.2 and 9.3 for further information on the requirement for cardiac assessment within the lapatinib comparison and for instruction on the management of reduction in LVEF.

8.1.3 Measures of Compliance / Adherence

Date of treatment, dose, delays and reasons for delays or dose modifications of all study drugs infusions should be recorded on the Treatment CRF (ST03/3 and ST03/8) for all patients randomised to the lapatinib comparison. (Treatment CRFs are not required for those patients who are registered into the MRI and/or PET/CT sub-studies only. See Table 13.2 for CRF schedule for these patients.)

8.1.4 Capecitabine & Lapatinib Diary Cards

In order to assess patient compliance in taking the full prescribed capecitabine and lapatinib doses per cycle, patients will be issued with a capecitabine diary card and a lapatinib diary card (Arm D only) to complete for each cycle. Research staff should ensure that the diary card is collected at the end of each cycle and for patients randomised to the lapatinib comparison the number of unused tablets, if any, are recorded on the treatment CRF (ST03/3-HER and ST03/8-HER). (Diary Cards are not required for those patients who are registered into the MRI and/or PET/CT sub-studies only. See Table 13.2 for CRF schedule for these patients.)

8.2 Administration of Control Arm C: Standard sECX

The control arm consists of 3 pre-operative cycles of sECX given over a period of 9 weeks at 3 weekly intervals followed by surgery 5-6 weeks after completion of pre-operative chemotherapy. 6-10 weeks later, 3 post-operative cycles of sECX are administered over 9 weeks.

Pre-operative Chemotherapy

- 3 cycles of epirubicin 50 mg/m^2 IV and cisplatin 60 mg/m^2 IV Day 1 and capecitabine 1250mg/m^2/day.
- The daily dose of capecitabine is based on 1250mg/m^2 /day but will be rounded to the nearest achievable dose based on three BSA levels and will be administered according to Table 8.1, unless a copy of your local dose banding schedule has been supplied to the CTU.
- The capecitabine will be administered *per oral* (PO) in 2 divided doses continuously from Days 1 to 21 per cycle (see Table 8.1).
- Capecitabine tablets are available in 500mg and 150mg and should be administered morning and evening and swallowed with water. If the total daily dose requires uneven distribution of tablets then the larger dose should be given in the evening. Administration of capecitabine should be within 30 minutes (before or after) a meal.
- Standard anti-diarrhoeal treatments (e.g. loperamide) may be used, whilst receiving capecitabine.
- Each cycle is repeated every 21 days for 3 cycles.

Table 8.1: sECX Capecitabine Dose Calculation, According to Body Surface Area

BSA (m^2)	Total daily dose (mg)	Number of tablets administered in the morning		Number of tablets administered in the evening	
		150mg	500mg	150mg	500mg
<1.6	1800	2	1	0	2
1.6-1.8	2150	0	2	1	2
>1.8	2500	0	2	0	3

Example of how to calculate the capecitabine total cycle dose*, which is required on the Treatment CRF (ST03/3 and ST03/8) is given below.*

If BSA is 1.7, then the total daily dose prescribed will be 2150mg.
Total cycle dose is 2150 x 21 day = 45150 mg

An example of how to calculate the total daily dose of capecitabine and the number of tablets to administer in the morning and evening is given in **Appendix K**

Problems Administering Capecitabine

For patients who have difficulty swallowing capecitabine, the tablets can be dissolved in water. Place capecitabine tablets in approximately 200ml of water. By agitating the

tablets for approximately 15 minutes, the tablets should dissolve. The tablets should be dissolved immediately before use and the solution swallowed immediately as there is no stability data for any form of suspension. The solution may also be administered through a naso-gastric or other enteral feeding tube. The solution has a very bitter taste and a fruit juice can be added to make the solution more palatable, but capecitabine should not be mixed with grapefruit juice.

If a patient vomits after taking a dose of capecitabine, the dose should not be taken again.

A recommended schedule for administrating epirubicin and cisplatin is given in Table 8.2. Local policy for administering sECX may be substituted if preferred, ensuring adequate hydration for cisplatin. However, the dose of cisplatin should not be split and the treatment should always be given over one day only.

Table 8.2 Recommended Epirubicin and Cisplatin hydration

**Time (T)**	**Recommended Medication**
T=-1 hour<br>IV chemotherapy	Patients will receive frusemide 40mg IV bolus or *po* and sodium chloride 0.9% (normal saline) 1 litre + 20mmol KCl + 10mmol $MgCl_2$ IV over 1 hour
T=0hr Prior to administration of IV chemotherapy	Patients should receive as anti-emetics Granisetron 1mg IV bolus (2mg if >100kg) and dexamethasone 8mg IV bolus
T=0hr	**Epirubicin** 50mg/m^2 will be administered as an IV bolus via fast running drip
T=0hr	**Cisplatin** 60mg/m^2 IV will be administered in sodium chloride 0.9% (normal saline) 1 litre + 20mmol KCl over 4 hours
T=0hr	Mannitol 10% 200ml should be administered IV over 4 hours concurrently with cisplatin
T=+4hr	Sodium chloride 0.9% (normal saline) 1 litre + 20 mmol KCl + 10mmol $MgCl_2$ should be administered IV over 2 hours
T=+6hr	Sodium chloride 0.9% (normal saline) 500mls + 10 mmol KCl should be administered IV over 1 hour

Oral anti-emetics:
Patients should be provided with oral anti-emetics and guidance on when they should be taken as follows:

- Dexamethasone 4mg *po tds* Day 2-4
- Metoclopramide 10mg *po tds* Day 1-3

Local policy for administering anti-emetics, including the use of 5-HT_3 antagonists and aprepitant, may be substituted if preferred.

Post-operative Chemotherapy
Post-operative chemotherapy is identical to the pre-operative chemotherapy and should commence 6-10 weeks after surgery. Epirubicin 50 mg/m^2 IV and cisplatin 60 mg/m^2 IV Day 1, with capecitabine 1250mg/m^2 po daily in 2 divided doses given continuously from Days 1 to 21. Cycle repeated every 21 days for a further three cycles (see above for details).

8.3 Toxicities and Dose Modifications for Standard sECX

Please note that if the dose of capecitabine is stopped for any reason, **the doses are omitted, not delayed** and capecitabine treatment should stop at day 21 of each cycle

and not continue until all tablets have been taken. For patients randomised to the lapatinib comparison, any dose modifications that remain from a previous cycle should be indicated as modifications on all subsequent treatment CRFs.

8.3.1 Dihydropyrimidine Dehydrogenase Deficiency

With any 5FU regimen, the occasional patient is encountered (approximately 1-3%) who has markedly exaggerated toxicity due to reduced 5FU catabolism. If this occurs, await full recovery and then re-start capecitabine at a 50% reduction.

8.3.2 Haematological Toxicities

Modifications due to toxicity in previous cycle**:**

If at any time during the previous cycle, infection/fever associated with neutropenia has occurred e.g.:
CTC grade 3 (neutrophil count <1.0x10^{9}/l), delay all chemotherapy until neutrophil count recovers, then dose reduce epirubicin by 25% of the previous cycle.

CTC grade 4, (neutrophil count <0.5x10^{9}/l), delay all chemotherapy until neutrophil count recovers then dose reduce epirubicin by 50% of the previous cycle.

Dose reductions relate to the dose of the previous cycle and should be maintained in subsequent cycles.

8.3.3 Modification Based on Day 1 FBC

Check FBC on day 1 of (or up to 3 days before) each cycle.
If neutropenia/thrombocytopenia (in the absence of infection/fever) is present on the day treatment is due, delay treatment until neutrophil count recovers to $\geq$1.0 x 10^{9}/l and platelet count recovers to $\geq$75 x 10^{9}/l, then dose reduce as indicated in tables 8.3 and 8.4. Dose reductions related to epirubicin should apply to all subsequent cycles.

GCSF may be used if this is local practice.

Please note that the **greatest reduction for toxicity** should be followed.

For example if there is grade 4 neutropenia and grade 2 thrombocytopenia on day 1 of treatment, capecitabine should be stopped and treatment should be delayed until blood counts have recovered. Capecitabine should be restarted at full dose, epirubicin should be reduced ***by 50%*** *and cisplatin should be given at full dose.*

Table 8.3: Dose Modifications for sECX based on Neutrophil Count (on day of treatment)

Neutrophil count (x10^{9}/l)	CTC grade	Action
≥1.0	0-2	Full dose of all drugs
0.5-0.9	3	Stop capecitabine and delay epirubicin and cisplatin until recovery (e.g. 1 week later). Restart capecitabine at full dose.<br>Reduce epirubicin by 25% on subsequent cycles.<br>Restart cisplatin at full dose<br>GCSF may be used as per local practice
<0.5	4	Stop capecitabine and delay epirubicin and cisplatin until recovery (e.g. 1 week later).<br>Restart capecitabine at full dose.<br>Reduce epirubicin by 50% on subsequent cycles.<br>Restart cisplatin at full dose<br>GCSF may be used as per local practice

Table 8.4: Dose Modifications for sECX based on Platelet Count (on day of treatment)

Platelet count ($x10^9/l$)	CTC grade	Action
≥75	0-1	Full dose of all drugs
50-74	2	Stop capecitabine and delay epirubicin and cisplatin until recovery (e.g. 1 week later).<br>Restart capecitabine at full dose.<br>Restart cisplatin at full dose.<br>Reduce epirubicin by 25% on subsequent cycles.
25-49	3	Stop capecitabine and delay epirubicin and cisplatin until recovery (e.g. 1 week later).<br>Restart capecitabine at full dose.<br>Restart cisplatin at full dose.<br>Reduce epirubicin by 50% on subsequent cycles.
<25	4	Stop capecitabine and delay cisplatin until recovery (e.g. 1 week later).<br>Restart capecitabine at full dose.<br>Restart cisplatin at full dose.<br>Omit epirubicin from subsequent cycles.

8.3.4 Cardiac Toxicity

- **Cardiac Failure:** All patients (including those registered for the MRI and/or PET/CT sub-studies only) with symptoms consistent with heart failure should undergo physical examination, ECG, cardiac troponin measurement, chest x-ray and ECHO/MUGA as soon as possible. Those with left ventricular impairment (LVEF <50% for ECHO or <LLN for MUGA) should be started on an ACE-inhibitor unless contraindicated and referred to a cardiologist. **Epirubicin and lapatinib (if randomised to Arm D) should be discontinued permanently for these patients.**
- **If LVEF is <50% for ECHO (or <LLN for MUGA) at any of the planned cardiac assessment within the lapatinib comparison arms (Arms C and D) please refer to Section 9.2.2 and 9.3.2 for further details on patient management.**
- **Fluoropyrimidine-related chest pain:** Fluoropyrimidines are known to rarely cause a syndrome of angina like chest pain, which may be associated with coronary artery spasm.
  - If patients develop angina like pain whilst receiving capecitabine, then treatment should be **discontinued immediately** pending further assessment.
  - An ECG must be performed and serum troponin measured. Patients should be admitted overnight if significant pain has occurred within the previous 24 hours (with repeat ECGs and serial troponin).
  - If abnormalities are found on ECG or serial cardiac marker levels, then a cardiology opinion should be considered.

- If chest pain is deemed to be capecitabine related, patients should not recommence treatment with capecitabine. The ST03 Trial Manager should be contacted and the query will be referred to the CI to discuss suitable alternatives.

- **Grade 3 or 4 arterial thromboembolic** events (including transient ischaemic attacks (TIA), cerebrovascular accident (CVA) and myocardial infarction (MI)) and **Grade 3 or 4 congestive heart failure or left ventricular dysfunction** (symptomatic heart failure, LVEF <40% or a ≥20% reduction in LVEF to below LLN) are Notable Events within the lapatinib comparison (Arms C and D) and should be reported to the trials unit on a SAE form. See Section 17.5 for further details on additional Notable Events. (Notable events are not required to be reported for patients registered into the MRI and/or PET/CT sub-studies only)
- Caution should be taken if Lapatinib is administered to patients who have or may develop prolongation of QTc. These conditions include patients with hypokalemia or hypomagnesemia, congential long QT syndrome, patients taking anti-arrhythmic medicines or other medicinal products that lead to QT prolongation. Hypokalemia, hypocalcemia or hypomagnesemia should be corrected prior to lapatinib administration.

8.3.5 Liver Toxicity

- **Bilirubin** If bilirubin increases to >1.5xULN (upper limit of normal range), epirubicin should be omitted until bilirubin returns to acceptable levels i.e. ≤1.5xULN.
- **Raised Transaminases** Capecitabine undergoes hepatic metabolism. Patients on capecitabine may have temporary treatment-related elevation of transaminases. An isolated rise in transaminase above 5xULN during treatment is likely to be treatment-related, and capecitabine should be interrupted until recovery i.e. ≤2.5xULN.

8.3.6 Renal Toxicity

Creatinine clearance should be calculated or measured at baseline and prior to each cycle of chemotherapy. Calculations can be made using the Cockcroft Gault formula given in **Appendix I** or according to local practice. If the serum creatinine is above normal then creatinine clearance should be measured not estimated. The Cockcroft Gault formula usually under estimates GFR compared with EDTA or measured 24 hr creatinine clearance. Therefore if the calculated creatinine clearance falls below 60mls/min then the measured GFR should be checked. If the measured creatinine clearance is < 60mls/min then the dose reductions of both cisplatin and capecitabine should be made according to Table 8.5.

Table 8.5: Cisplatin and Capecitabine dose modifications for sECX

Creatinine clearance	Cisplatin Dose	Capecitabine Dose
**≥ 60 mls/min**	100%	100%
**50-59 mls/min**	50%	100%
**40-49 mls/min**	50%	75%
**30-39 mls/min**	Replace cisplatin with carboplatin	75%

Capecitabine can resume at normal dose upon recovery of renal function.

The cisplatin dose should continue at the reduced level.

Substituting Cisplatin with Carboplatin

In patients whose creatinine clearance is <40mls/min, cisplatin should be replaced by carboplatin at a dose of AUC5 (area under the curve), so that patients receive epirubicin, carboplatin and capecitabine (E-Carb-X). Patients receiving E-Carb-X should have a neutrophil count of $\geq$1.5 x10^9/l and a platelet count of $\geq$100 x10^9/l on the day of treatment.

8.3.7 Neurotoxicity / ototoxicity

Patients with CTC grade 2 or greater neurotoxicity or new functional deterioration in hearing, new tinnitus or new significant high frequency hearing loss on audiogram should have cisplatin replaced with carboplatin at a dose of AUC5, so that patients receive epirubicin, carboplatin and capecitabine (E-Carb-X)

8.3.8 Plantar-Palmar erythema (PPE)

- For CTC grade 2, stop capecitabine. On resolution of toxicity to $\leq$ grade 1, restart capecitabine with 15% dose reduction.
- For CTC grade 3, stop capecitabine. On resolution of toxicity to $\leq$ grade 1, restart capecitabine with 30% dose reduction.
- For recurrent CTC grade 3, stop capecitabine. On resolution of toxicity to $\leq$ grade 1, restart capecitabine with 50% dose reduction.

When capecitabine is stopped for toxicity the doses are omitted, not delayed.

8.3.9 Stomatitis, Diarrhoea, Nausea and Vomiting

For CTC grade 2-3 toxicity, stop capecitabine and administer appropriate symptomatic management (e.g. sucralfate for stomatitis, codeine phosphate for diarrhoea). If toxicity is adequately controlled with symptomatic measures alone within 2 days, then capecitabine may be restarted at 100% full dose. If toxicity persists, dose reductions as indicated in Table B4.6 should be made. Doses of capecitabine should be rounded to the nearest 150mg tablets

When capecitabine is stopped for toxicity the doses are omitted, not delayed.

Table 8.6: Management of Stomatitis, Diarrhoea, Nausea and Vomiting

Incidence	Grade 2	Grade 3	Grade 4
1st appearance	Interrupt treatment until resolved to grade 0-1, then continue capecitabine at same dose.	Interrupt treatment until resolved to grade 0-1, then continue capecitabine at 75% of original dose with prophylaxis where possible.	Discontinue treatment (if the PI considers it to be in the best interest of the patient to continue at 50% of original dose, once toxicity has resolved to grade 0-1 this should first be discussed with the MRC CTU who will confirm this with the CI.)
2nd appearance of same toxicity	Interrupt treatment until resolved to grade 0-1, then continue capecitabine at 75% of original dose.	Interrupt treatment until resolved to grade 0-1, then continue capecitabine at 50% of original dose.	Discontinue treatment
3rd appearance of same toxicity	Interrupt treatment until resolved to grade 0-1, then continue capecitabine at 50% of original dose.	Discontinue treatment	N/A
4th appearance of same toxicity	Discontinue treatment	N/A	N/A

8.4. Administration of the Investigational Arm D: mECX+L

The instructions provided for dose modifications due to toxicity within Section 8.2 for the sECX arm (Arm C) should be followed for the mECX+L regimen. The dose modifications are provided as percentage reductions and can therefore be applied to the mECX+L treatment regimen.

PLEASE NOTE THAT THERE MAY BE CHANGES IN THE DOSES OF CAPECITABINE AND LAPATINIB.

THE TRIAL WILL START WITH DOSE LEVEL 0 – SEE TABLE 8.7 BELOW

All patients randomised to Arm D (mECX+L) should be prescribed loperamide (2-4mg prn) with each cycle, to be taken only in the event of diarrhoea.

8.4.1 Safety assessment and criteria for dose level escalation or reduction

Due to the potential for overlapping toxicity between ECX and lapatinib (diarrhoea), **the first 10 patients randomised to treatment with mECX+L will be treated with a reduced dose of capecitabine (1000mg/m^2/day)** indicated by dose level 0 below in Table 8.7. A formal safety assessment will take place after 10 patients have completed pre-operative mECX+L. Dose escalation of capecitabine to 1250mg/m^2/day (dose level +1) will be made if grade 3-4 diarrhoea is reported in less than 2/10 patients. If grade 3-4 diarrhoea is reported in 2/10 patients, then a further 10 patients will continue to be treated at the reduced dose of capecitabine (1000mg/m^2/day), see dose level 0 in Table 8.7below. If more than 2 out of 10 patients develop grade 3-4 diarrhoea, then a dose reduction to lapatinib to 1000mg/day will be made (dose level -1). Following a dose level escalation or reduction, toxicity will again be formally reviewed after 10 patients have completed pre-operative mECX+ lapatinib at the new dose level. The dose levels are summarised in Table 8.7 below:

Table 8.7 Dose levels for modified ECX + lapatinib

Dose level	Epirubicin mg/m^2	Cisplatin mg/m^2	Capecitabine mg/m^2/day	Lapatinib mg/day
+1	50	60	**1250**	1250
0 **(starting dose)**	50	60	1000	1250
-1	50	60	1000	**1000**

Centres will be informed at randomisation as to which dose level is currently being used.

Maintenance lapatinib will be delivered at a dose of 1500mg/day at all initial dose levels of mECX+L unless patients have required a further dose reduction to lapatinib due to toxicity. Patients that required a dose reduction to lapatinib due to toxicity should receive maintenance therapy starting at 1250mg/day. If no Grade 3/4 toxicity is experienced after 21 days then this dose may be escalated to 1500mg/day.

A formal safety review will be conducted once 10 patients have received at least one cycle of lapatinib maintenance monotherapy.

8.4.2. mECX Capecitabine dose level 0

Table 8.8: mECX Capecitabine Dose Calculation, According to Body Surface Area

BSA (m^2)	Total daily dose (mg)	Number of tablets administered in the morning		Number of tablets administered in the evening	
		150mg	500mg	150mg	500mg
<1.6	1500	0	1	0	2
1.6-1.8	1800	2	1	0	2
>1.8	2000	0	2	0	2

Example of how to calculate the capecitabine total cycle dose*, which is required on the Treatment CRF (ST03/3 and ST03/8) is given below.*

If BSA is 1.7, then the total daily dose prescribed will be 1800mg.
Total cycle dose is 1800 x 21 day = 37800 mg

An example of how to calculate the total daily dose of capecitabine and the number of tablets to administer in the morning and evening is given in **Appendix K**

8.4.3. Administration of Lapatinib

Oral lapatinib will be supplied as 250 mg tablets. During the 6 cycles of mECX+L, lapatinib will be administered at a dose of 1250 mg per day (5 tablets) unless dose level -1 is required (1000 mg per day).

Patients on Arm D should take the lapatinib tablets either at least 1 hour before or 1 hour after their evening meal at approximately the same time each day, commencing on day 1 of cycle 1 of mECX. Lapatinib must not be taken with grapefruit or grapefruit juice, and no grapefruit or its juice should be consumed during the study.

Patients who vomit following a dose of lapatinib should not take further lapatinib until the following day when the next dose is scheduled. Persistent vomiting should be reported to the study team and managed as soon as possible.

8.4.4. Problems Administering Capecitabine or lapatinib

For patients who have difficulty swallowing capecitabine, the tablets can be dissolved in water. Place capecitabine tablets in approximately 200ml of lukewarm water. By agitating the tablets for approximately 15 minutes, the tablets should dissolve. The tablets should be dissolved immediately before use and the solution swallowed immediately as there is no stability data for any form of suspension. The solution may also be administered through a naso-gastric or other enteral feeding tube. The solution has a very bitter taste and a fruit juice can be added to make the solution more palatable, but capecitabine should not be mixed with grapefruit juice.
If a patient vomits after taking a dose of capecitabine, that dose should not be taken again.
Similarly, for patients on Arm D e.g. receiving lapatinib who are unable to swallow tablets or have a nasogastric or gastrostomy tube, lapatinib must not be crushed but

may be administered as dispersion in water. This should not be mixed with grapefruit juice.

8.4.5. Intervals between peri-operative treatment and surgery

Patients will have a 5-6 week break between pre-operative chemotherapy and surgery (5 weeks from last administration of capecitabine and lapatinib in cycle 3). Post-operative treatment should commence 6-10 weeks after surgery. These intervals should be the same in all treatment arms and are necessary to minimize the potential for increased peri-operative risk that may be related to chemotherapy, bevacizumab or lapatinib. If at 10 weeks post surgery, the Investigator's assessment is that a patient remains unfit to commence chemotherapy, then this should be reported to the Trial Manager at the CTU. Patients on the experimental arm may be allowed to commence lapatinib alone, after discussion with the Chief Investigator, via the ST03 Trial Manager.

8.4.6. Lapatinib maintenance therapy

Lapatinib monotherapy should be commenced immediately after the patient completes cycle 6 of mECX+L. Maintenance lapatinib will be delivered at a dose of 1500mg/day in 21 days cycles for 6 cycles at all initial dose levels of mECX+L unless patients have required a further dose reduction to lapatinib due to toxicity. Patients that required a dose reduction to lapatinib due to toxicity should receive maintenance therapy starting at 1250mg/day. If no Grade 3/4 toxicity is experienced after 21 days then this dose may be escalated to 1500mg/day.

Dose reductions should be made according to Section 8.5.

8.5. Toxicities and dose modifications for lapatinib

8.5.1. Haematological toxicities

Lapatinib must be withheld for patients with grade 3-4 anaemia, neutropenia or thrombocytopenia, until the haemoglobin, neutrophil and/or platelet counts have recovered to ≥8g/dL, $\geq 1.0 \times 10^9$/L and $\geq 75 \times 10^9$/L respectively. Lapatinib can be resumed at full dose (as per starting dose level), but for patients experiencing more than one episode of grade 3-4 haematological toxicity, the dose should be reduced by 250mg/day from the starting dose level.

8.5.2. Non-haematological toxicities

Diarrhoea

In previous studies diarrhoea occurred in approximately 65 % of patients who received lapatinib in combination with capecitabine and in 64 % of patients who received lapatinib in combination with letrozole. Most cases of diarrhoea were grade 1 or 2 and did not result in discontinuation of treatment with lapatinib (81). As the incidence of Grade ≥3 diarrhoea is an important safety outcome measure in the feasibility study it is essential that diarrhoea is managed as specified in the protocol and the CTU informed if any Grade ≥3 occurs.

ST03 Recommendations:

Uncomplicated grade 1 diarrhoea: Managed with oral rehydration and loperamide as an initial 4mg dose, then 2mg after each subsequent loose motion. For symptoms persisting for 24 hours or more despite these measures, the patient should be reviewed and consideration given to stool culture and oral antibiotics such as ciprofloxacin.

Uncomplicated grade 2 diarrhoea: Stop lapatinib. Manage as for uncomplicated grade 1 diarrhoea above. Restart lapatinib once toxicity has resolved to ≤grade 1. Lapatinib should be restarted at 1000mg/day if patient commenced treatment on dose level 0/+1, or 750mg/day if patient commenced treatment dose level -1 (i.e. 250mg decreased dose from starting dose of lapatinib).

Grade 3-4 diarrhoea or complicated grade 1-2 diarrhoea (patients with additional moderate-severe nausea or vomiting, abdominal pain, bleeding, deterioration of performance status, dehydration, grade 3-4 neutropenia, fever or sepsis): Stop lapatinib. These patients should be immediately reviewed by the medical team and may require admission to hospital. Initial treatment with loperamide is recommended and octreotide may be added for severe dehydration and/or refractory diarrhoea.

For grade 3 diarrhoea restart lapatinib once diarrhoea has resolved to ≤grade 1. Lapatinib should be restarted at 1000mg/day if patient commenced treatment on dose level 0/+1, or 750mg/day if patient commenced treatment dose level -1 (i.e. 250mg decreased dose from starting dose of lapatinib).

Grade 4 diarrhoea at any time must result in permanent cessation of lapatinib.

Recurrence of ≥ Grade 2 diarrhoea: Further dose reduction to 750mg/day, if patient commenced treatment on dose level 0/+1, or 500mg/day if patient commenced treatment on dose level -1 should be made for more than one episode of diarrhoea (i.e. 500mg decreased dose from starting dose of lapatinib).

Maintenance therapy: If Grade ≥3 diarrhoea occurs during maintenance therapy lapatinib should initially be withheld and then restarted at a dose reduction of 250mg/day less than the initial maintenance starting dose once diarrhoea has resolved to ≤grade 1.
A further dose reduction by another 250mg/day (total 500mg/day less than maintenance starting dose) should be made for more than one episode of grade ≥3 diarrhoea during maintenance therapy.

Grade 4 diarrhoea at any time must result in permanent cessation of lapatinib.

Nausea and vomiting

Grade ≥2 nausea or vomiting: Stop lapatinib. Lapatinib must be withheld in patients with grade 2-3 nausea or vomiting and restarted at 1000mg/day if patient commenced treatment on dose level 0/+1, or 750mg/day if patient commenced treatment on dose level -1 once the nausea or vomiting has resolved to ≤grade 1.

Recurrence of grade ≥2 nausea or vomiting: Further dose reduction to 750mg/day if patient commenced treatment on dose level 0/+1, or 500mg/day if patient commenced treatment on dose level -1 should be made for more than one episode of grade ≥2 nausea or vomiting.

Maintenance therapy: If Grade ≥2 nausea or vomiting occurs during maintenance therapy lapatinib should initially be withheld until toxicity resolves to ≤grade 1 and then restarted at a dose reduction of 250mg/day less than the initial maintenance starting dose. A further dose reduction by another 250mg/day (total 500mg/day less than maintenance starting dose) should be made for more than one episode of grade ≥2 nausea or vomiting.

Grade 4 nausea or vomiting at any time must result in permanent cessation of lapatinib.

Skin toxicity

Lapatinib can cause a papulopustular (acneiform) rash usually affecting the face, arms and trunk, dry skin, pruritis, photosensitivity and hair and nail changes including paronychia. Rash occurred in approximately 28 % of patients who received lapatinib in combination with capecitabine and in 45 % of patients who received lapatinib in combination with letrozole (81). Rash was generally low grade and did not result in discontinuation of treatment with lapatinib. Regular use of emollients and sun protection during exposure is recommended for all patients receiving lapatinib.

Grade 1-2 reactions: Should be managed with topical steroids (e.g 1% hydrocortisone) +/- oral tetracyclines (e.g lymecycline 408 mg daily) without dose interruption.

Persistent grade 2 reactions: Grade 2 reactions which persist despite these measures should be managed with dose interruption for up to 14 days and reintroduction with a dose reduction to 1000mg/day if patient commenced treatment on dose level 0/+1, or 750mg/day if patient commenced treatment on dose level -1 once the toxicity has resolved to ≤grade 1.

Recurrent persistent grade 2 reactions: Further dose interruption and further dose reduction to 750mg/day if patient commenced treatment on dose level 0/+1, or 500mg/day if patient commenced treatment on dose level -1 should be made for recurrence of persistent grade 2 reactions.

Grade 3 reactions: Withhold lapatinib for up to a maximum of 14 days. Treatment with topical steroids, oral tetracylines (and a short course of oral steroids if >50% of the body surface area is affected is recommended.

Once toxicity has resolved to ≤grade 1, lapatinib should be recommenced but with dose reduction to 1000mg/day if patient commenced treatment on dose level 0/+1, or 750mg/day if patient commenced treatment on dose level -1.

Recurrent grade 3 reactions: Grade 3 reactions which occur on more one occasion should be managed medically as above and with another dose interruption and another dose reduction to 750mg/day if patient commenced treatment on dose level 0/+1, or 500mg/day if patient commenced treatment on dose level -1 reactions.

Maintenance therapy: If Grade 2 or 3 reaction occurs during maintenance therapy lapatinib should initially be withheld until the toxicity has resolved to ≤grade 1 and then restarted at a dose reduction of 250mg/day less than the initial maintenance starting dose. A further dose reduction by another 250mg/day (total 500mg/day less than maintenance starting dose) should be made for more than one episode of persistent grade 2 toxicity.

A 3rd recurrence of grade 3 or grade 4 toxicity should result in permanent cessation of lapatinib.

Referral to a dermatologist is recommended for grade 3-4 reactions.

Hand-foot syndrome, stomatitis or peripheral neuropathy

Treatment with lapatinib should be interrupted for patients with grade 2 or more hand foot syndrome, stomatitis or peripheral neuropathy, then restarted at full dose once the toxicity has resolved to grade 1 or less.

Renal toxicity

For patients whose creatinine clearance falls to below 40mls/minute, lapatinib should be discontinued.

Hepatotoxicity:

Hepatoxicity has been observed with lapatinib therapy. The hepatotoxicity may be severe and deaths have been reported. Hepatotoxicity may occur days to several months after initiation of treatment however, often seems to be the result of a prolonged exposure to the drug of three months or longer. The majority of these cases were reversible and most patients experienced a decline in liver enzymes with drug withdrawal.

ST03 recommendations:

Patients with AST/ALT >3 X ULN should be managed according to the algorithm in figure B5:

Criteria for discontinuation of lapatinib:

Lapatinib dosing should be **permanently discontinued** if changes in liver function are severe (AST/ALT >3 × ULN and total bilirubin >2.0 × ULN (>35% direct; bilirubin fractionation required) and patients should not be retreated. This is a **notable event** for the study.

Please Note: Bilirubin fractionation should be performed if testing is available. If testing is unavailable and a subject meets the criterion of total bilirubin >2.0 × ULN, then the event should still be reported as a **notable event** and actions taken as described in figure B5.

The notable event initial or follow-up report should include the following information if applicable:

- the appearance or worsening of clinical symptoms of hepatitis, or hypersensitivity, fatigue, decreased appetite, nausea, vomiting, abdominal pain, jaundice, fever, or rash.
- use of concomitant medications, acetaminophen, herbal remedies, other over the counter medications, or putative hepato-toxins
- alcohol use

Criteria for interuption to lapatinib:

Patients with AST/ALT >3 × ULN **but** total bilirubin ≤2 × ULN **and** ALT >8 x ULN **or** ALT>5 x ULN persisting for ≥ 2 weeks, **or** with signs or symptoms of hepatitis or hypersensitivity (the appearance or worsening of fatigue, nausea, vomiting, right upper quadrant pain or tenderness, fever, rash, or eosinophilia), should:

- should have treatment with lapatinib interrupted for 2 weeks
- repeat LFTs in two weeks
- re-challenge may be considered if the transaminitis has improved but only after discussion with the Chief Investigator via the ST03 trial manager.
- LFTs should be monitored at a minimum of every two weeks until normalised, and then should continue to be monitored as per protocol schedule.

Continuation of lapatinib

Patients with AST/ALT >3 × ULN **but** <5 × ULN **and** total bilirubin ≤2 × ULN, without signs or symptoms of hepatitis or hypersensitivity, **and** who can be monitored weekly should:

- continue lapatinib;
- but should have weekly LFT testing until the transaminits resolves. If the derangement persists for 4 weeks or more, lapatinib should be discontinued.
- if at any time the subject meets any of the above lapatinib stopping criteria, then proceed as described above;

Figure 8: Hepatotoxicity Algorithm

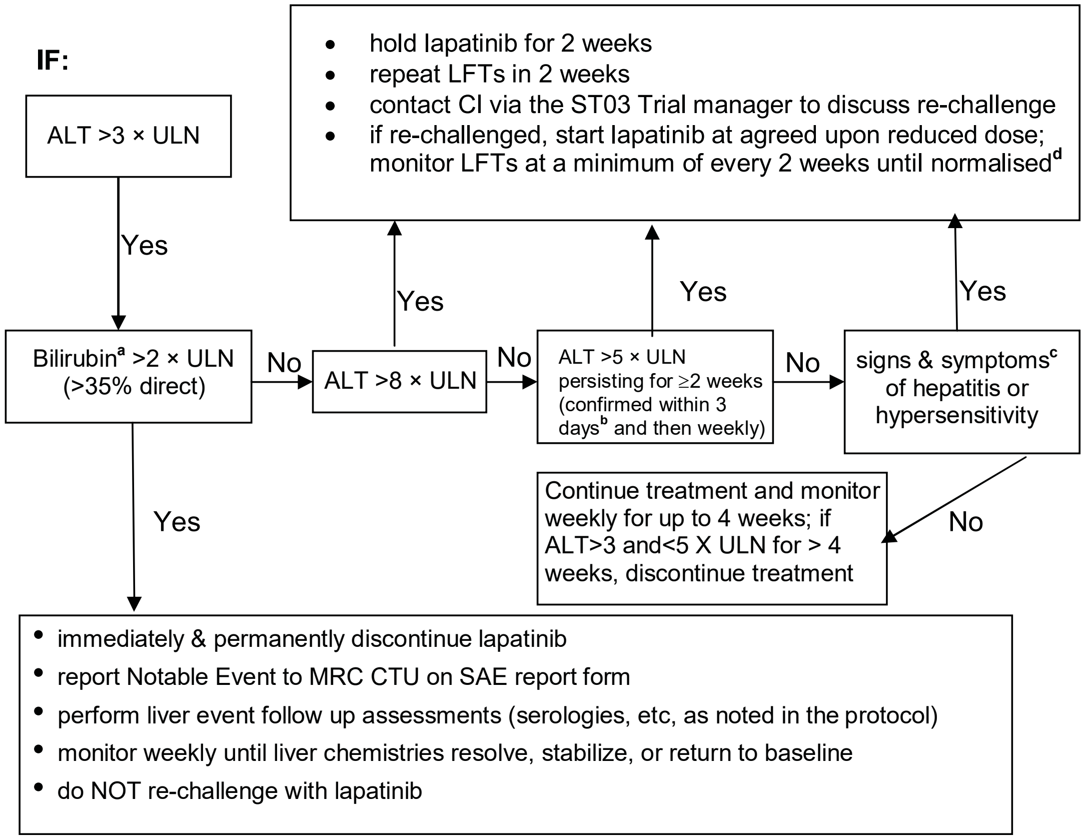

Notes:

a. Bilirubin fractionation should be performed if testing is available. If testing is unavailable and a subject meets the criterion of total bilirubin >2.0 × ULN, then the event should still be reported as a Notable Event and actions taken as described.

b. Retest within 3 days from the first occurrence and then weekly to determine if ALT elevation persists

c. The appearance or worsening of fatigue, nausea, vomiting, right upper quadrant pain or tenderness, fever, rash, or eosinophilia

d. Once LFTs normalise, then continue to monitor as per the protocol schedule.

<u>Investigations for patients with ALT/AST >3 x ULN and total bilirubin >2 x ULN:</u>

A specialist or hepatology consultation is recommended;

The following investigations should be considered:

1. Viral hepatitis serology for Hepatitis A IgM Ab, Hepatitis B sAg and Hepatitis B Core Ab (IgM); Hepatitis C RNA; Cytomegalovirus (CMV) IgM Ab; Epstein-Barr viral (EBV) capsid Ag IgM Ab (or if unavailable, obtain heterophile antibody or monospot testing); Hepatitis E IgM Ab (if the patient usually resides or has travelled outside UK in past 3 months);
2. Serum creatine phosphokinase (CPK) and lactate dehydrogenase (LDH);
3. FBC with differential to assess eosinophilia;

Cardiotoxicity

Patients with symptoms consistent with heart failure should undergo physical examination, ECG, cardiac troponin measurement, chest x-ray and ECHO/MUGA as soon as possible. Those with left ventricular impairment (LVEF <50% for ECHO or <LLN for MUGA) should be started on an ACE-inhibitor unless contraindicated and referred to a cardiologist. **Lapatinib and epirubicin should be discontinued permanently for these patients.**

Patients randomised into lapatinib comparison (Arm C: sECX or Arm D: mECX+L) are required to have at least two additional cardiac assessments, one before surgery and the other at the end of post-operative chemotherapy.

Lapatinib and epirubicin should be **permanently discontinued** if the patient's LVEF falls by ≥10% to <50% for ECHO (or <LLN for MUGA) from the previous cardiac assessment. An ACE inhibitor should be commenced unless contraindicated and the patient should undergo reassessment with physical examination, ECG, cardiac troponin measurement and chest x-ray. The patient should be reassessed including repeat ECHO/MUGA 4-6 weeks later. If they have developed symptoms consistent with heart failure or there is no improvement in LVEF the patient should be referred to a cardiologist.

Lapatinib should be withheld if the patient's LVEF falls by <10% to <50% for ECHO (or <LLN for MUGA) from the previous cardiac assessment. An ACE inhibitor should be commenced unless contraindicated and a further cardiac assessment should be taken 4-6 weeks later. If there is no improvement in LVEF or LVEF has decreased the patient should be referred to a cardiologist and lapatinib and epirubicin should be permanently discontinued. If the LVEF recovers to >50% (or >LLN for MUGA) then lapatinib and epirubicin may be continued.

See sections 9.2 and 9.3 for further information on the requirement for pre-surgery and post-operative chemotherapy cardiac assessments within the lapatinib comparison and Sections 9.2.2 and 9.3.2 for instruction on the management of reduction in LVEF.

Interstitial lung disease (Pneumonitis)

Interstitial lung disease has been reported in patients treated with EGFR inhibitors. Lapatinib should be discontinued in patients that develop clinical or radiological evidence of interstitial lung disease (any signs or symptoms of pneumonitis that are ≥Grade 3, defined as radiographic changes and requiring oxygen).

8.5.3 Specific toxicities requiring discontinuation of lapatinib

Any patient who develops any one of the following toxicities attributable to lapatinib must **not** receive further lapatinib:

- Grade 4 diarrhoea, nausea, vomiting or skin rash (for dose reductions for < grade 4 toxicity please see Section 8.5.2)
- Any signs or symptoms of pneumonitis that are ≥Grade 3 (defined as radiographic changes and requiring oxygen)
- Grade 3/4 congestive heart failure or left ventricular systolic or diastolic dysfunction (EF <40% of symptomatic heart failure, or a ≥20% decrease in LVEF to below LLN)
- AST/ALT >3x ULN and bilirubin >2x ULN (>35% direct bilirubin)

The events above require expedited reporting and discontinuation of lapatinib, please see Section 17.5 for more details as to the requirements for reporting notable events within the lapatinib comparison.

If a patient develops a second episode of diarrhoea, nausea, vomiting or skin rash as a grade 3 toxicity then the PI should not re-start lapatinib without first discussing this with the ST03 Trial Manager who will discuss the patient's situation with the Chief Investigator.

9. Assessments and Procedures within the Lapatinib Comparison

All assessments and trial procedures must be performed in compliance with the most up to date version of the protocol. Summaries of the timing of assessments and CRF data return are given in Tables 9.1, 9.2 and 9.3.

For details on the pre-randomisation assessments, see Section 6.4 Pre-Randomisation Investigation and Screening Procedures.

9.1 Assessments Required Prior to Each Cycle of Treatment with sECX or mECX+L

Toxicity Assessment (Day 1, or up to 3 working days before)
Patients will be assessed for the worst toxicity experienced (according to CTCAE v3.0 see Appendix G) in the preceding cycle and for suitability to proceed with the next cycle of treatment.

Cardiac Assessment
In addition to baseline cardiac assessment, patients should have a repeat ECG performed prior to each cycle of treatment. Any new abnormalities detected on ECG which may be potentially clinically significant should be investigated further before proceeding with treatment. This may include 24-hour Holter monitoring in patients with new onset rhythm abnormalities.

Haematology and Serum Biochemistry (FBC and Nadir Blood Count)
FBC should be performed on first day of each cycle or up to 3 days before. Coagulation profile will not be repeated as a routine unless clinically indicated.

All patients must have a nadir blood count during cycle 1 treatment (at approximately day 10 [± 2 days] of treatment) as described in Section 8.1.1.

Renal Function Assessment
An estimated creatinine clearance should be performed on the first day of each cycle or up to 3 days before, if this is <60ml/min Section 8.3.6 provides instructions for dose reductions in response to low creatinine clearances **(see Appendix I: Cockcroft and Gault Formula).**

During post-operative treatment and lapatinib maintenance therapy, the same schedule of assessment as during pre-operative treatment applies.

9.2. Assessments Prior to Surgery within the Lapatinib Comparison

9.2.1. Pre-Surgery Cardiac Assessment

Patients randomised into the lapatinib comparison (Arms C: sECX and D: mECX+L) are required to have reassessment of their cardiac function prior to surgery. This should be performed between completion of pre-operative chemotherapy and surgery. The method of assessment, whether by ECHO or MUGA, must be the same as that which was used for baseline assessment, patients should have a left ventricular ejection fraction ≥50% for ECHO (or >LLN for MUGA). **If the LVEF is <50% for ECHO (or <LLN for MUGA) please see Section 9.2.2 for management of reduction in LVEF.**

The assessment should be carried out even if the patient has not completed protocol treatment and should be done at the approximate time that this would have taken place had the patient completed all chemotherapy cycles. This also applies to the post-

operative cardiac assessment for patients who do not undergo surgery or do not complete post-operative chemotherapy.

In addition to baseline cardiac assessment, patients should have a repeat ECG performed prior surgery.

9.2.2. Management of Reduction in LVEF measured prior to Surgery

LVEF is measured as a percentage. In this section, the changes in LVEF refer to reductions in absolute LVEF percentage points. For example, a reduction from a baseline LVEF of 65% to a LVEF of 45% is an absolute reduction in LVEF of 20%. Where it is not possible to determine an exact value of LVEF, a range should be reported and management should be based on the lower bound of this range.

If a reduction in LVEF is detected in the pre-operative scan, patients should be managed as follows:

- Patients with a reduction in LVEF to a value which remains ≥50% for ECHO (or ≥LLN for MUGA) can continue to receive all protocol treatment. For patients with a reduction in LVEF of ≥15% from baseline, an additional ECHO/MUGA should be performed approximately 4-6 weeks later before the patient starts their post-operative chemotherapy, to ensure that LVEF has not dropped to <50% for ECHO (or <LLN for MUGA).

- Patients with symptoms consistent with heart failure should undergo physical examination, ECG, cardiac troponin measurement, chest x-ray and ECHO/MUGA as soon as possible. Those with left ventricular impairment, LVEF <50% for ECHO (or <LLN for MUGA), should be started on an ACE-inhibitor unless contraindicated and referred to a cardiologist. **Lapatinib and epirubicin should be discontinued permanently for these patients.**

- Asymptomatic patients, in whom a ≥10% fall in LVEF from baseline to <50% for ECHO (or <LLN for MUGA) is noted, should undergo reassessment with physical examination, ECG, cardiac troponin measurement and chest x-ray. **Lapatinib and epirubicin should be permanently discontinued.** The patient should be started on an ACE-inhibitor (e.g. perindopril 2mg od) unless contraindicated. This should be titrated to the maximum tolerated dose in consultation with the patient's general practitioner. Patients should have their renal function and electrolytes checked prior to ACE-inhibitor initiation and 1 to 2 weeks after initiation or dose titration. Patients should be reassessed including repeat ECHO/MUGA 4-6 weeks later. If they have developed symptoms consistent with heart failure or there is no improvement in LVEF the patient should be referred to a cardiologist.

- Patients in whom a <10% fall in LVEF from baseline to <50% for ECHO (or <LLN for MUGA) is noted, should be started on an ACE-inhibitor unless contraindicted. Lapatinib and epirubicin should be withheld. A further cardiac assessment should be taken 4-6 weeks later, if there is no improvement in LVEF or LVEF has decreased the patient should be referred to a cardiologist and lapatinib and epirubicin should be permanently discontinued. If the LVEF is >50% or LLN then lapatinib and epirubicin may be continued within post-operative chemotherapy.

Please note: The anaesthetist and surgeon should be made aware of these abnormal findings in case there are implications to the safety of proceeding with surgery or the peri-operative risk.

Please refer to Appendix N: Algorithm for cardiac monitoring requirements for more detail.

9.2.3 Tumour Response Assessment

Following completion of pre-operative treatment the patients should be assessed for response, within 2 weeks of completing pre-operative treatment. This assessment should be carried out regardless of whether the patient continues on protocol treatment. If the patient stops treatment early then a Tumour Assessment Form (ST03/4-HER) should also be completed at the time treatment stops. This assessment measures tumour response using the RECIST Version 1.0 guidelines (see Appendix F).

This tumour assessment should include:

- Spiral/multi-slice CT, with oral contrast or water including chest, abdomen (pelvis optional). Maximum slice width 5mm. IV contrast/venous phase.

- Other staging investigations, such as laparoscopy, EUS or PET scanning, should only be performed if clinically indicated, and according to local practice.

Patients should also be assessed for fitness for surgery and anaesthetic risk according to local practice.

9.3. Assessments after Completion of Post-Operative Chemotherapy within the Lapatinib Comparison

9.3.1. Post-Chemotherapy Cardiac Assessment

Patients randomised into the lapatinib comparison (Arms C: sECX and D: mECX+L) are required to have a further cardiac assessment after completion of post-operative chemotherapy treatment (i.e. after the last dose of capecitabine prior to lapatinib maintenance treatment). The method of assessment, whether by ECHO or MUGA, must be the same as that which was used for the baseline and pre-surgery assessment, patients should have a left ventricular ejection fraction ≥50% for ECHO (or >LLN for MUGA). **If the LVEF is <50% for ECHO (or <LLN for MUGA) please see Section 9.3.2 below for management of reduction in LVEF.**

The assessment should be carried out even if the patient has not completed protocol treatment and should be done at the approximate time that this would have taken place had the patient completed all chemotherapy cycles. This also applies for patients who do not undergo surgery.

9.3.2. Management of Reductions in LVEF measured after Post-Operative Chemotherapy

If a reduction in LVEF is detected after completion of post-operative chemotherapy, patients should be managed as follows:

- Patients with a reduction in LVEF to a value which remains ≥50% for ECHO (or ≥LLN for MUGA) can continue to receive all protocol treatment. For patients with a reduction in LVEF of ≥15% from their previous cardiac assessment, an additional ECHO/MUGA should be performed approximately 4-6 weeks later, to ensure that LVEF has not dropped to <50% for ECHO (or <LLN for MUGA).

- Patients with symptoms consistent with heart failure should undergo physical examination, ECG, cardiac troponin measurement, chest x-ray and ECHO/MUGA as soon as possible. Those with left ventricular impairment, LVEF <50% for

ECHO (or <LLN for MUGA), should be started on an ACE-inhibitor unless contraindicated and referred to a cardiologist. **Lapatinib should be discontinued permanently for these patients (i.e. lapatinib maintenance doses not given).**

- Asymptomatic patients, in whom a ≥10% fall in LVEF from their previous assessment to <50% for ECHO (or <LLN for MUGA) is noted, should undergo reassessment with physical examination, ECG, cardiac troponin measurement and chest x-ray. **Lapatinib maintenance doses should be permanently discontinued.** They should be started on an ACE-inhibitor (e.g. perindopril 2mg od) unless contraindicated. This should be titrated to the maximum tolerated dose in consultation with the patient's general practitioner. Patients should have their renal function and electrolytes checked prior to ACE-inhibitor initiation and 1 to 2 weeks after initiation or dose titration. Patients should be reassessed including repeat ECHO/MUGA 4-6 weeks later. If they have developed symptoms consistent with heart failure or there is no improvement in LVEF the patient should be referred to a cardiologist.

- Patients in whom a <10% fall in LVEF from their previous assessment to <50% for ECHO (or <LLN for MUGA) is noted, should be started on an ACE-inhibitor unless contraindicted. Lapatinib maintenance doses should be withheld. A further cardiac assessment should be taken 4-6 weeks later, if there is no improvement in LVEF or LVEF has decreased the patient should be referred to a cardiologist and lapatinib should be permanently discontinued. If the LVEF is >50% (or >LLN for ECHO) then lapatinib maintenance doses may be reintroduced.

Please refer to Appendix N: Algorithm for cardiac monitoring requirements for more detail.

Table 9.1 ST03 Trial Assessments for Lapatinib Comparison (Arms C and D)

Required Trial Assessments	Pre-Randomisation	Before each cycle (pre-operative & post-operative)	Pre-surgery	Before each cycle of Maintenance Lapatanib	Follow-up	Time Point
Clinical Examination (including BP)	Y	Y	Y	Y	Y	• Within 4 weeks prior to randomisation
CT scan-abdomen and chest (neck pelvis optional)	Y		Y		As clinically indicated	▪ **See note 1 at bottom of table**<br>▪ Within 2 weeks of completing last capecitabine tablet in pre-operative chemotherapy
EUS mandatory all lower oesophageal and OGJ tumours	Y					• Mandatory for all lower oesophageal and OGJ tumours within 4 weeks prior to randomisation<br>• According to local policy for gastric tumours
Laparoscopy for gastric, Siewert type II and III OGJ tumours only	Y					• Within 4 weeks prior to randomisation
MRI scans (**only if patient is participating in the MRI sub-study**)	Y (only once eligibility to ST03 confirmed)		Y			• Baseline can be pre or post randomisation provided eligibility has been confirmed and patient has given consent<br>• MRI within 2 weeks of completing last capecitabine tablet in pre-operative chemotherapy
FDG PET/CT scans (**only if patient is participating in the PET/CT sub-study**)	Y	Y between days 14-21 of cycle 1				• Baseline FDG PET/CT pre-randomisation as per standard care<br>• Post cycle 1 FDG PET/CT performed cycle 1 days 14-21 cycle 1
ECHO or MUGA<br>Ejection fraction should be ≥50% or above centre's LLN for MUGA if this is <50%	Y		Y	Y (at end of post-op chemotherapy)	If LVEF drops ≥15% or to <LLN further measurement is required 4-6 weeks after abnormal measurement	• Within 6 weeks of randomisation<br>• Between completion pre-op chemotherapy and surgery<br>• At the end of post-operative chemotherapy(or equivalent timepoints if patient does not complete protocol treatment)4-6 weeks following an abnormal measurement or drop of ≥ 15%
ECG	Y	Y	Y	Y		• Within 4 weeks of randomisation, before each cycle and prior to surgery
Spirometry: FEV1 should be ≥1.5 l (for OGJ/lower oesophageal tumours only)	Y					• Within 6 weeks of randomisation
FBC: WBC >3 x 10^9/l<br>Platelets ≥100 x 10^9/l<br>Neutrophils ≥1.5 x 10^9/l<br>Nadir Blood Count day 10 cycle 1	Y	Platelets ≥75 x 10^9/l<br>Neutrophils ≥1 x 10^9/l<br>Nadir Blood Count (day 10 cycle 1)		Y	If necessary	▪ 1 week before randomisation<br>▪ Day 1 of each cycle or up to 3 days before Nadir blood count at day 10 cycle 1

Required Trial Assessments	Pre-Randomisation	Before each cycle (pre-operative & post-operative)	Pre-surgery	Before each cycle of Maintenance Lapatanib	Follow-up	▪ Time Point
Glomerular Filtration Rate (measured or estimated)	Y	Y				▪ Within 1 week before randomisation<br>▪ Day 1 of each cycle or up to 3 days before
Coagulation Profile (INR/APTT normal range)	Y					▪ Within 1 week before randomisation<br>▪ Coagulation profile not to be repeated unless clinically indicated
U and E serum creatinine	Y	Y				Within 1 week before randomisation
Liver Function Tests ≤1.5xULN, x2.5 ALT/ AST, x3 ALP	Y	Y		Y		▪ 1 week before randomisation<br>▪ Day 1 of each cycle or up to 3 days before
PET/CT scan(For centres NOT participating in FDG PET/CT substudy)	optional					**See note 1 at bottom of table**
**Trans ST03 Blood sample collection**	Y					Before patient starts chemotherapy provided consent given
**Trans-ST03 collection of tumour samples**	Y		Y*			Before patient starts chemotherapy provided consent given<br>***Biopsy and Resection blocks to be sent to the RMH Laboratory after surgery provided consent given**
QoL Assessment	Y	End of Cycle 3 & 6	Y		Y	**See Table 9.3for QoL schedule**
Pregnancy Test	Y					-ve pregnancy test result, within 7 days of starting treatment, if appropriate

Note 1: The last tumour staging investigation, which may include EUS, CT, laparoscopy or PET/CT should normally be within 28 days prior to randomisation.

See Section 10 and Table 13.1 for Recommended Trial Assessments for Patients registered into the MRI and/or PET/CT sub-studies only.

9.4 Time Point for CRF Return Schedule

Table 9.2 Time Points for Completion of CRFs for lapatinib comparison Arms C and D

Time	Form(s)
**Pre-registration**	Registration Form (ST03/0-HER)
**Pre-Randomisation**	Randomisation Form (ST03/1-HER)<br>Pre Treatment Screening Form (ST03/2-HER)<br>Cardiac monitoring Form (ST03/14-HER)<br>Quality of Life (ST03/QL)
**Pre-treatment**	MRI Sub-study Local Reader Form for baseline MRI scan (ST03/16), if applicable
**Treatment Pre Surgery:**<br>Cycle 1, cycle 2, cycle 3<br>Cycle 3 Day 21	 Treatment Pre –surgery Form (ST03/3-HER)<br>Quality of Life (ST03/QL)
**Before Surgery**<br>End of Chemotherapy<br>Within 3 weeks before Surgery	 Tumour Assessment (ST03/4-HER)<br>MRI Sub-study Local Reader Form pre-surgery MRI scan (ST03/16), if applicable<br>Cardiac monitoring Form (ST03/14-HER)<br>Quality of Life (ST03/QL)
**After surgery**	Surgery Form (ST03/5-HER)<br>Pathology Form (ST03/6-HER)<br>MRI Sub-study Pathology Form (ST03/17), if applicable<br>Central Pathology Review Form (ST03/Path-HER)<br>Trans-ST03 Tissue and Blood Collection (Trans-ST03/HER)
**6 weeks Post Surgery Follow-Up**	Post-operative Report (ST03/7-HER)
**Treatment Post-surgery:**<br>Cycle 4, cycle 5, cycle 6<br><br>Cycle 6, Day 21	Post Surgery Treatment (ST03/8-HER)<br><br>Quality of Life (ST03/QL)
**9 weeks from the start of cycle 4**	End of Chemotherapy Form (ST03/9-HER)<br>Cardiac monitoring Form (ST03/14-HER)<br>Follow up Assessment (ST03/11-HER)
**Maintenance Lapatinib weeks 1, 4 and 7**<br>**18 weeks follow up assessment from the start of cycle 4**	Lapatinib Maintenance Treatment (ST03/10-HER)<br><br>Follow-Up Assessment (ST03/11-HER)<br>Quality of Life (ST03/QL)
**Maintenance Lapatinib weeks 10,13,16**<br>**27 weeks follow up assessment from the start of cycle 4**	Maintenance Lapatanib (ST03/10-HER)<br><br>Follow up Assessment (ST03/11-HER)
**12 Months Post Surgery**	Follow up Form (ST03/11-HER)<br>Quality of Life (ST03/QL)
**18 Months Post Surgery**	Follow up Form (ST03/11-HER)<br>Quality of Life (ST03/QL)
**24 Months Post Surgery**	Follow up Form (ST03/11-HER)<br>Quality of Life (ST03/QL)
**30 Months Post Surgery**	Follow up Form (ST03/11-HER)
**36 Months Post Surgery**	Follow up Form (ST03/11-HER)
**Annually thereafter**	Follow up Form (ST03/11-HER)
**Death**	Death Form (ST03/12-HER)
**Withdrawal From Trial**	Withdrawal Form (ST03/15-HER)

Table 9.3 Intended CRF completion schedule according to treatment received during the trial

CRF type (number)	All protocol treatment received	Scenario 1†	Scenario 2†
**Registration (0)**	Pre-registration	Pre-registration	Pre-registration
**Randomisation (1)** **Pre-chemo screening (2)**	Pre-randomisation	Pre-randomisation	Pre-randomisation
**Pre-surgery chemo (3)**	End cycles 1-3	End of each cycle received	End of each cycle received
**Tumour assessment (4)**	After end cycle 3	After last pre-op cycle received	After last pre-op cycle received
**Surgery (5)**	Immediately after surgery	Immediately after surgery	At time of decision not to perform surgery
**Pathology (6)**	Immediately after surgery	Immediately after surgery	N/A
**Post-operative (7)**	6 wks post-op	6 wks post-op	N/A
**Post-surgery chemo (8)**	End cycles 4-6	N/A	N/A
**End of chemo (9)**	After end cycle 6	At time of decision not to give post-op chemo	At time of decision not to perform surgery
**Lapatinib maintenance* (10)**	After each 21 day lapatinib cycle	N/A	N/A
**Follow-up (11)**	-	-	23 wks from start cycle 1
	9 wks from start cycle 4	32 wks from start cycle 1	32 wks from start cycle 1
	18 wks from start cycle 4	41 wks from start cycle 1	41 wks from start cycle 1
	27 wks from start cycle 4	50 wks from start cycle 1	50 wks from start cycle 1
	12 mths post-op	12 mths post-op	15 mths after start cycle 1
	18 mths post-op	18 mths post-op	21 mths after start cycle 1
	24 mths post-op	24 mths post-op	27 mths after start cycle 1
	30 mths post-op	30 mths post-op	33 mths after start cycle 1
	36 mths post-op	36 mths post-op	39 mths after start cycle 1
	Annually thereafter	Annually thereafter	Annually thereafter
**Death (12)**	As appropriate	As appropriate	As appropriate
**SAE (13)**	As appropriate	As appropriate	As appropriate
**QoL forms**	Pre-randomisation	Pre-randomisation	Pre-randomisation
	End cycle 3	9 weeks from start cycle 1	9 weeks from start cycle 1
	Within 3 weeks prior to surgery	Within 3 weeks prior to surgery	12 weeks from start cycle 1
	End cycle 6	32 wks from start cycle 1	32 wks from start cycle 1
	18 wks from start cycle 4	41 wks from start cycle 1	41 wks from start cycle 1
	12, 18 & 24 mths post-op	12, 18 & 24 mths post-op	15, 21 & 27 mths from start cycle 1

† **Scenario 1:** Patient receives some or all of the pre-operative chemotherapy and undergoes surgery but does not start post-operative chemotherapy

Scenario 2: Patient receives some or all of the pre-operative chemotherapy but does not undergo surgery (or subsequent post-operative chemotherapy)

* mECX+L patients only

In general, the dates given in the treatment schedule sent to you after each patient randomisation should be followed, even if patients do not complete protocol treatment. Please contact the trial office if you would like an updated treatment schedule sent to you for any patients that come off protocol treatment.

10. Selection of Patients for the MRI and/or PET/CT Substudies

10.1. Inclusion Criteria For Patients to be Registered into the MRI and/or PET-CT Sub-studies ONLY

In order to proceed to registration into the MRI and/or PET sub-studies (whether or not HER-2 testing has taken place) patients must meet all the inclusion criteria listed below:

a) **All patients must be scheduled to receive peri-operative sECX chemotherapy and surgery consistent with the ST03 Protocol.**

b) i) **MRI Sub-study:** Patients with histologically verified lower oesophageal, Siewert Type I or II OGJ adenocarcinoma, who have not received any treatment for their cancer.

ii) **PET/CT Sub-study:** Patients with histologically verified lower oesophageal, Siewert Type I, II or III OGJ or gastric adenocarcinoma, who have not received any treatment for their cancer.

The inclusion criteria described below are based on the TNM classification 6th edition for gastric cancer (gastric cancer and Siewert type III OGJ tumours), and oesophageal cancer (lower oesophageal cancer and Siewert type I/II OGJ tumours). Should you use TNM7 for staging of a patient's disease, please see Appendix M for guidance or speak to the ST03 team at the MRC CTU

c) **i) Siewert type III OGJ or Gastric Adenocarcinomas (using gastric cancer staging system)** **<u>PET/CT SUBSTUDY ONLY</u>**
Tumours should be Stage Ib (T1 N1, T2a/b N0), II, III or stage IV (T4 N1 or N2) with no evidence of distant metastases (M0) where the surgeon believes that an R0 resection can be achieved by excision of a contiguous structure.
Patients with linitis plastica should not be randomised.

ii) Lower Oesophageal or Siewert type I/II OGJ Adenocarcinomas (using oesophageal cancer staging system)
Tumours should be Stage II to Stage IVa (T1 N1, T2 N1, T3 N0-1, but not T2N0). T4 (N0 or N1) tumours are also eligible providing that they involve only the crura OR invade only the mediastinal pleura, where the surgeon believes that an R0 resection can be achieved by excision of a contiguous structure.
Patients with nodal disease affecting the origin of the left gastric and splenic artery or coeliac axis (hitherto staged as M1a) are also eligible.

d) All patients should have a CT of chest and abdomen (pelvis is optional) prior to study entry. Patients with gastric and Siewert type II and III OGJ adenocarcinomas should also have a laparoscopy prior to study entry. Endoscopic ultrasound (EUS) should be performed for all **lower oesophageal and OGJ** adenocarcinomas and according to local practice for other tumours.

Assessment of the suitability of patients to receive ECX chemotherapy should be performed as per standard local requirements. Recommended assessments are described in section 6.1, 6.2 and 6.4 of the protocol.

10.2 Exclusion Criteria For Patients to be Registered into the MRI and/or PET-CT Sub-studies Only

Assessment of the suitability of patients to receive ECX chemotherapy should be performed as per standard local requirements. Recommended assessments are described in section 6.1, 6.2 and 6.4 of the protocol.

10.2.1 Exclusion Criteria for the MRI Sub-Study only

Patients meeting the following exclusion criteria **must not** be registered into the MRI sub-study:

- Patients with a contraindication to MRI

10.2.2 Exclusion Criteria for the PET/CT Sub-Study only

Patients meeting the following exclusion criteria **must not** be registered into the PET/CT sub-study:

- Poorly controlled diabetes mellitus
- Non-FDG-avid and structurally non-measurable lesions on pre-treatment ^{18}F-FDG PET/CT and contrast enhanced CT respectively
- Unable to carry out PET/CT scan

10.2.3 Excluded concomitant medications

Any patient receiving the following concomitant medication should **not** be considered for the ST03 sub-studies:

a) Dipyridamole use should be avoided and concomitant administration of capecitabine and sorivudine (or sorivudine analogues e.g. brivudine) is contraindicated. Patients receiving phenytoin concomitantly with capecitabine should be regularly monitored for increase phenytoin plasma concentrations and associated symptoms.

10.2.4 Medications to be used with caution

- Patients receiving concomitant capecitabine and oral anticoagulants should have their anticoagulant response (INR or prothrombin time) monitored frequently in order to adjust the anticoagulant dose accordingly. Altered coagulation parameters and/or bleeding, including death, have been reported in patients taking capecitabine concomitantly with coumarin-derived anticoagulants such as warfarin and phenprocoumon. A pharmacokinetic interaction has been observed. The use of low molecular weight heparin instead of coumarin is advised but at the discretion of the Investigator.

10.3 Pre-Registration Investigations and Screening Procedures for Patients Registering for the MRI and/or PET/CT Sub-studies ONLY

10.3.1 Screening Assessments

Screening assessments should be performed as per local requirements for patients registering for the MRI and/or PET/CT sub-studies only. Recommended screening assessments are described in section 6.4 of the protocol.

The recommended assessments for the MRI and/or PET/CT sub-studies are summarised in Table 13.1 ST03 Recommended Trial Assessments (**see Section 13.**)

10.3.2 Tumour Staging Investigations

For patients who have consented to HER-2 testing a CT scan must be performed prior to registration for HER-2 testing to confirm no metastasis, however all further staging investigations can be performed whilst awaiting the HER-2 test result.

Tumour staging investigations should be performed as per local requirements for patients registering for the MRI and/or PET/CT sub-studies only. Recommended tumour staging investigations are described in section 6.4.2 of the protocol.

Please note that in order to be eligible for registration into the MRI and/or PET/CT sub-studies only patients must be staged as described in section 10.1.

10.3.4 Baseline MRI Scan for the MRI Sub-study

For those centres who are participating in the MRI Sub-study the baseline MRI scan must only be performed once the staging and screening investigations have confirmed eligibility listed in Section 10.1 and 10.2 for the MRI sub-study.

The site must ensure that informed consent is obtained from the patient prior to the baseline MRI scan being performed.

The baseline MRI scan should be performed as close to the staging investigations as possible once the patient has been confirmed to be eligible and has been registered into the MRI sub-study and **must** be performed prior to the patient commencing treatment.

10.3.5 Baseline PET/CT Scan for the PET/CT Sub-study

For those centres who are participating in the PET/CT sub-study the baseline PET/CT can be performed prior to consent and registration into the PET/CT sub-study as part of the standard clinical care.

The baseline PET/CT scan should be performed as close to the staging investigations as possible and **must** be performed prior to the patient commencing treatment.

Please note that the cycle 1 (day 14 – 21) FDG PET/CT can only be performed after the patient has been confirmed eligible for the PET/CT sub-study using the eligibility criteria listed in Sections 10.1 and 10.2 and consent for the sub-study has been obtained from the patient.

11. Registration into the MRI and/or PET/CT Sub-studies ONLY

In order to register patients for the MRI and/or PET/CT sub-studies only, the patient must meet the eligibility criteria detailed previously in Sections 10.1 and 10.2.

11.1 Written Informed Consent

Before a patient is registered into the MRI and/or PET/CT sub-studies, written informed consent for entry into the sub-study must be obtained.

Written confirmation that the patient has given their consent to participate in the sub-study should be recorded by a qualified, experienced nurse or a clinician according to local practice. Details of written informed consent for the MRI and PET/CT sub-studies are described below.

Patients who give their written informed consent to participate in the sub-studies can only be registered into the MRI and/or PET/CT sub-studies provided they fulfil all the eligibility criteria in Sections 10.1 and 10.2.

11.1.1 MRI Sub-study consent

Centres participating in the MRI sub-study must ensure that the patient is introduced to the **MRI sub-study PIS** prior to obtaining consent within the **MRI Sub-study Consent Form.** It will be explained to the patient that they will only receive the baseline MRI scan once they have been confirmed eligible for the MRI sub-study. The site must ensure that written informed consent has been obtained prior to the baseline MRI scan being performed.

The baseline MRI scan must only be performed once eligibility has been confirmed for the MRI sub-study. The baseline MRI scan should be performed as close to the staging investigations as possible once the patient has been confirmed to be eligible and has been registered into the MRI sub-study and **must** be performed prior to the patient commencing treatment.

11.1.2. PET/CT Sub-Study Consent

Centres participating in the PET/CT sub-study must ensure that the patients eligible for the PET/CT sub-study are introduced to the **PET/CT Sub-study PIS** prior to obtaining consent within the **PET/CT Sub-study Consent Form.** The baseline PET/CT may be performed prior to consent and registration into the PET/CT sub-study as part of the standard clinical care.

The baseline PET/CT scan should be performed as close to the staging investigations as possible and **must** be performed prior to the patient commencing treatment.

Please note that the cycle 1 (day 14 – 21) FDG PET/CT can only be performed after consent for the sub-study has been obtained from the patient.

11.1.3. Trans-ST03: Blood and Tissue Collection for Translational Research

Participation in the Trans-ST03 study is open to all patients registered for the MRI and/or PET/CT sub-studies and strongly encouraged. In order to collect tissue and blood samples for future translational research, patients are required to sign the **Trans-ST03 Consent Form.** Although most patients are expected to consent to participation in the translational study, the wishes of patients who do not want to be involved in the translational research part of the ST03 trial will be respected and they will be allowed to enter the clinical trial only.

20ml EDTA blood should be collected from the patients that consent to Trans-ST03 before commencing treatment.

For more information on the tissue and blood collection, please see Section 32 Pathology Research.

11.2 Procedure for Registration into the MRI and PET/CT sub-studies

11.2.1 Registration Following HER-2 Testing

The patient may not be eligible for the lapatinib comparison following HER-2 testing due to a HER-2 negative or inconclusive test result or the patient is found to be ineligible for randomisation into the lapatinib comparison or the patient does not consent to randomisation into the lapatinib comparison.

Provided **all** the eligibility criteria for registration into the MRI and/or PET/CT sub-studies in Sections 10.1 and 10.2 have been confirmed, the patient may register for the MRI and/or PET sub-studies only.

Once the patient has been confirmed eligible for registration into the MRI and/or PET/CT sub-studies, the following CRF should be completed before registering the patient for the sub-studies with the MRC CTU:

- MRI and PET/CT Sub-study Registration form (ST03/1-IMG)

When registering the patient for the MRI and/or PET/CT sub-studies **please ensure that the MRC CTU is aware that the patient has been registered previously for HER-2 testing** and state the trial number. You will be asked to confirm the patient initials and date of birth to verify that the correct patient is being registered. The trial number will remain the same and the patient will be registered into the MRI and/or PET/CT sub-studies as applicable.

11.2.2 Registration without HER-2 testing

Patients in whom HER-2 testing is not possible (i.e. patient did not consent to be tested) may still be registered into the MRI and/or PET/CT sub-studies providing they meet the eligibility criteria in Sections 10.1 and 10.2.

Once the eligibility criteria have been confirmed, the following CRF should be completed before phoning to register the patient into the MRI and/or PET/CT sub-studies as questions will be asked from this form:

- MRI and PET/CT Sub-study Registration form (ST03/1-IMG)

To register a patient telephone the MRC CTU:

+44 (0)207 670 4777 (9am - 5pm, Mon-Fri)

12. Treatment of Patients Registered to MRI and/or PET/CT Sub-studies Only

For guidance on treatment and dose reductions for patients registered into the MRI and/or PET/CT sub-studies only it is recommended that administration of sECX is as described in Section 8. Please refer to Sections 8.1 – 8.3 for further information.

13. Assessments and Procedures within the MRI and/or PET/CT Sub-studies Only

All assessments and trial procedures for the MRI and PET/CT sub-studies must be performed in compliance with the most up to date version of the protocol. Summaries of the timing of mandatory and recommended assessments and CRF data return are given in Tables 13.1 and 13.2.

For details on the pre-registration assessments, please see section 10.3.

13.1 Baseline MRI and PET/CT Scans Prior to Commencing Treatment

13.1.1 Baseline MRI Scan

For those centres who are participating in the MRI Sub-study the baseline MRI scan must only be performed once the staging and screening investigations have confirmed eligibility listed in Section 10.1 and 10.2 for the MRI sub-study.

The site must ensure that informed consent is obtained from the patient prior to the baseline MRI scan being performed.

The baseline MRI scan should be performed as close to the staging investigations as possible once the patient has been confirmed to be eligible and has been registered into the MRI sub-study and **must** be performed prior to the patient commencing treatment.

13.1.2 Baseline PET/CT Scan

For those centres who are participating in the PET/CT sub-study the baseline PET/CT can be performed prior to consent and registration into the PET/CT sub-study as part of the standard clinical care.

The baseline PET/CT scan should be performed as close to the staging investigations as possible and **must** be performed prior to the patient commencing treatment.

13.2 Assessments Prior to Each Cycle of Treatment

A**ssessments should be performed as per local requirements for patients registering for the MRI and/or PET/CT sub-studies only. Recommended screening assessments are described** below. **For information on recommended dose modifications please see section 8.**

Toxicity Assessment (Day 1, or up to 3 working days before)
Patients will be assessed for the worst toxicity experienced (according to CTCAE v3.0 see Appendix G) in the preceding cycle and for suitability to proceed with the next cycle of treatment.

Haematology and Serum Biochemistry (FBC and Nadir Blood Count)
FBC should be performed on first day of each cycle or up to 3 days before. Coagulation profile will not be repeated as a routine unless clinically indicated.

It is recommended that all patients have a nadir blood count during cycle 1 treatment (at approximately day 10 [± 2 days] of treatment) as described in Section 8.1.1. These results do not need to be sent to MRC CTU for patients registered to the MRI and/or PET/CT sub-studies only, but it is recommended thatpatient who develops grade 3 – 4 neutropenia should be treated as per instructions in Section 8.1.1.

Renal Function Assessment
An estimated creatinine clearance should be performed on the first day of each cycle or up to 3 days before, if this is <60ml/min Section 8.3.6 provides instructions for dose reductions in response to low creatinine clearances **(see Appendix I: Cockcroft and Gault Formula).**

During post-operative treatment, the same schedule of assessment as during pre-operative treatment applies.

Cardiac Assessments
Patients registered for the sub-studies only do not require additional cardiac assessments however it is recommended that all patients who develop symptoms consistent with heart failure are treated as per the instructions in sections 8.1.12 and 8.3.4.

13.3 Post Cycle 1 PET/CT Scan

The post cycle 1 PET/CT can only be performed after consent for the PET/CT sub-study has been obtained from the patient and should be performed after 1 cycle of chemotherapy at day 14 to 21.

13.4 Assessments Prior to Surgery

13.4.1 Pre-surgery MRI Scan
The pre-surgery MRI scan should be performed within 2 weeks of completing pre-operative chemotherapy (i.e. last dose of capecitabine) in order to restage the tumour prior to surgery.

13.4.2 Tumour Response Assessment
Following completion of pre-operative treatment the patients should be assessed for response, within 2 weeks of completing pre-operative treatment. This assessment should be carried out regardless of whether the patient continues on protocol treatment. If the patient stops treatment early then a Tumour Assessment Form (ST03/4-HER) should also be completed at the time treatment stops. This assessment measures tumour response using the RECIST Version 1.0 guidelines (see Appendix F).

This tumour assessment should include:

- Spiral/multi-slice CT, with oral contrast or water including chest, abdomen (pelvis optional). Maximum slice width 5mm. IV contrast/venous phase.

- Other staging investigations, such as laparoscopy, EUS or PET scanning, should only be performed if clinically indicated, and according to local practice.

Patients should also be assessed for fitness for surgery and anaesthetic risk according to local practice.

For instruction on MRI sub-study procedures please go to section 14.

For instruction on PET/CT sub-study procedures please go to section 15.

TABLE 13.1 ST03 TRIAL ASSESSMENTS RECOMMENDED FOR PATIENTS REGISTERED INTO THE MRI AND/OR PET/CT SUB STUDIES ONLY

Recommended Trial Assessments	Pre-registration to the MRI/PET/CT sub-studies	Before each cycle (pre-operative & post-operative)	Pre-surgery	Follow-up	Time Point
Clinical Examination (including BP)	Y	Y	Y	Y	• Within 3 weeks prior to registration into the sub-studies
CT scan-abdomen and chest (neck pelvis optional)	Y		Y	As clinically indicated	▪ **See note 1**<br>▪ Within 2 weeks of completing last capecitabine tablet in pre-operative chemotherapy
EUS mandatory all lower oesophageal and GOJ tumours	Y				• Recommended for all lower oesophageal and OGJ tumours within 4 weeks prior to registration into the sub-studies<br>• And according to local policy for gastric tumours
Laparoscopy for gastric, Siewert type II and III OGJ tumours only	Y				• Within 4 weeks of registration into sub-studies
**MRI scans (only if patient is participating in the MRI sub-study)**	**Y**		**Y**		• **Baseline post registration provided consent obtained**<br>• **MRI within 2 weeks of completing last capecitabine tablet in pre-operative chemotherapy**
**FDG PET/CT scans (only if patient is participating in the PET/CT sub-study)**	**Y**	**Y between days 14-21 of cycle 1**			• **Baseline FDG PET/CT pre-registration as per standard clinical care**<br>• **Post cycle 1 FDG PET/CT performed cycle 1 days 14-21**
ECHO or MUGA<br>Ejection fraction should be ≥50% or above centre's LLN for MUGA if this is <50%	Y			I	• Within 6 weeks prior to registration into the sub-studies
ECG	Y				• Within 4 weeks prior to registration into the sub-studies
Spirometry: FEV1 should be ≥1.5 l (for OGJ/lower oesophageal tumours only)	Y				• Within 6 weeks prior to registration into the sub-studies
FBC: WBC >3 x 10^9/l<br>Platelets ≥100 x 10^9/l<br>Neutrophils ≥1.5 x 10^9/l<br>Nadir Blood Count day 10 cycle 1	Y	Platelets ≥75 x 10^9/l<br>Neutrophils ≥1 x 10^9/l<br>Nadir Blood Count (day 10 cycle 1)		If necessary	▪ Within 1 week before prior to registration into the sub-studies<br>▪ Day 1 of each cycle or up to 3 days before<br>▪ Nadir blood count at day 10 cycle 1

					recommended
Glomerular Filtration Rate (measured or estimated)	Y	Y			▪ Within 1 week before prior to registration into the sub-studies<br>▪ Day 1 of each cycle or up to 3 days before
Coagulation Profile (INR/APTT normal range)	Y				▪ Within 1 week before prior to registration into the sub-studies<br>▪ Coagulation profile not to be repeated unless clinically indicated
U and E serum creatinine	Y	Y			▪ Within 1 week before prior to registration into the sub-studies
Liver Function Tests ≤1.5xULN, x2.5 ALT/ AST, x3 ALP	Y	Y			▪ Within 1 week before prior to registration into the sub-studies<br>▪ Day 1 of each cycle or up to 3 days before
**Trans-ST03 Blood Sample Collection**					**Before patient starts chemotherapy provided consent given**
**Trans-ST03 collection of tumour samples**	**Y**		**Y***		**Before patient starts chemotherapy provided consent given**<br>**Biopsy and Resection blocks to be sent to the RMH Laboratory after surgery**
Pregnancy Test	Y				negative pregnancy test result, within 7 days of starting treatment, if appropriate

Note 1: The last tumour staging investigation, which may include EUS, CT, laparoscopy or PET/CT should normally be within 28 days prior to registration into the sub-studies

Please note asssements highlighted in bold are mandatory for patients participating in the relevant sub-studies (e.g. MRI scan for MRI sub-study).

Table 13.2 Time points for completion of CRFs (for patients registered into MRI and/or PET/CT sub-studies only)

Time	Form(s)
**Pre-Registration for HER-2 Testing**	Registration Form (ST03/0-HER) (if applicable)
**Pre-Registration for MRI and/or PET/CT sub-studies**	MRI and PET/CT sub-study Registration Form (ST03/1-IMG)
**Pre-Treatment**	MRI Sub-study Local Reader Form for baseline MRI scan (ST03/16), if applicable
**Before Surgery**<br>End of Chemotherapy<br>Within 3 weeks before Surgery	 Tumour Assessment (ST03/4-IMG)<br>MRI Sub-study Local Reader Form for pre-surgery MRI scan (ST03/16), if applicable
**After surgery**	Surgery Form (ST03/5-IMG)<br>Pathology Form (ST03/6-IMG)<br>MRI Sub-study Pathology Form (ST03/17)<br>Trans-ST03 Tissue and Blood Collection (Trans-ST03/IMG)<br>Central Pathology Review Form (ST03-Path/IMG) *(MRI sub-study Only)*
**6 weeks Post Surgery Follow-Up**	Post-operative Report (ST03/7-IMG)
**9 weeks from the start of cycle 4**	End of Chemotherapy Form (ST03/9-IMG) *(PET/CT sub-study Only)*<br>Follow up Assessment (ST03/11-IMG) *(PET/CT sub-study Only)*
**18 weeks follow up assessment from the start of cycle 4**	Follow-Up Assessment (ST03/11-IMG) *(PET/CT sub-study Only)*
**27 weeks follow up assessment from the start of cycle 4**	Follow up Assessment (ST03/11-IMG) *(PET/CT sub-study Only)*
**12 Months Post Surgery**	Follow up Form (ST03/11-IMG) *(PET/CT sub-study Only)*
**18 Months Post Surgery**	Follow up Form (ST03/11-IMG) *(PET/CT sub-study Only)*
**24 Months Post Surgery**	Follow up Form (ST03/11-IMG) *(PET/CT sub-study Only)*
**30 Months Post Surgery**	Follow up Form (ST03/11-IMG) *(PET/CT sub-study Only)*
**36 Months Post Surgery**	Follow up Form (ST03/11-IMG) *(PET/CT sub-study Only)*
**Annually thereafter**	Follow up Form (ST03/11-IMG) *(PET/CT sub-study Only)*
**Death**	Death Form (ST03/12-IMG)
**Withdrawal From Trial**	Withdrawal Form (ST03/15-IMG)

14. MRI Sub-study Procedures

14.1 Timing of the MRI Scans

14.1.1 The Baseline MRI Scan

The baseline MRI scan must only be performed once the staging and screening investigations listed in Sections 10.1 and 10.2 have confirmed eligibility for the MRI sub-study. The site must also ensure that informed consent is obtained from the patient prior to the baseline MRI scan being performed.

The baseline MRI scan should be performed as close to the ST03 staging investigations as possible once the patient has been confirmed to be eligible and has been registered into the MRI sub-study and **must** be performed prior to the patient commencing treatment.

14.1.2 The Pre-Surgery MRI Scan

The pre-surgery MRI scan should be performed within 2 weeks of completing pre-operative chemotherapy (i.e. last dose of capecitabine) in order to restage the tumour prior to surgery.

14.2 MRI Sub-study Trial Schema

Figure 14. MRI Sub-study Trial Schema

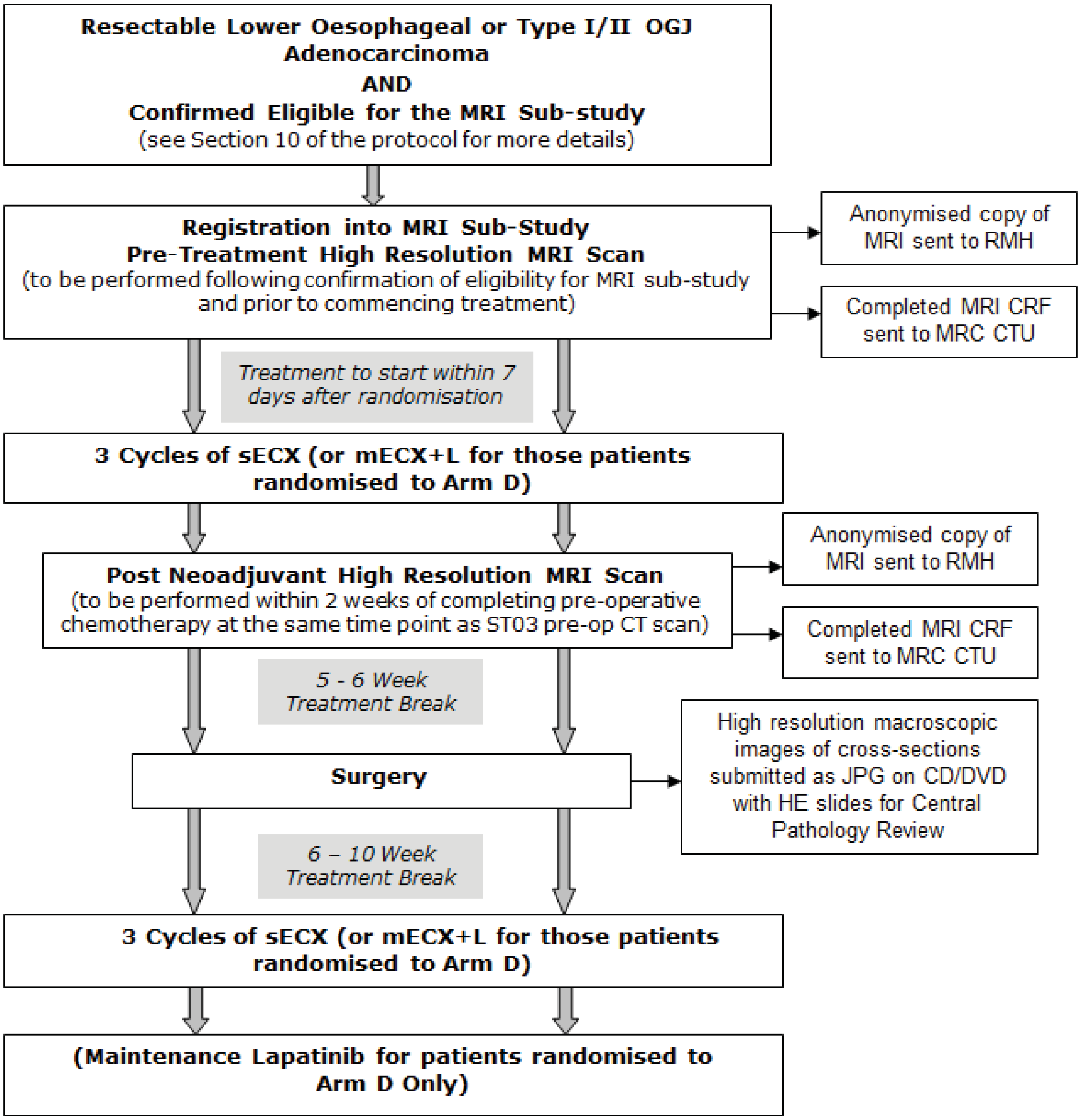

* RMH – Royal Marsden Hospital
Please send the requested copies of the MRI scans to the ST03 MRI Sub-study Chief Investigator, Dr Angela Riddell at the below address:

Dr Angela Riddell
Radiology Department
Royal Marsden Hospital
Downs Road
Sutton
Surrey

14.3 MRI Sequence

The sequence protocol for the MRI is given below. Recruiting centres are encouraged to adhere to the protocol when ever possible to generate a consistent dataset for analysis.

14.3.1 Patient Preparation

- Patients should be starved for 2 hours prior to the scan.
- Patients should be given 400mls of water to drink in the 20 minutes prior to the scan. With a further 50mls when on the table just prior to scanning.
- An antispasmodic agent such as Hyoscine butylbromide (Buscopan®) 20mg im) should be administered prior to the scan. If there are contra indications, such as Glaucoma or Atrial Fibrillation, Glucagon 1mg should be administered.
- Ensure there is good contact between the ECG tabs and the chest wall to achieve a good ECG trace.

14.3.2 Coil Selection

- Phased array surface coil. Ideally a 5 channel surface coil
- Coil position depends on tumour site. For oesophago-gastric tumours centre on the Xiphisternum. It is essential the coil position is optimum to achieve the maximum signal to noise ratio.

14.3.3 Sequences and Parameters

The technique uses T2-weighted sequences. The parameters below have been optimised for a Philips Intera® 1.5T magnet:

Parameter	Sag TSE T2W	Axial TSE T2W	Axial TSE T2W with cardiac gating	SSh Cor T2W
FOV (mm)	400	225	250	375
Matrix (mm2)	263x1024	224x256	296x512	144x256
Slice thickness /gap (mm)	3/0.4	3/0.3	4/0.4	5/1.0
TSE Factor	35	16	24	91
TR (ms)	3500	3620	3000	830
TE (ms)	120	80	90	80
NSA	4	6	8	1

For optimised parameters on alternative platforms (Siemens, GE) individual centres can contact the chief investigator Dr Angela Riddell.

Functional Data Collection

Diffusion Weighted Imaging (DWI) should also be collected. In order to standardise Apparent Diffusion Coefficient (ADC) calculations please adhere to the parameters shown below

**Plane of imaging**	Axial
**Type of Fat supression**	SPAIR if available
**Respiratory Gating**	Phase based navigator triggered or navigator triggered
**Echo Time**	Set Min TE (this is likely to be in the order of 60ms)
**Repetition Time**	Set Min TR with 2-3 concatenations
**Slice Thickness**	5mm with 1mm gap
**EPI Factor**	90-130
**Acquired Matrix**	128 - 140 matrix with partial fourier to 6/8
**No of phase encoding steps**	98
**Pixel bandwidth**	1770 - 3000Hz
**Flip angle**	90
**b values**	50 - 100 - 500
**Diffusion Directions**	Siemens: three scan trace; GE / Philips: tetrahedral
**Coverage**	Cover the Primary tumour (& regional lymph nodes if possible)

In order to standardise the data for the purposes of quantative analysis you should:

- Optimise the DWI sequence within the parameters provided. Once optimised use these without variation for all patients
- Ensure each patient undergos each scan on same scanner using an identical protocol
- Where possible ensure all patients are scanned on the same MRI scanner

14.3.4 Scanning technique

- Coil positioning is extremely important. The tumour must be in the centre of the field of view. Reposition after the survey if necessary.
- It is essential that the high resolution axial images are acquired perpendicular to the length of the tumour.

14.4 Data Collection & Storage

14.4.1 MRI CRF

The MRI data collected should be recorded on the MRI Sub-study Local Reader CRF (ST03/16). The CRF should be completed by a nominated Radiologist involved with the MRI sub-study at each centre. A copy of the MRI CRF should be retained by the local radiologist and a second copy should be collected by the Trial Research Nurse and returned to the ST03 Trial Manager at the MRC CTU with an anonymised copy of the formal MRI report.

14.4.2 MRI Scan Data Collection

In order to evaluate the of reproducibility of the MRI technique (by assessing interobserver variability) an anonymised copy of the each MRI scan, labelled with the patient's ST03 ID number and initials, should be sent to the MRI sub-study Chief Investigator, Dr Angela Riddell, at The Radiology Department, The Royal Marsden Hospital, Downs Road, Sutton, Surrey, SM2 5PT, for review & second reporting.

14.4.3 Pathology Data Collection from Resection

The resection specimen will be worked up as described in the guidance booklet for pathologists and high resolution macroscopic images of individual cross-sections should be taken at time of cut up (see guidance booklet). These images should be submitted, preferably as jpg files on a CD/DVD labelled with the ST03 patient ID number, initials, and pathology specimen number, together with the HE stained slides for the central pathology review (see Section 32.4 and the pathologist guidance booklet for more details).

If your centre is unable to fully comply with the pathology requirements as described above, you may still recruit patients to the MRI sub-study, please contact the ST03 trial manager for advice if this is the case.

14.5 Finance

14.5.1 ST03 MRI Sub-study Funding

The MRI scans are observational and will not alter the patient pathway. Separate funding has been secured for the scans via a Cancer Research UK Biomarkers & Imaging Discovery & Development (BIDD) grant.

14.5.2 Centre Reimbursement for MRI Scans

Centres will be reimbursed for the MRI scans (£300 per scan) upon receipt of the requested information for each patient as detailed below:

1. Baseline MRI Scan: - Anonymised copy of the MRI received by the CI at RMH
- Completed MRI CRF returned to ST03 Trial Manager

2. Pre-Surgery MRI Scan: - Anonymised copy of the MRI received by the CI at RMH
- Completed MRI CRF returned to ST03 Trial Manager
- High Resolution Macroscopic images received on CD/DVD at Leeds with the HE Slides for the pathology review

Reimbursement for the scans will be subject to receipt of all required information for each patient as listed above. Only patients confirmed eligible for ST03 and who meet the MRI sub-study inclusion criteria will receive reimbursement for MRI scans performed. Therefore it is important that the patient undergoes all the ST03 staging and screening investigations in order to confirm eligibility prior to the baseline MRI scan.

15. PET/CT Sub-study Procedures

15.1 PET/CT sub-study Method

Please see **Appendix S** for further details on the scanning, data collection and reporting procedures within the PET/CT sub-study.

(i) All patients entered in the PET/CT sub-study will have a pre-treatment FDG-PET/CT scan and a repeat FDG PET/CT scan after 1 cycle of chemotherapy at day 14 to 21.

(ii) Scans will be archived centrally. The first PET/CT (pre-treatment) will be reported locally by the local PET investigator as per routine. The local clinical investigators (treating clinicians) should however be blinded to the post-cycle 1 PETs' results, therefore NO clinical report should be issued by the local PET investigator. The second PET/CT will be analysed by the central PET readers only. (See **Appendix S**)

(iii) Patients will be treated according to the STO3 protocol.

(iv) Resection specimens will be assessed for tumour response.

(v) Metabolic response will be assessed in accordance with Objective Response Criteria (Appendix T).

(vi) Two central PET/CT Specialists (Dr S Chua, Prof G Cook) will review and report the pre- and post-treatment PET/CT blinded to the outcome of treatment. Differences in reporting will be resolved by consensus between two PET/CT Specialists or by a third PET/CT Specialist at another participating centre where agreement cannot be reached.

(vii) The PET/CT scans will be reported in batches and archived centrally. Analysis of data will be performed after completion of recruitment.

(viii) The result of the PET/CT scan will be correlated to treatment outcome, i.e. PFS, OS and pathological and clinical response.

Please note that PET emission acquisition **should be started 60 minutes after F-FDG administration and no longer than 70mins** after injection.

15.2 Timing of Scans

15.2.1. The Baseline PET/CT Scan

The baseline PET/CT can be performed prior to consent and registration into the PET/CT sub-study as part of standard clinical care. The baseline PET/CT should be performed as close to the staging investigations as possible and **must** be performed prior to the patient commencing treatment.

15.2.2. The Post Cycle 1 PET/CT Scan

The post cycle 1 PET/CT can **only** be performed after consent for the PET/CT sub-study has been obtained from the patient and should be performed after 1 cycle of chemotherapy at day 14 to 21.

15.3.PET/CT Sub-study Trial Schema

Figure 15. PET/CT Sub-study Trial Schema

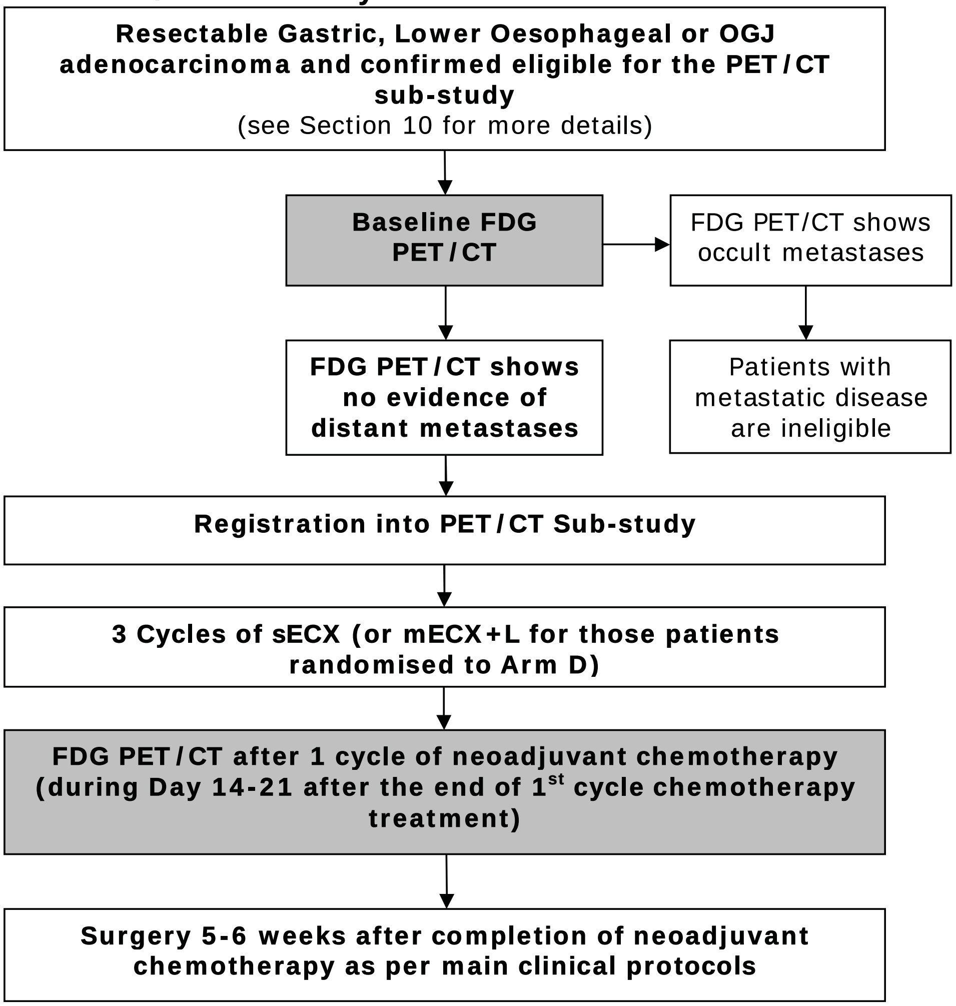

15.4 Data Collection & Storage

See **PET Sub-Study Appendix S** for instruction on the data collection, storage and reporting procedures.

15.5 Finance

The costs of the second PET/CT scan (after one cycle of neoadjuvant chemotherapy) are defined as Treatment Costs. As set out in Health Service Guidance 'HSG(97)32 Responsibilities for meeting Patient Care Costs associated with Research and Development in the NHS', the Treatment Costs of non-commercial R&D in the NHS, including Excess Treatment Costs, should be met through the normal arrangements for commissioning patient care i.e. by Primary Care Trusts performance managed by Strategic Health Authorities. A letter from the R&D Finance dept. of the Department of Health confirming the financial support for this trial can be obtained from the ST03 trial manager if required.

15.6 Publication Policy for the PET/CT Sub-study

The aim of the investigators is to author and publish the mature results of this study in a peer reviewed journal. All presentations and publications require authorisation from the Chief Investigator of the FDG PET/CT sub-study.

Authorship of any publications resulting from this study will be determined on the basis of the Uniform Requirement for Manuscripts Submitted to Biomedical Journals (International Committee of Medical Journal Editors, 2005), which states:

- Authorship credit should be based on (1) substantial contributions to conception and design, acquisition of data, or analysis and interpretation of data; (2) drafting the article or revising it critically for important intellectual content; (3) final approval of the version to be published. Authors should meet conditions 1, 2, and 3.
- When a large, multicentre group has conducted the work, the group should identify the individuals who accept direct responsibility for the manuscript. These individuals should fully meet the criteria for authorship defined above.
- Acquisition of funding, collection of data, or general supervision of the research group, alone, does not justify authorship.
- All persons designated as authors should qualify for authorship, and all those who qualify should be listed.
- Each author should have participated sufficiently in the work to take public responsibility for appropriate portions of the content.

16. Surgery – FOR ALL PATIENTS

Within 2 weeks of completing the pre-operative treatment (last capecitabine tablet) the tumour should be restaged to ensure that surgery is still appropriate [see Ssection 9.2.3 Tumour Response Assessments]. If your centre is participating in the MRI or FDG PET sub-studies please refer to section 14 and 15 respectively for more information.

Surgery should take place 5-6 weeks after the completion of pre-operative chemotherapy (last capecitabine or lapatinib tablet). For patients who received pre-operative bevacizumab prior to the October 2013 Urgent Safety Amendment, as bevacizumab is administered on day 1 of the last cycle of treatment and patients receive a further 3 weeks of capecitabine before pre-operative treatment is completed, at least 8 weeks should separate the last bevacizumab administration from surgery.

Post-operative treatment will not recommence till at least 6 weeks has elapsed from surgery, and will not occur unless patients are fully recovered. This is standard practice for the use of peri-operative chemotherapy in patients after gastrectomy or oesophago-gastrectomy regardless of bevacizumab/lapatinib administration.

16.1 Surgical Procedures

16.1.1 Gastric cancer including Siewert type III tumours

There are three acceptable types of resections that can be used for gastric and Siewert type III cancers at the surgeons discretion. These are:

- proximal gastrectomy (tumours of the cardia Siewert Type III)
- total gastrectomy (tumours of the cardia, fundus or body)
- distal subtotal gastrectomy (tumours of the antrum)

With any of the above operations surgeons may include the resection of any adjacent organ where the surgeon believes that by doing so, they will achieve a complete macroscopic tumour resection. This includes the extent of oesophageal resection for proximally located tumours.

The extent of lymphadenectomy is influenced by the site of the primary tumour and the type of gastric resection chosen. The principles to be followed are as indicated below:

Excision of Group 1 nodes (perigastric nodes:-stations 1-6)
All of these nodes should be removed when performing a total gastrectomy. With proximal gastrectomy, station 5, station 6 and some station 4 nodes along the greater curve will not be removed. Similarly with distal gastrectomy, station 2 and some station 4 nodes on the short gastric vessels will not be removed.

Excision of Group 2 nodes (left gastric, hepatic and splenic artery-stations 7-11)
With all types of resection, the station 7 nodes (left gastric) will be removed. An adequate number of nodes should be removed from the other stations to ensure that

the total number of lymph nodes excised exceeds fifteen. In the case of proximally located tumours, this must include the splenic artery territory nodes and in the case of distal tumours, this must include hepatic artery nodes. Formal D2 dissection can be used. Routine D3/4 dissections should not be carried out (82).

With total and distal gastrectomy, the greater omentum should be removed. The following are all acceptable methods of reconstruction and can be performed by hand suturing and/or stapling according to the surgeon's preference: oesophago-gastrostomy, oesophago-jejunostomy, (Roux-en-Y), oesophago-jejuno-gastrostomy (jejunal interposition), gastro-jejunostomy (Roux-en-Y or Billroth II), gastro-jejuno-duodenostomy (jejunal interposition).

Lymph nodes can be left attached to the main resection specimen. Should the surgeon wish to dissect the lymph nodes from the resected specimen, they should be submitted to the pathology department in separate specimen containers labelled either by their topographical location or by nodal station. When dissecting lymph nodes, this should be performed in such a way that it does not compromise the correct interpretation of any of the longitudinal or radial resection margins.

16.1.2 Siewert type II oesophagogastric junctional tumours

There are two acceptable resections that can be used for type II junctional tumours at the surgeon's discretion. These are:

- Extended gastrectomy
- Two phase oesophago-gastrectomy with a two-field lymphadenectomy (see section 5.1.3).

Extended Total Gastrectomy

The principles described above for total and distal gastrectomy should be applied for extended total gastrectomy. It is recommended that the hiatal dissection is approached either as in oesophago-gastrectomy described below (*) or via a transhiatal approach or via a left thoraco-abdominal approach depending on access to perform an adequate tumour and lower mediastinal lymph node dissection and construct the anastomosis. Frozen section examination of the oesophageal resection margin is recommended to ensure microscopic clearance.

16.1.3 Lower Oesophageal and Siewert Type I oesophagogastric junctional tumours

If your centre is participating in the MRI & FDG PET sub-studies, patients with lower oesophageal, Siewert type I and II cancers who are treated by an oesophago-gastrectomy will have been asked to participate in the MRI & FDG PET sub-studies. If this is the case, special attention needs to be given to the resection specimen by the surgeon (see section 5.1.4 for more details).

Oesophago-gastrectomy

Oesophago-gastrectomy should be performed via either a two phase right thoraco-abdominal approach or a left thoraco-abdominal approach with a two field lymphadenectomy (abdomen and thorax). It is recommended that the abdominal phase is carried out first. Stomach is the preferred organ for reconstruction and gastric mobilization should be based on the right gastro-epiploic arcade dividing the gastro-colic omentum and preserving the right gastric artery with division of the left gastric artery and vein. The gastro-splenic ligament should be divided as far laterally as possible with splenic preservation. Where the stomach is unavailable as a suitable conduit, colonic transposition should be performed according to surgeon's preference.

It is recommended to remove sufficient crural fibres and a cuff of diaphragm together with the pericardial fat pad and adjacent strips of parietal pleura to minimise the risk of a positive radial resection margin. The exact extent of this dissection will be influenced

by the results of the pre-operative staging, as well as intra-operative assessment. Placement of a feeding jejunostomy, intra-abdominal and intra-thoracic drains is an option at the surgeon's discretion.
The intra-abdominal lymph node dissection should be as described above for proximal gastrectomy and include group 1, 2, 3, 7, 8 and 11.

The chest can be opened through either a right or left thoracotomy. The mediastinal pleura overlying the oesophagus should be excised in continuity with the oesophagus. Para-oesophageal and diaphragmatic nodes (groups 108,110,111) should be removed in continuity with the lower oesophagus. Nodes at the tracheal bifurcation and along the right and left main bronchi to the pulmonary hilus (nodal groups 107, 109), should ideally also be removed en bloc.

16.1.4 Oesophago-gastrectomy resection specimens from patients participating in the MRI and/or FDG-PET sub-studies

As part of the MRI & FDG-PET sub-studies (see Sections 14 and 15 respectively) MRI & FDG-PET/CT imaging findings will be related to histopathological findings. One important point is therefore that the orientation of the resection specimen needs to be preserved throughout the process and the oesophageal tube/GOJ of the resection specimen cannot be opened longitudinally prior to fixation. However, if the local surgeon/pathologist wishes to sample fresh tissue from the tumour, the specimen can be opened along the distal (gastric) resection margin and tumour tissue can be sampled reaching into the oesophagus from inside leaving the wall intact. If the tumour is small, it may be possible to turn the oesophageal tube inside out for this purpose. The tube should be turned back before fixation. The specimen should be submitted to the pathology department fresh e.g. NOT in fixative to avoid specimen distortion.

As it can be very difficult for the pathologist to identify anterior/posterior and right/left side of the resection specimen, the surgeon will need to attach marking sutures to the specimen before submitting the specimen to the pathology laboratory. It is recommended to attach a long (10cm) suture onto the anterior surface of the oesophagus, a second long (10cm) suture onto the anterior surface of the stomach and a third short (5cm) suture onto the right side of the oesophagus. It is also recommended that the surgeon indicates on the histopathology request form that the patient is participating in the ST03 MRI substudy and that orientation sutures have been attached including details of their location.

Ideally, the specimen should be submitted with all its surrounding material (fat, pleura, lymph nodes, duct etc) attached to the pathology laboratory where it will be cross-sectioned after fixation to have MRI compatible slices.

However, if the surgeon wishes to dissect lymph nodes off the specimen, it is recommended, that the tissue surrounding the tumour will not be dissected but left attached to the specimen to preserve the radial resection margin around the tumour. Furthermore, if lymph nodes are dissected and if technically possible, peri-oesophageal nodes should be separated by their anatomical location and in addition labelled with reference to the oesophageal tube as anterior, posterior, right or left. This will allow to match the MRI information with the histopathological findings.

16.1.5 Minimally Invasive Surgery

Centres who wish to enter patients into the ST03 trial and perform the operation as a total minimally invasive procedure should contact the ST03 Trial Manager. An open chest procedure combined with a laparoscopic abdominal procedure is permitted for lower oesophageal/OGJ Type I and II tumours without review of previous cases as was permitted in the OEO5 trial.

In order to maintain surgical quality assurance, each surgeon/surgical team that wishes to perform totally minimally invasive procedure for ST03 patients will be asked to provide summary evidence of their previous 20 minimally invasive operations that are relevant to the conduct of ST03 (i.e. totally minimally invasive gastrectomy or oesophagogastrectomy) for review by the Trial Management Group. To achieve 20 oesophagectomy cases without withdrawing from recruiting to the trial, it would be permissible to include laparoscopic operations for squamous cell cancers and for high grade dysplasia, however; at least 10 of the 20 cases should be either T3 or N1. The following information should be supplied, dates of the operations, lymph node yields in both the abdomen and the mediastinum and post-operative complication rates.

16.2 Complications with surgery

Gastric and lower oesophageal surgery is associated with complications. These may occur during the operation itself, whilst recovering from surgery in hospital and there are complications that may occur after discharge and during the months following surgery.

16.2.1 Operative complications

During the operation it may be necessary to perform additional surgery because of damage to organs close to the stomach or because the blood supply to other organs is damaged during the dissection. Splenectomy may be necessary and although this does not usually lead to long-term problems after recovery from surgery it is necessary to remain on long term antibiotics.

Damage to the pancreas may occur during mobilisation of the stomach. This may lead to leakage of pancreatic enzymes and formation of an intrabdominal abscess or a fistula to the skin. Pancreatic collections/fistula may be small and lead to a delay in discharge, occasionally they are large and problematic requiring percutaneous drainage, prolonged courses of antibiotics and rarely re-operation.

16.2.2 In hospital complications

Failure of an anastomosis or staple line to heal may occur following a total or subtotal gastrectomy or oesphago-gastrectomy. Leakage of the oesophago-jejunal anastomosis, gastroenterostomy or jejenojejunostomy may occur. Leakage may also occur from the duodenal stump.

Leakage may lead to problems with sepsis requiring intravenous antibiotics, or it may cause an intra-abdominal collection and abscess requiring percutaneous or open drainage. An anastomotic leak may also require reoperation to adequately drain or repair the problem. If there is an anastomotic leak after surgery, it may result in multi-system organ failure and admission to an intensive care unit.

16.2.3 Small bowel obstruction

It is possible that small bowel obstruction may occur after surgery. This may be related to adhesions or to mechanical problems with the roux en y or the feeding jejunostomy.

16.2.4 General complications

Folllowing surgery, patients are at risk of general complications that may occur after any abdominal (+/- thoracic) procedure. These include chest infection (pneumonia), pulmonary embolic problems (deep vein thrombosis and pulmonary emboli), wound infections, cardiac dysfunction (arrthymia or ischaemia) or renal failure or urinary problem related to catheterisation.

16.2.5 Post operative mortality

Following any of the above complications there is a risk of death whilst in hospital. The estimated risk of in-hospital mortality after gastrectomy is about 1 in 20 in large cancer centres, although figures from a national audit suggest that it may be as much as 1 in 10.

16.2.6 Frequency of Complications

Patients should be monitored closely following surgery and may resume treatment with bevacizumab provided that there are no other contraindications.

Based on a review of cumulative data from the ST03 trial, the IDMC noted an increased risk of anastamotic leaks in a subset of patients receiving bevacizumab. Of the 255 oesophagectomies/gasto-oesophagectimes in the bevacizumab comparison the anastamotic leak rate was 9% in the sECX arm and 24% in the sECX+B arm. This resulted in an Urgent Safety Amendment which was implemented with immediate effect in October 2013 to amend the inclusion criteria to remove patients with lower oesophageal and OGJ adenocarcinomas. This Urgent Safety Amendment also mandated that patients with lower oesophageal, Siewert Type I, II or III OGJ adenocarcinomas who were already entered into the sECX+B arm (Arm B) must not receive any further pre-operative bevacizumab.

Other complications are less frequent although there is a spectrum of general cardio-respiratory, infective and thrombo-embolic complications.

In the MAGIC trial the overall rate of postoperative complications was 46%, but this covered both general and local complications. The overall operative mortality was 6% and this rate would be expected in STO3 and not exceeded. In addition in MAGIC the rate of curative resection was 72%, palliative resection 24% and inoperability 4%. The same rates would be expected in STO3.

17. Safety Reporting

ICH GCP requires both investigators and sponsors to follow specific procedures when reporting adverse events/reactions in clinical trials. These procedures are described in this section of the protocol.

SAEs occurring in patients registered into the MRI and/or PET/CT sub studies ONLY do not require reporting to the MRC CTU.

17.1 Definitions of Adverse Events/Reactions

The definitions from ICH GCP apply in this protocol. These definitions are given in Table 17.1:

TABLE 17.1 TERMS AND DEFINITIONS FOR ADVERSE EVENTS

Term	Definition
**Adverse Event (AE)**	Any untoward medical occurrence in a subject to whom a medicinal product has been administered including occurrences which are not necessarily caused by or related to that product.
**Adverse Reaction (AR)**	Any untoward and unintended response in a subject to an investigational medicinal product, which is related to any dose administered to that subject.
**Serious Adverse Event (SAE)**<br>**Serious Adverse Reaction (SAR)**	Any untoward medical occurrence or effect that at any dose:<br>• results in death<br>• is life-threatening*<br>• requires hospitalisation or prolongation of existing hospitalisation**<br>• results in persistent or significant disability or incapacity<br>• consists of a congenital anomaly or birth defect<br>• other important medical event(s)***
**Suspected Unexpected Serious Adverse Reaction (SUSAR)**	A SUSAR is a SAR that is classified as 'unexpected' i.e. a SAR, the reaction, the nature and severity of which is not consistent with the information about the medicinal product in question set out in the summary of product characteristics (SPC) or Investigator brochure (IB) for that product.

* The term 'life-threatening' in the definition of 'serious' refers to an event in which the patient was at risk of death at the time of the event; it does not refer to an event which hypothetically might have caused death if it were more severe.

** Hospitalisation is defined as an inpatient admission, regardless of length of stay, even if the hospitalisation is a precautionary measure for continued observation. Hospitalisations for a pre-existing condition, including elective procedures that have not worsened, do not constitute an SAE.

*** Other events that may not result in death, are not life threatening, or do not require hospitalisation may be considered a serious adverse experience when, based upon appropriate medical judgement, the event may jeopardise the patient and may require medical or surgical intervention to prevent one of the outcomes listed above (excluding new cancers or result of overdose).

17.2 Severity/grading of adverse events

Adverse events will be graded using the NCI Common Toxicity Criteria (CTCAE) Version 3.0, an abridged version is given in Appendix J. The complete CTCAE V3 can be found at http://ctep.cancer.gov/reporting/ctc.html. Previously reported (and, therefore, expected) reactions from Epirubicin, Cisplatin, Capecitabine are listed in **Appendix L**.

All patients experiencing a SAE or an adverse event should be followed up as outlined in the protocol. For patients who decide to withdraw from the trial treatment after an adverse event the procedure outlined in Section 8 should be followed.

17.3 SAE Events Excluded from Expedited Notification

The following events **should not be considered as SAEs** and are therefore excluded from expedited reporting. They do not need to be reported on a SAE form but should be recorded on the toxicity assessment section of the Treatment form.

(i) **Disease progression** or **death** as a result of disease progression (should be reported on a follow-up form (ST03/11) or death form (ST03/12))

(ii) **Elective hospitalisation** and surgery for treatment of **oesophagogastric cancer** or its complications

(iii) **Elective hospitalisations** to **simplify treatment** or procedures

(iv) **Elective hospitalisation** for **pre-existing conditions** that have not been exacerbated by trial treatment.

(v) **Nausea and Vomiting** caused by chemotherapy

(vi) **Diarrhoea caused by Capecitabine only for patients randomised to sECX+/-B comparison.** Diarrhoea meeting the criteria for seriousness or a notable event (i.e. Grade 3 or 4 diarrhoea, see section 6.5) in patients randomised to sECX Vs mECX+lapatinib should **always** be reported.

(vii) **Neutropenia**

(viii) SAEs occurring in patients who are registered ONLY in the MRI and/or PET/CT sub studies

17.4 ST03 Additional Notable Events that Require Expedited Reporting for Patients within the sECX+/-B Comparison (Arms A and B)

The following events are regarded as **notable events for the sECX+/-bevacizumab comparison** and must be reported within 1 working day on a Serious Adverse Event Reporting Form, even if they do not fulfil the definition of a SAE:

- A participant **confirmed pregnant** whilst receiving Bevacizumab treatment
- All new cases of **Grade 3 and 4 hypertension** which occur up to 18 months after commencing treatment
- All new cases of **Grade 3 and 4 proteinuria** which occur up to 18 months after commencing treatment.
- All cases of **post operative wound healing complications** or delayed wound healing which occur up to 18 months after commencing treatment
- All cases of **gastrointestinal perforations** which occur up to 18 months after commencing treatment
- All new cases of **Grade 3 and 4 arterial thromboembolic** events, including, transient ischaemic attack (TIA), cerebrovascular accident (CVA) and myocardial infarction occurring up to 18 months after commencing treatment.
- All new cases of **Grade 3 and 4 haemorrhagic events** occurring for up to 18 months after commencing treatment

- **Grade 4 venous thromboembolic events** (see section A4.2.5)

- All grades of **fistulae**

- **Grade 3 and 4 congestive heart failure or left ventricular dysfunction** (symptomatic heart failure, LVEF <40%)

17.5 ST03 Additional Notable Events that Require Expedited Reporting for Patients within the sECX Vs mECX + L Comparison (Arms C and D)

The following events are regarded as **notable events for the sECX Vs mECX+lapatinib comparison** and must be reported within 1 working day on a Serious Adverse Event Reporting Form, even if they do not fulfil the definition of a SAE:

- **Grade 3 or 4 Diarrhoea**

- A participant **confirmed pregnant** whilst receiving lapatinib treatment

- **Grade 3 and 4 congestive heart failure or left ventricular dysfunction** (symptomatic heart failure, LVEF <40% or a $\geq$20% reduction in LVEF to below LLN) occurring up to 18 months after commencing treatment

- Development **$\geq$Grade 3 pneumonitis** (defined as radiographic changes and requiring oxygen) within 18 months after commencing treatment

- **Severe liver dysfunction**; AST/ALT >3 X ULN and total bilirubin >2.0 X ULN (>35% direct; bilirubin fractionation required. Note: Bilirubin fractionation should be performed if testing is available. If testing is unavailable and a subject meets the criterion of total bilirubin >2.0 × ULN, then the event should still be reported as a **notable event**) within 18 months after commencing treatment.

- All new cases of **Grade 3 and 4 arterial thromboembolic** events, including, transient ischaemic attack (TIA), cerebrovascular accident (CVA) and myocardial infarction occurring up to 18 months after commencing treatment

- **Grade 4 venous thromboembolic events**

17.6 Thromboembolic events and ECX chemotherapy

Arterial and venous thromboembolic events (including myocardial infarctions, cerebrovascular accidents, transient ischemic attacks, peripheral arterial embolism with acute limb ischaemia, deep vein thromboses and pulmonary emboli) are **uncommon, but expected** events for patients with advanced oesophago-gastric cancer treated with the combination regimen, ECX. This was demonstrated in the REAL-2 trial, with a reported frequency of thromboembolic events of 13.3% in patients treated with ECX (83).

17.7 Investigators Assessment

Seriousness

When an AE/AR occurs the investigator responsible for the care of the patient must first assess whether the event is **serious** using the definitions given in Table 6.1. If the event is serious and not exempt from expedited reporting, or is one of the additional notable events then an SAE form must be completed and faxed to MRC CTU

Causality

The Investigator must assess the causality of all events/reactions in relation to the trial therapy using the definitions in Table 6.1. There are 5 causality categories: unrelated, unlikely, possible, probable and definitely related. If the causality assessment is unrelated or unlikely to be related then for reporting purposes the event will not be regarded as an adverse reaction to trial therapy. If the causality is assessed as either possible, probable or definitely related then for reporting purposes the event is classified as an adverse reaction. (see Table 17.2).

Expectedness

The Investigator must assess the expectedness of all serious adverse reactions from the lists of expected toxicities for each trial medication. A summary of these for ECX are given in **Appendix L: Summary of Product Characteristics**, the bevacizumab IB should be used for assessing expectedness for bevacizumab and the Summary of Product Characteristics found at the below link should be used for assessing expectedness for lapatinib:
http://www.medicines.org.uk/emc/medicine/20929/SPC/tyverb/

TABLE 17.2: DEFINITIONS OF CAUSALITY

Relationship	Description	Event Type
**Unrelated**	There is no evidence of any causal relationship	Adverse event
**Unlikely**	There is little evidence to suggest there is a causal relationship (e.g. the event did not occur within a reasonable time after administration of the trial medication). There is another reasonable explanation for the event (e.g. the patient's clinical condition, other concomitant treatment).	Adverse event
**Possible**	There is some evidence to suggest a causal relationship (e.g. because the event occurs within a reasonable time after administration of the trial medication). However, the influence of other factors may have contributed to the event (e.g. the patient's clinical condition, other concomitant treatments).	Adverse reaction
**Probable**	There is evidence to suggest a causal relationship and the influence of other factors is unlikely.	Adverse reaction
**Definitely**	There is clear evidence to suggest a causal relationship and other possible contributing factors can be ruled out.	Adverse reaction

17.8 SAE Notification Procedures By Investigator

Investigators must notify the MRC CTU of all SAEs occurring from the time of randomisation until 30 days after the last protocol treatment administration. SARs, SUSARs and notable events must be notified to the MRC CTU indefinitely (i.e. no matter when they occur after randomisation).

1. **The SAE form** must be completed by the Investigator (consultant or delegated other person named on the signature list and delegation of responsibilities log who is responsible for the patient's care). It is essential that the following data is provided on the initial report: **patient details, why event was serious, SAE name**, **grade, causality** and **expectedness of the event**. In the absence of the responsible investigator the form should be completed and signed by a member of

the site trial team. The responsible investigator should subsequently check the SAE form, make changes as appropriate, sign and then re-fax to the MRC CTU as soon as possible. The initial report shall be followed by detailed, written reports as appropriate.

The initial SAE report must be faxed to the MRC CTU within one working day of the investigator's knowledge of the event. **Fax Number: + 44 (0) 20 7670 4818**

2. **Follow-up SAE:** Patients must be followed-up until clinical recovery is complete and laboratory results have returned to normal or baseline, or until the event has stabilised. Follow-up should continue after completion of protocol treatment if necessary. Follow-up information should be noted on a further SAE form by completing the box marked 'type of report' and faxed to the MRC CTU as information becomes available. Extra, annotated information and/or copies of test results may be provided separately. The patient **must** be identified by trial number, date of birth and initials only. The patient's name **should not** be used on any correspondence.

3. **Clarification Procedures:** Investigators may be asked to clarify submitted SAE reports. The initial request for clarification will be sent to the responsible clinician. The centre is asked to respond to the query within 2 working days.

4. Staff at the centre must **notify** their local research ethics committee (LREC) of the event (as per the centres standard local procedure).

A flowchart in **Figure 17** may help to explain the notification procedures. Any questions concerning this process should be directed to the ST03 Trial Manager.

17.9 MRC CTU Responsibilities

Medically qualified staff at the MRC CTU and the Chief Investigator (or a delegate) will evaluate all SAEs received for seriousness, expectedness and causality. Investigator reports of suspected SARs will be reviewed immediately and those that are SUSARs identified and reported to regulatory authorities. The causality assessment given by the local Investigator at the hospital cannot be overruled and in the case of disagreement, both opinions will be provided with the report.

The MRC CTU is undertaking the duties of trial sponsor and is responsible for the reporting of SUSARs and other SARs to the regulatory authorities (MHRA, competent authorities of other European member states in which the trial is taking place and, if required, the research ethics committees) as follows:

- **SUSARs** which are **fatal** or **life-threatening** must be reported **not later than 7 days** after the MRC CTU is first aware of the reaction. Any additional relevant information must be reported within a further 8 days.
- **SUSARs** that are **not fatal or life-threatening** must be reported **within 15 days** of the MRC CTU first becoming aware of the reaction.
- A list of all SARs (expected and unexpected) must be reported annually.

The MRC CTU will inform the main REC in the UK of all SUSARs on an annual basis. The MRC CTU will send the reports directly to international central ethics committees unless there is a local co-ordinating centre, which will assume this responsibility. The MRC CTU will also send an annual safety report containing a list of all SARs to MREC (and all international centralised ethics committees). A copy of the report will be sent to the Principal Investigator at all institutions participating in the trial for their information.

17.10 Follow-up after adverse events

It is up to the discretion of the treating clinician as to whether trial participants should continue with protocol treatment following an adverse or serious adverse event subject to the dose modification schedules in **Section 8 for patients randomised to the lapatinib comparison or Appendix T, Section 6 for patients randomised to the bevacizumab comparison.** Treatment delays and dose modifications should be detailed on the requisite treatment form.

Figure 17. Safety Reporting Flowchart

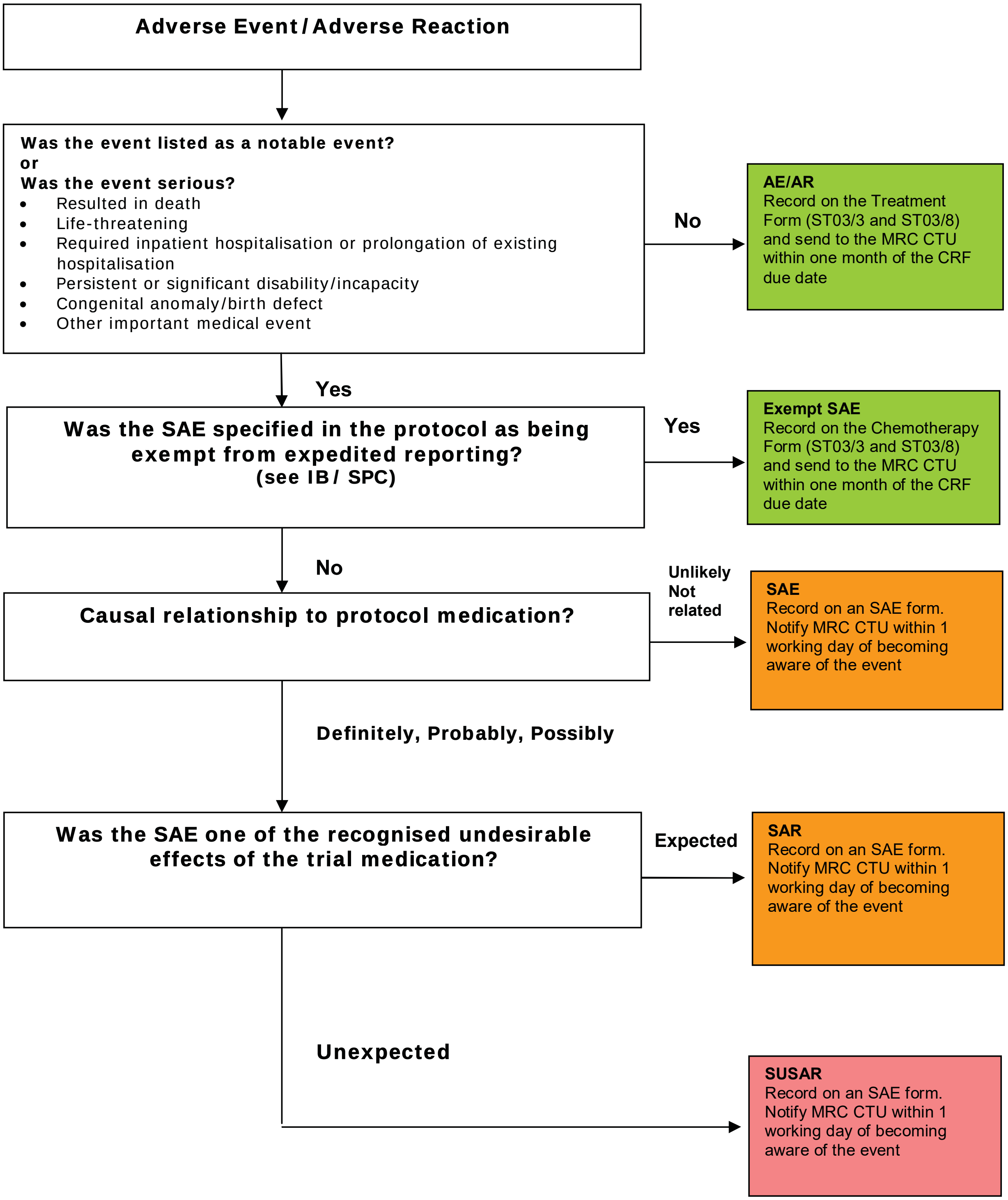

CRF: Case report form
SAE: Serious adverse event
SPC: Summary of product characteristics
IB: Investigator's brochure
SAR: Serious adverse reaction
SUSAR: Suspected unexpected serious adverse reaction

18. Patient Follow-Up

Patients randomised to the lapatinib comparison (or previously randomised to the bevacizumab comparison) will be assessed 3 weekly while they are receiving pre- and post-operative chemotherapy.

All patients, including those registered into the MRI and/or PET/CT sub-studies only, should commence 9 weekly follow-up assessments at 9, 18 and 27 weeks from the start of cycle 4. Patients will receive follow up 1 year from surgery, 6 monthly for years 2 and 3 and then annually until death.

Please note for those patients registered into the MRI substudy only, the same frequency of follow-up is recommended, however data collection beyond the post-operative period is not required for trial purposes.

If the patient attends between these appointments and a new event occurs, then an additional relevant Follow-up form (ST03/11, ST03/11-HER or ST03/11-IMG) should be completed. Follow-up forms should be also used when there is information concerning new events, such as local recurrence, detection of metastases, if patient receives further treatment.

If a patient does not follow the protocol treatment at any stage, or protocol treatment is stopped for whatever reason, the patient should remain in the trial for the purpose of follow-up and subsequent data analysis. Patients registered and randomised from the UK will be asked to consent for their personnel details held by the NHS and maintained by the NHS Information Centre and NHS Central Register to be used to obtain long term follow-up information on survival, in the event that patients are lost to follow-up in the clinical centres.

19. Stopping Trial Treatment

A patient may stop trial treatment for the following reasons:

- Tumour progression whilst on therapy
- Unacceptable toxicity
- Patient withdraws consent from protocol treatment
- Intercurrent illness, which prevents further treatment
- Any change in the patient's condition, which in the clinician's opinion, justifies the discontinuation of treatment

Please note that by providing consent to enter the trial, patients are agreeing to trial treatment, trial follow-up and data collection, therefore all CRF data return should still be returned to the trials unit as per protocol, unless the patient explicitly states their wish not to contribute further data to the study. This should then be recorded on the Withdrawal form **(see Section 23).**

20. Patient Moves to New Centre

For patients moving from the area, every effort should be made for the patient to be followed-up at another participating trial centre and for this trial centre to take over responsibility for the patient. A copy of the patients ST03 CRFs will need to be provided to the new site. Consent must be re-signed by the patient at the new centre.

21. Trial Closure

For regulatory purposes the end of the trial is defined as 12 weeks after the date of the last treatment visit for the last patient undergoing randomised protocol treatment. However, for randomised patients, this is followed by the non-interventional phase of long term follow-up, which will continue for a minimum of 5 years, after entry of the last patient or until death. Patients registered to the MRI and/or PET/CT sub-studies only will continue observational follow-up until the follow-up point for the primary analysis is reached. In all cases follow-up will initially be via the hospital, but in the longer term may employ national registers.

21.1 Site closure procedures

Site wishing to close to the ST03 trial should contact the ST03 Trial Manager to discuss site closure.

Before a site can close to the ST03 trial the following criteria must be met:

- No patients on active treatment
- No patients on active follow-up
- No outstanding data
- No unresolved data queries/SAEs
- Any issues identified at monitoring visits are now resolved
- All site payments are complete as agreed

The site closure documentation must be completed and returned to the MRC CTU who will contact MREC, Roche and GSK, as applicable, to confirm site closure. The site pharmacist will also be contacted to confirm drug destruction and documentation procedures for closure of the site. Once all close out procedures are complete the ST03 trial team will send confirmation of closure of the site and instruction for archieving arrangements.

22. Archiving

The documents which individually and collectively permit evaluation of the conduct of a clinical trial and the quality of the data produced are defined as essential documents. These documents serve to demonstrate the compliance of the investigator, sponsor and monitor the standards of GCP and with applicable regulatory requirements. They should be filed in an organised way that will facilitate management of the clinical trial, audit and inspection (Trial Master File). ST03 essential documents must be retained (archived) for 10 years to allow for audit and inspection by regulatory authorities and should be readily available upon request. An archive index/log should be maintained to record all essential documents that have been entered into the archive, and to track and retrieve documents on loan from the archive.

The Investigator should make the MRC CTU (Sponsor/trial organisers) aware of the storage arrangements for the documents to be stored at investigator sites. If the investigator becomes unable to store their essential documents, the MRC CTU should be notified in writing so that alternative storage arrangements can be agreed. If the investigator is no longer able to maintain custody of their essential documents, the MRC CTU should be notified in writing and the investigator/institution see to it that appropriate arrangements can be made.

23. Withdrawal of Patients

Patients should be given every encouragement to adhere to protocol treatment and follow-up, in order to reduce biases. However, a patient has the right to withdraw consent for participation in any aspect of this trial at any time. They may refuse to take

certain treatments, attend scheduled follow-up visits, or may move from the area. Clear distinction must be made as to whether the patient is withdrawing from trial treatments/procedures whilst allowing further follow-up, or whether the patient refuses **any** further trial treatments/procedures **and** follow up participation. In all instances the MRC CTU should be informed by the participating centre as soon as possible by the completion of the Withdrawal Form (ST03/15).

23.1 Withdrawal of Consent

If a patient explicitly withdraws consent to have any data recorded their decision must be respected and the ST03 Trial Manager must be notified in writing. All communications surrounding the withdrawal should be noted in the patient's records and no further ST03 CRFs should be completed for that patient.

Patients can change their minds about withdrawal at any time and re-consent to participate in the trial.

24. Statistical Considerations

24.1 Method of Randomisation

Patients will be randomised centrally using a computerised algorithm developed and maintained by the MRC CTU. Randomisation will be performed using the method of minimisation over a number of clinically important stratification factors with an additional random element. To decrease determinability, the factors are not listed here.

Recruitment to the bevacizumab comparison is now complete, for further details on the original ST03 trial design statistical considerations and for the treatment and follow up of patients in the bevacizumab comparison please refer to Appendix T.

24.2 Outcome Measures

24.2.1 Lapatinib Feasibility Study

Primary outcome measure

The primary endpoint for the feasibility study will be the establishment of a recommended dose (capecitabine and lapatinib) for a subsequent phase III trial. The recommended dose must have an estimated grade 3/4 diarrhoea rate of no more than 20%.

Formal safety reviews will occur after 10 and 20 patients have completed pre-operative mECX+lapatinib. Additionally, a further safety review will be conducted once 10 patients have completed 1 cycle of maintenance lapatinib. We intend to publish the results of the safety of mECX+lapatinib once the recommended dose is established.

In the first treatment cohort (dose level 0) patients start capecitabine at a lower than licensed (standard) dose of 1000mg/m2. Of the 1st 10 treated at this dose level:

- If 0 or 1 have grade 3/4 diarrhoea, the dose of capecitabine is increased to what is considered standard, 1250mg/m^2, in the next 10 patients.
- If 2 have grade 3/4 diarrhoea, a further 10 will be treated at the same dose level, if no more than 4/20 have grade 3/4 diarrhoea, this is the recommended final dose
- If 3 or more have grade 3/4 diarrhoea, the dose of capecitabine is maintained at 1000mg/m^2 but the lapatinib dose is reduced to 1000mg/m^2 in the next 10 patients.

At randomisation, the current dose of the drugs will be confirmed.

Secondary outcome measures

The secondary outcome measures will be feasibility in terms of (i) HER2 testing within an acceptable timeframe (proportion of test results returned within 10 and 15 working days of registration) and (ii) Assessment of HER-2 positivity rate in the study population (% of tumours tested that are IHC 3+ or IHC 2+ with confirmation of HER-2 amplification by DDISH). Toxicity, including notable event rates and the proportion of patients with a significant fall in LVEF, defined as a fall of at least 10% from baseline to below the lower limit of normal for MUGA or 55% for ECHO, will also be considered.

24.2.2 ST03 MRI Sub-study

Primary Outcome Measure

The primary outcome measure on which the study has been powered is the sensitivity of MRI, i.e. the proportion of patients in whom a complete resection is NOT possible who are correctly identified by the pre-operative MRI.

Secondary Outcome Measures

Specificity (proportion of patients undergoing an R0 resection who are correctly identified by the pre-operative MRI)
Overall accuracy (proportion of all surgical outcomes correctly predicted by the pre-operative MRI).
Association between apparent diffusion co-efficient (ADC) values and pathological response (Mandard Grade).

24.2.3. ST03 PET/CT Sub-study

Primary outcome measure

Progression-free survival in metabolic responders (>20% reduction in metabolic activity) vs non-responders

Secondary outcome measures

Metabolic response itself will be assessed as a secondary outcome measure for treatment comparisons.

In addition, the association between metabolic response and the following outcome measures will also be assessed:
Pathological response, as judged by Mandard grade (categories 1-3)
Radiological response prior to surgery
Ro resection rate
Overall survival

24.3 Sample Size

24.3.1 Lapatinib Feasibility Study

In the pilot study, it is estimated that 200-400 patients will undergo screening for HER-2 in order to provide 40 HER-2 positive patients for randomisation.

24.3.2 ST03 MRI Sub-study

The following assumptions have been made for the power calculation based on oesophageal and OGJ patients in the ST03 trial:

- Approximately 15% of such patients randomised in ST03 will not have surgery
- Approximately 35% of patients who undergo surgery will not have an R0 resection
- High power to detect a benefit to MRI should be retained at the potential cost of an increased risk of concluding MRI is useful when it is not – hence target power is 80-85% and significance level 10%

There is limited data on which to confirm these assumptions at present, therefore we present a minimum and maximum sample size which we believe to be achievable within the timeframe of the ST03 trial.

With true sensitivity of between 65% and 75%, using a Fleming design it will be possible to exclude rates below 50% with at least 80% power at the 10% significance level if 44-49 patients who do not undergo an R0 resection are included. To achieve this number of patients, a total of between 126 and 141 undergoing surgery and approximately 148 - 166 patients randomised and undergoing pre-operative MRI would be required.

To achieve 85% power to exclude the same rates, 52-58 non-R0 resections and therefore 175 – 195 patients undergoing MRI would be required. **Thus we aim to recruit a minimum of 150 patients and would increase this to a maximum of 200 if this can be achieved within the accrual period to ST03.**

24.3.3 ST03 PET/CT Sub-study

The study is primarily powered to detect a clinically relevant difference in early progression-free survival in metabolic responders and non-responders, while ensuring enough patients to estimate the negative predictive value (NPV) of the early PET scan with sufficient accuracy to exclude unacceptable rates. Here, NPV is the proportion of metabolic non-responders (patients predicted to be pathological non-responders) who prove to be pathological non-responders. We require this to be high, ideally >90%, as a 10% or greater rate of patients incorrectly taken off chemotherapy early would be considered unacceptable.

We propose a target sample size of **130 eligible patients**; this may need to be increased slightly to allow for exclusion of patients with inadequate baseline FDG uptake, or with uncontrolled diabetes. We have assumed that ~50% of patients will be metabolic responders (32% of patients in the Ott study and 50% of patients in the Municon study were metabolic responders based on achieving >35% reduction in SUV); sample size calculations with respect to progression-free and overall survival are not materially affected should the observed rate be 10% higher or lower than this.

With a total of 130 patients recruited over 12-18 months and followed for a minimum of 12 months, we expect to observe 67 events (progressions or deaths). This would give 80% power (2-sided significance level of 5%) to detect a HR of 0.5, corresponding to 1 year (2 yr) PFS rates of 53% (29%) and 73% (54%) in metabolic non-responders and responders respectively.

Patients from both the ECX+/- B and ECX+/-L randomisations may be included in the PET study. Depending on the distribution of patients, exploratory subgroup analyses within treatment arms may be possible. If at least 29 PFS events are observed in any subgroup, this would give 80% power to detect a HR of 0.34 (i.e. equivalent to the survival HR seen in the Ott study) and over 90% power to detect the recurrence-free survival HR (0.22) observed in the same trial amongst Ro patients. No data comparing treatment arms will be presented until the main trial has reported its primary outcome results.

Power for selected secondary outcome measures

With extended follow-up (i.e. extended to the completion of ST03) to increase the number of deaths observed, the same HRs for overall survival would be detectable with similar power. At this stage, it would also be possible to look not only at the prognostic value of metabolic response for overall survival, but also to gain initial data on the potential value of metabolic response as a surrogate marker of survival benefit to bevacizumab.

The operating characteristics of the early PET scan will also be assessed with respect to pathological response rate (Mandard grade). Approximately 7% of patients may not undergo surgery for reasons other than disease progression and therefore Mandard grade is likely to be assessable in approximately 120 patients. Assuming ~60 of these will be metabolic non-responders, the negative predictive value will be estimated with a standard error of ~6%. In particular, if the observed NPV is at least 90% (54/60), the lower limit of an exact 90% confidence interval would exclude NPV rates below 80%. If the overall diagnostic accuracy is >80%, close to that seen in the Ott study, the lower limit of an exact 90% CI would exclude rates below 73% when considering the whole study population (120 patients) and would still exclude rates below 70% when assessed within patients allocated ECX alone (n=60).

24.4 Interim Monitoring and Analyses

24.4.1 Lapatinib Feasibility study

Monitoring of toxicity data for this pilot study will occur in real time. Formal interim analyses of the accumulating data will be performed at regular intervals (approximately 6-monthly) for review by an Independent Data Monitoring Committee (IDMC) (see also section 19). These analyses will be performed by the trial team at the MRC CTU. The IDMC will be asked to give advice on whether the accumulating data from the trial, together with results from any other relevant trials, justifies continuing recruitment of further patients or further follow-up. A decision to discontinue recruitment, in all patients or in selected subgroups will be made only if the result is likely to convince a broad range of clinicians including those entering patients into the trial and the general clinical community. If a decision is made to continue, the IDMC will advise on the frequency of future reviews of the data on the basis of accrual and event rates. The IDMC will make recommendations to the Trial Steering Committee **(TSC, see section 19)** as to whether the trial should continue in its present form. While the trial is ongoing the accumulating data will remain confidential.

24.5 Outline Analysis Plan

The principal analysis methods for the primary outcome measures are described below; a more detailed analysis plan will be prepared and made available as a separate document.

24.5.1 Lapatinib feasibility study

(i) Acceptance of dosing regimen

As described in 13.2.2, the first 10 HER-2 positive patients randomised to lapatinib will be treated with a 20% dose reduction to capecitabine (1000mg/m^2/day) and there will be real-time monitoring of toxicity. If <2 out of the first 10 patients develop grade 3-4 diarrhoea during pre-operative treatment, then the dose of capecitabine will be increased to the full dose of 1250mg/m^2/day. If 2/10 patients develop grade 3-4 diarrhoea then a further 10 patients will be treated at this dose level to confirm the rate of 20% or less. If a 3rd patient in the first 10 develops grade 3-4 diarrhoea, then the dose of lapatinib will be reduced to 1000mg/day for the next 10 patients. The final decision about acceptability of dosing will also take account of other toxicity reported across the randomised arms including notable event rates and the proportion of patients with a significant fall in LVEF, defined as a fall of >10% from baseline to below LLN for MUGa/55% for ECHO.

ii) Feasibility of HER-2 testing

This will be based on the proportion of consenting patients for whom a formalin-fixed paraffin-embedded (FFPE) block containing tumour from the diagnostic biopsy can be retrieved from the pathology department, sent to a central laboratory (if applicable), be analysed for HER-2 status and a reliable result returned to the MRC CTU for treatment allocation within ten working days of their initial consent. The first 10 patients would be used as a run-in, the next 50 patients would then be used to assess the feasibility as follows: we require the proportion of samples with HER-2 results provided within 10 working days to be 90%. If we were to see a turnaround within 10 working days in ≤40/50 patients, this would not be considered acceptable (the 90% CI for the turnaround rate would lie entirely below 90%), and we would aim to implement changes and assess their impact in the next 50 patients. This could include introducing regional testing at selected centres, subject to appropriate training and accreditation. As HER-2 testing will be carried out in parallel with other staging investigations, a key criterion is that testing does not introduce delays in randomising or starting treatment. Evidence from the FOCUS3 trial suggests this is achievable in the majority of patients with a 15-day turnaround, therefore the proportion of sample tests achieving this without introducing treatment delays will also be recorded.

(iii) HER-2 positive rate

The proportion of all patients undergoing testing who have HER-2 positive disease, defined as IHC 3+ or IHC 2+ and DDISH +ve. Feasibility of a national HER-2 trial will depend on the HER-2 positivity rate. With 200 patients tested, if the HER-2 positive rate is <10%, the 95% CI would also exclude rates > 15%.

24.5.2 ST03 MRI Sub-study

Overall Accuracy

The minimum number of patients undergoing surgery under the sample size assumptions would be 126. With an overall accuracy of 80%, we would have >90% power to exclude rates below 70%.

If sensitivity of 70% and specificity of 85% is achieved, the diagnostic odds ratio (DOR, where a DOR of 1 indicates no discriminatory ability) would be approximately 13 ie the odds of a R0 resection in those predicted by MRI to undergo an R0 resection would be 13 x higher than the odds of an R0 resection in those predicted to undergo an R1 resection. Taking the lower limits of the corresponding confidence intervals (55% for sensitivity, 75% for specificity), the DOR would still be approximately 3.7.

Secondary Analyses

Reproducibility of the MRI technique using interobserver variability
Accuracy of MRI for T & N staging compared with histopathology.
Accuracy of MRI for T & N staging compared with the accuracy of conventional staging.

MRI response assessment based on RECIST Version 1.0 criteria compared with the histopathology tumour regression grade based on the Mandard grading system.
ADC values as a predictor of pathological response (Mandard grade)

24.5.3 ST03 PET/CT Sub-study

Follow-up of the PET study patients will continue until 67 PFS events have been observed. At this point PFS Kaplan Meier curves will be produced and compared using the log rank test. The date of the PET scan will be the start date for calculation of PFS duration; disease progression or death will be events and patients without an event will be censored at the date last known to be alive and free from progression.

A multivariate Cox regression analysis will be carried out including metabolic response and the major baseline prognostic factors as covariates, in order to assess the independent prognostic value of early metabolic response.

Following analysis of the main trial results, and dependent upon the distribution of patients across the two randomisations, these analysis will be repeated in the subgroups receiving or not receiving bevacizumab and potentially lapatinib. Follow-up may be extended in these subgroups to obtain a minimum of 29 PFS events.

Follow-up of all patients will continue as required in ST03, enabling a further analysis of metabolic response as a predictor of overall survival to be carried out when the primary results of ST03 are available. An exploratory analysis of both metabolic response rate and survival by randomised treatment allocation will give an indication of whether metabolic response is a potentially useful surrogate marker for treatment benefit.

The sensitivity, specificity, NPV, PPV and overall accuracy of the early PET scan with respect to pathological response rates (based on Mandard grade, <10% residual cancer) will be presented with 95% confidence limits in all patients and in the treatment subgroups. In addition, the Mandard grade (1-5) will be compared in metabolic responders and non-responders using a chi-square test for trend.

Response rates (RECIST) and R0 resection rates will also be presented and compared in metabolic responders and non-responders.

Exploratory analyses will look for evidence that the prognostic value of early metabolic reponse differs in patients allocated ECX vs ECX+B and at a later stage ECX vs ECX + L. Tests for interaction between treatment arm and early PET response with respect to pathological response rates and progression free survival will be carried out. It is acknowledged that these will have low power; an interaction p value < 0.1 would give suggestive evidence of differential treatment effects.

25. Trial Monitoring

25.1 Risk Assessment

The MRC CTU has performed a risk assessment to assess the impact of trial participation on the rights and safety of patients, the reliability of trial results and the impact of trial, including the lapatinib feasibility study, results on the research site leading the trial. This has guided the development of procedures in the trial with respect to informed consent, confidentiality and trial monitoring.

25.2 Monitoring at the MRC CTU

The MRC CTU will conduct day-to day central monitoring of the trial. The MRC CTU staff will:

- Check that participating centres have confirmed that they have an up to date trial master file
- Check centres adhere to the current version of the protocol
- Review source documents centrally and on site (e.g. pathology reports, consent forms) to confirm patients existence where possible
- Perform data entry (database) checks for validity and consistency
- Missing or questionable data will be returned to the centre for correction or clarification via a data clarification form (DCF)
- Check that CRFs are completed by authorised persons listed on the ST03 Signature and Delegation Log
- Ensure that adverse events are reported in accordance with the protocol and CRF
- Review data return rates from centres at regular intervals
- Review recruitment during the recruitment period of the trial
- For the feasibility study rates of diarrhoea will be monitored in real time and the CI and trial physician will be informed once 10 patients are treated.

25.3 Clinical Site Monitoring

25.3.1 Direct Access to Data

Participating investigators should agree to allow trial-related monitoring, including audits, ethics committee review and regulatory inspections by providing direct access to source data/documents as required. Patients' consent for this must be obtained.

25.3.2 Centre Selection for Site Monitoring

A visit may be made during the trial to:

- Larger centres, who have randomised a minimum of 10 patients
- A random sample of smaller centres
- Those centres found to have poor compliance (e.g. poor quality data or late documentation)

Please note that all centres could potentially be visited.

25.4 Confidentiality of Trial Documents and Patient Records

The PI must assure that patient's anonymity will be maintained and that their identities are protected from unauthorised parties. On CRFs patients will not be identified by their

names, but by an identification code. The PI should keep a patient enrolment log showing codes, names and addresses.

25.5 Criteria for Stopping the Trial

Strict monitoring of tumour perforation and cardiovascular events during the safety stage of the bevacizumab comparison will take place under the guidance of an Independent Data Monitoring Committee (IDMC). Providing the events observed do not exceed a clinically relevant rate, and the IDMC identify no other reason to modify or terminate the trial at the 1st formal interim review with ~200 patients randomised, the trial will progress to the second efficacy stage. This has taken place as of June 2011. After this stage, the safety and efficacy data will continue to be monitored by the IDMC (these meetings are expected to be approximately every 6-12 months, depending on the accrual rate) and may recommend closure on the basis of the safety and efficacy data they review, as described in Appendix U, Section 9.4.1. The final decision about early closure of the trial rests with the independent Trial Steering Committee (TSC).

Based on a review of cumulative data from the ST03 trial, the IDMC noted an increased risk of anastamotic leaks in a subset of patients receiving bevacizumab. Of the 255 oesophagectomies/gasto-oesophagectimes in the bevacizumab comparison the anastamotic leak rate was 9% in the sECX arm and 24% in the sECX+B arm. The IDMC looked at other factors that could explain this but it was not apparent on initial review of the data. As a precautionary measure after agreement from the TSC an Urgent Safety Amendment was implemented in October 2013 to amend the inclusion criteria to exclude patients with lower oesophageal and OGJ adenocarcinomas from entering the bevacizumab comparison. The comparison remained unchanged for patients with gastric adenocarcinomas who did not require an oesophagogastrectomy. This Urgent Safety Amendment also mandated that patients with lower oesophageal and OGJ adenocarcinomas who were already entered into the sECX+B arm (Arm B) must not receive any further pre-operative bevacizumab. Following surgery patients may resume treatment with bevacizumab provided that there are no other contraindications.

With respect to the lapatinib feasibility study, rates of ≥grade 3 diarrhoea will be monitored in real time. Additional cardiac monitoring will be performed during the study as performed for phase II bevacizumab comparison. Formal interim analyses of the accumulating data will be performed at regular intervals (approximately 6-monthly) for review by the IDMC, as described in Section 24..4.1, who may advise on early termination or modification to the trial. The IDMC will make recommendations to the Trial Steering Committee **(TSC, see section 30)** as to whether the trial should continue in its present form. While the trial is ongoing the accumulating data will remain confidential.

26. Ethical Considerations and Approval

26.1 Ethical considerations

Within the randomised components of this trial, neither the patients nor their physicians will be able to choose the patients' treatment. Treatment will be allocated randomly using a computer-based algorithm. This is to ensure that the groups of patients receiving each of the different treatments are similar.

26.1.1 Patients Randomised to the Bevacizumab Comparison (now closed to randomisations)

Patients randomised to the trial arm sECX+B are required to attend hospital after completion of their post-operative chemotherapy for an additional 6 injections of bevacizumab given as maintenance therapy. Treatment on the sECX+B arm will last for approximately 27 weeks after surgery rather than 9 weeks if randomised to the sECX arm.

Patients participating in the trial will have some additional hospital visits and extra assessments that would not normally be considered as part of routine care to ensure that it is safe for the patient to participate in the trial and to continue on trial treatment.

During stage 1 of the bevacizumab comparison the first 200 patients were required to receive:

- A minimum of 2 extra heart scans (ECHO/MUGA) before surgery and after completing the post-operative treatment.
- 12 additional assessments to monitor patients heart (ECG) before each cycle of treatment
- 13 urine samples

The original ST03 trial bevacizumab comparison was in two stages. The first stage, phase II, was completed in April 2010 and confirmed the safety and feasibility of combining bevacizumab with chemotherapy in this clinical setting (Okines, ASCO 2011). The second stage, phase III, will assess the efficacy of this combination.

In addition, the requirement for the additional cardiac assessments have now been reviewed by the IDMC and are no longer required for the bevacizumab comparison.

See Section 7.2 of Appendix T for specific severe adverse events of bevacizumab and recommendations for treatment.

26.1.2 Patients Randomised to the Lapatinib Comparison

Patients in the mECX + L arm may be at risk of excessive diarrhoea. There may also be a risk of dermatological toxicity, hepatotoxicity, cardiotoxicity and pulmonary toxicity. The aim of the study is to determine a tolerable dose of this combination, such that the potential benefits of targeting patients who are HER-2 positive with lapatinib can be weighted against any negative asplects.

Patients randomised to the trial arm mECX+L will be required to attend hospital after completion of their post-operative chemotherapy for an additional 6 visits during the period of lapatinib maintenance therapy. Treatment on the mECX+L arm will last for approximately 27 weeks after surgery rather than 9 weeks if randomised to the sECX arm.

Patients participating in the lapatinib comparison will have some additional hospital visits and extra assessments that would not normally be considered as part of routine care to

ensure that it is safe for the patient to participate in the trial and to continue on trial treatment.

These include:

- A minimum of 2 extra heart scans (ECHO/MUGA) before surgery and after completing the post-operative treatment.
- 12 additional assessments to monitor patients heart (ECG) before each cycle of treatment

See Section 8 for expected toxicities within the lapatinib comparison and recommendations for treatment.

26.1.3 Patients Registered to the ST03 MRI Sub-study

Patient participating in the MRI sub-study will be required to have two additional MRI scans, one before treatment commences and another prior to surgery.

The MR imaging data will be collected prospectively but **not** used to inform patient management.

26.1.4 Patients Registed to the ST03 PET/CT Sub-study

The ST03 PET/CT sub-study is an observational registration only sub-study . Patients participating in the PET/CT sub-study will be required to have an extra FDG-PET/CT scan following cycle 1 of chemotherapy. The baseline FDG-PET/CT scan would form part of routine clinical care.

The PET imaging data will be collected prospectively but **not** used to inform patient management.

26.2 Ethical approval

The protocol has a Favourable Opinion from its main Research Ethics Committee (REC), but each site must also gain a favourable Site Specific Assessment (SSA) before patients can be entered into the trial. The patient's informed consent to participate in the trial should be obtained after a full explanation has been given of the treatment options, including the conventional and generally accepted methods of treatment.

The right of the patient to refuse to participate in the trial without giving reasons must be respected. After the patient has entered the trial, the clinician must remain free to give alternative treatment to that specified in the protocol, at any stage, if he feels it to be in the best interest of the patient. However, the reason for doing so should be recorded and the patient will remain within the trial for the purpose of follow-up and data analysis according to the treatment option to which he has been allocated. Similarly, the patient must remain free to withdraw at any time from the protocol treatment without giving reasons and without prejudicing his further treatment.

27. Regulatory Approval

This trial has been registered with the MHRA and has been granted a Clinical Trial Authorisation (CTA). The CTA reference is 00316/0221/001.

This is a Clinical Trial of an Investigational Medicinal Product (IMP) as defined by the EU Directive 2001/20/EC. Therefore, a CTA is required in the UK. The EUdraCT number for the trial is 2006-000811-12.

This protocol has been reviewed and approved by the MHRA.

Safety reports, including expedited reporting and SUSARS, as well as the progress of the trial and safety issues will be reported to the MHRA in accordance with local requirements and practices in a timely manner.

28. Indemnity

University College London (UCL) holds insurance against claims from participants for injury caused by their participation in this clinical trial. Participants may be able to claim compensation if they can prove that UCL has been negligent. However, as this clinical trial is being carried out in a hospital, the hospital continues to have a duty of care to the participant of the clinical trial. UCL does not accept liability for any breach in the hospital's duty of care, or any negligence on the part of hospital employees. This applies whether the hospital is an NHS Trust or otherwise. Participants may also be able to claim compensation for injury caused by participation in this clinical trial without the need to prove negligence on the part of UCL or another party. Participants who sustain injury and wish to make a claim for compensation should do so in writing in the first instance to the Chief Investigator, who will pass the claim to the UCL's Insurers, via the UCL's office.

29. Finance

The ST03 trial will be co-ordinated at the MRC CTU in London. The trial is funded by Cancer Research UK, Grant No: C1504/A6410 and supported by the MRC CTU.

The trial has National Cancer Research Network (NCRN) approval and, therefore, local NCRN funds may be available at each centre to support entry of patients into this trial.

29.1 ST03 Bevacizumab Comparison Funding

Roche F Hoffmann-La Roche Limited, Basel have agreed to support this trial by providing free bevacizumab for patients randomised to the sECX+B investigational arm (Arm B) of this trial, an educational grant to cover the costs of the additional cardiac investigations for the first 200 patients and provide centres with per patient payments for all patients participating in the sECX+/-B randomisation of this trial.

29.2 ST03 Lapatinib Feasibility Study Funding

GlaxoSmithKline have agreed to support this trial by providing free lapatinib for patients randomised to the mECX+L investigational arm (Arm D) of the lapatinib feasibility study, an educational grant to cover the costs of HER-2 testing for all patients screened for the study, the additional cardiac investigations for 40 HER-2 positive patients and provide centres with per patient payments for all HER-2 positive patients participating in the feasibility study and randomised to sECX Vs mECX+L.

29.3 ST03 MRI Sub-study Funding

Separate funding has been secured for the scans via a Cancer Research UK Biomarkers & Imaging Discovery & Development (BIDD) grant. Reimbursement for the scans will be subject to receipt of all required information for each patient. Only patients confirmed eligible for ST03 and who meet the MRI sub-study inclusion criteria will receive reimbursement for MRI scans performed.

29.4 Trans-ST03 Funding

Trans-ST03 for the bevacizumab comparison has received funding from CRUK for the collection of blood and tissue samples. ST03 pathology departments will receive a payment of £30 per patient sample (£20 for the resection & £10 for the biopsy) that is submitted to the Leeds pathology laboratory. Sites will also be supplied with pre-paid blood boxes.

The Trans-ST03 for the lapatinib comparison, MRI and PET/CT sub-studies collection of blood and tissue samples is funded by the RMH. ST03 pathology departments will receive a payment of £30 per patient sample (£20 for the resection & £10 for the biopsy) that is submitted to the RMH pathology laboratory. Sites will also be supplied with pre-paid blood boxes.

30. Trial Committees

30.1 Trial Management Group (TMG)

A Trial Management Group (TMG) has been formed comprising the Chief Investigator, other co-investigators and members of the MRC CTU. The membership of the TMG may be expanded if other groups of trialists wish to participate. The TMG will be responsible for the day-to-day running and management of the trial and will meet by teleconference at least 6 monthly and in person as needed. The TMG members are detailed in **Appendix O**.

30.2 Trial Steering Committee (TSC)

A Trial Steering Committee (TSC) will be formed to provide overall supervision for the trial and provide advice through its independent chair. The ultimate decision for the continuation of the trial lies with the TSC. The TSC will meet twice a year.

30.3 Independent Data Monitoring Committee (IDMC)

An Independent Data Monitoring Committee (IDMC) will be formed. The IDMC will be the only group who sees the confidential, accumulating data to the trial. Reports to the IDMC will be produced by the MRC CTU. The IDMC will meet within 6 months of the trial opening with the frequency of meetings dictated by the IDMC. The IDMC will consider data in accordance with the analysis plan **(see Sections 24.4 and 24.5**) and will be advisory to the TSC. The IDMC can recommend premature closure or reporting of the trial, or that recruitment to any research arm be discontinued.

Further details of IDMC functioning and the procedures for interim analysis and monitoring are provided in the IDMC charter (available on request).

Figure 30 - Diagram of Relationships between Trial Committees

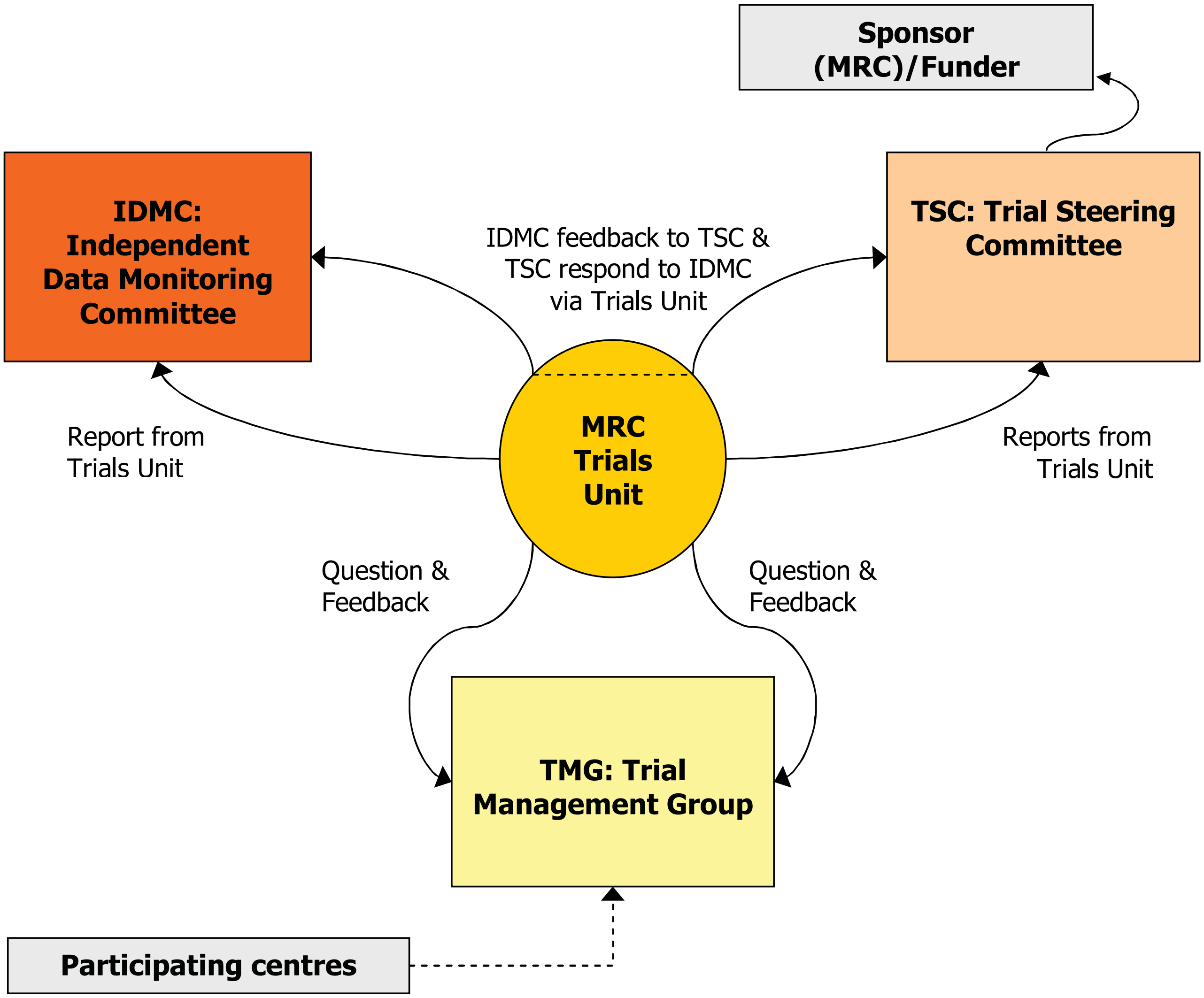

31. Ancillary Studies

31.1 Rationale for QoL Study

The primary objective of the original ST03 trial bevacizumab comparison was to assess the safety and efficacy of adding monoclonal antibody bevacizumab to ECX chemotherapy administered peri-operatively in patients with operable adenocarcinoma of the stomach and oesophagogastric junction. Stage 1 of the study assessed the safety of the investigational arm and stage 2 assessed whether the addition of bevacizumab to standard treatment, results in improved overall survival.

The primary objective for the lapatinib feasibility study is to assess the safety of adding lapatinib, a small molecule tyrosine kinase inhibitor of HER-2 and EGFR, to ECX chemotherapy administered peri-operatively in patients with operable adenocarcinoma of the lower oesophagus, stomach and oesophagogastric junction.

Assessment of health-related quality of life (QOL) as a secondary end point in these studies will provide detailed information about the impact of treatments, of patients' self reported physical, emotional and social well-being. The purpose of including a QOL assessment, therefore, is to collate data that can subsequently inform clinical decision-making, because it will allow survival benefits to be considered alongside risks of treatment and impact on QOL. In addition detailed QOL data will provide data for full informed consent if other clinical outcomes are equivalent.

31.2 Quality of Life Measures

Generic aspects of Quality of life will be assessed with the EORTC Quality of Life Questionnaire (QLQ-C30) version 3 (84). This is a generic cancer instrument composed of multi-item and single scales. There are five functional scales (physical, role, emotional, social and cognitive function), three symptom scales (fatigue, emesis and pain), a global health scale and six single items (dyspnoea, insomnia, anorexia, constipation, diarrhoea and financial difficulties). This questionnaire lacks some items that are relevant in patients with stomach cancer and these will be assessed with the disease specific module, the EORTC QLQ-STO22 (85). The module has been developed and validated in patients undergoing surgery and chemotherapy for gastric cancer. It includes scales assessing eating restrictions, dysphagia, body image and reflux as well as some single items (86).

31.3 Administration of QoL Questionnaires

Baseline questionnaires will be given to the patient in clinic after written consent has been obtained and within 3 days prior to, or on the day of randomisation, but in either case before the patient is informed which trial treatment s/he has been assigned to. Follow-up questionnaires will be subsequently administered during treatment and during the follow up period. Patients will be asked to fill out the questionnaires themselves as completely and accurately as possible. The average time to complete the entire questionnaire is 10-15 minutes. It is recommended that a key person (e.g. research nurse) at each centre be responsible for the data collection to optimise compliance and completeness of the data. The person responsible for questionnaire administration should check each questionnaire for its completeness, ensuring that the correct date of completion and patient identifiers are present. The research nurse should approach patients at appropriate clinical visits to complete a questionnaire. If no clinical visit is scheduled for the patient (within a time window of 4 weeks around the expected date), the responsible person should organise posted completion of the questionnaire. Time points for QOL assessments are detailed below in **Table 31.1.**

If the patient stops treatment early or does not proceed to surgery, where appropriate the QOL questionnaire should still be completed at the same time points. Please see the individual patient schedules for approximate dates, or contact the Trials Office for an updated CRF schedule.

Please note patients registered into the MRI and/or PET/CT sub-studies only are not required to complete QoL questionnaires.

Table 31.1 QoL Assessments

**QoL Assessments**	**Time-Point**
Pre- Randomisation QoL (1)	Within 1 week before randomisation
Treatment QoL (2)	Cycle 3 Day 21
Pre-surgery QoL(3)	Within 3 weeks before surgery
End of Post Surgery Treatment QoL (4)	Cycle 6, Day 21
6 Month QoL (5)	6 month Post Surgery
12 month QoL (6)	12 month Post Surgery
18 month QoL (7)	18 month Post Surgery
24 month QoL (8)	24 month post Surgery

31.4 Radiology Review

For quality assurance, a reference radiologist may review up to 10% of pre-treatment CT scans. Requests would be made every 6 months with patients being randomly selected centrally by staff at the MRC CTU for review, from those patients who have completed protocol treatment. Scans for those patients selected would be sent by the CTU to the reviewer. All scans submitted for review would be returned to the clinical centre once the review is complete.

31.5 Health Economics

A health economics (HE) sub-study will be performed for the bevacizumab comparison. Core resource use information will be collected, using CRFs on days in hospital (by speciality) and outpatient visits. Data being collected on concomitant medication will also be used in the economic analysis. Information on patients' use of primary care and community-based services will be collected as additional questions in the QL questionnaire. Costs will be calculated on the basis of representative UK unit costs at the point of analysis. Health outcomes will be assessed in terms of quality-adjusted life years (QALYs). Quality adjustments will be based on patients' responses to the EQ-5D health status measure which will be administered at baseline and each point of follow-up as part of the QL questionnaire (**Appendix P**).

31.6 MRI Sub-Study

A number of ST03 centres will be participating in the MRI sub-study. The MRI sub-study is an observational study designed to assess the potential benefit of MRI in the selection of patients for surgery based on the ability of MRI to predict patients who are likely to have a positive circumferential resection margin (CRM).

The study will use T2 weighted high resolution MRI as a predictive biomarker for resectability in patients with oesophageal and oesophagogastric junction cancer recruited into the ST03 trial. The MR imaging data will be collected prospectively but

not used to change patient management. In this way the MRI prediction of resectability can be validated against the surgical and histological findings and the integrity of the chemotherapy trials maintained.

31.7 FDG-PET Sub-study

A number of ST03 centres will be participating in the FDG PET sub-study. This is an observational study designed to assess the potential role of early interim FDG PET in the evaluation of response to conventional and investigational chemotherapeutic agents.

The baseline FDG PET scan is part of standard clinical care. If this demonstrates metastatic disease, the patient will not be eligible to enter the main STO3 clinical trial.
The early interim FDG PET scan will obtain data on the prognostic value of metabolic response for overall survival, and also to gain initial data on the potential value of metabolic response as a surrogate marker of survival benefit to bevacizumab.

32. ST03 Pathology Research

Please note there is a separate ST03 Pathologist Guidance Booklet which provides details about the pathology work up of the resection specimen and the tissue blocks that should be taken for the Trans-ST03 sub-study.

The ST03 pathology research involves two sub-studies:

1. Central Pathology Review
2. Trans-ST03: Blood and Tissue Collection, which will be stored for future research.

32.1 ST03 Central Pathology Review

Two recent audits into the reporting of oesophageal and gastric cancer pathology in the UK have demonstrated that pathology reports were often incomplete despite the introduction of the Royal College of Pathologists reporting proforma (87-89). We recognise that accurate, standardised and complete pathology data will strengthen this clinical trial.

Central Pathology Review of all pre-treatment biopsies and all resection specimens from all patients randomised to the bevacizumab comparison (now closed to randomisation) and the lapatinib comparison will enable us to comprehensively assess the quality of the pathology within the ST03 trial and lapatinib feasibility study and to ensure that high quality pathology data are available for all analyses.

For patients registered to the MRI sub-study the biopsy and resection slides should be sent to Leeds as described below.
However the Central Pathology Review will not be performed for the patients registered into the PET/CT sub-study only therefore there is no need to send any material for Central Pathology Review for these patients.

All ST03 centres are expected to participate in the pathology review. Patient consent for the Central Pathology Review is included within the PIS A and Consent Form A for the bevacizumab comparison (now closed to randomisation) and PIS B and Consent Form B for the lapatinib comparison.For those patients participating in the MRI sub-study only consent for the Central Pathology Review is included within the MRI sub-study PIS and Consent Form.

After the pathology of the resection specimen for the patient randomised to the bevacizumab comparison or lapatinib comparison or registered for the MRI sub-study has been fully reported and reviewed by the local MDT, which is usually within 4 weeks after the surgical procedure, the ST03 RN from each centre will need to request the following material from their local pathology department for the ST03 Central Pathology Review using the relevant ST03 Pathology Request Letter provided.

Table32.1: Pathology Review Material

Pathology Review Material	***Quantity***
Pre-treatment biopsy Haematoxylin/Eosin stained slides	*all*
Pre-treatment biopsy pathology report	*X1*
Resection specimen Haematoxylin/Eosin stained slides	*all*

Resection specimen pathology report	*X1*
Macroscopic images of cross-sections (for patients participating in the MRI sub-study)<br>*Other macroscopic images if available.*	*all*<br><br>*all*

This request should usually coincide with the request for paraffin blocks for the Trans-ST03 sub-study detailed in Section 32.2 provided that the patient has separate given consent for the Trans-ST03 blood and tissue collection.

The HE stained slides should be labelled with the name of the trial (e.g. ST03), patient's trial number and paraffin block identifier (usually a combination of the pathology case number and additional numbers or letters depending on local practice) before sending the material to the Leeds laboratory (address below) together with a copy of the pathology reports and Central Pathology Review Form (ST03/Path-HER). Patient's names need to be blacked out (made illegible) on the slides as well as on the copy of the pathology reports. Depending on local practice, the so called 'paraffin block identification list' (the list which specifies what material is embedded in which particular paraffin block) may not be part of the macroscopic description of the pathology report, so this 'block list' will also need to be retrieved for each case from the pathology department and submitted together with the HE stained slides and copy of the reports to Leeds.

One copy of the Central Pathology Review Form (ST03/Path-HER) should be retained by the local pathologist along with the ST03 Pathology Request Letter, a second copy should be returned to the RN at the site to be filed in the patient file and a third copy should be sent to the MRC CTU as confirmation that the pathology samples have been sent for review.

As all HE slides included in the Central Pathology Review will be returned to the centre after being scanned it is recommended that local pathologists submit the original diagnostic HE stained slides for this purpose. There is no need to recut blocks for the purpose of the Central Pathology Review.

If there are major discrepancies between the local pathologist and the Central Pathology Review panel that might result in changes to the patient's prognosis, the case will first be discussed between the reviewers and the local pathologist. If confirmed by the local pathologist, a letter will be sent to the recruiting clinician and the case should be re-discussed at the local MDT Meeting.

The ST03 Central Pathology Review is led by Dr Heike Grabsch, Senior Clinical Lecturer in GI Pathology and Honorary Consultant Histopathologist in Leeds.

All material for the ST03 Central Pathology Review should be sent directly to the ST03 Central Pathology Review panel at the following address:

c/o Dr Heike Grabsch
Pathology and Tumour Biology
Leeds Institute of Molecular Medicine
Wellcome Trust Brenner Building, Level 4
St James's University Hospital
Beckett Street
Leeds, LS9 7TF

Please note that the Central Pathology Review will not be performed for the patients registered into the PET/CT sub-study only therefore there is no need to send any slides to Leeds for these patients.

32.2 Trans-ST03: Tissue and Blood Collection for Translational Research

The Trans-STO3 sub-study aims to collect blood and tissue samples from oesophagogastric cancer patients entered into the ST03 bevacizumab comparison (now closed to randomisation), ST03 lapatinib feasibility study and/or registered into the MRI and PET/CT sub-studies. The material will be processed and stored so that researchers can perform studies aiming to identify molecular markers that predict which patient will benefit most from which therapeutic approach. All ST03 sites are expected to participate in the Trans-ST03 sub-study.

Patient Consent for Translational Research

In order to collect the tissue and blood samples for the Trans-ST03 sub-study, patients will be required to sign the **Trans-ST03 Consent Form**. The Trans-ST03 sub-study should be discussed in full with the patient at the same time as the ST03 lapatinib feasibility study and/or the MRI and PET/CT Sub-studies are explained, information on the Trans-ST03 sub-study is included within PIS B for the lapatinib comparison and the PIS for the MRI and PET/CT sub-studies.

The site will be asked at the time of randomisation of the patient into the lapatinib comparison or registration of the patient into the MRI and/or PET/CT sub-studies whether the patient has given consent to participate in the Trans-ST03 study.

Although most patients are expected to consent to participate in the translational research programme, the wishes of patients who do not want to be involved in this part of the ST03 trial will be respected and they will be allowed to enter the ST03 feasibility study and/or MRI and PET/CT sub-studies without contributing samples to the translational research component.

Custodians of the Translational Research Samples

The custodian of the samples is UCL. Proposals for translational research projects involving the ST03 material should be registered with the ST03 Trial Manager and will be considered for approval by the ST03 Sample Access Committee (SAC) (see Appendix O for contact details) and Independent Trial Steering Committee (TSC).

Blood Samples

- A minimum of 4 x 5ml or 2 x 10ml blood should be collected into standard EDTA containing tubes ideally at baseline prior to starting treatment. Should this opportunity have been missed it is still acceptable to collect a blood sample at a later date. However, in such a case it should be clearly indicated on the form at what stage of treatment the sample has been taken.
- Each tube should be labelled with the Trial name (i.e. ST03) the patient's trial ID number if available, patient initials, Hospital No. and date and time of blood collection before placing it in the pre-paid Royal Mail 'Safebox' supplied to your site at the time of site set up. It is important that the Trans-ST03 Blood Sample Information Form should also be sent along with the blood sample to the Leeds laboratory.

Pre-treatment Biopsy Blocks

Paraffin embedded diagnostic endoscopic biopsies are very small. However, these biopsies will be an invaluable resource to validate whether potential molecular markers are truly able to predict response to therapy. The tissue within these blocks will never be sectioned to exhaustion. The paraffin block will be returned to the local pathology department instantly if a local clinical necessity arises.

Tumour Resection Specimen Blocks

Please refer to the ST03 Pathologist Guidance Document for further details regarding the pathology work up of the resection specimen and preparation of additional paraffin blocks for the translational studies if appropriate.

It will help to ease the workflow in the pathology department if the pathologist is informed at the time of specimen receipt in the pathology laboratory that a particular patient has been recruited into the ST03 trial. This can be achieved by for example attaching a prominent coloured sticker with the trial name onto the histopathology request form.

The local pathologist is asked to provide the following material:

Table 32.2: Requested Material

**Specimen**	**No. of Paraffin Blocks**
Either representative viable primary tumour or in the case of a complete histopathological response material from areas with evidence of tumour regression	3
Non-involved gastric mucosa from different anatomical regions	3
Non-involved oesophageal mucosa (if available)	1
Lymph node with metastases if present	1
Lymph node with no metastasis	1
Other metastases if present	1

If preferred by the local pathology department, the pathologist in Leeds or RMH can select the most appropriate blocks for the Trans-ST03 sub-study at the time of ST03 Central Pathology Review. Alternatively, under exceptional circumstances if the local pathologist does not want to submit the HE stained slides, all blocks can be shipped to Leeds/RMH and the Leeds/RMH laboratory will recut all blocks.

Should there be any queries, please contact the ST03 Trial Manager at the MRC CTU.

Request and Shipment of Trans-ST03 Samples

The research staff from each centre should request the Trans-ST03 material from the pathology department(s) of the hospitals where the resection and the biopsy were performed using the relevant ST03 Pathology Request Letter provided. This request should coincide with the request of slides and pathology reports for the central pathology review e.g. approximately 4 weeks after surgery, if applicable. The material should be submitted together with the Trans-ST03 Tissue Collection CRF (Trans-ST03/HER). One copy of the form should be retained by the local pathologist along with the pathology request letter, a second copy should be returned to the RN at the site to be filed in the patient file and a third copy should be sent to the MRC CTU as confirmation that the biopsy and resection samples have been sent for storage.

On receipt of the material, the research staff will anonymise the tissue blocks if necessary. The pathology specimen number and block identification number or letters need to stay on the block, only patient names need to be remove or blacked out. In addition, blocks should be labelled with the patient's trial number, patient initials and hospital number using a sticky label prior to sending the material to the Leeds laboratory/RMH (see Section 32.5) in appropriate packaging.

Trans-ST03 Samples from Patients Randomised to the Bevacizumab Comparison (Arms A and B): now closed to randomisations
The Trans-ST03 sample collection for all patients randomised to the bevacizumab comparison (arms A and B) is performed at Leeds. Please send all samples for patients who have consent to Trans-ST03 to the following address:

c/o Dr Heike Grabsch
Pathology and Tumour Biology
Leeds Institute of Molecular Medicine
Wellcome Trust Brenner Building, Level 4
St James's University Hospital
Beckett Street
Leeds, LS9 7TF

The biopsy blocks of all patients who consent and are registered for HER-2 testing within the bevacizumab comparison are sent to RMH laboratory for centralised testing of HER-2 status . If the centre has been approved for local HER-2 testing the biopsy block will be sent to RMH laboratory for central verification.

The RMH will forward the biopsy blocks for those patients who are randomised to the bevacizumab comparison and have consented to Trans-ST03 to Leeds. The baseline blood samples and resection specimen blocks required for Trans-ST03 should be sent to the Leeds laboratory at the address provided above.

Please note that biopsy blocks sent to RMH laboratory for HER-2 testing for the lapatinib feasibility study will be returned to the local pathologist if, after testing, the patient does not consent to Trans-ST03 or is ineligible for randomisation into the ST03 trial or registration into the and/or MRI and PET/CT sub-studies.

Trans-ST03 Samples from Patients Randomised to the Lapatinib Comparison (Arms C and D) or registered to the MRI and/or PET/CT sub-studies only
The Trans-ST03 sample collection for all patients randomised to the lapatinib comparison (arms C and D) or registered to the MRI and/or PET/CT sub-studies only is performed at RMH. Please send all samples to the following address:

RMH Laboratory address:
Sandra Yusuf-Adam
Biological Specimens Co-ordinator
GI & Lymphoma Unit
Department of Medicine
Royal Marsden Hospital NHS Foundation Trust
Fulham Road
Chelsea, SW3 6JJ

The biopsy blocks of all patients who consent and are registered for HER-2 testing within the lapatinib feasibility study will be sent to RMH laboratory for centralised testing of HER-2 status prior to entry into the ST03 feasibility study or imaging sub-studies (as per Sections 5.4 and 5.5). If the centre has been approved for local HER-2 testing the biopsy block will be sent to RMH laboratory for central verification.

The RMH will retain the biopsy blocks for those patients who are randomised to the lapatinib comparison and/or registered for the MRI and/or PET/CT sub-studies only and have consented to Trans-ST03. The baseline blood samples and resection specimen blocks required for Trans-ST03 should be sent to the RMH laboratory at the address provided above.

Please note that biopsy blocks sent to RMH laboratory for HER-2 testing for the lapatinib feasibility study will be returned to the local pathologist if, after testing, the patient does not consent to Trans-ST03 or is ineligible for randomisation in the lapatinib comparison or registration into the MRI and/or PET/CT sub-studies.

If the patient has not consented to HER-2 testing but is registering for the MRI and/or PET/CT sub-studies only and has consented to the Trans-ST03 sample collection the Trans-ST03 samples should be sent to the address provided above.

32.3 Trans-ST03 Funding

Trans-ST03 for the bevacizumab comparison has received funding from CRUK for the collection of blood and tissue samples. ST03 pathology departments will receive a payment of £30 per patient sample (£20 for the resection & £10 for the biopsy) that is submitted to the Leeds pathology laboratory. Sites will also be supplied with pre-paid blood boxes.
The Trans-ST03 for the lapatinib comparison, MRI and PET/CT sub-studies collection of blood and tissue samples is funded by the RMH. ST03 pathology departments will receive a payment of £30 per patient sample (£20 for the resection & £10 for the biopsy) that is submitted to the RMH pathology laboratory. Sites will also be supplied with pre-paid blood boxes.

32.4 Centres participating in the MRI sub-study

It is expected that the resection specimen will be worked up for the MRI sub-study as described in the ST03 Guidance Booklet for Pathologists and that high resolution macroscopic images of individual cross-sections are taken at time of cut up. These images should be sent to the pathology lab in Leeds, preferably as jpg files on a CD/DVD labelled with the patient's ST03 ID Number, initials and pathology specimen number, together with the HE stained slides for the central pathology review.

If your centre is unable to fully comply with the pathology requirements as described above, you may still recruit patients to the MRI sub-study, please contact the ST03 trial manager for advice if this is the case.

32.5 Summary of the Destination of Pathology Research Samples in the ST03 Trial:

For patients randomised to Bevacizumab comparison (Arms A and B) –

- Trans-ST03 Sample Collection: Send all Trans-ST03 samples to Leeds.
  (If the patient was registered for HER-2 testing at the RMH, the RMH will send biopsy blocks to Leeds provided patient has consented to Trans-ST03)
- Central Pathology Review: Send all slides to Leeds

For patients randomised to Lapatinib comparison (Arms C and D)

- Trans-ST03 Sample Collection: Send all Trans-ST03 samples to RMH.
  (If the patient was registered for HER-2 testing at the RMH, the RMH will retain the biopsy blocks provided patient has consented to Trans-ST03)
- Central Pathology Review: Send all slides to Leeds

For patients registered into the MRI and/or PET/CT sub-studies only

- Trans-ST03 Sample Collection: Send all Trans-ST03 Samples to RMH
  (If the patient was registered for HER-2 testing at the RMH, the RMH will retain the biopsy blocks provided patient has consented to Trans-ST03)

- MRI sub-study only: Send resection specimen images to Leeds.
- MRI sub-sudy only: Central Pathology - send HE biopsy and resection slides to Leeds

NB. If the patient is participating in the PET/CT sub-study only, HE slides do not need to be sent to Leeds for central pathology review

33. Publication Policy

The results from different centres will be analysed together and published as soon as possible. Individual clinicians must not publish data concerning their patients that are directly relevant to questions addressed in the study until the TMG has published its report. The TMG together with the ST03 collaborators will form the basis of the writing committee and decide on the nature of the publications. All publications will acknowledge the participating centres, and these will be detailed in an appendix to the main report.

34. Protocol Amendments

Please check with the MRC CTU ST03 Trial Manager that you are using the most recent version of the ST03 protocol. For full details of the amendments submitted to the Sunderland MREC by the MRC CTU, please refer to your site's ST03 Master File Documents

ST03 Protocol Version 1, 10th October 2006
Approved by MREC on 7th January 2007

ST03 Protocol Version 2, 14th March 2008
Approved by MREC on 23rd April 2008
The main changes concerned the additional of the Translation substudy, additional funding available as well as further clarification to the protocol

- Patient Inclusion Criteria has been updated
- Patient Exclusion Criteria: patients with clinically apparent hearing impairment has been removed from the exclusion criteria.
- Warfarin has been removed as an excluded concomitant medication, but is advised that it should be used with caution.
- Guidance is given for patients with raised BP and hearing impairment.
- Tissue and Blood Collection for Translational Research has been added
- Capecitabine Diary Card has been added
- Cisplatin and Capecitabine dose modifications for ECX has been updated to be more reader friendly.
- If cisplatin is substituted for carboplatin, patients should continue to receive epirubicin.
- Instructions on all patients receiving lansoprazole 30mg od po, or an alternative proton pump inhibitor (PPI), is given.
- New guidance has been added for patients that remain unfit to commence post-operative chemotherapy, 10 weeks post surgery
- The intervals for the initial follow-up assessments have been corrected, this is also reflected in the ST03 Trial schema diagram and trial summary
- Specific toxicities requiring discontinuation of bevacizumab. Patients with any grade of fistulae should have bevacizumab discontinued
- Recommendations for managing patients that develop fistula are given.
- All grades of fistulae has been added as an ST03 notable event
- Sites will receive additional funding via per patient payments for the first 100 patients randomised
- The central pathology review has been updated and new information has been added to describe the collection of blood and tumour samples for future translational research
- Patient Information Sheet has been updated
- ST03 Trial Consent Form has been updated

- Trans ST03 Consent Form has been added

ST03 Protocol Version 3, 17th June 2009
Approved by MREC on 21st July 2009

The main changes concern the additional of the Type II junctional tumours to the inclusion criteria as well as further clarification to the protocol. Full details of the protocol changes are given in the summary of amendments to Version 3, however, the key changes include:

- Type II tumours now eligible for the trial
- 24 hour urine collection is no longer mandatory at baseline
- Patients with high frequency hearing loss are eligible for ST03, they should be treated with cisplatin but changed to carboplatin if there is any evidence of deterioration
- Patients with severe tinnitus should not be randomised
- Positive serology for HIV, Hepatitis C of active Hepatitis B have been added as exclusion criteria
- Some flexibility with screening assessments and investigations have been added
- Updated information on proteinuria to include use of urine protein-creatinine ratio
- New guidelines provided to include surgical techniques for Type II junctional tumours and information on performing totally minimally invasive surgery
- New table added to show CRF completion schedule for patients who do not complete protocol treatment
- Further guidance and clarification on various elements of the central pathology review and storage of translational ST03 samples
- SPC updated where necessary

ST03 Protocol Version 3 – Addendum 1 Urgent Safety Amendment, Feb 2010

A nadir blood count is now required during cycle 1 at approximately day 10 of treatment. Any patients who experience Grade 4 neutropenia should receive GCSF for 3-5 days starting on day 10. Any patients who have Grade 3 neutropenia should be treated with GCSF at the discretion of the Investigator. These patients should also receive GSCF during all further cycles of ECX+/-B.

ST03 Protocol Version 4, 27th May 2010
Approved by MREC on 2nd July 2010

The main changes concern the addition of a nadir blood count during cycle one and guidance for commencing GCSF, changes to the cardiac monitoring recommendations and TNM7 staging clarification.

Section 6.1 Patient Inclusion Criteria

Guidance for any patients staged according to the TNM7 staging criteria

Cardiac function measured by MUGA can be above centre's LLN if this is lower than 50%

Patients who have received chemotherapy or radiotherapy for a previous malignancy are ineligible

Section 8.1 Treatment of Patients

A nadir blood count is now required during cycle 1 at approximately day 10 of treatment and any patients who experience Grade 4 should receive GCSF for 3-5 days starting on day 10. Any patients who have Grade 3 neutropenia should be treated with GCSF at the discretion of the Investigator. These patients should also receive GSCF during all further cycles of ECX+/-B.

Section 8.1.2 Cardiac Monitoring

Patients in whom a drop in LVEF of $\geq$15% or to less than 50% is seen should have a further measurement taken 3 months later

Section 8.4.2 Guidance on omitting doses of Bevacizumab

Bevacizumab may be reintroduced if it was ceased following a reduction in LVEF if recovery to the normal range is observed at the next cardiac monitoring scan.

Section 9.2.6 Hypertension

An ACE inhibitor should be used as first line to treat hypertension.

Section 11.2 Management of LVEF
Where a range is reported, management should be based on the lower bound of the range.
Any patients with a reduction in LVEF of ≥15% or to <50% should have an additional scan 3 months after the abnormal measurement. Epirubicin and/or Bevacizumab may be restarted if the LVEF recovers to >50%.

Appendix M: TNM 7th edition staging criteria
Conversion table added for any patients staged using TNM7.

Appendix N: Algorithm for cardiac monitoring requirements
Flow diagram added to provide guidance on cardiac monitoring recommendations.

ST03 Protocol Version 5, 15th Feb 2011
Approved by MREC on 23rd March 2011

The main changes concern the inclusion of lower oesophageal and Type I tumours and associated updated staging, screening, surgery and pathology guidance, changes to the hypertension recommendations and ECG requirements, guidance on treatment recommendations for patients with symptomatic Pulmonary Embolisms and addition of the MRI Sub-study.

Title Updated: A Randomised Phase II/III trial of Peri-operative chemotherapy with or without Bevacizumab in Operable Oesophagogastric Adenocarcinoma.

Section 6.1 Patient Inclusion Criteria
Both lower oesophageal and Siewert Type I OGJ adenocarcinomas are now eligible for the trial
Inclusion criteria and staging information has been updated to reflect this (please see section 6 for more information)
All patients should have a CT, patients with gastric or Type II or III OGJ tumours should also have a laparoscopy and patients with lower oesophageal or OGJ tumours should also have an EUS. Table 11.1 has been updated with the revised ST03 trial assessment requirements and updated TNM 6/7 conversion tables have been included in Appendix M.

Section 6.2 Patient Exclusion Criteria
Uncontrolled hypertension is defined as BP ≥140/90mmHg.
Patients with a BP ≥140/90mmHg prior to randomisation should be commenced on an ACE inhibitor or other antihypertensive agent then BP re-checked a few days later, if BP is controlled to <140/90mmHg then the patient may be entered into the trial.
Patients with an oesophageal or gastric stent (metal or biodegradable) in situ are ineligible for the study.

Section 6.3.2 Medications to be used with caution
Possibility that previous or concomitant treatment with bisphosphonates in patients receiving bevacizumab may increase the risk of oestonecrosis of the jaw (ONJ). Patients on ECX+B who have received previous bisphosphonate therapy should have a dental examination prior to commencing treatment and should be encouraged to report any symptoms of pain in the mouth, teeth or jaw, numbness or heaviness in the jaw or looseness of a tooth.

Section 8.1.1 Nadir Blood Count
Patients who experience Grade 3 or 4 neutropenia on day 1 of subsequent cycles dose reduction recommendations within section 8.3.3 should be followed and GCSF can be administered as per local practice.

Section 8.3.8 Plantar-Palmar Erytehma (PPE)
Recommendation to treat PPE with Pyridoxine has been removed (see section 8.3.8 for updated recommendations)

Section 9 General information on adverse events associated with bevacizumab
Overall safety profile of bevacizumab has been updated in line with most recent information.
Bevacizumab should be permanently ceased in patients developing congestive cardiac failure, myocardial infarction or cerebrovascular disease. Patients diagnosed with new ischaemic heart disease during the study should also permanently cease bevacizumab.
Treatment recommendations for a patient with a symptomatic Pulmonary Embolism (grade 4 venous thromboembolic event) have been updated. Bevacizumab should be stopped upon

diagnosis but can be resumed again once the patient has been anti-coagulated and has become asymptomatic from the Pulmonary Embolism (see section 9.2.5 for more information)
Treatment recommendations for hypertension have also been updated in section 9.2.6 hypertension should be controlled to <140/90mmHg prior to receiving treatment (see section 9.2.6 and Appendix E for more information).

Section 10 Surgery
The surgery section has been updated with information relevant for the increased inclusion criteria and the MRI sub-study.

Section 11.1 Assessments required prior to each cycle of treatment with ECX +/- bevacizumab
The requirement for an ECG prior to each cycle of chemotherapy has been removed. Patients are only requested to have an ECG at baseline to confirm eligibility and before surgery.

Section 11.9 Archiving
Archiving instructions have been added.

Appendix Q: MRI Sub-study
Details of the MRI sub-study have been added at Appendix Q and at relevant places throughout the protocol. Only a small number of selected centres will be participating in the MRI Sub-study, if your centre is not participating then please ignore the instructions relating to the sub-study.

ST03 Protocol Version 5 – Addendum 1, Urgent Safety Amendment July 2011

The additional cardiac monitoring assessments (pre-op and post chemo ECHO/MUGAs) have been discontinued from the sECX+/- bevacizumab arms (Arms A and B) of the study following a safety analysis. Study visit schedules and CRF return schedules have also been updated to reflect this.

ST03 Protocol Version 6, 28th Sept 2012
Approved by MREC 22nd October 2012

The main changes concern the addition of a lapatinib feasibility study within the ST03 trial. Selected centres will participate in this feasibility trial which will involve testing the HER-2 status of the patient's tumour biopsy centrally at the Royal Marsden Hospital (or locally if permitted) prior to randomisation into the trial. Patients with HER-2 positive tumours will be randomised to sECX Vs mECX+Lapatinib and patients with HER-2 negative or unknown/undefined tumours will be randomised to sECX+/-Bevacizumab. This is in response to newly published data in the advanced setting (73). Section B has been added to the protocol to include instructions for the selected centres involved in the feasibility study.

Section A provides information for centres not involved in the feasibility study and the patients with HER-2 negative or unknown/undefined tumours randomised into the sECX+/-Bevacizumab arms.
Section B provides information for the centres involved in the feasibility study on the registration of patients and the HER-2 sample testing process and then goes on to provide details for the management of patients with HER-2 positive tumours randomised to the sECX Vs mECX+Lapatinib arms.

Title Updated: A Randomised Phase II/III trial of Peri-operative Chemotherapy with or without Bevacizumab in Operable Oesophagogastric Adenocarcinoma and (in selected centres) A Feasibility Study Evaluating Lapatinib in HER-2 Positive Oesophagogastric Adenocarcinomas

Section A1.2 and B1.3 Patient Exclusion criteria
Exclusion criteria added. Patients with a history of interstitial lung disease or radiological evidence of lung fibrosis are ineligible for this study.

Section A3.1: Nadir Blood Test
This section has been updated to emphasise that nadir blood tests in cycle 1 are mandatory for all patients.

Section A: Cardiac Monitoring within the sECX+/-B comparison
The additional cardiac monitoring assessments (pre-op and post chemo ECHO/MUGAs) have been discontinued from the sECX+/- bevacizumab arms (Arms A and B) of the study following a

safety analysis. Study visit schedules and CRF return schedules have also been updated to reflect this.

Section B: Lapatinib Feasibility Study
New section added to the protocol for use by the centres participating in the feasibility study only. Information provided on the registration and HER-2 testing of patients and randomisation and treatment instruction for patients with HER-2 positive tumours to be entered into the sECX Vs mECX + Lapatinib arms.

Section 6.5 ST03 Additional Notable Events that Require Expedited Reporting for sECX Vs mECX + Lapatinib
Details of the notable events that should be reported on an SAE form for patients in the sECX Vs mECX+L arm have been added (applies to selected centres only)

Section 21.2 Trans-ST03
Information added on destination of Trans-ST03 samples for those patients who are registered for HER-2 testing, relevant to lapatinib feasibility centres only.

Appendix R: Capecitabine and Lapatinib Modifications and Dose Levels
Details of the capecitabine and lapatinib modifications and dose levels in the feasibility study have been added.

Appendix S: Concomitant Medications
Guidance on concomitant medications in sECX+/-B arms and sECX Vs mECX+L have been added.

Appendix U: FDG PET Sub-study
Details of the FDG PET/CT sub-study have been added at Appendix U and at relevant places throughout the protocol. Only a small number of selected centres will be participating in the FDG PET/CT sub-study, if your centre is not participating then please ignore the instructions relating to the sub-study.

ST03 Protocol Version 6 – Addendum 1, Urgent Safety Amendment October 2013
Inclusion criteria for the ST03 main trial (sECX+/-bevacizumab comparison) amended to exclude patients with lower oesophageal, Siewert Type I, II or III oesophagogastric junctional (OGJ) adenocarcinomas from entering this comparison. The trial remains open and unchanged for patients with gastric adenocarcinomas who do not require oesophagogastrectomy.

Patients with lower oesophageal, Siewert Type I, II or III OGJ adenocarcinomas who have already entered the sECX+B arm (Arm B) must not receive any further pre-operative bevacizumab. Following surgery patients may resume treatment with bevacizumab provided that there are no other contraindications.

ST03 Protocol Version 7, 17th February 2014
The main changes to the protocol are organisational and have been made to reflect the closure of the bevacizumab comparison and to allow patients with a negative or unknown/undefined HER-2 status to be registered into the observation only, imaging sub-studies (MRI and PET/CT) at participating centres.
The following information regarding the bevacizumab comparison (now closed to recruitment) has been moved to **Appendix T**:

Trial Summary
Background and Rationale
Trial Design and Objectives
Selection of Patients
Randomisation
Treatment of Patients (including dose modifications for toxicities)
General Information on adverse events associated witrh Bevacizumab
Assessment and Procedures within the sECX+/- Bevacizumab comparison
Statistical Considerations for the Bevacizumab comparison

All other information applicable to all patients in the trial remains in section 16 onwards

The MRI sub-study protocol previously found in Appendix T has been integrated into the main body of the protocol at relevant sections with the majority of procedural information found in section 14.

The PET/CT sub-study protocol previously found in Appendix U has been integrated into the main body of the protocol at relevant sections with the majority of procedural information found in section 15.

Throughout the protocol references to the managaing organisation have been updated following MRC CTU becoming part of University College London on 1st August 2013.

Section 1.1 Type of Design
Details regarding the data that resulted in the urgent safety amendment Oct 2013 have been added.

Section 6.1 Inclusion Criteria for patients with HER-2 Positive Tumours Entering the Lapatinib Comparison
Inclusion criteria added. Normal corrected QTc (corrected for heart rate)

Section 6.2 Exclusion Criteria for patients with HER-2 Positive Tumours Entering the Lapatinib Comparison
Exclusion criterion removed:
Definition of uncontrolled hypertension, >140/90.
Known allergy to Chinese hamster ovary cell proteins or other recombinant human or humanized antibodies or to any excipients of bevacizumab formulation, platinum compounds or to any other components of the study drugs.
Due to an increase in perforations associated with self-expandable metal stents in patients with colorectal cancer receiving bevacizumab, patients with an oesophageal or gastric stent (metal or biodegradable) in situ are ineligible for the study.

Section 8.3.4 Cardiac Toxicity
Information added regarding caution to be taken with patients taking lapatinib with QTc prolongation.

Section 10.1 Inclusion criteria for Patients to be Registered into the MRI and/or PET/CT sub-studies ONLY
Eligibility criteria changed. Patients with gastric tumours are now eligible for the PET/CT sub-study

Section 14.3 MRI Sequence
MRI sequence updated to allow assessment of the difference between apparent diffusion coefficient values and pathological response which is now a secondary outcome measure.

Section 17 Safety Reporting
Clarification added that SAEs will not be required to be reported for patients who are registered only to the MRI and/or PET CT sub-studies.

Section 25 Criteria for Stopping the Trial
Information on urgent safety measure October 2013 has been included.

Appendix T ST03 Original Trial Bevacizumab Comparison (Now Complete)
Appendix added to contain the information relating to the bevacizumab comparison. Includes new information in relation to the urgent safety amendment.

35. References

1. Cunningham D, Allum W, Stenning S, et al: Perioperative Chemotherapy versus Surgery Alone for Resectable Gastroesophageal Cancer: A Randomised, controlled trial (MAGIC trial, ISRCTN 93793971). N Engl J Med 355:11-20, 2006

2. Boige V, Pignon J, Saint-Aubert B, et al. Final results of a randomized trial comparing preoperative 5-fluorouracil (F)/cisplatin (P) to surgery alone in adenocarcinoma of stomach and lower esophagus (ASLE): FNLCC ACCORD07-FFCD 9703 trial. J Clin Oncol 25: 18s 2007 (abstr 4510).

3. Twelves C, Boyer M, Findlay M, et al: Capecitabine (Xeloda) improves medical resource use compared with 5-fluorouracil plus leucovorin in a phase III trial conducted in patients with advanced colorectal carcinoma. Eur J Cancer 37:597-604, 2001

4. NICE Technology Appraisal Guidance 61: Guidance on the use of capecitabine and tegafur with uracil for metastatic colorectal cancer. London: NICE, 2003

5. Cassidy J, Twelves C, Van Cutsem E, et al: First-line oral capecitabine therapy in metastatic colorectal cancer: a favorable safety profile compared with intravenous 5-fluorouracil/leucovorin. Ann Oncol 13:566-75, 2002

6. Cunningham D, Rao S, Starling N, Iveson T, Nicolson M, Coxon F, et al. Randomised multicentre phase III study comparing capecitabine with fluorouracil and oxaliplatin with cisplatin in patients with advanced oesophagogastric (OG) cancer: The REAL 2 trial. J Clin Oncol 2006;24(No18s (June 20 suppl)):abstr LBA 4017

7. Evans TR, Pentheroudakis G, Paul J, et al: A phase I and pharmacokinetic study of capecitabine in combination with epirubicin and cisplatin in patients with inoperable oesophago-gastric adenocarcinoma. Ann Oncol 13:1469-78, 2002

8. Galizia G, Lieto E, Orditura M, et al: Epidermal growth factor receptor (EGFR) expression is associated with a worse prognosis in gastric cancer patients undergoing curative surgery. World J Surg 31:1458-68, 2007

9. Lieto E, Ferraraccio F, Orditura M, et al: Expression of vascular endothelial growth factor (VEGF) and epidermal growth factor receptor (EGFR) is an independent prognostic indicator of worse outcome in gastric cancer patients. Ann Surg Oncol 15:69-79, 2008

10. Wang KL, Wu TT, Choi IS, et al: Expression of epidermal growth factor receptor in esophageal and esophagogastric junction adenocarcinomas: association with poor outcome. Cancer 109:658-67, 2007

11. Douillard JY, Siena S, Cassidy J, et al: Randomized, Phase III Trial of Panitumumab With Infusional Fluorouracil, Leucovorin, and Oxaliplatin (FOLFOX4) Versus FOLFOX4 Alone As First-Line Treatment in Patients With Previously Untreated Metastatic Colorectal Cancer: The PRIME Study. J Clin Oncol, 2010

12. Van Cutsem E, Kohne CH, Hitre E, et al: Cetuximab and chemotherapy as initial treatment for metastatic colorectal cancer. N Engl J Med 360:1408-17, 2009

13. Bokemeyer C, Bondarenko I, Makhson A, et al: Fluorouracil, leucovorin, and oxaliplatin with and without cetuximab in the first-line treatment of metastatic colorectal cancer. J Clin Oncol 27:663-71, 2009

14. Peeters M, Price TJ, Cervantes A, et al: Randomized Phase III Study of Panitumumab With Fluorouracil, Leucovorin, and Irinotecan (FOLFIRI) Compared With FOLFIRI Alone As Second-Line Treatment in Patients With Metastatic Colorectal Cancer. J Clin Oncol, 2010

15. Jonker DJ, O'Callaghan CJ, Karapetis CS, et al: Cetuximab for the treatment of colorectal cancer. N Engl J Med 357:2040-8, 2007

16. Amado RG, Wolf M, Peeters M, et al: Wild-type KRAS is required for panitumumab efficacy in patients with metastatic colorectal cancer. J Clin Oncol 26:1626-34, 2008

17. Karapetis CS, Khambata-Ford S, Jonker DJ, et al: K-ras mutations and benefit from cetuximab in advanced colorectal cancer. N Engl J Med 359:1757-65, 2008

18. De Roock W, Claes B, Bernasconi D, et al: Effects of KRAS, BRAF, NRAS, and PIK3CA mutations on the efficacy of cetuximab plus chemotherapy in chemotherapy-refractory metastatic colorectal cancer: a retrospective consortium analysis. Lancet Oncol 11:753-62, 2010

19. Cunningham D, Humblet Y, Siena S, et al: Cetuximab monotherapy and cetuximab plus irinotecan in irinotecan-refractory metastatic colorectal cancer. N Engl J Med 351:337-45, 2004

20. Bonner JA, Harari PM, Giralt J, et al: Radiotherapy plus cetuximab for squamous-cell carcinoma of the head and neck. N Engl J Med 354:567-78, 2006

21. Lynch TJ, Bell DW, Sordella R, et al: Activating mutations in the epidermal growth factor receptor underlying responsiveness of non-small-cell lung cancer to gefitinib. N Engl J Med 350:2129-39, 2004

22. Tsao MS, Sakurada A, Cutz JC, et al: Erlotinib in lung cancer - molecular and clinical predictors of outcome. N Engl J Med 353:133-44, 2005

23. Moore MJ, Goldstein D, Hamm J, et al: Erlotinib plus gemcitabine compared with gemcitabine alone in patients with advanced pancreatic cancer: a phase III trial of the National Cancer Institute of Canada Clinical Trials Group. J Clin Oncol 25:1960-6, 2007

24. Lordick F, Luber B, Lorenzen S, et al: Cetuximab plus oxaliplatin/leucovorin/5-fluorouracil in first-line metastatic gastric cancer: a phase II study of the Arbeitsgemeinschaft Internistische Onkologie (AIO). Br J Cancer, 2010

25. Pinto C, Di Fabio F, Barone C, et al: Phase II study of cetuximab in combination with cisplatin and docetaxel in patients with untreated advanced gastric or gastro-oesophageal junction adenocarcinoma (DOCETUX study). Br J Cancer 101:1261-8, 2009

26. Pinto C, Di Fabio F, Siena S, et al: Phase II study of cetuximab in combination with FOLFIRI in patients with untreated advanced gastric or gastroesophageal junction adenocarcinoma (FOLCETUX study). Ann Oncol 18:510-7, 2007

27. Adelstein DJ RL, Carroll MA, Rice TW, Mekhail T: Phase II trial of gefitinib for recurrent or metastatic esophageal or gastroesophageal junction (GEJ) cancer., ASCO Annual Meeting, Journal of Clinical Oncology, 2005 ASCO Annual Meeting Proceedings. , 2005, pp 4054

28. Ferry DR, Anderson M, Beddard K, et al: A phase II study of gefitinib monotherapy in advanced esophageal adenocarcinoma: evidence of gene expression, cellular, and clinical response. Clin Cancer Res 13:5869-75, 2007

29. Janmaat ML, Gallegos-Ruiz MI, Rodriguez JA, et al: Predictive factors for outcome in a phase II study of gefitinib in second-line treatment of advanced esophageal cancer patients. J Clin Oncol 24:1612-9, 2006

30. Yamamoto T, Ikawa S, Akiyama T, et al: Similarity of protein encoded by the human c-erb-B-2 gene to epidermal growth factor receptor. Nature 319:230-4, 1986

31. Yokota J, Yamamoto T, Toyoshima K, et al: Amplification of c-erbB-2 oncogene in human adenocarcinomas in vivo. Lancet 1:765-7, 1986

32. Matsui Y, Inomata M, Tojigamori M, et al: Suppression of tumor growth in human gastric cancer with HER2 overexpression by an anti-HER2 antibody in a murine model. Int J Oncol 27:681-5, 2005

33. Tanner M, Hollmen M, Junttila TT, et al: Amplification of HER-2 in gastric carcinoma: association with Topoisomerase IIalpha gene amplification, intestinal type, poor prognosis and sensitivity to trastuzumab. Ann Oncol 16:273-8, 2005

34. Fujimoto-Ouchi K, Sekiguchi F, Yasuno H, et al: Antitumor activity of trastuzumab in combination with chemotherapy in human gastric cancer xenograft models. Cancer Chemother Pharmacol 59:795-805, 2007

35. Kim JW, Kim HP, Im SA, et al: The growth inhibitory effect of lapatinib, a dual inhibitor of EGFR and HER2 tyrosine kinase, in gastric cancer cell lines. Cancer Lett 272:296-306, 2008

36. Slamon DJ, Godolphin W, Jones LA, et al: Studies of the HER-2/neu proto-oncogene in human breast and ovarian cancer. Science 244:707-12, 1989

37. Allgayer H, Babic R, Gruetzner KU, et al: c-erbB-2 is of independent prognostic relevance in gastric cancer and is associated with the expression of tumor-associated protease systems. J Clin Oncol 18:2201-9, 2000

38. Grabsch H, Sivakumar S, Gray S, et al: HER2 expression in gastric cancer: Rare, heterogeneous and of no prognostic value - conclusions from 924 cases of two independent series. Cell Oncol 32:57-65, 2010

39. Lee HR, Kim JH, Uhm HD, et al: Overexpression of c-ErbB-2 protein in gastric cancer by immunohistochemical stain. Oncology 53:192-7, 1996

40. Mizutani T, Onda M, Tokunaga A, et al: Relationship of C-erbB-2 protein expression and gene amplification to invasion and metastasis in human gastric cancer. Cancer 72:2083-8, 1993

41. Motojima K, Furui J, Kohara N, et al: erbB-2 expression in well-differentiated adenocarcinoma of the stomach predicts shorter survival after curative resection. Surgery 115:349-54, 1994

42. Nakajima M, Sawada H, Yamada Y, et al: The prognostic significance of amplification and overexpression of c-met and c-erb B-2 in human gastric carcinomas. Cancer 85:1894-902, 1999

43. Ohguri T, Sato Y, Koizumi W, et al: An immunohistochemical study of c-erbB-2 protein in gastric carcinomas and lymph-node metastases: is the c-erbB-2 protein really a prognostic indicator? Int J Cancer 53:75-9, 1993

44. Pinto-de-Sousa J, David L, Almeida R, et al: c-erb B-2 expression is associated with tumor location and venous invasion and influences survival of patients with gastric carcinoma. Int J Surg Pathol 10:247-56, 2002

45. Tateishi M, Toda T, Minamisono Y, et al: Clinicopathological significance of c-erbB-2 protein expression in human gastric carcinoma. J Surg Oncol 49:209-12, 1992

46. Yonemura Y, Ninomiya I, Yamaguchi A, et al: Evaluation of immunoreactivity for erbB-2 protein as a marker of poor short term prognosis in gastric cancer. Cancer Res 51:1034-8, 1991

47. Slamon DJ, Leyland-Jones B, Shak S, et al: Use of chemotherapy plus a monoclonal antibody against HER2 for metastatic breast cancer that overexpresses HER2. N Engl J Med 344:783-92, 2001

48. Smith I, Procter M, Gelber RD, et al: 2-year follow-up of trastuzumab after adjuvant chemotherapy in HER2-positive breast cancer: a randomised controlled trial. Lancet 369:29-36, 2007

49. Bang YJ, Van Cutsem E, Feyereislova A, et al: Trastuzumab in combination with chemotherapy versus chemotherapy alone for treatment of HER2-positive advanced gastric or gastro-oesophageal junction cancer (ToGA): a phase 3, open-label, randomised controlled trial. Lancet 376:687-97, 2010

50. Geyer CE, Forster J, Lindquist D, et al: Lapatinib plus capecitabine for HER2-positive advanced breast cancer. N Engl J Med 355:2733-43, 2006

51. Cameron D, Casey M, Press M, et al: A phase III randomized comparison of lapatinib plus capecitabine versus capecitabine alone in women with advanced breast cancer that has progressed on trastuzumab: updated efficacy and biomarker analyses. Breast Cancer Res Treat 112:533-43, 2008

52. Blackwell KL, Burstein HJ, Storniolo AM, et al: Randomized study of Lapatinib alone or in combination with trastuzumab in women with ErbB2-positive, trastuzumab-refractory metastatic breast cancer. J Clin Oncol 28:1124-30, 2010

53. Johnston S, Pippen J, Jr., Pivot X, et al: Lapatinib combined with letrozole versus letrozole and placebo as first-line therapy for postmenopausal hormone receptor-positive metastatic breast cancer. J Clin Oncol 27:5538-46, 2009

54. Di Leo A, Gomez HL, Aziz Z, et al: Phase III, double-blind, randomized study comparing lapatinib plus paclitaxel with placebo plus paclitaxel as first-line treatment for metastatic breast cancer. J Clin Oncol 26:5544-52, 2008

55. Boussen H, Cristofanilli M, Zaks T, et al: Phase II study to evaluate the efficacy and safety of neoadjuvant lapatinib plus paclitaxel in patients with inflammatory breast cancer. J Clin Oncol 28:3248-55, 2010

56. von Minckwitz G, Eidtmann H, Loibl S, et al: Integrating bevacizumab, everolimus, and lapatinib into current neoadjuvant chemotherapy regimen for primary breast cancer. Safety results of the GeparQuinto trial. Ann Oncol

57. Baselga J, Bradbury I, Eidtmann H, et al. Lapatinib with trastuzumab for HER2-positive early breast cancer (NeoALTTO): a randomised, open-label, multicentre, phase 3 trial. Lancet. 2012 Feb 18; 379 (9816): 633-40. Equb 2012 Jan 17.

58. Iqbal S GB, Lenz HJ, Fenoglio-Preiser CM, Blanke CD: S0413: A phase II SWOG study of GW572016 (lapatinib) as first line therapy in patients (pts) with advanced or metastatic gastric cancer, ASCO Annual Meeting. Chicago, Journal of Clinical Oncology, 2007 ASCO Annual Meeting Proceedings Part I. , 2007, pp 4621

59. J. R. Hecht SGU, M. Koehler, C. Ellis, R. Gagnon, A. Kemner, M. Versola, N. Spector, M. F. Press, D. J. Richel Lapatinib monotherapy in recurrent upper gastrointestinal malignancy: Phase II efficacy and biomarker analyses, Gastrointestinal Cancers Symposium, 2008, pp Abstract number 43

60. Pishvaian M, Sakaeva D, Hsieh RK, A global mutlicentre phase II trial of lapatinib plus capecitabine in gastric cancer. J Clin Oncol 2011. 29 (suppl 4; abstr 88)

61. Hecht J BY, Sobrero A, Elme A, Patel G, Park J, Kemner A, Afenjar K, Koehler M: A phase III study of CapeOx +/- lapatinib in HER2 positive locally-advanced/metastatic upper gastrointestinal adenocarcinoma: interim safety results, Joint ECCO 15 - 34TH ESMO Multidisciplinary Congress. Berlin, European Journal of Cancer Supplements, 2009, pp Page 385

62. Satoh T BY, Wang J, Xu J, Chung HC, Yeh K, Chen J, Mukaiyama A, Yoshida P, Ohtsu A: Interim safety analysis from TYTAN: A phase III Asian study of lapatinib in combination with paclitaxel as second-line therapy in gastric cancer, ASCO Annual Meeting. Chicago, J Clin Oncol 2010, pp Abstr 4057

63. Joensuu H, Kellokumpu-Lehtinen PL, Bono P, et al: Adjuvant docetaxel or vinorelbine with or without trastuzumab for breast cancer. N Engl J Med 354:809-20, 2006

64. Allum WH, Stenning SP, Bancewicz J, Clark PI, Langley RE: Long-term results of a randomized trial of surgery with or without preoperative chemotherapy in esophageal cancer. J Clin Oncol 2009, 27:5062-7.

65. Kelsen DP, Winter KA, Gunderson LL, Mortimer J, Estes NC, Haller DG, Ajani JA, Kocha W, Minsky BD, Roth JA, Willett CG; Long-term results of RTOG trial 8911 (USA Intergroup 113): a random assignment trial comparison of chemotherapy followed by surgery compared with surgery alone for esophageal cancer. J Clin Oncol 2007, 25:3719-25.

66. Sujendran V, Wheeler J, Baron R, Warren BF, Maynard N: Effect of neoadjuvant chemotherapy on circumferential margin positivity and its impact on prognosis in patients with resectable oesophageal cancer. Br J Surgery 2008 95:191-4.

67. Cancer Research UK's Strategic Workshop on Oesphageal Cancer 4th-5th March 2008, Redhill, UK

68. Gebski V, Burmeister B, Smithers BM, Foo K, Zalcberg J, Simes J; Australasian Gastro-Intestinal Trials Group. Survival benefits from neoadjuvant chemoradiotherapy or chemotherapy in oesophageal carcinoma: a meta-analysis. Lancet Oncol 2007;8:226–234.

69. Refaely Y, Krasna MJ: Multimodality therapy for esophageal cancer. Surg Clin North Am 2002, 82:729-746.

70. Krause BJ, Herrmann K, Wieder H. 18F-FDG PET and 18F-FDG PET/CT for Assessing Response to Therapy in Esophageal Cancer. J Nucl Med. 2009 May;50 Suppl 1:89S-96S

71. Westerterp M, van Westreenen HL, Reitsma JB, Hoekstra OS, Stoker J, Fockens P, Jager PL, Van Eck-Smit BL, Plukker JT, van Lanschot JJ, Sloof GW. Esophageal cancer:

CT, endoscopic US, and FDG PET for assessment of response to neoadjuvant therapy—systematic review. Radiology 2005, 236:841-851.

72. Chowdhury FU, Bradley KM, Gleeson FV. The role of 18F-FDG PET/CT in the evaluation of oesophageal carcinoma. Clin Radiol 2008 Dec;63(12):1297-309.

73. Kobe C, Dietlein M, Franklin J et al. Positron emission tomography has a high negative predictive value for progression or early relapse for patients with residual disease after first-line chemotherapy in advanced-stage Hodgkin lymphoma. Blood. 2008 Nov 15;112(10):3989-94

74. Dose Schwarz J, Bader M, Jenicke L, Hemminger G, Jänicke F, Avril N. Early Prediction of Response to Chemotherapy in Metastatic Breast Cancer Using Sequential 18F-FDG PET. J Nucl Med. 2005 Jul;46(7):1144-50

75. Kasamon YL, Wahl RL, Swinnen LJ. FDG PET and high-dose therapy for aggressive lymphomas: toward a risk-adapted strategy. Curr Opin Oncol. 2004;16:100–105

76. Suttie SA, Welch AE, Park KG. Positron emission tomography for monitoring response to neoadjuvant therapy in patients with oesophageal and gastro-oesophageal junction carcinoma. European journal of surgical oncology 35(10):1019-29, 2009 Oct.

77. Lordick F, Ott K, Krause BJ et al. PET to assess early metabolic response and to guide treatment of adenocarcinoma of the oesophagogastric junction: the MUNICON phase II trial. Lancet Oncol. 2007 Sep;8(9):797-805.

78. van Heijl M, Omloo JM, van Berge Henegouwen MI. NEOadjuvant therapy monitoring with PET and CT in Esophageal Cancer (NEOPEC-trial)BMC Med Phys. 2008 Jul 31;8:3

79. Young H, Baum R, Cremerius U, et al., Measurement of clinical and subclinical tumour response using [18F]-fluorodeoxyglucose and positron emission tomography: review and 1999 EORTC recommendations. European Organization for Research and Treatment of Cancer (EORTC) PET Study Group. Eur J Cancer. 1999 Dec;35(13):1773-82.

80. Rüschoff J, Dietel M, Baretton G, Arbogast S, Walch A, Monges G, Chenard MP, Penault-Llorca F, Nagelmeier I, Schlake W, Höfler H, Kreipe HH.HER2 diagnostics in gastric cancer-guideline validation and development of standardized immunohistochemical testing. Virchows Arch. 2010 Sep;457(3):299-307.

81. Lapatinib spc reference: http://www.medicines.org.uk/emc/medicine/20929/SPC/tyverb/

82. Japanese Research Society for Gastric Cancer. The general rules for gastric cancer surgery and pathology. Jpn J Surg 1981; 11: 127-45

83. Cunningham D, Starling N, Rao S, et al. Capecitabine and Oxaliplatin for Advanced Oesophagogastric Cancer. N Engl J Med 358:36-46, 2008

84. Aaronson NK, Ahmedzai S, Bergman B, Bullinger M, Cull A, Duez NJ, et al. The European Organization for Research and Treatment of Cancer QLQ-C30: a quality of life instrument for use in international clinical trials in oncology. J Natl Cancer Inst 1993;85:365-376.

85. Blazeby JM, Conroy T, Bottomley A, Vickery C, Arraras J, Sezer O, et

al. Clinical and psychometric validation of a questionnaire module, the EORTC QLQ-STO 22, to assess quality of life in patients with gastric cancer. Eur J Cancer 2004;40:2260-2268.

86. Vickery, C. W., Blazeby, J. M., Conroy, T., Arraras, J., Sezer, O,Koller, M. et al. Development of an EORTC disease specific quality of life module for use in patients with gastric cancer. European Journal of Cancer 37, 966-971. 2001

87. Burroughs, S. H. et al. Oesophageal and Gastric Cancer Pathology Reporting: A regional audit. J Clin Pathol 52: 435-9, 1999.

88. King, P. M, Blazeby J.M, Gupta, J, Alderson D et al. Upper gastrointestinal cancer pathology reporting: a regional audit to compare standards with minimum datasets.J Clin Pathol 57: 702-5, 2004.

89. Scottish Executive Health Department. Scottich Audit of Gastric anc Oseophageal Cancer, Edinburgh 2002
http://www.crag.scot.nhs.uk/committees/CEPS/reports/0_prelims.pdf

Appendices

Appendix A: Additional Definitions

A1: WHO Performance Status

Grade	Performance Status
0	Able to carry out all normal activity without restriction.
1	Restricted in strenuous physical activity but ambulatory and able to carry out light work.
2	Ambulatory and capable of all self-care but unable to carry out any work; up and about more than 50% of waking hours.
3	Capable of only limited self-care; confined to bed or chair more than 50% of waking hours.
4	Completely disabled; cannot carry on any self-care; totally confined to bed or chair.

A2a: TNM Staging (6th ed.): Gastric/type III OGJ Tumours

Stage Group	T Stage	N	M
**Stage 0**	Tis	N0	M0
**Stage IA**	TI	N0	M0
**Stage IB**	T1	N1	M0
	T2a/b	N0	M0
**Stage II**	T1	N2	M0
	T2a/b	N1	M0
	T3	N0	M0
**Stage IIIA**	T2a/b	N2	M0
	T3	N1	M0
	T4	N0	M0
**Stage IIIB**	T3	N2	M0
**Stage IV**	T4	N1-3	M0
	T1-3	N3	M0
	Any T	Any N	M1

A2b: TNM Staging (6th ed.): Lower oesophageal/type I-II OGJ Tumours

Stage Group	T Stage	N	M
**Stage 0**	Tis	N0	M0
**Stage I**	TI	N0	M0
**Stage IIA**	T2	N0	M0
	T3	N0	M0
**Stage IIB**	T1	N1	M0
	T2	N1	M0
**Stage III**	T3	N1	M0
	T4	Any N	M0

**Stage IV**	any T	Any N	M1
**Stage IVA**	Any T	Any N	M1a (coeliac LN)
**Stage IVB**	Any T	Any N	M1b (other distant metastasis)

A3: New York Heart Association (NYHA) Classification

A functional and therapeutic classification for prescription of physical activity for cardiac patients.

**Class I**	patients with no limitation of activities; they suffer no symptoms from ordinary activities.
**Class II:**	patients with slight, mild limitation of activity; they are comfortable with rest or with mild exertion.
**Class III**	patients with marked limitation of activity; they are comfortable only at rest.
**Class IV**	patients who should be at complete rest, confined to bed or chair; any physical activity brings on discomfort and symptoms occur at rest.

Appendix B: Patient Information Sheets

Centres participating in the lapatinib feasibility study:

ST03 Patient Information Sheet for HER-2 Testing

ST03 Patient Information Sheet B for Patients with HER-2 Positive tumours entering the Lapatinib Comparison

Centres participating in the MRI sub-study

MRI Sub-study Patient Information Sheet

Centre participating in the FDG-PET/CT sub-study

PET/CT Sub-study Patient Information Sheet

Electronic copies of the Patient Information Sheets can be obtained by email from the ST03 Trial Team.

Appendix C: Consent Forms

Centres participating in the lapatinib feasibility study:

ST03 Consent Form for HER-2 Testing

ST03 Consent Form B for Patients with HER-2 Positive Tumours entering the Lapatinib Comparison

Centres participating in the MRI sub-study

MRI Sub-study Consent Form

Centre participating in the PET/CT sub-study

PET/CT Sub-study Consent Form

All Centres

Trans-ST03 Consent Form

Electronic copies of the Patient Consent Forms can be obtained by email from ST03 Trial Team.

Appendix D: GP Letters

Centres participating in the lapatinib feasibility study:

ST03 GP Letter B for Patients with HER-2 Positive Tumours entering the Lapatinib Comparison

ST03 GP Letter for Patients Registered into the MRI sub-study

ST03 GP Letter for Patients Registered into PET/CT Sub-study

Electronic copies of the GP Letters can be obtained by email from ST03 Trial Team.

Appendix E: Guidance on Treating Hypertension

Management of hypertension should be done by a clinician experienced in managing hypertension and should take into account the management guidelines issued by NICE.

ACE inhibitors should be the first line of antihypertensive therapy unless contraindicated.

This section Includes:

- Sample Letter to the patients GP with information regarding hypertension
- Table for recording patient's blood pressure

Appendix E(i): SAMPLE GP Letter – For Patients that Develop Hypertension

This is a <u>sample letter</u> to be sent to the patient's GP if a patient develops hypertension while on ST03 treatment. It provides information for the GP regarding the recognised side effect and states that management should be done according to local practice and that there are no known interactions between any of the medications used on the study and any anti-hypertensive medications. This is so hopefully the hypertension can be managed by the GP closer to the patient's home.

To be printed on local headed paper

Dear Dr_____________

ST03 Trial: Chemotherapy with Bevacizumab or Lapatinib for Oesophagogastric Cancer

Patient:__________________ **DOB:**___________ is currently being treated on the ST03 trial and has been allocated to receive:

☐ **Control Arm A or C or MRI and/or PET sub-study Only** (peri-operative chemotherapy, epirubicin, cisplatin and capecitabine)

☐ **Research Arm B with** bevacizumab with peri-operative chemotherapy, epirubicin, cisplatin and capecitabine

☐ **Research Arm D with** lapatinib with peri-operative chemotherapy, epirubicin, cisplatin and capecitabine

Your patient has had their blood pressure measured and it has been found to be elevated. This is one of the recognised side effects of bevacizumab. It is usually easily managed with standard anti-hypertensive therapy. BP should be managed according to standard local guidelines, however we would recommend the use of an ACE inhibitor as first line and in the absence of a known contraindication, we have started perindopril at 2mg daily. There are no specific interactions known between bevacizumab, Lapatinib, or the chemotherapy (epirubicin cisplatin or capecitabine) being used in this trial and any anti-hypertensive therapies. However, please note that the calcium channel blockers verapamil and diltiazem are excluded concomitant medications with Lapatinib and are prohibited drugs on research arm D (lapatinib arm).

It would be appreciated if you could repeat and continue to monitor your patient's blood pressure, renal function and electrolytes, adjusting their medication as required according to your normal practice. The aim should be to attain a blood pressure

consistently below 140/90mmHg. We would also appreciate you, or your patient, recording their blood pressure on the patient's blood pressure sheet included.

If you have any concerns or questions, or require any further information, please contact the responsible doctor:

Dr ____________________________

at ______________________________(Hospital)

Tel: ____________________________

If the patients contacts you and is unwell or you are worried please contact the above doctor, or if out of hours then contact

____

Yours sincerely,

Name:

Job Title:

Appendix E (ii) ST03 Trial Blood Pressure Monitoring Form

DATE	BLOOD PRESSURE	THERAPY

Appendix F: Modified RECIST Version 1.0 Response Definitions

RECIST (Response Evaluation Criteria In Solid Tumours) has now superseded the old WHO response criteria for solid tumours.

The key differences are:

- instead of measuring lesions in 2 dimensions it is now only necessary to measure the longest diameter.
- disease is classified as measurable or not measurable but the term evaluable is no longer used.

Measurable disease:

- Disease is measurable if there is at least one measurable target lesion. Target lesions should be selected on the basis of size and suitability for repeat measurement, up to a maximum of 5 measurable lesions per organ, and up to a maximum of 10 lesions in total. These should be representative of all involved organs.
- Target lesion must be accurately measurable in at least 1 dimension, with the longest diameter ≥20 mm (or ≥10 mm with spiral CT scan). If the lesion is smaller than this then it is classed as non-measurable.
- Measurements must be taken as close as possible to the beginning of treatment and never more than 4 weeks before the start of treatment. Target lesions should be assessed by CT, MRI or CXR, not by clinical assessment alone. The same imaging modality should be used throughout for any given patient.
- When intra-venous contrast agents are given with CT, it is important to measure hepatic lesions in the same vascular phase on subsequent examinations.
- If MRI is used than the same sequence (e.g. T1 or T2 weighted images) in the same anatomical plane should be used.
- Add the longest diameters of the target lesions and report this as the **baseline sum longest diameter.** This will be used as a reference by which the tumour response will be measured.

Response definitions:

Complete response (CR): disappearance of all lesions (i.e. all evidence of disease, not just the target lesions) determined by 2 observations not less than 4 weeks apart (in *ST03* the pre surgery assessment should be used as the confirmatory assessment; there is no need for additional confirmatory scans.

Partial response (PR): ≥30% decrease in the sum of longest diameters of target lesions compared to baseline, with response or stable disease observed in non-target lesions, and no new lesions.

Stable disease (SD): neither sufficient shrinkage to qualify for response or sufficient increase to qualify for progressive disease in target lesions, with response or stable disease observed in non-target lesions, and no new lesions

Progressive disease (PD): ≥20% increase in the sum of longest diameters of target lesions compared to smallest sum longest diameter recorded, or unequivocal progression of non-target lesions, or appearance of new lesions.

Reminder:

Response is judged against baseline, but progression is judged against the smallest recorded score.

Example:

Month	0	3	6	9	12
Measurement (mm)	100	90	50	55	≥60
Classification	Baseline	SD	PR	PR	PD

References:

Therasse P, Arbuck SG, Eisenhauer EA, et al. New guidelines to evaluate the response to treatment in solid tumours. J Natl Cancer Inst 2000, 92, 205-216.

Gehan EA and Tefft MC. Will there be resistance to the RECIST (Response Evaluation Criteria in Solid Tumours) J Natl Cancer Inst 2000, 92, 179-181.

Appendix G: CTCAE Toxicity Criteria

NB: These are selected categories. For full list see http://ctep.cancer.gov/reporting/ctc.html

ST03 Common Toxicity Criteria based on CTCAE Version 3.0

**GRADE**						
**Short Name**	**0**	**1**	**2**	**3**	**4**	**5**
**AUDITORY/EAR**						
**Hearing (without monitoring program)**	None	-	Hearing loss not requiring hearing aid or intervention (i.e. not interfering with activities of daily living (ADL)	Hearing loss requiring hearing aid or intervention (i.e. interfering with ADL)	Profound bilateral hearing loss (>90 dB)	-
**BLOOD/BONE MARROW**						
**Haemoglobin**	WLN[1]	< LLN-10.0g/dL	8.0 - 9.9g/dL	6.5 - 7.9g/dL	<6.5g/dL	Death
**Neutrophils**		< LLN-1.5 x 10^9/L	< 1.5 – 1.0 x 10^9/L	<1.0 – 0.5 x 10^9/L	<0.5 x 10^9/L	Death
**Platelets**		< LLN-75.0 x 10^9/L	< 75.0 – 50.0 x $10^{9/L}$	<50.0 – 25.0 x 10^9/L	<25.0 x 10^9/L	Death
**ALT**	WLN[BB]	> ULN - 2.5 x ULN	2.5 - 5 x ULN	5 - 20 x ULN	> 20 x ULN	-
**AST**	WLN[BB]	> ULN - 2.5 x ULN	2.5 - 5 x ULN	5 - 20 x ULN	> 20 x ULN	-
**Bilirubin**	WLN[BB]	> ULN - 1.5 x ULN	1.5 - 3.0 x ULN	3 - 10 x ULN	> 10 x ULN	-
**CARDIAC GENERAL**						
**Cardiac Ischemia/infarction**	Normal	Asymptomatic arterial narrowing without ischaemia	Asymptomatic and testing suggesting ischaemia; stable angina	Symptomatic and testing consistent with ischemia; unstable angina; intervention indicated	Acute myocardial infarction	Death
**Hypertension**	Normal	Asymptomatic, transient (<24hrs) increase by > 20mmHg (diastolic) or to > 150/100 if previously WLN[Y]; intervention not indicated	Recurrent or persistent (>24hrs) or symptomatic increase by > 20mmHg (diastolic) or to >150/100 if previously WLN; monotherapy may be indicated	Requiring more than one drug or more intensive therapy than previously	Hypertensive crisis	Death
**Left ventricular systolic dysfunction**	Normal	Asymptomatic, resting ejection fraction (EF) <60-50%; shortening fraction (SF) <30-24%	Asymptomatic, resting EF <50-40% SF <24-15%	Symptomatic congestive heart failure (CHF) responsive to intervention. EF <40—20% SF <15%	Refractory CHF or poorly controlled; EF <20%; intervention such as ventricular assist device, ventricular reduction surgery, or heart transplant indicated	Death
COAGULATION						

[1] Within Normal Limits

**INR (International Normalized** ratio of prothrombin time)	WNL[BB]	>1 - 1.5 x ULN	>1.5 - 2 x ULN	> 2 x ULN	-	-
**PTT** (Partial Thromboplastin Time)	WNL[BB]	>1 - 1.5 x ULN	>1.5 - 2 x ULN	> 2 x ULN	-	-
**DERMATOLOGY / SKIN**						
**Alopecia**	Normal	Thinning or patchy	Complete	-	-	-
**Hand-foot**		Minimal Skin changes or dermatitis (e.g., erythema) without pain	Skin changes (e.g., peeling, blisters, bleeding, edema) or pain, not interfering with function	Ulcerative dermatitis or skin changes with pain interfering with function	-	-
**Injection site reaction / extravasation reaction**	None	Pain, itching, erythema	Pain or swelling, with inflammation or phlebitis	Ulceration or necrosis that is severe, operative intervention indicated	-	-
**Wound complication, non-infectious**	None	Incisional separation of ≤25% of wound, no deeper than superficial fascia	Incisional separation of > 25% of wound with local care, asymptomatic hernia	Symptomatic hernia without evidence of strangulation; fascial disruption/dehiscence without evisceration; primary wound closure or revision by operative intervention indicated; hospitalization or hyperbaric O_2 therapy indicated	Symptomatic hernia with evidence of strangulation; fascial disruption with evisceration; major reconstruction flap, grafting, resection or amputation indicated	Death
**GASTROINTESTINAL**						
**Diarrhoea**	None	Increase of <4 stools per day over baseline; mild increase in ostomy output compared to baseline	Increase of 4-6 stools per day over baseline; IV fluids indicated <24 hrs; moderate increase in ostomy output compared to baseline; not interfering with ADL	Increase of ≥ 7 stools per day over baseline; incontinence; IV fluids <24 hrs; hospitalisation; severe increase in ostomy output compare to baseline; interfering with ADL	Life-threatening consequences (e.g. hemodynamic collapse)	Death
**Mucositis (clinical exam) Select: -Oral cavity -Pharynx**	None	Erythema of the mucosa	Patchy ulcerations or pseudomembranes	Confluent ulcerations or pseudomembranes; bleeding with minor trauma	Tissue necrosis; Significant spontaneous bleeding; life threatening consequences	Death
**Nausea**	None	Loss of appetite without alteration in eating habits	Oral intake decreased without significant weight loss, dehydration or malnutrition; IV fluids indicated <24hrs	Inadequate oral caloric or fluid intake; IV fluids, tube feedings or total parenteral nutrition (TPN) indicated ≥24 hrs	Life-threatening consequences	Death
**Vomit**	None	1 episode in 24 hours	2-5 episodes in 24 hours; IV fluids indicated < 24hours	≥ 6 episodes in 24 hours; IV fluids, or TPN indicated ≥ 24hrs	Life- threatening consequences	Death

**Fistula, GI**	None	Asymptomatic, radiographic findings only	Symptomatic; altered GI function (e.g., altered dietary habits, diarrhoea or GI fluid loss); IV fluids indicated <24 hours	Symptomatic and severely altered GI function (e.g., altered dietary habits, diarrhoea or GI fluid loss); IV fluids, tube feedings, or TPN indicated ≥24 hours	Life threatening consequences	Death
REMARK: A fistula is defined as an abnormal communication between two body cavities, potential spaces and/or the skin. The site indicated for a fistula should be the site form which the abnormal process is believed to have originated. For example, a tracheo-oesophageal fistula arising in the context of a resected or irradiated oesophageal cancer is graded as Fistula, GI – oesophagus.						
**Perforation GI (oesophagus, Stomach, duodenum, ileum, colon)**	None	Asymptomatic, radiographic finding only	Medical intervention indicated, IV fluids indicated < 24hrs	IV fluids, tube feedings, or TPN indicated ≥ 24hrs; operative intervention indicated	Life-threatening consequences	Death
**HAEMORRHAGE/BLEEDING**						
**Haemorrhage, GI**	None	Mild, intervention not indicated	Symptomatic and medical intervention or cauterization indicated (depending on site)	Transfusion, interventional radiological, endoscopic or operative intervention indicated; radiotherapy (haemostasis of bleeding site)	Life- threatening consequences; major urgent intervention indicated	Death
Metabolic/Laboratory						
**Creatinine**	Normal	>ULN-1.5 x ULN	>1.5-3.0 x ULN	>3.0-6.0 x ULN	>6.0 x ULN	Death
**Glomerular Filtration Rate**	Normal	<75-50% LLN	<50-25% LLN	<25% LLN, chronic dialysis not indicated	Chronic dialysis or renal transplant indicated	Death
**Proteinuria**	None	1+ or 0.15-1.0g.24hrs	2+ to 3+ or 1-3.5g/24hrs	4+ or >3.5g/24hr	Nephrotic syndrome	Death
**VASCULAR**						
**Arterial vessel injury**	None	Asymptomatic diagnostic finding, intervention not indicated	Symptomatic (e.g. claudication) not interfering with ADL, repair or revision not indicated	Symptomatic interfering with ADL; repair or revision indicated	Life-threatening; disabling; evidence of end organ damage (e.g. CVA, MI, organ or limb loss)	Death
**Thrombosis/ thrombus/embolism**	None	-	Deep vein thrombosis or cardiac thrombosis; intervention (e.g., anticoagulation, lysis, filter, invasive procedure) not indicated	Deep vein thrombosis or cardiac thrombosis; intervention (e.g., anticoagulation, lysis, filter, invasive procedure) indicated	Embolic event including pulmonary embolism or life threatening thrombus	Death

Appendix H: Surface area Nomogram

Nomogram for determination of body surface area from height and weight

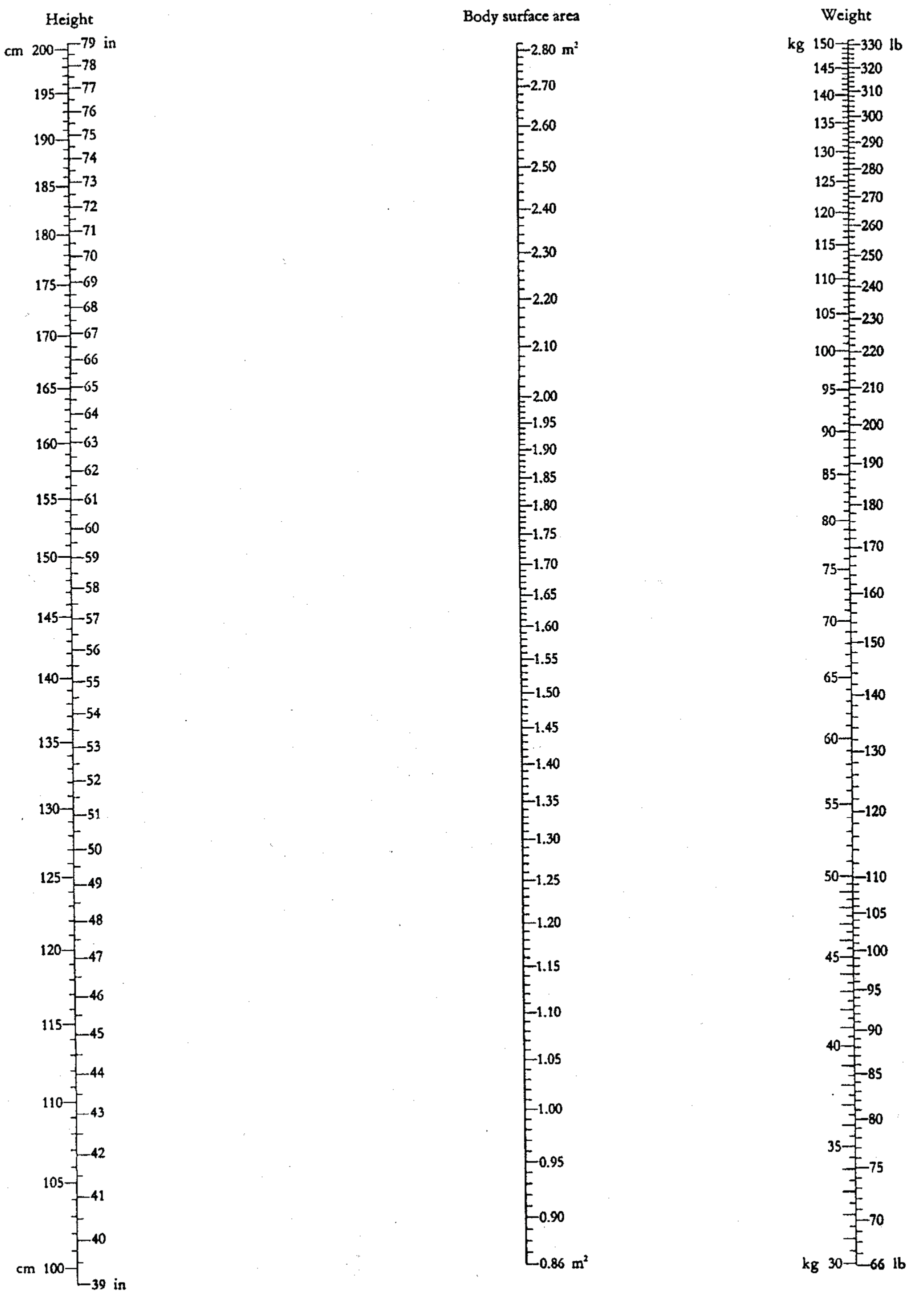

From the formula of Du Bois and Du Bois, *Arch.intern. Med.*, **17**, 863 (1916): $S = W^{0.425} \times H^{0.725} \times 71.84$, or $\log S = \log W \times 0.425 + \log H \times 0.725 + 1.8564$ (S = body surface in cm², W = weight in kg, H = height in cm)

Appendix I: Cockcroft and Gault Formula

The estimated GFR is given by:

Males: $$\frac{1.25 \times (140 - \text{age}) \times \text{weight (kg)}}{\text{serum creatinine } (\mu\text{mol/l})}$$

Females: $$\frac{1.05 \times (140 - \text{age}) \times \text{weight (kg)}}{\text{serum creatinine } (\mu\text{mol/l})}$$

- This formula usually under-estimates GFR by 10-30% compared with EDTA or measured 24-hour creatinine clearance.
- A Cockcroft/Gault estimate of ≥ 60 ml/min is accepted as evidence of adequate renal function for screening purposes.
- Patients with a Cockcroft/Gault estimate of < 60 ml/min prior to randomisation (or registration in to the MRI and/or PET/CT sub-studies) should have formal GFR measurement with EDTA or 24 hour urinary creatinine, which must be within the normal range. The uncorrected EDTA clearance should be ≥60ml/min.
- After the start of treatment, if the Cockcroft/Gault estimate falls by >25% from baseline, to below 60 ml/min, the EDTA measurement should be re-checked.

Centres may follow their standard practice for estimating creatinine clearance.

Appendix J: Drug Supply Information

Control Arm Drugs

The pharmacy department will be responsible for managing the supply of drugs in the control arm (Epirubicin, Cisplatin and Capecitabine), which are commercially available.

Bevacizumab

Recruitment to the bevacizumab comparison is now complete. Please use this section of Appendix J for information on bevacizumab for the treatment of patients within the bevacizumab comparison.

F.Hoffman-La Roche Limited has agreed to provide free bavacizumab for ST03 patients randomised to the investigational arm (sECX + bevacizumab).

- Bevacizumab is provided in a 4 ml or 16 ml vial of concentrate for solution infusion.
    - 100 mg of bevacizumab in 4 ml
    - 400 mg of bevacizumab in 16 ml

- Bevacizumab is not licensed for gastric cancer, stocks will therefore be labelled "for trial use only" before being dispatched to pharmacy.

For further details on bevacizumab please refer to the SPC which are posted on: www.medicines.org.uk

Pharmacy Information

A ST03 Pharmacy information pack including a pharmacy initiation checklist, drug accountability form, bevacizumab order form, information on storage and preparation of bevacizumab as well as procedures for drug destruction, will be sent to the lead pharmacist recorded on the signature list and delegation of responsibilities on the ST03 accreditation form.

For full details on the administration of bevacizumab please refer to Appendix T section 6.4.

The ST03 Trial Manager should have informed Roche to dispatch the initial supply of bevacizumab (for one cycle of treatment) to the pharmacy department once a centre has fulfilled the accreditation requirements (see Section 4.2 Accreditation Process).

The pharmacist will be responsible for ordering a further supply of bevacizumab for subsequent cycles of treatment. The pharmacist should regularly check the expiry date of the bevacizumab and ensure that replacement supplies are ordered from Roche via Catalent in good time to receive the shipment prior to any scheduled patient treatment. Delivery of bevacizumab can take up to 5 working days from point of order, urgent deliveries are not possible. In the event of trial stock not being available, commercial stock may be used if this is in the patient's best interest and only after obtaining approval from the ST03 Trial Manager. However, commercial stock will not be replaced or reimbursed if used. In the event that this is authorised by the ST03 Trial Manager the site must document this incident with a file note in the Pharmacy Site file and Master site File/Patient File and should complete and return the incident report documentation provided by the ST03 Trial Manager.

Randomisations (For information only: randomisation into the bevacizumab comparison is now closed)

When a new patient is randomised into the ST03 trial, the MRC CTU will notify the pharmacist (recorded on the ST03 accreditation) of the randomisation and the treatment

regimen to which the patient has been allocated and the patient's treatment schedule will be sent to the Research Nurse.

Procedures for Destruction of Bevacizumab
Returned or out of date stock should be destroyed in accordance with local policies and procedures. Destruction of bevacizumab should be documented on the 'ST03 Drug Destruction Form' for auditing and monitoring purposes and faxed to Roche.

Lapatinib

Glaxo-Smith-Kline Ltd has agreed to provide free lapatanib for ST03 patients randomised to the investigational arm (mECX +lapatinib).

Lapatinib is supplied as a film-coated tablet (tablet) which are oval, biconvex, and yellow with "GS XJG" debossed on one side.

Each film-coated tablet contains lapatinib ditosylate monohydrate, equivalent to 250 mg lapatanib.

Lapatinib is not licensed for gastric cancer, stocks will therefore be labelled "for trial use only" before being dispatched to pharmacy.

For further details on lapatanib please refer to the SPC which are posted on: www.medicines.org.uk

Pharmacy Information
A ST03 Pharmacy information pack including a pharmacy initiation checklist, drug accountability form, lapatanib order form, information on storage and preparation of lapatanib as well as procedures for drug destruction, will be sent to the lead pharmacist recorded on the signature list and delegation of responsibilities on the ST03 accreditation form.

For full details on the administration of lapatanib please refer to Section 8.4.
Please note lapatinib dose levels are subject to reduction or escalation as per Sections 8.4 and 8.5, centres will be informed at randomisation as to which dose level is currently being used.

Once a centre has fulfilled the accreditation requirements (see section 4.2 Accreditation Process), the ST03 Trial Manager will inform GSK to dispatch the initial supply of lapatanib (for one cycle of treatment) to the pharmacy department.

The pharmacist will be responsible for ordering a further supply of lapatanib for subsequent cycles of treatment. The pharmacist should regularly check the expiry date of the lapatinib and ensure that replacement supplies are ordered from GSK via Catalent in good time to receive the shipment prior to any scheduled patient treatment. Delivery of lapatanib can take up to 5 working days from point of order, urgent deliveries are not possible. In the event of trial stock not being available, commercial stock may be used if this is in the patient's best interest and only after obtaining approval from the ST03 Trial Manager. However, commercial stock will not be replaced or reimbursed if used. In the event that this is authorised by the ST03 Trial Manager the site must document this incident with a file note in the Pharmacy Site file and Master site File/Patient File and should complete and return the incident report documentation provided by the ST03 Trial Manager.

Randomisations
When a new patient is randomised into the ST03 lapatinib comparison, the MRC CTU will notify the pharmacist (recorded on the ST03 accreditation) of the randomisation and the treatment regimen to which the patient has been allocated and the patient's treatment schedule will be sent to the Research Nurse.

Procedures for Destruction of lapatinib

Returned or out of date stock should be destroyed in accordance with local policies and procedures. Destruction of lapatinib should be documented on the 'ST03 Drug Destruction Form' for auditing and monitoring purposes and faxed to GSK.

Appendix K: How to calculate the number of capecitabine tablets to dispense following a dose reduction

Capecitabine is dose banded according to 3 BSA levels. Depending on the type of toxicity patients may require 1 of 4 possible dose reductions. The tables below show the percentage dose reduction based on the initial dose given at start of cycle 1.

Table 1: sECX Daily dose after a dose reduction (All patients randomised to sECX or sECX+B)

Initial daily dose given at cycle 1 according to BSA	15% dose reduction (daily dose) mg	25% dose reduction (daily dose) mg	30% dose reduction (daily dose) mg	50% dose reduction (daily dose) mg
**1800 mg**	1530	1350	1260	900
**2150 mg**	1828	1613	1505	1075
**2500 mg**	2125	1875	1750	1250

To assist you in working out the rounded total daily dose and the number of capecitabine tablets to dispense, please use the table 3 below.

Table 2: mECX Daily dose after a dose reduction (Patients randomised to mECX+L)

Initial daily dose given at cycle 1 according to BSA	15% dose reduction (daily dose) mg	25% dose reduction (daily dose) mg	30% dose reduction (daily dose) mg	50% dose reduction (daily dose) mg
**1500 mg**	1275	1125	1050	750
**1800 mg**	1530	1350	1260	900
**2000 mg**	1700	1500	1400	1000

To assist you in working out the rounded total daily dose and the number of capecitabine tablets to dispense, please use the table 3 below.

Table 3: Rounded total Daily Dose and No. of Tablet to dispense

Daily dose after reduction	Rounded total daily dose	Number of tablets to be taken in the morning		Number of tablets to be taken in the evening	
		**150mg**	**500mg**	**150mg**	**500mg**
776 – 900	800mg	2	0	0	1
901 – 1075	1000mg	0	1	0	1
1076 - 1225	1150mg	0	1	1	1
1226 – 1375	1300mg	1	1	1	1
1376 – 1525	1450mg	1	1	2	1
1526 – 1700	1600mg	2	1	2	1
1701 – 1900	1800mg	2	1	0	2
1901 – 2075	2000mg	0	2	0	2
2076 – 2225	2150mg	0	2	1	2

Example:
If a patient who received 2150mg as their total daily dose during cycle 1, required a 30% dose reduction, their total daily dose would be reduced to 1505 mg per day as shown in table 1. The actual rounded total daily dose prescribed would be 1450mg and administered as shown in Table 3.

Appendix L: Summary of Products Characteristics –Undesirable Effects

For the up to date summary of product characteristics including Contraindications, Special warnings and Special Precautions for Use and the Undesirable Effects for the drugs in the ECX chemotherapy please see the links below.

Please note that thromboembolic events are uncommon but expected events associated with combined ECX chemotherapy.

The current Investigator Brochures should be referred to for details on Bevacizumab and Lapatinib.

CAPECITABINE

http://www.medicines.org.uk/emc/medicine/4619/SPC/Xeloda+150 mg+and+500mg+Film-coated+Tablets/

Please note that this is the link to the Roche Xeloda SPC, if you are using an alternative brand of capecitabine please access the relevant SPC at www.medicines.org.uk

EPIRUBICIN

http://www.medicines.org.uk/emc/medicine/14474/SPC/PHARMORU BICIN/

Please note that this is the link to the Pfizer Pharmorubicin SPC, if you are using an alternative brand of epirubicin please access the relevant SPC at www.medicines.org.uk

CISPLATIN

http://www.medicines.org.uk/emc/medicine/623/SPC/Cisplatin+1+ mg+ml+Sterile+Concentrate/

Please note that this is the link to the Hospira Cisplatin SPC, if you are using an alternative brand of epirubicin please access the relevant SPC at www.medicines.org.uk.

Appendix M: TNM 7th edition staging criteria

Should your centre stage the tumours using TNM 7th edition, the table below should be used to convert the staging back to TNM6 in order to check that the patient is eligible for the trial.
In the TNM 7th ed. gastric and Siewert type III OGJ tumours are staged according to the TNM staging for stomach cancers and lower oesophageal and Siewert type I and II tumours are staged according to the TNM staging for oesophageal cancers.

The main point to note is that patients who have gastric/type III OGJ cancer with N3a disease according to TNM7 (N2 using TNM6) will be eligible, but patients with N3b disease according to TNM7 (N3 using TNM6) will NOT be eligible.

For any patient to be eligible, the surgeon must believe that an R0 resection can be achieved by excision of a contiguous structure.
When randomising the patient, the TNM6 tumour stage should be provided. If this is not available, please make sure this is stated during the call and the TNM7 options will be provided for you.

**TNM6**	**TNM7**	**Definition STOMACH (also to be used for Siewert type III OGJ tumours)**
		**Primary tumour (T)**
TX	TX	Primary tumour cannot be assessed
T0	T0	No evidence of primary tumor
Tis	Tis	Carcinoma in situ/High grade dysplasia
T1	-	Tumour invades lamina propria, muscularis mucosae, or submucosa
-	T1a	Tumour invades lamina propria or muscularis mucosae
-	T1b	Tumour invades submucosa
-	T2	Tumour invades muscularis propria
T2a	-	Tumour invades muscularis propria
T2b	-	Tumour extends into the subserosa
T3	-	Tumour perforates serosa (visceral peritoneum)
-	T3	Tumour invades subserosa
T4	-	Tumour invades adjacent structures
-	T4a	Tumour perforates the serosa
-	T4b	Tumour invades adjacent structures
		**Regional lymph nodes (N)**
NX	NX	Regional lymph nodes cannot be assessed
N0	N0	No regional lymph nodes
N1	-	Metastasis in 1 to 6 regional lymph nodes
-	N1	Metastasis in 1 to 2 regional lymph nodes
-	N2	Metastasis in 3 to 6 regional lymph nodes
N2	N3a	Metastasis in 7 to 15 regional lymph nodes
N3	N3b	Metastasis in more than 15 regional lymph nodes
		**Distant metastasis (M)**
M0	M0	No distant metastasis
M1	M1	Distant metastasis

TNM6	TNM7	Definition OESOPHAGUS (also to be used for Siewert type I / II OGJ tumours)
		**Primary tumour (T)**
TX	TX	Primary tumour cannot be assessed
T0	T0	No evidence of primary tumour
Tis	Tis	Carcinoma in situ (TNM6) high grade dysplasia (TNM 7)
T1	T1	Tumour invades lamina propria, muscularis mucosa or submucosa
-	T1a	Tumour invades lamina propria or muscularis mucosae
-	T1b	Tumour invades submucosa
T2	T2	Tumour invades muscularis propria
T3	T3	Tumour invades adventitia
T4	-	Tumour invades adjacent structures
-	T4a	Resectable tumour invading pleura, pericardium or diaphragm
-	T4b	Unresectable tumour invading adjacent structures, such as aorta, vertebral body, trachea etc.
		**Regional lymph nodes (N)**
NX	NX	Regional lymph nodes cannot be assessed
N0	N0	No regional lymph nodes
N1	-	Regional lymph nodes
-	N1	Metastasis in 1 to 2 regional lymph nodes
-	N2	Metastasis in 3 to 6 regional lymph nodes
-	N3	Metastasis in 7 or more regional lymph nodes
		**Distant metastasis (M)**
MX	MX	Distant metastases cannot be assessed
M0	M0	No distant metastasis
M1<br>M1a<br>M1b	M1<br>-<br>-	Distant metastasis<br>Metastasis in coeliac lymph nodes<br>Other distant metastasis

Appendix N: Algorithm for cardiac monitoring requirements for patients randomised to the lapatinib comparison (Arms C and D) only

* Please contact the MRC CTU should you have any queries regarding the required cardiac assessments or treatment advice.

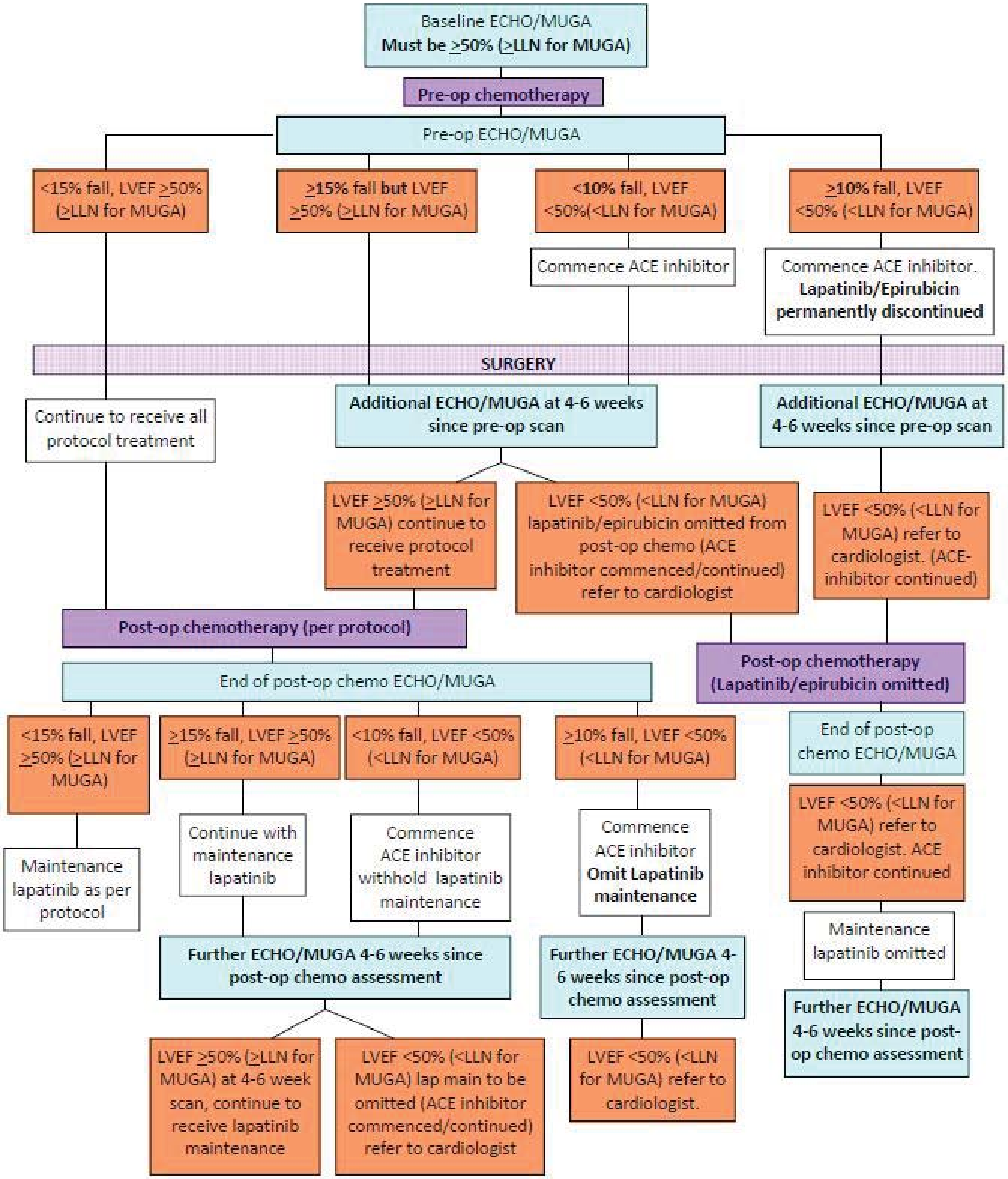
Baseline ECHO/MUGA
Must be >50% (>LLN for MUGA)
Pre-op chemotherapy
Pre-op ECHO/MUGA
<15% fall, LVEF >50% (>LLN for MUGA)
>15% fall but LVEF >50% (>LLN for MUGA)
<10% fall, LVEF <50%(<LLN for MUGA)
>10% fall, LVEF <50% (<LLN for MUGA)
Commence ACE inhibitor
Commence ACE inhibitor. Lapatinib/Epirubicin permanently discontinued
SURGERY
Continue to receive all protocol treatment
Additional ECHO/MUGA at 4-6 weeks since pre-op scan
Additional ECHO/MUGA at 4-6 weeks since pre-op scan
LVEF >50% (>LLN for MUGA) continue to receive protocol treatment
LVEF <50% (<LLN for MUGA) lapatinib/epirubicin omitted from post-op chemo (ACE inhibitor commenced/continued) refer to cardiologist
LVEF <50% (<LLN for MUGA) refer to cardiologist. (ACE-inhibitor continued)
Post-op chemotherapy (per protocol)
Post-op chemotherapy (Lapatinib/epirubicin omitted)
End of post-op chemo ECHO/MUGA
<15% fall, LVEF >50% (>LLN for MUGA)
>15% fall, LVEF >50% (>LLN for MUGA)
<10% fall, LVEF <50% (<LLN for MUGA)
>10% fall, LVEF <50% (<LLN for MUGA)
End of post-op chemo ECHO/MUGA
Maintenance lapatinib as per protocol
Continue with maintenance lapatinib
Commence ACE inhibitor withhold lapatinib maintenance
Commence ACE inhibitor Omit Lapatinib maintenance
LVEF <50% (<LLN for MUGA) refer to cardiologist. ACE inhibitor continued
Maintenance lapatinib omitted
Further ECHO/MUGA 4-6 weeks since post-op chemo assessment
Further ECHO/MUGA 4-6 weeks since post-op chemo assessment
Further ECHO/MUGA 4-6 weeks since post-op chemo assessment
LVEF >50% (>LLN for MUGA) at 4-6 week scan, continue to receive lapatinib maintenance
LVEF <50% (<LLN for MUGA) lap main to be omitted (ACE inhibitor commenced/continued) refer to cardiologist
LVEF <50% (<LLN for MUGA) refer to cardiologist.

Appendix O: ST03 Trial Committees

Trial Management Group

Chief Investigator

Professor David Cunningham
Dept of Oncology
Royal Marsden Hospital
Downs Road Sutton,
Surrey SM2 5PT
Tel: 0208 661 3156
Fax: 0208 643 9414
Email: David.Cunningham@rmh.nhs.uk

Clinical Investigators

Professor Derek Alderson
Dept. of Surgery
Academic Department of Surgery
Room 29, 4th Floor
Queen Elizabeth Hospital
Edgbaston
Birmingham B15 2TH
Tel: 0121 627 2276
Fax: 0121 472 1230
Email: d.alderson@bham.ac.uk

Mr. William Allum
Dept. of Surgery
Royal Marsden Hospital
Fulham Road
London
Tel: 0208 642 6011
Fax: 01372 277494
Email: wmallum@hotmail.com

Dr Tom Crosby
Dept. of Oncology
Velindre Hospital
Whitchurch
Cardiff CF14 2TL
Wales
Tel: 029 20316202/029 2061 5888
Fax: 029 2019 6118
Email: tom.crosby@velindre-tr.wales.nhs.uk

Mr. Robert Mason
Dept. of Surgery
St Thomas' Hospital
Lambeth Palace Road
London SE1 7EH

Tel: 020 7188 2572
Fax: TBC
Email: Robert.Mason@gstt.nhs.uk

Dr Kate Sumpter
Dept. of Oncology
Newcastle General Hospital
Westgate Road
Newcastle-Upon-Tyne
NE4 6BE
Tel: 0191 256 3594
Fax: 0191 256 3670
Email: Kate.Sumpter@nuth.nhs.uk

Professor Jane Blazeby
Dept. of Surgery
Bristol Royal Infirmary
Marlborough Street
Bristol
BS2 8HW
Tel: 0117 928 3512
Fax: 0117 9252 736
Email: J.M.Blazeby@bristol.ac.uk

Dr Heike Grabsch
Pathology and Tumour Biology
Leeds Institute of Molecular Biology
Wellcome Trust Brenner Building
St James's University Hospital
Beckett Street
Leeds, LS9 7TF
Tel: 0113 3438626
Email: h.i.grabsch@leeds.ac.uk

Dr Angela Riddell **MRI Sub-study CI**
Department of Diagnostic Imaging
Royal Marsden Hospital
Fulham Road
London
SW3 6JJ

Tel: 0208 642 6011 ext1026
Email: Angela.Riddell@rmh.nhs.uk

Professor Mike Griffin
Northern Oesophago Gastric Unit
Royal Victoria Infirmary
Queen Victoria Road
Newcastle upon Tyne
NE1 4LP
Tel: 0191 282 0234
Email: Michael.Griffin@nuth.nhs.uk

Dr Sue Chua **PET/CT Sub-study CI**
Nuclear Medicine and PET Department
Royal Marsden Hospital
Fulham Road
London
SW3 6JJ
Tel: 0208 661 3544
Email: Sue.Chua@rmh.nhs.uk

MRC CTU, Cancer Group Staff

MRC CTU, Aviation House, 125 Kingsway, London, WC2B 6NH

Trial Managers

Claire Robb
Tel: 0207 670 4804
Email: c.robb@ucl.ac.uk

Rahela Choudhury
Tel: 0207 670 4801
Email: Rahela.choudhury@ucl.ac.uk

Trial Fax: 0207 670 4818

Data Managers

Linda Ly
Tel: 020 7670 4922
Email: linda.ly@ucl.ac.uk

Leah Meaden
Tel: 020 670 4776
Email: l.meaden@ucl.ac.uk

Trial Email: ST03@ctu.mrc.ac.uk

Senior Trial Statistician
Prof Sally Stenning
Tel: 020 7670 4707
Email: sally.stenning@ucl.ac.uk

MRC CTU Lead
Dr Ruth Langley
Tel:: 020 7670 4718
Email: ruth.langley@ucl.ac.uk

Trial Statistician
Sam Rowley
Tel: 020 7670 4854
Email: samuel.rowley.09@ucl.ac.uk

Clinical Trial Project Manager
Lizzie Armstrong
Tel: 020 7670 4812
Email: elizabeth.armstrong@ucl.ac.uk

Trial Steering Committee (TSC)

The role of the TSC is to provide overall supervision of the trial and ensure that the trial is conducted according to the MRC guidelines for Good Clinical Practice. It also provides advice, through its independent Chairman, on all aspects of the trial. The TSC reviews the progress of the trial, adherence to the protocol and patient safety. It also considers any new information relevant to the trial and any results from other trials that may have a direct bearing on the future conduct of the trial. The Gastric Intestinal /Gynaecology TSC, chaired by Dr Chris Parker, will oversee this trial.

Independent Data Monitoring Committee (IDMC)

The role of the IDMC is to monitor the data during the trial and to make recommendations to the TSC on whether there are any ethical or safety reasons why the trial should be modified or discontinued. It will also consider any reported adverse events and any data emerging from other related studies. The IDMC is the only body involved in the trial that has access to unblinded data. Members of this committee include:

CHAIR: Dr Pippa Corrie
Oncologist

Professor Janet Dunn
Statistician

Mr Mike Hallissey
Surgeon

ST03 Sample Access Committee (SAC)

Proposals for translational research projects involving the ST03 material will be considered for approval by the ST03 Sample Access Committee and Independent Trial Steering Committee (TSC). The ST03 sample Access committee includes:

Dr. Heike Grabsch	Leeds Teaching Hospital NHS Trust
Dr Olorunda Rotimi	Leeds Teaching Hospital NHS Trust
Dr Andrew Wotherspoon	Royal Marsden Hospital NHS Trust
Prof. David Cunningham	Royal Marsden Hospital NHS Trust
Dr Ruth Langley	MRC CTU

Gastric Intestinal / Gynaecology TSC

Appendix P - Health Economics

The economic evaluation will take the form of a cost-effectiveness analysis (1) taking the perspective of the UK National Health Service. It will relate health service costs to changes in patients' quality-adjusted life expectancy. It will adhere to the methods guidelines for cost-effectiveness published by the National Institute for Health and Clinical Excellence (NICE) (2).

The differential cost of the two therapeutic strategies will be estimated on the basis of the resources consumed by patients in the trial and unit costs/prices routinely available in the NHS. Resource use will be measured in all patients during the follow-up period. Items measured will include details of the use of study medications, days in hospital (ward,intensive care etc), other anti-cancer therapies, out-patient attendances and use of some primary care/community-based services. These will be collected as part of the case record forms and through some additional questions on the quality of life questionnaires. To value this resource use in monetary terms, unit costs and prices will be applied based on the financial year in which the analysis is undertaken. These will be taken from routine NHS sources such as NHS Reference costs (3) and the British National Formulary.

Health-related benefits will be quantified in terms of quality-adjusted life years (QALYs) (1). These will reflect mortality in these patients, together with quality of life weights derived from patients' responses to the EQ5D (4) questionnaire which will be administered at time intervals defined for the quality of life questionnaires in Table 20.1.

The cost-effectiveness analysis will be implemented using a decision analytic model (5). This will provide analytical flexibility in several key respects. Firstly, it will facilitate extrapolation beyond the trial follow-up period which is necessary to estimate the differential mean quality-adjusted survival duration between the treatment strategies. Secondly, it will allow an assessment of cost-effectiveness in sub-groups based on patients' baseline characteristics. Potential sub-group characteristics will be specified in advance and will be consistent with those used in the clinical analysis. Thirdly, the use of a decision model will provide the opportunity to model the cost-effectiveness of additional treatment strategies to those assessed in the trial by bringing in external evidence. This will provide maximum information to decision makers such as NICE.

The cost-effectiveness analysis will present the cost-effectiveness of each management strategy in terms of mean costs and mean QALYs over patients' lifetimes. The uncertainty in the evidence from the trial (and, if appropriate, in external evidence) will be reflected using probabilistic methods (5) and presented using cost-effectiveness acceptability curves. The potential value of additional research will also be assessed based on these methods (5).

References:

1. Drummond MF, Sculpher MJ, Torrance GW, O'Brien B, Stoddart GL. *Methods for the* Economic Evaluation of Health Care Programmes. 3rd ed. Oxford: Oxford University Press, 2005.

2. National Institute for Clinical Excellence (NICE). Guide to the Methods of Technology Appraisal. London: NICE, 2004.

3. HS Executive. The New NHS - 2005 Reference Costs. www.doh.gov.uk/nhsexec/refcosts.htm. London: NHS Executive, 2005.

4. Kind P. The Euro QoL instrument: an index of health-related quality of life. In: Spilker B, editor. Quality of Life and Pharmacoeconomics in Clinical Trials. 2nd ed. Philadelphia: Lippincott-Raven, 1996:191-201.

5. Briggs A, Sculpher M, Claxton K. Decision Modelling for Health Economic Evaluation. Oxford: Oxford University Press, 2006.

Appendix Q: ST03 HER-2 Testing and Sample Handling Procedure

Pathology Sample for HER-2 Testing

All patients who give their consent to HER-2 testing require prospective testing of the HER-2 status of their primary tumour to determine their eligibility for randomisation and treatment within the lapatinib feasibility study. Each patient will have given consent for release of tumour material and as the pathologist you will be provided with a copy of the HER-2 testing consent form.

Receipt of these samples in an appropriate timeline is a rate-limiting step in starting the patient's treatment within the trial so it is very important that these samples are sent to the central testing laboratory at the Royal Marsden Hospital as soon as possible.

Procedures for pathologists:

- Once you receive the Sample Request and Report Form (ST03/HER-2Test 1), please locate a tumour block for that patient containing the maximum quantity of viable tumour as well as a copy of the associated histology report.

- Ensure the tumour block and associated pathology reports are anonymised; only include the patient's ST03 trial number and pathology number. If the patient has consented to future research, any other further analyses will also be performed anonymously.

- Complete the second section of the Sample Request and Report Form (ST03/HER-2Test 1) and enclose with the block and histology report and send all three to the RMH.

- Fax a copy of the completed Sample Request and Report Form (ST03/HER-2Test 1) to MRC CTU (0207 670 4818) and keep a copy for your own records.

One of the aims of the feasibility study is to assess the feasibility of the sample collection and testing process. We will therefore be documenting the dates of each step of the process.

A flow chart detailing the process is found below in Figure Q1:

Figure Q1: Overview of Pathology Block Request Process

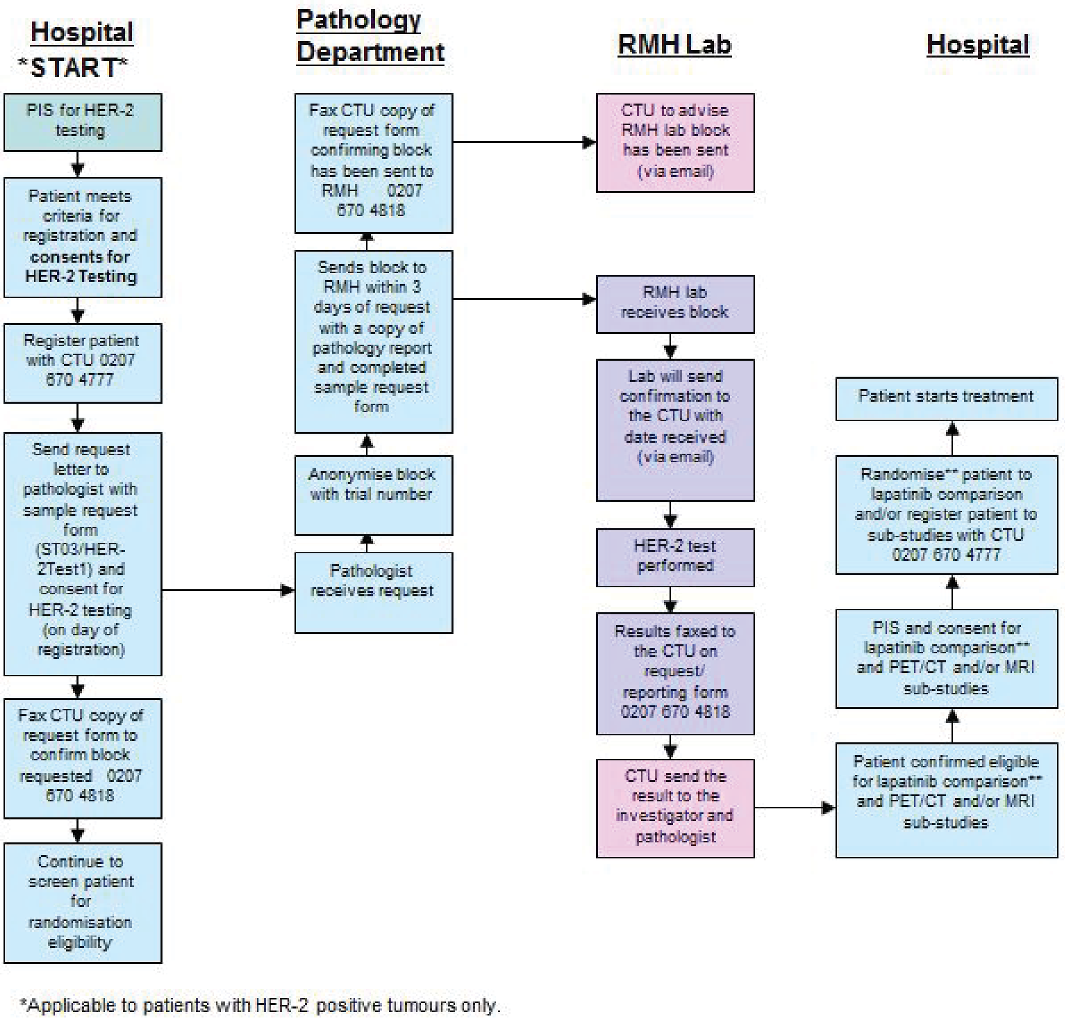

Please note it is estimated that it will take a maximum of ten working days to turnaround the results from the date of block receipt. The MRC CTU will contact the investigator/lead research nurse and pathology contact at each site as soon as the results are available.

We will return all blocks at the end of the trial but will undertake to return the blocks at short notice if they are required to further assist patient management.

A detailed laboratory standard operating procedure has been prepared for the RMH central testing laboratory. This can be supplied to your pathology department on request.

APPENDIX R – Capectabine and Lapatinib Modification and Dose Levels

mECX Capecitabine (Dose level 0 and Dose level -1 for mECX+L Arm)

See Table R1 below for the Capecitabine dose calculation to be used in mECX+L arm dose level 0 and dose level -1.

Table R1: Capecitabine Dose Calculation for mECX Dose Level 0 and mECX Dose level -1, According to Body Surface Area

BSA (m^2)	Total daily dose (mg)	Number of tablets administered in the morning		Number of tablets administered in the evening	
		150mg	500mg	150mg	500mg
<1.6	1500	0	1	0	2
1.6-1.8	1800	2	1	0	2
>1.8	2000	0	2	0	2

sECX Capecitabine (Dose level +1 for mECX+L Arm)

See Table R2 below for the Capecitabine dose calculation to be used in sECX, sECX+B and mECX+L dose level +1.

Table R2: Capecitabine Dose Calculation for sECX and mECX Dose Level +1 According to Body Surface Area

BSA (m^2)	Total daily dose (mg)	Number of tablets administered in the morning		Number of tablets administered in the evening	
		150mg	500mg	150mg	500mg
<1.6	1800	2	1	0	2
1.6-1.8	2150	0	2	1	2
>1.8	2500	0	2	0	3

Lapatinib Dose Levels

See table R3 below for lapatinib dose to be prescribed at dose level -1, dose level 0 and dose level -1 for patients in the mECX+L arm

Table R3: Lapatinib dose according to current dose level*

Dose level	Lapatinib mg/day	Number of tablets administered in the evening
		250mg
+1	1250	5
0	1250	5
-1	**1000**	4

***Centres participating in the Lapatinib feasibility study will be informed at randomisation which dose level is currently being used.**

Appendix S: PET/CT Sub-study Scanning Procedures

PET/CT sub-study Scanning procedures:

Patient Scheduling:

- Sequential PET exams for a patient must be performed in the same centre using the same accredited PET-CT system as the baseline scan. The patient preparation, FDG administration, image acquisition and reconstruction for these scans must be matched for each subsequent patient scan acquired for the study.
- If a diagnostic contrast-enhanced CT with intravenous or bowel contrast (other than water) is indicated this should ideally be performed after the PET-CT scan. If a contrast-enhanced CT has already been performed, the PET-CT scan should be performed a minimum of 3 days after the CT scan.

Patient Preparation:

- Non-diabetic patients should fast for at least 4 hours prior to the start of the PET study.
- Plain (non sugary / unflavoured) water should be taken during the period of fasting and the uptake period to ensure good hydration.
- Diabetics on oral medication should ideally be given a morning appointment, asked to fast for 4 hours and should omit their hypoglycaemic medication that morning.
- Diabetics on insulin should eat and administer their insulin as normal before 4h fast. If blood glucose level is >11mmol/l (>200mg/dl) then consideration should be given to rescheduling the scan. Insulin should not be administered to lower blood glucose.
- The blood glucose level of all patients should be measured on arrival at the imaging centre. This should be performed using a calibrated glucometer or similar bedside device.
- Patients should avoid strenuous exercise for 6 hours prior to the scan.
- Patients should be weighed without shoes and coats using a calibrated device.
- Intravenous CT contrast media should not be administered prior to the PET study.
    - If local protocols include a diagnostic CT scan using contrast as part of the PET-CT examination this should be performed after the PET scan. In this case a separate low dose CT without contrast should also be acquired before the PET acquisition and this scan should be used for attenuation correction of the PET images.

Radiopharmaceutical Administration:

Radiopharmacutical:	^{18}F-fluorodeoxyglucose (FDG)
Route of Administration:	Intravenous administration via butterfly cannula under quiet conditions.
Suggested dosage:	The injected dose is dependent on the PET system that is utilized and the patient weight:<br><br>Suggest 200 -350MBq (+/- 10%) for 3D systems and 300-400MBq (+/- 10%) under ARSAC 400MBq DRL and local centre discretion of injected activity > 400MBq for heavy patients. This is dependent on the local imaging criteria and users should not exceed country specific diagnostic reference levels.<br><br>For larger patients (>90kg) it is suggested to increase the scanning time rather than increasing the injected dose.<br><br>Injected activity for pre and post treatment scans should be matched +/-10%.
Procedure:	Assay the FDG injectate residue, and record the net activity injected and time of injection.

Uptake Period:

- During the ^{18}F-FDG administration, uptake phase and the PET-CT exam, the patient should remain seated and be kept warm to avoid uptake in the muscles or brown fat.
- Patients should be asked to void immediately prior to the PET-CT scan to reduce bladder activity.
- The PET emission acquisition should be started 60 minutes after the dose administration with a maximum of 70 minutes after injection.
- The response scans must be performed at the same time after injection as the baseline scan $\pm$ 10 minutes, but not less than 55 minutes post injection.

Image Acquisition:

- The PET and CT scan region should include the base of the brain to the upper thigh.
- Patients should be scanned with arms above the head if tolerated. Patient positioning should be matched on the response scans.
- Separate body and head and neck scans can be performed if locally accepted practice. If a separate head and neck is acquired the arms should be by down the sides.
- All other imaging parameters i.e. with regard to time per bed position, 2D or 3D, CTAC parameters must be agreed with the NCRI PET Core Lab prior to the start of the study. These should then be used throughout the study. Any changes to these parameters must be agreed with the Core lab before scanning any more patients.

Image Reconstruction:

- Attenuation correction should be performed using the low dose CT.
- Iterative reconstruction should be used e.g. OSEM or similar
- Both attenuation-corrected and non attenuation-corrected PET images should be reconstructed.
- All other reconstruction parameters i.e. with regard to number of iterations or filtering parameters must be agreed with the Core Lab prior to the start of the study. These should then be used throughout the study. Any changes to these parameters must be agreed with the Core lab before scanning any more patients.

Information to be recorded for each patient:

For each patient study, the PET-CT acquisition information and patient information must be recorded on the **PET-CT Acquisition Form** and sent to the Core Lab with the PET-CT images.

An electronic copy of the PET-CT Acquisition Form can be obtained by email from the ST03 Trial Team.

Data Transfer & Archive

PET-CT data must be transferred to the NCRI PET Core Lab at the same time as the completed PET-CT acquisition form.

The baseline and the early post-treatment PET-CT data should be saved locally on an approved data storage device. The following DICOM files are required:

- CT attenuation corrected half body images (skull base to mid thigh)
- Non-attenuation corrected half body images
- Half body CT scan
- PET/CT report from local imaging team

All image files must be compliant with DICOM PART 10 format. It is highly recommended that CD's or images be created and sent directly from the acquisition PET/CT workstation rather than from a secondary PACS system or file library. Specifically, image files that have been converted to savescreens and then reconverted back to DICOM format are NOT acceptable. Projection images (MIPs) and fused images are not required.

All PET-CT studies must be clearly named using the following filename convention:

STO3_<trial ID>_<initials>_baseline

STO3_<trial ID>_<initials>_response

Images and acquisition forms should be sent on CD by post to:

Lucy Pike
NCRI PET Core Lab
PET Imaging Centre
LG Floor, Lambeth Wing
St Thomas' Hospital
London SE1 7EH

It is strongly recommended that scans be sent by registered post (recorded or special delivery) or courier. These can be tracked on the Royal Mail/ courier website.

Alternatively scans can be sent electronically using secure file transfer. This will be established and validated for each scanning site by the NCRI PET Core Lab. In this case PET acquisition forms can be emailed (pet-trials@kcl.ac.uk) or faxed (0207 620 0790) to the Core Lab

Following review, all scan data will be archived at the **Central Review Lab, Nuclear Medicine and PET Department, The Royal Marsden Hospital.**

A. **PET/CT REPORTING AND REASSESSMENT**

To be completed by trial team

Baseline PET-CT will be done prior to the commencement of neoadjuvant chemotherapy. An interim PET/CT scan will be done after completion of the first cycle of neoadjuvant chemotherapy.

• PET/CT scans will be reviewed and scored by two named PET/CT specialists (Dr. Sue Chua, The Royal Marsden Hospital and Prof. Gary Cook, Guy's and St. Thomas' Hospital) at the core lab. Differences in reporting will be resolved by consensus between two PET/CT Specialists or by a third PET/CT Specialist from another trial centre where agreement cannot be reached. Visual and semi-quantitative interpretation will be used. Reviewers will not be blinded to the patient's clinical status. Differences in reporting will be resolved by consensus between two PET/CT specialists at the same core lab or by a third PET/CT specialist at another participating centre where agreement cannot be reached.

The PET/CT response scans will be scored with reference to sites of presumed disease involvement on the PET/CT baseline scan. FDG-PET scan is defined as positive (score 3, 4, 5) and negative (score 1, 2).

<u>Negative PET scan:</u>

1 no uptake

2 uptake ≤ mediastinum

NOTE if mediastinal blood pool activity is equal or greater than liver then the uptake within the lesion should be compared with liver (lesion uptake less than liver = score 2)

<u>Positive PET scan:</u>

3 uptake > mediastinum but ≤ liver

4 moderately increased uptake compared to liver at any site

5 markedly increased uptake compared to liver at any site or new areas of uptake thought to be related to the primary tumour

X new areas of uptake unlikely to be related to the primary tumour **(e.g. infective / inflammatory process)**

Scores 1, 2 with uptake in sites abnormal on the staging scan equal or less than liver uptake will be regarded as 'negative' for disease and scores 3, 4, 5 with uptake greater than liver will be regarded as 'positive' for disease. For the purposes of treatment, patients with a score of 3 will be regarded as positive for disease. Scores 1X and 2X will also be regarded as 'negative' for oesophageal carcinoma.

RADIATION DOSIMETRY

The effective dose associated with an administration of 400 MBq 18-FDG is 8.0 mSv (ARSAC Notes for Guidance 2006). The target organ is the bladder wall, which will receive 68.0 mGy (ICRP Publication 53). The CT attenuation correction using 80 mA and 140 kV will be approximately 8 mSv for the half body. (This will be country specific).

National regulations must be complied with in regard to the administration of radioactive substances and the CT exposure for the purpose of this study.

ARSAC approval

An ARSAC research certificate needs to be obtained at the local centre for the early post treatment FDG PET/CT and this must be obtained individually for each participating PET centre prior to starting the study. A copy of the letter required for an ARSAC application and an approved ARSAC certificate obtained by the lead centre (Royal Marsden Hospital) is available from the ST03 Trial Manager on request.

PET/CT QC PROCEDURES

Common standards and careful quality control is essential for the success of multi-centre trials such as this one. The procedures below fulfil the requirements of the NCRI PET Clinical Trials Network and are based on the EANM procedure guidelines for tumour PET Imaging (Boellaard et al 2009).

Imaging Facilities

Only full-ring dedicated PET-CT scanners are acceptable.
Scanning facilities must undergo the site accreditation process as detailed below and have received written confirmation that they fulfil the requirements of the study before scanning any patients as part of the trial.

A documented quality assurance program must be in place and records kept covering daily, weekly, monthly, quarterly and annual QC testing. A copy of the QC schedule should be provided to the Core Lab along with example results.

Anonymised scan data will be transferred between scanning facilities and the Core Lab using established secure electronic transfer or via CD.

All files must be clearly named using a pre-arranged file naming convention.

Named persons (and their deputies) should be identified with responsibility for scanning, QC and data transfer at participating PET-CT centres.

It must be demonstrated that image quality is comparable between centres and standard uptake values can be reliably determined from the PET-CT images.

The proposed data acquisition/reconstruction protocol (including details of the time per bed position, 2D or 3D, CTAC parameters, reconstruction parameters etc) must be agreed with the Core Lab before scanning can start. Generally a time per bed of less than 2 minutes for 3D and less than 3 for 2D are not acceptable.

All image files must be compliant with DICOM PART 10 format.

Site Accreditation Process

Before a PET centre can participate in the trial it must undergo the formal site accreditation process. **Scanning as part of the clinical trial must not start until written confirmation of compliance with the technical requirements of the trial is received from the Core Lab.**

The site accreditation must also be repeated by a PET Centre in the following situations:
After any software or hardware changes which may affect the scanner image quality.
If there are any significant changes to the acquisition or reconstruction parameters originally specified in the ***PET-CT Scan Quality Control document***.

Any other circumstances which arise that the Core Lab deems may alter the image quality, such as QC failures, apparent scanner degradation or poor image quality.

It is the responsibility of the PET centre to inform the Core Lab of any upgrades to the scanner hardware or software prior to the upgrade. If the upgrade is likely to affect the image quality the site will be required to repeat the phantom scans before continuing to scan patients as part of the trials.

No patients are to be scanned until all of the following steps have been completed:

The ***PET-CT Scan Quality Control document*** must be completed and forwarded to the core lab.

Initial 'start-up' scanner quality control procedures must be performed

Two anonymised representative patient studies must be transferred to the core lab.
The data transfer and anonymization procedure must be set up and validated
Written confirmation from the core lab that scanning can now start at your centre must be received.

A copy of the ARSAC certificate must be sent to the **clinical trials unit**.

Initial start-up QC procedures

All PET-CT scanners to used for the trial should be calibrated against the institutions own radionuclide calibrator.

The restriction of the study to full ring dedicated PET-CT scanners should ensure that the images acquired at all centres are of a comparable quality. In order to confirm this and check the SUV accuracy of each scanner, a standard phantom should be scanned at each of the participating centres using the local study protocol. This could be done by a representative from the core lab who visits the scanning facility or a representative at the scanning facility if approved by the core lab. Ideally however a personal visit from the core lab to scanning facilities is useful to establish contact and answer individual questions relating to the study for smooth running of the study.

The phantom will consist of the NEMA IEC PET body phantom or EU chest phantom, filled with water throughout, containing 6 small spheres. The spheres will be filled with 25 kBq/ml of ^{18}F- solution and the rest of the phantom with 5 kBq/ml of ^{18}F- to simulate small regions of tracer uptake in the abdomen.

Data will be acquired using the same acquisition and processing parameters that will be used for the patient studies. These parameters may vary between sites. Data will be evaluated in terms of absolute activity measurements for the background and the spheres. Two nuclear medicine physicians or radiologists trained in PET-CT will also assess the visual quality of the scans. If significant disparities are observed, for example, from the use of widely differing reconstruction parameters, these will be resolved prior to the start of the study.

The phantom images will be assessed at the NCRI PET Core Lab based at St Thomas' Hospital.

Ancillary Equipment

As this study uses SUVs defined in terms of patient weight, the scales used to weigh the patients must be calibrated. As a minimum the scales must be checked using a standard weight at least annually and should be accurate to within ±1kg of a standard weight of 70 kg and records kept.

The BM glucometer QC should be performed according to the manufacturer's or institution's procedure to ensure proper functioning.

Quality assurance procedures for the radionuclide calibrator must be in place and activity measurements for ^{18}F should be traceable to a primary standard. QC tests should include daily constancy checks and annual accuracy and linearity.

Clocks used to record the assay time and injection time should be synchronized to the scanner time.

Representative Patient Studies

Two anonymised patient studies (attenuation corrected PET, CT and non-attenuation corrected PET) acquired using the proposed study protocol should be transferred to the core lab for quality assessment together with the **TEST PATIENT DATA FORM**.

Data Format and Archiving

All studies to be transferred to the core lab (attenuation corrected PET, non-attenuation corrected PET, and CT) must be in DICOM format. BMP files, jpeg files, screen saves and hard copies are not acceptable. In general image data transferred through a PACS system will not be accepted, as many PACS systems convert DICOM images to another format and then reconvert them back to DICOM when exporting to a CD or FTP. Data should ideally be recorded directly onto CD using the PET scanner's workstation or other multimodality image viewing software. The data transfer procedure will be tested and validated by the Core Lab. The reconstructed CT, PET AC (CT-attenuated PET) and NAC (non-attenuated PET) data are to be archived locally. Raw PET data must be archived according to local protocol, and at least until the images have been accepted by the Core Lab in case additional reconstructions are required.

Data transfer and anonymisation procedure

All patient identifying information must be removed from the images prior to transfer. A procedure for naming, anonymising and transferring studies from the scanning site must be established. This will vary between sites. This can be validated when transferring the test phantom and patient data as above.

Routine scanner QC procedures

A documented PET-CT scanner quality assurance program must be in place and records kept, covering daily, monthly, quarterly and annual QC testing. A copy of the QC schedule must be sent to the core lab along with example results. Records should be made available for inspection by the Core lab if requested

The PET scanner should have an up-to-date calibration and normalization. On the day of scanning a trial patient the manufacturer's recommended daily QC should be performed and if any failures or abnormalities are identified that could affect the quality of the PET scan; consideration should be given to rescheduling the scan.

The routine CT QC should be performed according to the manufacturer's recommendations, but must include a water filled phantom scanned on a weekly basis, to measure image noise and CT number as described in IPEM (Institute of Physics and Engineering in Medicine) report 91.

Additional scanner QC required during the trial

SUV is used as a primary response end point, therefore accurate and consistent estimation of Standard Uptake Values for all patient scans and between all participating centres is required. This will be achieved via a rigorous and regular testing of SUV accuracy and consistency of all participating scanners.

A uniform phantom must be scanned prior to the start of each scanning session in which a patient is to be scanned as part of the trial. This can either be a resin ^{68}Ge phantom (where available) or an ^{18}F water filled phantom. The activity concentration in the ^{18}F phantom should be approximately 5kBq/ml. The average SUV for a large ROI placed at the centre of the phantom must be 1.00 $\pm$ 10% and on visual inspection the image should show no artefacts. The relevant sections of the patient data sheet must be completed to confirm the results of this test. The ^{18}F or ^{68}Ge phantom images must be sent, with the patient images to the core lab. If the test fails the named physicist at the core lab should be contacted. The scan must not take place until the reason for this failure has been resolved.

Confirmation that study can start at your site

When all the above has been completed, a letter will be forwarded to both the PET centre and the clinical trials unit to confirm that the centre can now participate in the trial. No subjects should be scanned before this has been received.
Scanning sites must inform the Core Lab of any upgrades to the scanner hardware or software prior to the upgrade. If the upgrade is likely to affect the image quality the site will be required to repeat the phantom scans before continuing to scan patients as part of the trial. Sites must also notify the Core Lab immediately of any deviations in QC and scan acquisition or reconstruction parameters from those agreed.

Contact

For enquiries relating to the scanning protocol, please contact the PET-CT Sub-study CI:

Name:	Dr. Sue Chua
Central Review Centre:	Nuclear Medicine and PET Department<br>The Royal Marsden Hospital<br>Downs Road<br>Sutton SM2 5PT
Phone No:	0208 661 3544
Email Address:	sue.chua@rmh.nhs.uk

For enquiries relating to the quality control and data transfer only, please contact the following at the core lab:

Name:	Lucy Pike
Core Lab Centre:	NCRI PET Core Lab<br>PET Imaging Centre<br>LG Floor, Lambeth Wing<br>St Thomas' Hospital<br>London SE1 7EH
Phone No:	0207 188 7445
Fax No:	0207 620 0790
Email Address:	pet-trials@kcl.ac.uk

For all other enquiries please contact the ST03 trial team at the MRC CTU .

PET-CT SCAN QUALITY CONTROL DOCUMENT

Please complete this document and return to the NCRI PET Core Lab (contact details provided at the end of the form), before any patient from the trial undergoes a PET/CT scan.

PET Centre:	

Contacts at Scanning Site:

Person responsible for performing the scanning procedures

Name	
Telephone	
Email	

Deputy to cover leave

Name	
Telephone	
Email	

Person responsible for ensuring adherence to quality control procedures

Name	
Telephone	
Email	

Deputy to cover leave

Name	
Telephone	
Email	

Person responsible for data anonymisation and data transfer

Name	
Telephone	
Email	

Deputy to cover leave

Name	
Telephone	
Email	

Scanner Technical Specification:

Please confirm that you have a:

Full ring PET/CT scanner	☐ Yes	☐ No

Please state:

Manufacturer & Model	
Date of installation	
Axial Field of View	
Sensitivity in cps/MBq/ml for a uniform 20cm cylinder	

Quality Control Procedures:

Is a documented quality assurance program in place?	☐ Yes	☐ No

Setup and Normalisation:

The frequency at which the PM tubes of the PET scanner are set-up is	
The frequency at which normalisation is carried out on the PET scanner is	

Daily and Weekly PET and CT Checks:

CT tube warm up and air calibration are carried out on a daily basis	☐ Yes	☐ No
Manufacturer's recommended daily PET QC test carried out *	☐ Yes	☐ No
* Please provide details of the manufacturer's daily PET QC tests and any further daily QC tests carried out (continue on a separate sheet if required)		
CT number and noise are measured on a weekly basis (as described in IPEM Report 91)	☐ Yes	☐ No

Monthly / Annual Quality Control:

Sensitivity of the PET scanner checked on at least an annual basis	☐ Yes	☐ No
Annual CT checks are carried out by CT experts on an annual basis (as described in IPEM Report 91)	☐ Yes	☐ No
Frequency of PET/CT scanner alignment is (minimum annual)		

Additional Procedures to be undertaken as Part of this Study:

Scan of a uniform $^{18}F/^{68}Ge$ phantom will be carried out to check image quality and confirm that SUV measures 1 ± 10% on the morning	☐ Yes	☐ No

of the study	
QC of the weighing scales will be carried out at least annually	☐ Yes ☐ No
If any of the procedures described in this document cannot be carried out for whatever reason a physicist from the core lab will be contacted immediately and no further studies will be undertaken by your centre until the issues have been resolved	☐ Yes ☐ No

Data Acquisition and Reconstruction:

Please supply the following information for the protocol to be used in this study, this will be the protocol used for all data acquired at your centre as part of this trial:

Half body emission scan duration per bed position (give time in minutes)	
Acquisition mode (specify 2D or 3D)	
Slice overlap	

CT details for half body attenuation correction:

MAs		kV_p		Pitch		Slice thickness (mm)	

Emission scan reconstruction parameters:

Matrix size (e.g. 128*128*31)	
Voxel size (e.g. 2.0*2.0*2.0mm^3)	
Reconstruction algorithm (e.g. OSEM)	
Smoothing filter and cut-off if used (e.g. Hannning, 0.5 Nyquist)	
Reconstruction algorithm parameters (number of iterations, subsets)	

Signed by person responsible for ensuring adherence to quality control procedures:

Name: Date:

Please return a signed copy of the completed form to:

Lucy Pike
PET Imaging Centre
LG Floor, Lambeth Wing
St Thomas' Hospital
Lambeth Palace Road
London SE1 7EH

Tel: +44 (0)20 7188 7445
Fax: +44 (0)20 7620 0790
Email: pet-trials@kcl.ac.uk

PHANTOM SCAN EVALUATION (to be completed by Core Lab)

PET Centre:

Scanner Manufacturer and Model:

QC tests performed by:

Date:

Qualitative Analysis

Image Quality	Acceptable	Not Acceptable
PET/CT alignment on core centre reporting system	Acceptable	Not Acceptable
Comments		
Sphere activity concentration at scan start time:		kBq/ml
Sphere activity concentration at scan start time:		kBq/ml

	**Activity Concentration**		
**Sphere diameter (mm)**	**Measured (M)** kBq/ml	**Actual (A)** kBq/ml	**Ratio** M / A
37			
28			
22			
17			
13			
10			

A recovery curve should be generated from the tabulated data:

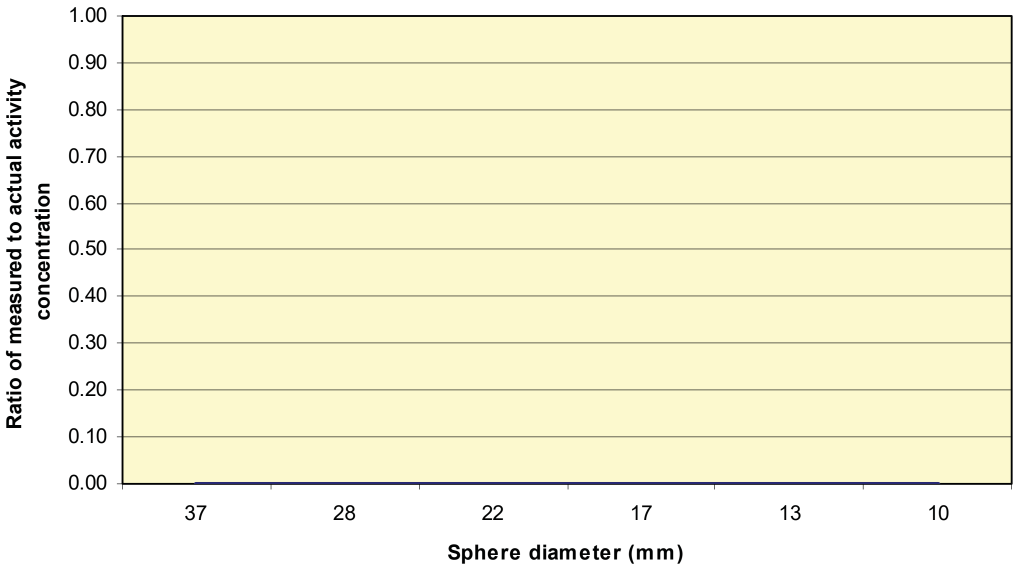

Average SUV for a large ROI positioned over the background: _________ (1.00 ± 0.10)

Recovery Curve:	Acceptable	Not acceptable

TEST PATIENT DATA FOR PET- CT SCAN - PATIENT 1

PET-CT scan acquired at   (PET/CT centre)

Patient's initials:

Date of PET-CT scan:

Time of administration of activity (hh:mm)	
Activity at time of administration (MBq)	
Patient height (cm)	
Patient weight (kg)	
Patient fasting state (time last ate)	
Patient blood glucose	
SUV measured for the uniform phantom scan	
Daily quality control result for the day of the scan	

	START TIME	NO OF BED POSITIONS	DURATION PER BED POSITION	TOTAL SCAN DURATION
Half body scan				
Head & neck scan (if acquired)				

Test data review (to be completed by Core Lab)
Comments

APPROVED	REJECTED

Name   Date

Name   Date

TEST PATIENT DATA FOR PET- CT SCAN - PATIENT 2

PET-CT scan acquired at (PET/CT centre)

Patient's initials:

Date of PET-CT scan:

Time of administration of activity (hh:mm)	
Activity at time of administration (MBq)	
Patient height (cm)	
Patient weight (kg)	
Patient fasting state (time last ate)	
Patient blood glucose	
SUV measured for the uniform phantom scan	
Daily quality control result for the day of the scan	

	START TIME	NO OF BED POSITIONS	DURATION PER BED POSITION	TOTAL SCAN DURATION
Half body scan				
Head & neck scan (if acquired)				

Test data review (to be completed by Core Lab)
Comments

APPROVED	REJECTED

Name Date

Name Date

NOTIFICATION TO PET SCANNING FACILITY OF APPROVAL TO SCAN PATIENTS IN TRIAL

From:	
To:	PET Scanning Facility
Fax Number:	
To:	**Trial co-ordinator**
Fax Number:	

Approved for trial

QC Procedures	Yes	No
Scan acquisition and reconstruction parameters	Yes	No
Phantom data and patient test data	Yes	No
Data transfer	Yes	No

Name Date

Patient test data	Yes	No

Name 1 Date

Name 2 Date

The above named Centre has complied with the requirements for PET-CT scanning and is a recognised scanning facility in the trial. The Centre undertakes to notify the Core Lab immediately of any deviations in QC and scan acquisition or reconstruction parameters from those agreed.

PET / CT Sub-study EORTC 1999 Response Criteria

Complete metabolic response (CMR) would be complete resolution of [^{18}F]-FDG uptake within the tumour volume so that it was indistinguishable from surrounding normal tissue.

Partial metabolic response (PMR) would be classified as a reduction of a minimum of 15–25% in tumour [^{18}F]-FDG SUV after one cycle of chemotherapy, and greater than 25% after more than one treatment cycle. Reporting would need to be accompanied by adequate and disclosed reproducibility measurements from each centre. An empirical 25% was found to be a useful cut-off point, but there is a need for a reproducibility analysis to determine the appropriate cut-offs for statistical significance. A reduction in the extent of the tumour [^{18}F]-FDG uptake is not a requirement for partial metabolic response.

Stable metabolic disease (SMD) would be classified as an increase in tumour [^{18}F]-FDG SUV of less than 25% or a decrease of less than 15% and no visible increase in extent of [^{18}F]-FDG tumour uptake (>20% in the longest dimension).

Progressive metabolic disease (PMD) to be classified as an increase in [^{18}F]-FDG tumour SUV of greater than 25% within the tumour region defined on the baseline scan, visible increase in the extent of [^{18}F]-FDG tumour uptake (>20% in the longest dimension) or the appearance of new [^{18}F]-FDG uptake in metastatic lesions.

Appendix T – ST03 Original Trial Bevacizumab Comparison Now Complete

1. ST03 Trial Summary

Recruitment to the bevacizumab comparison is now complete. Please use this Appendix for further information on the original ST03 trial design, background information and for the treatment and follow up of patients in the bevacizumab comparison.

1.1 Type of design

The ST03 original trial was a randomised, multi-centre, open-label phase II/III trial designed to evaluate the safety and efficacy of adding bevacizumab, a humanised monoclonal antibody against vascular endothelial growth factor (VEGF), to peri-operative chemotherapy for patients with Human Epidermal Growth Factor Receptor-2 (HER-2) negative (or unknown) operable oesophagogastric adenocarcinoma.

The original ST03 trial bevacizumab comparison was in two stages. The first stage, phase II, was completed in April 2010 and confirmed the safety and feasibility of combining bevacizumab with chemotherapy in this clinical setting (Okines, ASCO 2011). The second stage, phase III, will assess the efficacy of this combination.

In October 2013, based on a review of cumulative data from the ST03 trial bevacizumab comparison by the IDMC, an Urgent Safety Amendment was implemented to amend the inclusion criteria to exclude patients with lower oesophageal, Siewert Type I, II or III OGJ adenocarcinomas from entering the bevacizumab comparison. The comparison remained unchanged for patients with gastric adenocarcinomas who did not require an oesophagogastrectomy. This Urgent Safety Amendment also mandated that patients with lower oesophageal, Siewert Type I, II or III OGJ adenocarcinomas who were already entered into the sECX+B arm (Arm B) must not receive any further pre-operative bevacizumab. Following surgery patients may resume treatment with bevacizumab provided that there are no other contraindications.

Further details on the reasons for this change to eligibility criteria are given in Section 2.4 of this Appendix.

1.2 Eligible Patients

Patients with histologically confirmed gastric adenocarcinoma were eligible.

For full eligibility criteria for the bevacizumab comparison please refer to Section 4 of this appendix.

1.3 Protocol Therapy (Randomisation to Arms A and B is now closed)
Patients randomised to the bevacizumab comparison (Arms A and B)
Patients randomised to control **Arm A** will receive standard ECX (sECX) consisting of 3 cycles of Epirubicin 50 mg/m^2 IV day 1, Cisplatin 60 mg/m^2 IV day 1 and Capecitabine 1250 mg/m^2 po daily in 2 divided doses Day 1-21 pre-operatively, surgery as detailed in the protocol followed by 3 post-operative cycles of sECX at the same doses.

Patients randomised to the investigational **Arm B** (sECX + Bevacizumab) will receive treatment as specified above but in addition on day 1 of every cycle of chemotherapy they will receive bevacizumab **7.5 mg/kg IV**. In addition once the 3 cycles of post-

operative chemotherapy are completed they will receive 6 doses of maintenance bevacizumab **7.5mg/kg IV** once every 21 days.

1.4 Duration of Treatment

Patients randomised to the bevacizumab comparison (Arms A and B)

Control Arm A:

Pre-operative chemotherapy is expected to take 9 weeks and surgery should take place 5-6 weeks after this. Post-operative chemotherapy should recommence 6-10 weeks after surgery and should last for another 9 weeks. Therefore the duration of treatment in the control arms is expected to be 30-34 weeks.

Investigational Arm B: sECX+B

Treatment intervals will be the same as for the control arms A & C. The 5-6 week interval between the last capecitabine tablet and surgery will also ensure that patients have at least an 8 week break between the last pre-operative injection of bevacizumab given at the beginning of the 3rd cycle and their surgery, to minimise the risk of bevacizumab-related peri-operative morbidity. Post-operative chemotherapy should recommence 6-10 weeks after surgery and should last for another 9 weeks. Patients in this arm will also receive 6 maintenance injections of bevacizumab lasting 18 weeks so that the total duration of therapy on the investigational arm will be 52 weeks.

1.5 Outcome measures: sECX+/-B

Stage I was designed to assess the safety and feasibility of combining bevacizumab with sECX chemotherapy in patients with operable oesophagogastric adenocarcinoma. Safety was assessed by monitoring the number of gastric perforations at the site of the primary tumour, cardiac complications, wound healing complications, GI bleeding and perforations elsewhere in the intestine (1). Feasibility was assessed in terms of the patients' acceptance of the randomisation and the proportion of patients able to complete the treatment. In Stage II the primary outcome will be overall survival. Secondary outcomes will include response rates to pre-operative treatment, surgical complete resection rates, treatment-related morbidity, disease-free survival, quality of life and cost-effectiveness.

1.6 Data Maturity

The primary analysis of the bevacizumab comparison will take place when 420 deaths have been reported.

1.7 Assessments

Patients will be assessed 3 weekly while they are receiving pre- and post-operative chemotherapy. Patients will then commence 9 weekly follow-up assessments at 9, 18 and 27 weeks from the start of cycle 4. Patients will receive follow up 1 year from surgery, 6 monthly for years 2 and 3 and then annually until death. Patients will also be asked to complete questionnaires aimed at assessing the effects of the investigational treatments on their quality of life (QoL) and on their use of health care resources (Health Economics (HE) study).

1.8 Sample Size

For the original ST03 bevacizumab comparison, approximately 950 patients (max 1100) with HER-2 negative or undefined tumours will be recruited in order to observe 420 deaths. Recruitment is likely to take between 5-6 years for this comparison.

2. Background and Rationale for the use of Bevacizumab

2.1 Evidence for the Use of Bevacizumab

Bevacizumab is a monoclonal antibody against VEGF. VEGF is an important physiological and pathological regulator of angiogenesis (the development of new blood vessels) and therefore has a role in the growth of cancers. In addition to direct anti-angiogenic effects, bevacizumab is also believed to improve the delivery of chemotherapy to cancerous tissue by altering tumour vasculature and decreasing elevated interstitial pressure in tumours (2). Pre-clinical experiments have demonstrated bevacizumab's ability to inhibit the growth of a variety of human cancer cell lines grown in nude mice (3-5).

Bevacizumab has been evaluated in a number of phase II/III clinical trials in a number of different tumour types. The largest published phase III trials in the advanced disease setting are summarised in **Table 2** (6-13). Bevacizumab has been associated with a statistically significant improvement in overall survival in breast cancer, colorectal cancer and non-small cell lung cancer. For example, in a study by Hurwitz *et al*. bevacizumab combined with irinotecan and 5-FU improved the median duration of survival of patients with metastatic colon cancer to 20.3 months compared to 15.6 months in the chemotherapy alone group (HR for death of 0.66; $p<0.001$) (11). In the ECOG 3200 study, 829 patients with previously treated advanced colorectal cancer were treated with either bevacizumab alone or in combination with oxaliplatin and 5-FU (FOLFOX4) or FOLFOX4 alone. The median overall survival was 12.9 months for the combined arm and 10.8 months for chemotherapy alone ($p=0.0018$) (13). Benefit has also been demonstrated when bevacizumab has been combined with other chemotherapy regimens in phase II studies such as 5FU alone (14), and with oxaliplatin based chemotherapy in the TREE-2 (15) study. It is therefore reasonable to expect that similar benefits might be seen with other chemotherapy regimens such as ECX.

In a phase II study in advanced gastric cancer, where bevacizumab was combined with cisplatin and irinotecan the response rate was 65%, median time to progression was 8.3 months and median overall survival was 12.3 months (16). In comparison, a phase II study of cisplatin and irinotecan alone in similar patients reported an overall response rate of 58% for 36 assessable patients (17).

In advanced gastric cancer, the AVAGAST trial investigated the addition of bevacizumab to a cisplatin/capecitabine doublet which led to a statistically significant improved response rate (46% compared to 37%, $p=0.0315$) and prolongation of median progression-free survival (6.7 compared to 5.3 months, $p=0.0037$), but not to a significant prolongation of median overall survival (12.1 compared to 10.1 months, $p=0.102$) (7). Despite the negative findings of AVAGAST, it is felt that the rationale for the ST03 trial remains, especially given the increased response rate seen in the bevacizumab containing arm, as response rate is key in the neoadjuvant setting with respect to increased levels of complete resection. This is supported by the success of bevacizumab in increasing response rate and pathological response rate in a phase III randomised trial when added to neoadjuvant chemotherapy (epirubicin, cyclophosphamide and docetaxel) for the treatment of locally advanced triple negative breast cancer (see Table 2) (6)

High levels of VEGF expression in gastric adenocarcinoma have been shown to correlate with poor 5-year survival rates, lymph node metastasis and vascular invasion

(18). Targeting VEGF in gastric cancer is therefore a reasonable strategy and may prevent the development of metastases due to the requirement of VEGF for the growth of tumour vasculature. A potential benefit for combining bevacizumab with chemotherapy for adenocarcinomas of the stomach, oesophagogastric junction (OGJ) and lower oesophagus might be expected on the basis of the results described above in advanced gastric cancer (7).

Table 2: Summary of large phase III trials involving Bevacizumab in the advanced setting

	Treatment Arms	Response Rate (%)	Median PFS (months)	Median OS (months)
Locally advanced breast cancer (neo-adjuvant) (von Minkwitz et al) (12)	Epirubicin/cyclophosphamide →docetaxel<br><br>Epirubicin/cyclophosphamide →docetaxel/bevacizumab	14.9% (27.9 triple negative)<br>18.4% (39.3% triple negative)	Not Reported	Not Reported
Metastatic gastric cancer (Ohtsu et al) (13)	Cisplatin/fluoropyrimidine<br><br>Cisplatin/fluoropyrimidine Bevacizumab	37%<br><br>46%<br>p=0.0315	5.3<br><br>6.7<br>p=0.0037	10.1<br><br>12.1<br>p=NS
Advanced ovarian cancer (high risk patients) (Perren et al) (14)	Carboplatin/paclitaxel<br><br>Carboplatin/paclitaxel Bevacizumab	Not Reported<br><br>Not Reported	22.4<br><br>24.1<br>P=0.04	28.8<br><br>36.6<br>P = 0.002).
Metastatic Breast Cancer (2nd/3rd line treatment) (*Miller et al.*) (15)	Capecitabine (n=230)	9.1	4.17	14.5
	Capecitabine Bevacizumab (15mg/kg/3wks) (n=232)	19.8 (p=0.001)	4.86 (p=NS)	15.1 (p=NS)
Locally recurrent/<br><br>metastatic breast cancer (1st line) (*Miller et al.) (16)*	Paclitaxel (n=350)	14.2	6.1	21
	Paclitaxel Bevacizumab (10mg/kg/2wks) (n=365)	28.3 (p<0.0001)	11 (p<0.001 HR=0.50)	27 (p<0.01 HR=0.67)
Metastatic colorectal cancer (1st line) (*Hurwitz et al.) (17)*	Irinotecan, fluouracil and leucovorin (IFL) (n=411)	34.8	6.2	15.6
	IFL/placebo Bevacizumab (5mg/kg/2wks) (n=402)	44.8 (p<0.004)	10.6 (p<0.001 HR=0.66)	20.3 (p<0.001 HR 0.66)
Non-squamous NSCLC<br><br>IIIb/IV (*Sandler et al.*) (18)	Carboplatin Paclitaxel (n=431)	10	4.5	10.2
	Carboplatin Paclitaxel Bevacizumab (15mg/kg/3wks) (n=424)	27.2 (p<0.0001)	6.4 (p<0.0001 HR=0.62)	12.5 (p=0.007 HR=0.77)
Recurrent metastatic colorectal cancer (Prior 5FU/ irinotecan) ECOG 3200 (*Giantonio et al.) (19)*	Bevacizumab (10mg/kg/2wks) (n=243)	3.0	2.7	10.2
	FOLFOX4 (n=290)	9.2	4.8	10.8
	FOLFOX4 Bevacizumab (10mg/kg/2wks) (n=289)	21.8 (p<0.0001)	7.2 (p<0.0001 HR=0.64)	12.9 (p=0.0018 HR=0.76)

* Please note the data in this table was last updated September 2012.

2.2 Evidence for Maintenance Bevacizumab

The design of this study includes 6 doses of bevacizumab given as maintenance therapy after completion of post-operative chemotherapy and bevacizumab. This will prolong exposure to bevacizumab and is intended to optimise benefit with minimal increase in toxicity.

The recently reported results of the GOG218 study which evaluated the addition of bevacizumab to adjuvant carboplatin and paclitaxel included a second randomisation to 16-cycles of maintenance bevacizumab or placebo in the investigational arm. The study demonstrated that bevacizumab is beneficial in the adjuvant setting in ovarian, primary peritoneal and fallopian tube cancer, in particular supporting the role of maintenance bevacizumab in this patient group. Median PFS in patients receiving carboplatin and paclitaxel alone was 10.3 months, with carboplatin and paclitaxel-bevacizumab followed by placebo this was 11.2 months but in patients receiving carbotaxol-bevacizumab followed by maintenance bevacizumab, the median PFS was 14.1 months, (HR 0.717, 95% CI 0.625-0.824, p<0.0001). At the time of publication, no overall survival benefit was seen for patients in this trial (19). Supporting these data, the benefit of adding bevacizumab to chemotherapy, followed by a period of maintenance bevacizumab for advanced ovarian cancer (the ICON-7 study) was recently published and, this study demonstrated an 8 months survival benefit at 42 month follow up (28.8m vs. 36.6 months in the control and experimental arms respectively for high risk patients which is encouraging in this poor prognosis population (8).

However, in resected stage II-III colon cancer, the US C-08 study failed to show a benefit from adding bevacizumab to adjuvant FOLFOX chemotherapy (20). More recently, results from the parallel AVANT study in stage III colon cancer was presented at the 2011 ASCO Gastrointestinal Symposium. There was no benefit from bevacizumab and results suggested a detrimental effect on disease-free and overall survival in this setting (21). The UK QUASAR2 study's IDMC decided that these confirmatory data were sufficient to warrant ceasing bevacizumab in patients currently enrolled to the QUASAR2 study. It should be noted however, that in contrast to the neoadjuvant breast studies and those in ovarian cancer, these colorectal trials were conducted in patients with a minimal (if any) burden of residual disease. It would appear that the effects of bevacizumab are maximized when disease burden is higher as in the neoadjuvant setting.

Nevertheless, our current understanding of angiogenesis and tumour development seems to support the concept of maintenance therapy with bevacizumab (22). Tumour growth and development of metastases are dependent on the development of tumour vasculature in order to support the metabolic requirements of the tumour cells. The development of the neovasculature appears to be controlled by an "angiogenic switch", a balance of pro-angiogenic mechanisms including VEGF and negative regulators, the latter's influence diminished by malignant transformation (23). Anti-VEGF strategies may therefore prevent tumour progression or development of metastases by inhibiting the development of the neovasculature. Resistance to therapy is not thought to be an issue as VEGF affects normal vascular endothelium with a stable non-malignant genome. Evidence that bevacizumab inhibits the development of a tumour's vasculature comes from a study by Willet *et al.* who used computer tomography (CT) to show that tumour perfusion and microvessel density were decreased after the administration of bevacizumab (24).

Evidence that bevacizumab alone is an effective therapy comes from a randomised phase II trial in renal carcinoma where 2 doses of bevacizumab were compared to

placebo. The trial was closed early after an interim analysis showed an increase in time to progression for the bevacizumab arms (25).

The REGARD trial (26) evaluating Ramucirumab (a monoclonal antibody Vascular Endothelial Growth Factor Receptor-2 (VEGFR-2) antagonist), as second line monotherapy in patients with advanced gastric cancer showed an increase in median overall survival in patients receiving Ramucirmab as compared to those receiving best supportive care, 5.2m vs 3.8m HR 0.776 (95% CI 0.603–0.998, P=0.047).

There is currently no direct evidence addressing the issue of duration of bevacizumab therapy in oesophagogastric cancer. The investigators considered the option of performing a second randomisation for those patients allocated ECX and bevacizumab between maintenance bevacizumab and no maintenance but the substantial increase in the number of patients required was unrealistic. The current trial design maximises the chance of seeing improved outcomes with bevacizumab.

2.3 ST03: Safety results from ST03 Phase II of ECX +/- B

An initial safety report of the first 200 patients randomised to the ST03 study did not demonstrate any increase in the rates of gastrointestinal bleeding or wound healing complications in the experimental arm (1). On the advice of the Independent Data Monitoring Committee (IDMC) the additional ejection fraction monitoring required for the 1st cohort of patients because of potential concerns about effects on Left Ventricular Ejection Fraction (LVEF) from the combination of bevacizumab and an anthracycline have been discontinued.

2.4 ST03: Safety results from ST03 IDMC Review of ECX +/- B data October 2013

Based on a review of cumulative data from the ST03 trial, the IDMC noted an increased risk of anastamotic leaks in a subset of patients receiving bevacizumab. Of the 255 oesophagectomies/gasto-oesophagectimes in the bevacizumab comparison the anastamotic leak rate was 9% in the sECX arm and 24% in the sECX+B arm. The IDMC looked at other factors that could explain this but it was not apparent on initial review of the data. As a precautionary measure an Urgent Safety Amendment was implemented in October 2013 to amend the inclusion criteria to exclude patients with lower oesophageal and OGJ adenocarcinomas from entering the bevacizumab comparison. The comparison remained unchanged for patients with gastric adenocarcinomas who did not require an oesophagogastrectomy. This Urgent Safety Amendment also mandated that patients with lower oesophageal and OGJ adenocarcinomas who were already entered into the sECX+B arm (Arm B) must not receive any further pre-operative bevacizumab. Following surgery patients may resume treatment with bevacizumab provided that there are no other contraindications.

These changes were implemented immediately at all centres as an urgent safety amendment as a precautionary measure. Further review of these cases will take place.

3. Trial Design and Objectives for the Bevacizumab Comparison

3.1 Trial Design

The original ST03 trial was a randomised controlled, multi-centre, open-label phase II/III trial designed to evaluate the safety and efficacy sECX chemotherapy with or without bevacizumab.

All patients from centres not participating in the lapatinib feasibility study and patients from those centres who are participating and have HER-2 negative or undefined tumours were randomised to receive either 6 cycles of peri-operative sECX chemotherapy with or without bevacizumab, plus an additional 6 cycles of bevacizumab as maintenance therapy post surgery, in a 1:1 ratio. A maximum of 1100 patients were to be recruited in to the bevacizumab comparison over 5-6 years.

Stage 1 sECX+/-B (phase II) was limited to UK centres and was completed in April 2010.

Stage 2 sECX+/-B (phase III) of the trial was open to all centres that wished to participate and met the centre accreditation criteria and has now completed recruitment.

3.2 Trial Objectives

The primary objective of the ST03 bevacizumab comparison was to assess the safety and efficacy of adding the monoclonal antibody bevacizumab to sECX chemotherapy administered peri-operatively in patients with (HER-2 negative or undefined status) operable oesophagogastric adenocarcinoma

3.3 Primary Endpoints

3.3.1 Primary Endpoint Bevacizumab Comparison

Stage 1 assessed the **safety** of the investigational arm B (sECX+B) compared with the control arm A (sECX).

This was monitored by assessing the number of:
Perforations at the site of the primary tumour and elsewhere in the gastrointestinal tract
Wound-healing related events
Gastrointestinal bleeding events
Cardiovascular events

This stage was completed in April 2010. The IDMC reviewed the data in June 2011 and agreed the trial should continue and that the additional monitoring of LVEF could be discontinued.

Stage 2 will assess whether the addition of bevacizumab to sECX results in improved OS.

3.4 Secondary Endpoints

3.4.1 Secondary Endpoints sECX + / - B comparison

Stage 1 assessed the **feasibility** of the trial in terms of:
Patient’s acceptance of the trial
How many patients are able to complete the treatment

Stage 2 will assess:
Treatment-related morbidity
Response rates to pre-operative treatment
Surgical resection rates
Disease free survival
Quality of life
Cost-effectiveness

Figure 4. Trial Schema for Patients randomised to the bevacizumab comparison (Patients with HER-2 Negative or Unknown/Undefined Tumours)

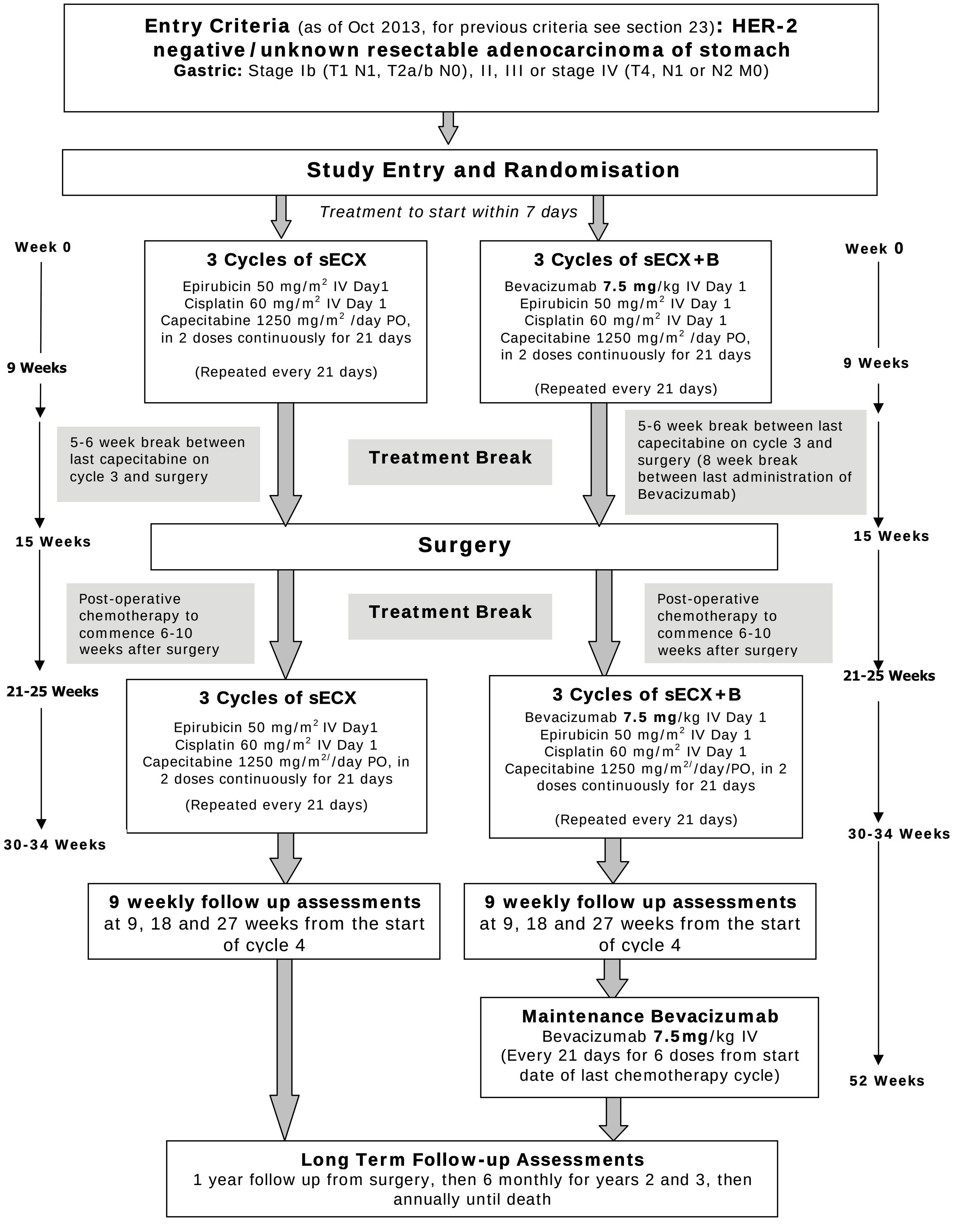

4. Selection of Patients

Please note that randomisation to the bevacizumab comparison is now complete. The information on the following pages remains only to describe the management of patients already randomised into the bevacizumab comparison.

4.1 Patient Inclusion Criteria (as of October 2013, please see Section 34 Protocol Amendments for previous eligibility criteria)

Patients with histologically verified gastric adenocarcinoma, who do not require oesphagogastrectomy and have not received any treatment for their cancer.

The inclusion criteria described below are based on the TNM classification 6th edition for gastric cancer.Should you use TNM7 for staging of a patient's disease, please see Appendix M for guidance or speak to the ST03 team at the MRC CTU.

i) Gastric Adenocarcinomas (using gastric cancer staging system)
Tumours should be Stage Ib (T1 N1, T2a/b N0), II, III or stage IV (T4 N1 or N2) with no evidence of distant metastases (M0) where the surgeon believes that an R0 resection can be achieved by excision of a contiguous structure.
Patients with linitis plastica should not be randomised.

All patients should have a CT of chest and abdomen (pelvis is optional) and laparoscopy prior to study entry. Endoscopic ultrasound (EUS) should be performed according to local practice.

The following assessments should normally be performed within 6 weeks prior to randomisation. If assessments are > 6 weeks or results are borderline please contact the ST03 Trial Manager who will refer your query to the Chief Investigator.

WHO performance status 0 or 1

Adequate cardiac ejection fraction (as determined by ECHO or MUGA scan) $\geq$ 50% or $\geq$ your centres LLN for MUGA, BP $\leq$140/90mmHg.

The following assessments should normally be performed within 1 week prior to randomisation and be as defined. If assessments are > 1 week or results are borderline please contact the ST03 Trial Manager who will refer your query to the Chief Investigator.

Adequate bone marrow function
Absolute neutrophil count (ANC) $\geq 1.5 \times 10^9$/l
White blood cell count $\geq 3 \times 10^9$/l
Platelets $\geq 100 \times 10^9$/l
Haemoglobin (Hb) $\geq$ 9g/dl (can be post-transfusion)

Adequate renal function: glomerular filtration rate (GFR) $\geq$60ml/min calculated or measured. If the calculated GFR is <60ml/min then a measured GFR is required (see

appendix I). The measured GFR should always take precedence over the calculated GFR.

Adequate liver function
serum billirubin ≤1.5x ULN
ALT/AST ≤2.5x ULN
ALP ≤3x ULN

Absence of proteinuria at baseline, defined as <2+ of protein on urine dipstick, ≤1g of protein/24 hr by a 24-hour urine collection or spot morning urine protein-creatinine ratio (UPCR) of ≤1.

Adequate Coagulation profile:
International normalised ratio (INR) ≤ 1.5
Activated ProThrombin Time (APTT) ≤ 1.5xULN

Patients on oral anticoagulation are **advised to** change to low molecular weight heparin prior to randomisation, to be eligible. **(See Section A1.3.2 Medication to be used with caution)**

Patients with high frequency hearing loss are eligible for ST03. They should be treated with cisplatin but changed to carboplatin if there is any evidence of deterioration (see Section A3.3.7).

Patient is fit to receive all protocol treatment

Completion of baseline quality of life questionnaire

Women of childbearing potential should have a negative pregnancy test within 7 days prior to commencing treatment, or have had amenorrhea for more than 2 years. Fertile men and women must agree to take adequate contraceptive precautions.

Male/female patients aged ≥18 years

Written informed consent obtained before randomisation

4.2 Patient Exclusion Criteria (as of October 2013, please see Section 23 Protocol Amendments for previous eligibility criteria)

Significant Co-Existing or Previous Medical Conditions

Cerebrovascular disease (including transient ischaemic attacks (TIA) and strokes) within 1 year before trial entry

Cardiovascular diseases as follows:
Myocardial infarction (≤ 1 year prior to randomisation)
Uncontrolled hypertension (defined as BP >140/90mmHg) while receiving chronic medication
Patients with a BP >140/90mmHg prior to randomisation should be commenced on an ACE inhibitor or other antihypertensive agent then BP re-checked a few days later, if BP is controlled to ≤140/90mmHg then the patient may be entered into the trial.
Angina requiring nitrate therapy within 1 year prior to randomization
New York Heart Association (NYHA) Grade II or greater congestive heart failure
Serious cardiac arrhythmia requiring medication (for example, ventricular tachycardia, supraventricular tachycardia or atrial fibrillation with a resting heart rate >110bpm)

Major surgery, major trauma or open biopsy within 28 days prior to study entry (not including staging laparoscopy)

Serious non-healing wound, ulcer or bone fracture

Evidence of bleeding diathesis or coagulopathy

Recent history of any active gastrointestinal inflammatory condition such as peptic ulcer disease, diverticulitis or inflammatory bowel disease. If patients have a known diagnosis of any of the above, evidence of disease control is required by negative endoscopy within the past 28 days.

Patients with mild/intermittent tinnitus can be randomised to ST03 but **patients with more severe tinnitus should not be randomised.**

Patients who have received chemotherapy or radiotherapy for a previous malignancy.

Other Exclusion Factors

Lack of physical integrity of the upper gastro-intestinal tract, malabsorption syndrome, or inability to take oral medication. Patients who require placement of enteral feeding tubes for nutrition due to inability to swallow are not eligible for randomisation. Patients who have prophylactic feeding jejunostomy tubes placed at the time of staging laparoscopy would not be excluded but this is not recommended.

Patients requiring ongoing treatment with contraindicated concomitant medication (see Section 4.3 of this appendix)

Positive serology for HIV, Hepatitis C or active Hepatitis B

Patients who have previously received anthracycline treatment

Known peripheral neuropathy ≥ Grade 1 (absence of deep tendon reflexes as the sole neurological abnormality does not render the patient ineligible)

Known dihydropyrimidine dehydrogenase (DPD) deficiency

Known allergy to Chinese hamster ovary cell proteins or other recombinant human or humanized antibodies or to any excipients of bevacizumab formulation, platinum compounds or to any other components of the study drugs

Due to an increase in perforations associated with self-expandable metal stents in patients with colorectal cancer receiving bevacizumab, patients with an oesophageal or gastric stent (metal or biodegradable) in situ are ineligible for the study.

Patients with a history of interstitial lung disease or radiological evidence of lung fibrosis are ineligible for this study.

4.3 Guidance on Concomitant Medication

4.3.1 Excluded Concomitant Medications

Any patient receiving the following concomitant medication should **not** be considered for ST03 randomisation:

Chronic, daily treatment with high-dose aspirin (> 325mg/day) or nonsteroidal anti-inflammatory drugs.
Thrombolytic therapy within 10 days prior to commencement of study treatment.
Chronic treatment with corticosteroids (dose of ≥ 10 mg/day methylprednisolone equivalent) (excluding inhaled steroids).
Dipyridamole use should be avoided and concomitant administration of capecitabine and sorivudine (or sorivudine analogues e.g. brivudine) is contraindicated. Patients receiving phenytoin concomitantly with capecitabine should be regularly monitored for increase phenytoin plasma concentrations and associated symptoms.

4.3.2 Medications to be used with caution

Patients receiving concomitant capecitabine and oral anticoagulants should have their anticoagulant response (INR or prothrombin time) monitored frequently in order to adjust the anticoagulant dose accordingly. Altered coagulation parameters and/or bleeding, including death, have been reported in patients taking capecitabine concomitantly with coumarin-derived anticoagulants such as warfarin and phenprocoumon. A pharmacokinetic interaction has been observed. The use of low molecular weight heparin instead of coumarin is advised but at the discretion of the Investigator.

It is advised that patients on the sECX+B arm should not receive oral anticoagulation (warfarin) when they are receiving chemotherapy. Warfarin is permitted when receiving bevacizumab monotherapy e.g. during the maintenance phase.

There is the possibility that previous or concomitant treatment with bisphosphonates in patients receiving bevacizumab may increase the risk of oestonecrosis of the jaw (ONJ). Patients randomised to the sECX+B Arm who have received previous bisphosphonate therapy should have a dental examination prior to commencing treatment with bevacizumab and all patients on the sECX+B Arm should be encouraged to report any symptoms of pain in the mouth, teeth or jaw, numbness or heaviness in the jaw or looseness of a tooth for further investigation.

Other cytotoxic agents or investigational drugs and radiotherapy are prohibited in this study unless there is evidence of disease progression. Any additional treatment that the responsible physician feels is appropriate is permitted.

4.3.3 Data on concomitant medication

All concomitant medication will be recorded on any subsequent Serious Adverse Event forms.

4.4 Pre-Randomisation Investigations and Screening Procedures

4.4.1 Screening Assessments

The following screening investigations should normally be performed **within 4 weeks** prior to randomisation**:**

Clinical history and examination, including blood pressure.
If BP >140/90mmHg patient should be commenced on an ACE inhibitor or other antihypertensive agent until controlled to ≤140/90mmHg prior to randomisation. (see Section 7.2.6 of this appendix for recommendations and Appendix E for guidance on treating hypertension).

ECG.

Baseline Echocardiogram (ECHO) or MUGA scan. Left Ventricular Ejection Fraction (LVEF) must be ≥50% or ≥ your centre's LLN for MUGA if this is less than 50%. There should also be an absence of other abnormal findings, such as abnormal wall motion.

Assessment of hearing and associated interpretation of results and administration of chemotherapy can be according to local practice but please note that patients with severe tinnitus **should not** be randomised.

If one or more of the above investigations cannot be performed within 4 weeks prior to randomisation, then the case should be discussed with the Trial Manager who will refer your query to the Chief Investigator.

The following assessments should normally be performed **1 week before randomisation:**

Renal function assessment. Calculated GFR as per local guidelines. If the calculated GFR < 60ml/min, or the serum creatinine is above the ULN for the local centre then a measured GFR is required. **(see Appendix I Cockroft Gault formula).**
Urine dipstick for protein. If 2+ or more protein is detected on dipstick then a 24 hr urine collection for protein should be performed to confirm that there is < 1g protein/24 hours or urinary protein-creatinine ratio should be ≤1
Baseline FBC, serum urea and electrolytes, creatinine and liver function tests and coagulation profile to include INR and APTT. (see inclusion criteria Section A1.1 for more details)

If one or more of the above investigations cannot be completed within 1 week prior to randomisation, then the case should be discussed with the Trial Manager who will refer your query to the Chief Investigator.

The above is summarised, along with the other assessments required throughout the trial in Table 8.1 ST03 Trial Assessments (**see Section 8 of this appendix**)

4.4.2 Tumour Staging Investigations

Each of the tumour staging investigations outlined below should be aimed at being performed within 4 weeks prior to randomisation. However if this is not possible, the last staging investigation, which may include CT, EUS, PET/CT or laparoscopy, **should normally be performed within 4 weeks prior to randomisation.** If the last staging investigation is >4 weeks please contact the ST03 Trial Manager who will refer the query to the Chief Investigator. The results of an EUS should be used to stage **local disease** (depth of tumour invasion and nodal status), whereas CT scan should take precedence when staging distant disease spread.

Histological confirmation of diagnosis and baseline staging investigations must be reviewed by a Multidisciplinary Team (MDT) to confirm patient's eligibility for the trial.
Staging investigations should include:
Laparoscopy.Spiral/multi-slice CT with oral contrast or water including chest, abdomen (pelvis is optional). Maximum slice width 5mm. IV contrast/venous phase.
Endoscopic ultrasound (EUS) according to local practice.
PET scans, MRI or bone scans may be used where clinically indicated according to local practice.

5. Randomisation

5.1 Written Informed Consent

Before a patient is randomised to the ST03 trial, written informed consent must be obtained. When obtaining consent from a patient, the ST03 trial and **patient information sheet (PIS) A** for patients with HER-2 negative or unknown/undefined tumours should be introduced in full. Written confirmation that the patient has given their consent to participate in the trial should be recorded on **consent form A** for patients with HER-2 negative or unknown/undefined tumours by a qualified, experienced nurse or a clinician according to local practice.

Patients who give their written informed consent to participate in the trial can only be randomised to the trial if they fulfil all the inclusion criteria and none of the exclusion criteria.

5.2 Randomisation Procedure

Once the eligibility criteria have been confirmed, it is recommended that the following CRFs are completed before calling the randomisation line as questions will be asked from each of these forms:
Randomisation form (ST03/1) (or for centres participating in the lapatinib feasibility: Randomisation form (ST03/1-HER))
Pre-treatment Screening form (ST03/2)
Baseline QoL questionnaire including EQ5D (must be completed before the patient is informed which treatment they have been allocated).

To enter a patient on the trial telephone the MRC CTU randomisation line:
+44 (0)207 670 4777 (9am - 5pm, Mon-Fri)

Centres participating in the feasibility study that are entering patients with HER-2 negative or undefined tumours into the sECX+/-B comparison should ensure that the CTU is aware that the patient has been registered previously and the patient's trial number provided at registration is stated. Confirmation of the patient initials and date of birth will be required to verify that the correct patient is being randomised.

Patients who did not consent to HER-2 testing but meet all eligibility criteria for the sECX+/-B comparison will need to be registered and randomised at the same time point in order to enter the trial. You should ensure that the CTU is aware that the patient is being registered and randomised at the same time. The Registration (ST03/0-HER) and Randomisation (ST03/1-HER) Forms must be completed before calling the CTU as the relevant information within the registration form must first be provided before going on to randomise the patient.

Upon randomisation a trial number (this will stay the same for patients previously registered) and treatment will be allocated and given over the telephone. In addition, a letter confirming these details and the patient's treatment schedule will be sent to the randomising centre. The trial number will be a unique identifier and the primary way in which the patient will be identified and should be used in all correspondence.

5.3 Patient Screening and Enrolment Log

The investigator should keep a patient screening and enrolment log of all patients being considered for the ST03 trial.

5.4 Co-enrolment Guidelines

Ideally, patients should not be participating in any other clinical trials unless this has been discussed with, and agreed to, by the ST03 TMG previously. The primary endpoint is overall survival and follow up should continue until this endpoint is met. The ST03 TMG should be contacted if co-enrolment is being considered.

5.5 Blood and Tissue Collection for Translational Research (Trans-ST03)

Participation in the Trans-ST03 study is open to all sites and strongly encouraged. In order to collect tissue and blood samples for future translational research, patients are required to sign the Trans-ST03 consent form. Although most patients are expected to consent to participation in the translational study, the wishes of patients who do not want to be involved in the translational research part of the ST03 trial will be respected and they will be allowed to enter the clinical trial only.

20ml EDTA blood should be collected from patients that consent to Trans-ST03 before commencing ST03 treatment. **For more information on the tissue and blood collection, please see Section 32 Pathology Research.**

6. Treatment of Patients

6.1 General Principles

- The Principal Investigator is responsible for ensuring that the protocol is followed for all patients treated at their centre.
- Treatment should normally commence **within 1 week** of randomisation for both treatment arms. Patients that cannot be treated within 1 week of randomisation should be discussed with the MRC CTU.
- A full blood count should be done within 5 days prior to the start of cycle 1 and either on day 1 or up to 3 days before for all subsequent treatment cycles.
- Dose modifications should only be made after consulting the protocol (if in doubt please discuss with the MRC CTU).
- Body surface area should be re-calculated before each cycle of chemotherapy. Treatment doses should be based on the re-calculated BSA value or according to local practice. Body surface area (BSA) should be calculated using the Dubois and Dubois formula or nomogram. **(see Appendix H).**
- Please note: Bevacizumab dose should remain the same for each cycle and will only be re-calculated for patients who experience a >10% change in body weight. Bevacizumab will be administered up to a maximum body weight of 135kg. (For further information see Section 6.4.1 of this appendix)
- For obese patients with a body mass index (BMI) > 30, BSA should be capped at 2.2m^2.
- Granulocyte-colony stimulating factor (G-CSF) may be used if it is local practice.
- Dose banding is allowed if it is local practice. If you need to use your local dose banding for the capecitabine, the dose banding schedule should be supplied to the MRC CTU.
- For hypertension management please see Appendix E and Section 7.2.6.

The worst toxicity grade observed during the cycle should be recorded in the Toxicity Assessment section of the cycle CRFs (ST03/3 and ST03/8).

6.1.1 Nadir Blood Test

All patients must have a nadir blood count during their cycle 1 treatment (at approximately day 10 (+/- 2 days) of treatment). **Any patients who experience Grade 4 (<0.5 x 109/L) neutropenia must start Granulocyte Colony Stimulating Factor (G-CSF).** This could be Filgrastim 300µg once daily sub-cut injection for 3-5 days, or lengograstim according to local practice. Pegylated Filgrastim (Neulasta) may also be used but should be administered at least 24 hours after intravenous chemotherapy to reduce the risk of potentiation of myelosupression. It is recommended that patients who experience Grade 3 (<1 – 0.5 x 109/L) neutropenia should also be treated with G-CSF, but this is at the treating investigator's discretion.
Cycle 1 nadir counts should be emailed to the MRC CTU when performed unless the centre has been informed that this is not required.

All patients who are treated for Grade 3-4 neutropenia in cycle 1 must also receive G-CSF during all further cycles of sECX +/- Bevacizumab. If the patient subsequently receives E-Carbo-X the G-CSF must be continued. The Investigator may wish to check the patients' nadir blood counts during future cycles, for example if the patient experiences Grade 2 neutropenia during cycle 1, however this is at the discretion of the Principal Investigator.

For those patients who experience Grade 3 or 4 neutropenia on day 1 of subsequent cycles the dose reduction recommendations within Section 6.3.3 of this appendix should be followed and GCSF can be administered as per local practice.

For those patients who are hospitalised prior to day 10 centres should follow local standard procedure to monitor neutrophil count, but if grade 4 neutropenia is documented then G-CSF is required in subsequent cycles.

6.1.2 Measures of Compliance/Adherence

Date of treatment, dose, delays and reasons for delays or dose modifications of all study drugs infusions should be recorded on the Treatment CRF (ST03/3 and ST03/8).

6.1.3 Capecitabine Diary Cards

In order to assess patient compliance in taking the full prescribed capecitabine dose per cycle, patients will be issued with a capecitabine diary card to complete for each cycle. Research staff should ensure that the diary card is collected at the end of each cycle and the number of unused tablets if any, are recorded on the treatment CRF (ST03/3 and ST03/8).

6.2 Administration of Control Arm: Standard ECX (sECX)

The control arm consists of 3 pre-operative cycles of sECX given over a period of 9 weeks at 3 weekly intervals followed by surgery 5-6 weeks after completion of pre-operative chemotherapy. 6-10 weeks later, 3 post-operative cycles of sECX are administered over 9 weeks.

Pre-operative Chemotherapy (sECX)

3 cycles of epirubicin 50 mg/m^2 IV and cisplatin 60 mg/m^2 IV Day 1 and capecitabine 1250mg/m^2/day.

The daily dose of capecitabine is based on 1250mg/m^2 /day but will be rounded to the nearest achievable dose based on three body surface area (BSA) levels and will be administered according to Table 6.2, unless a copy of your local dose banding schedule has been supplied to the MRC CTU.

The capecitabine will be administered *per oral* (PO) in 2 divided doses continuously from Days 1 to 21 per cycle (see Table 6.1 of this appendix).

Capecitabine tablets are available in 500mg and 150mg and should be administered morning and evening and swallowed with water. If the total daily dose requires uneven distribution of tablets then the larger dose should be given in the evening. Administration of capecitabine should be within 30 minutes after a meal.

Standard anti-diarrhoeal treatments (e.g. loperamide) may be used, whilst receiving capecitabine.

Each cycle is repeated every 21 days for 3 cycles.

Table 6.1: Capecitabine Dose Calculation, According to Body Surface Area

BSA (m^2)	Total daily dose (mg)	Number of tablets administered in the morning		Number of tablets administered in the evening	
		150mg	500mg	150mg	500mg
<1.6	1800	2	1	0	2
1.6-1.8	2150	0	2	1	2
>1.8	2500	0	2	0	3

***Example of how to calculate the capecitabine total cycle dose**, which is required on the Treatment CRF (ST03/3 and ST03/8) is given below.*

If BSA is 1.7, then the total daily dose prescribed will be 2150mg.

Total cycle dose is 2150 x 21 day = 45150 mg

An example of how to calculate the total daily dose of capecitabine and the number of tablets to administer in the morning and evening is given in **Appendix K.**

Problems Administering Capecitabine

For patients who have difficulty swallowing capecitabine, the tablets can be dissolved in water. Place capecitabine tablets in approximately 200ml of water. By agitating the

tablets for approximately 15 minutes, the tablets should dissolve. The tablets should be dissolved immediately before use and the solution swallowed immediately as there is no stability data for any form of suspension. The solution may also be administered through a naso-gastric or other enteral feeding tube. The solution has a very bitter taste and a fruit juice can be added to make the solution more palatable, but capecitabine should not be mixed with grapefruit juice.

If a patient vomits after taking a dose of capecitabine, the dose should not be taken again.

A recommended schedule for administrating epirubicin and cisplatin is given in Table 6.2 of this appendix. Local policy for administering sECX may be substituted if preferred, ensuring adequate hydration for cisplatin. However, the dose of cisplatin should not be split and the treatment should always be given over one day only.

TABLE 6.2 RECOMMENDED EPIRUBICIN AND CISPLATIN HYDRATION

Time (T)	Recommended Medication
T=-1 hour<br>IV chemotherapy	Patients will receive frusemide 40mg IV bolus or *po* and sodium chloride 0.9% (normal saline) 1 litre + 20mmol KCl + 10mmol $MgCl_2$ IV over 1 hour
T=0hr Prior to administration of IV chemotherapy	Patients should receive as anti-emetics Granisetron 1mg IV bolus (2mg if >100kg) and dexamethasone 8mg IV bolus
T=0hr	**Epirubicin** 50mg/m^2 will be administered as an IV bolus via fast running drip
T=0hr	**Cisplatin** 60mg/m^2 IV will be administered in sodium chloride 0.9% (normal saline) 1 litre + 20mmol KCl over 4 hours
T=0hr	Mannitol 10% 200ml should be administered IV over 4 hours concurrently with cisplatin
T=+4hr	Sodium chloride 0.9% (normal saline) 1 litre + 20 mmol KCl + 10mmol $MgCl_2$ should be administered IV over 2 hours
T=+6hr	Sodium chloride 0.9% (normal saline) 500mls + 10 mmol KCl should be administered IV over 1 hour

Oral anti-emetics:
Patients should be provided with oral anti-emetics and guidance on when they should be taken as follows:
Dexamethasone 4mg *po tds* Day 2-4
Metoclopramide 10mg *po tds* Day 1-3
Local policy for administering anti-emetics, including the use of 5-HT_3 antagonists and aprepitant, may be substituted if preferred.

Post-operative Chemotherapy (sECX)
Post-operative chemotherapy is identical to the pre-operative chemotherapy and should commence 6-10 weeks after surgery. Epirubicin 50 mg/m^2 IV and cisplatin 60 mg/m^2 IV Day 1, with capecitabine 1250mg/m^2 po daily in 2 divided doses given continuously from Days 1 to 21. Cycle repeated every 21 days for a further three cycles (see above for details).

6.3 Toxicities and Dose Modifications for sECX

Please note that if the dose of capecitabine is stopped for any reason, **the doses are omitted, not delayed** and capecitabine treatment should stop at day 21 of each cycle and not continue until all tablets have been taken. Any dose modifications that remain from a previous cycle should be indicated as modifications on all subsequent treatment CRFs.

6.3.1 Dihydropyrimidine Dehydrogenase Deficiency
With any 5FU regimen, the occasional patient is encountered (approximately 1-3%) who has markedly exaggerated toxicity due to reduced 5FU catabolism. If this occurs, await full recovery and then re-start capecitabine at a 50% reduction.

6.3.2 Haematological Toxicities
Modifications due to toxicity in previous cycle**:**
If at any time during the previous cycle, infection/fever associated with neutropenia has occurred e.g.:
CTC grade 3 (neutrophil count <1.0x10^9/l), delay all chemotherapy until neutrophil count recovers, then dose reduce epirubicin by 25% of the previous cycle.

CTC grade 4, (neutrophil count <0.5x10^9/l), delay all chemotherapy until neutrophil count recovers then dose reduce epirubicin by 50% of the previous cycle.

Dose reductions relate to the dose of the previous cycle and should be maintained in subsequent cycles.

6.3.3 Modification Based on Day 1 FBC
Check FBC on day 1 of (or up to 3 days before) each cycle.
If neutropenia/thrombocytopenia (in the absence of infection/fever) is present on the day treatment is due, delay treatment until neutrophil count recovers to ≥1.0 x 10^9/l and platelet count recovers to ≥75 x 10^9/l, then dose reduce as indicated in Tables 6.3 and 6.4 of this appendix. Dose reductions related to epirubicin should apply to all subsequent cycles.
GCSF may be used if this is local practice.

Please note that the **greatest reduction for toxicity** should be followed.
For example if there is grade 4 neutropenia and grade 2 thrombocytopenia on day 1 of treatment, capecitabine should be stopped and treatment should be delayed until blood counts have recovered. Capecitabine should be restarted at full dose, epirubicin should be reduced ***by 50%*** *and cisplatin should be given at full dose.*

TABLE 6.3: DOSE MODIFICATIONS FOR SECX BASED ON NEUTROPHIL COUNT (ON DAY OF TREATMENT)

**Neutrophil count (x10^9/l)**	**CTC grade**	**Action**
≥1.0	0-2	Full dose of all drugs.
0.5-0.9	3	Stop capecitabine and delay epirubicin and cisplatin until recovery (e.g. 1 week later). Restart capecitabine at full dose.<br>Reduce epirubicin by 25% on subsequent cycles.<br>Restart cisplatin at full dose.<br>GCSF may be used as per local practice.
<0.5	4	Stop capecitabine and delay epirubicin and cisplatin until recovery (e.g. 1 week later).<br>Restart capecitabine at full dose.<br>Reduce epirubicin by 50% on subsequent cycles.<br>Restart cisplatin at full dose.<br>GCSF may be used as per local practice.

TABLE 6.4: DOSE MODIFICATIONS FOR SECX BASED ON PLATELET COUNT (ON DAY OF TREATMENT)

**Platelet count ($x10^9/l$)**	CTC grade	**Action**
≥75	0-1	Full dose of all drugs
50-74	2	Stop capecitabine and delay epirubicin and cisplatin until recovery (e.g. 1 week later).<br>Restart capecitabine at full dose.<br>Restart cisplatin at full dose.<br>Reduce epirubicin by 25% on subsequent cycles.
25-49	3	Stop capecitabine and delay epirubicin and cisplatin until recovery (e.g. 1 week later).<br>Restart capecitabine at full dose.<br>Restart cisplatin at full dose.<br>Reduce epirubicin by 50% on subsequent cycles.
<25	4	Stop capecitabine and delay cisplatin until recovery (e.g. 1 week later).<br>Restart capecitabine at full dose.<br>Restart cisplatin at full dose.<br>Omit epirubicin from subsequent cycles.

6.3.4 Cardiac Toxicity

Cardiac Failure: Any patient who develops symptoms of heart failure (CTCAE v3 grade 3/4 left ventricular systolic dysfunction) while on treatment should be referred to a cardiologist. If during the trial the LVEF is found to be <50% on ECHO or <LLN for MUGA then further oncological management should be discussed with the CI by contacting the ST03 Trial Manager, and the patient referred to a cardiologist.

Fluoropyrimidine-related chest pain: Fluoropyrimidines are known to rarely cause a syndrome of angina like chest pain, which may be associated with coronary artery spasm.

If patients develop angina like pain whilst receiving capecitabine, then treatment should be **discontinued immediately** pending further assessment.

An ECG must be performed and serum troponin measured. Patients should be admitted overnight if significant pain has occurred within the previous 24 hours (with repeat ECGs and serial troponin).

If abnormalities are found on ECG or serial cardiac marker levels, then a cardiology opinion should be considered.

If chest pain is deemed to be capecitabine related, patients should not recommence treatment with capecitabine. The ST03 Trial Manager should be contacted and the query will be referred to the CI to discuss suitable alternatives.

Cardiac toxicities including cardiac failure, myocardial infarction and symptomatic arrhythmias are notable adverse events and should be reported to the trials unit on a SAE form.

6.3.5 Liver Toxicity

Bilirubin If bilirubin increases to >1.5xULN (upper limit of normal range), epirubicin should be omitted until bilirubin returns to acceptable levels i.e. ≤1.5xULN.

Raised Transaminases Capecitabine undergoes hepatic metabolism. Patients on capecitabine may have temporary treatment-related elevation of transaminases. An isolated rise in transaminase above 5xULN during treatment is likely to be treatment-related, and capecitabine should be interrupted until recovery i.e. ≤2.5xULN.

6.3.6 Renal Toxicity

Creatinine clearance should be calculated or measured at baseline and prior to each cycle of chemotherapy. Calculations can be made using the Cockcroft Gault formula

given in **Appendix I** or according to local practice. If the serum creatinine is above normal then creatinine clearance should be measured not estimated. The Cockcroft Gault formula usually under estimates GFR compared with EDTA or measured 24 hr creatinine clearance. Therefore if the calculated creatinine clearance falls by >25% from baseline to below 60ml/min then the measured GFR should be checked. If the measured creatinine clearance is < 60mls/min then the dose reductions of both cisplatin and capecitabine should be made according to Table 6.5 of this appendix.

TABLE 6.5: CISPLATIN AND CAPECITABINE DOSE MODIFICATIONS FOR SECX

Creatinine clearance	Cisplatin Dose	Capecitabine Dose
**≥ 60 mls/min**	100%	100%
**50-59 mls/min**	50%	100%
**40-49 mls/min**	50%	75%
**30-39 mls/min**	Replace cisplatin with carboplatin	75%

Capecitabine can resume at normal dose upon recovery of renal function.
The cisplatin dose should continue at the reduced level.

Substituting Cisplatin with Carboplatin

In patients whose creatinine clearance is <40mls/min, cisplatin should be replaced by carboplatin at a dose of AUC5 (area under the curve), so that patients receive epirubicin, carboplatin and capecitabine (E-Carb-X). Patients receiving E-Carb-X should have a neutrophil count of ≥1.5 $x10^9$/l and a platelet count of ≥100 $x10^9$/l on the day of treatment.

6.3.7 Neurotoxicity / ototoxicity

Patients with CTC grade 2 or greater neurotoxicity or new functional deterioration in hearing, new tinnitus or new significant high frequency hearing loss on audiogram should have cisplatin replaced with carboplatin at a dose of AUC5, so that patients receive epirubicin, carboplatin and capecitabine (E-Carb-X).

6.3.8 Plantar-Palmar erythema (PPE)

For CTC grade 2, stop capecitabine. On resolution of toxicity to ≤ grade 1, restart capecitabine with 15% dose reduction.
For CTC grade 3, stop capecitabine. On resolution of toxicity to ≤ grade 1, restart capecitabine with 30% dose reduction.
For recurrent CTC grade 3, stop capecitabine. On resolution of toxicity to ≤ grade 1, restart capecitabine with 50% dose reduction.
When capecitabine is stopped for toxicity the doses are omitted, not delayed.

6.3.9 Stomatitis, Diarrhoea, Nausea and Vomiting

For CTC grade 2-3 toxicity, stop capecitabine and administer appropriate symptomatic management (e.g. sucralfate for stomatitis, codeine phosphate for diarrhoea). If toxicity is adequately controlled with symptomatic measures alone within 2 days, then capecitabine may be restarted at 100% full dose. If toxicity persists, dose reductions as indicated in Table 6.6 of this appendix should be made. Doses of capecitabine should be rounded to the nearest 150mg tablets.

When capecitabine is stopped for toxicity the doses are omitted, not delayed.

Table 6.6: Management of Stomatitis, Diarrhoea, Nausea and Vomiting

Incidence	Grade 2	Grade 3	Grade 4
1st appearance	Interrupt treatment until resolved to grade 0-1, then continue capecitabine at same dose.	Interrupt treatment until resolved to grade 0-1, then continue capecitabine at 75% of original dose with prophylaxis where possible.	Discontinue treatment (if the PI considers it to be in the best interest of the patient to continue at 50% of original dose, once toxicity has resolved to grade 0-1 this should first be discussed with the MRC CTU who will confirm this with the CI.)
2nd appearance of same toxicity	Interrupt treatment until resolved to grade 0-1, then continue capecitabine at 75% of original dose.	Interrupt treatment until resolved to grade 0-1, then continue capecitabine at 50% of original dose.	Discontinue treatment
3rd appearance of same toxicity	Interrupt treatment until resolved to grade 0-1, then continue capecitabine at 50% of original dose.	Discontinue treatment	N/A
4th appearance of same toxicity	Discontinue treatment	N/A	N/A

6.4 Administration of the Investigational Arm: sECX+B

The investigational arm consists of 3 pre-operative cycles of sECX as in the control arm, but with the addition of **7.5 mg/kg IV** bevacizumab administered on day 1 of each cycle, either before or after the administration of the chemotherapy*. The pre-operative treatment is given at 3 weekly intervals over a 9 week period. This is followed by an 8 week break between surgery and the last injection of bevacizumab given at the beginning of cycle 3. Post-operative treatment commences 6-10 weeks after surgery, 3 cycles of sECX, plus 7.5 mg/kg IV bevacizumab are administered in the same way as the pre-operative treatment. Patients will then go on to receive 6 maintenance injections of bevacizumab given at the same dose of 7.5 mg/kg IV over 18 weeks e.g. one injection every 3 weeks.

If chemotherapy is delayed then the bevacizumab must also be delayed accordingly.

From October 2013, patients with lower oesophageal or Siewert Type I, II or III OGJ tumours randomised to the sECX+B arm (Arm B) who are currently receiving, or about to receive, pre-operative treatment with bevacizumab must not receive any further pre-operative bevacizumab. Following surgery patients may resume treatment with bevacizumab provided that there are no other contraindications.

6.4.1 Administration of Bevacizumab

The summary of product characteristics (SPC) for bevacizumab recommends that bevacizumab infusions should be used within 24 hours and stored between 2-8°C. Any unused portion of bevacizumab left in a vial should be discarded as the product contains no preservatives.

Bevacizumab may be given before or after chemotherapy treatment. No routine premedications, including anti-emetics, are required.

All patients should commence **lansoprazole 30mg od po**, or an alternative proton pump inhibitor (PPI), from day 1 of the first cycle of treatment. This should continue until 21 days after the final dose of Bevacizumab. Patients who have had a total gastrectomy do not need to continue the PPI beyond their surgery. Patients who are allergic to, or intolerant of PPIs, should be commenced on ranitidine 150mg bd po as an alternative, to prevent peptic ulceration.

The necessary amount of bevacizumab for a dose of 7.5 mg/kg of body weight should be withdrawn from one of the single use vials. The patient's actual weight from the baseline visit will be the reference weight throughout the study (i.e. patients will receive the same dose at each treatment). Doses of bevacizumab will be recalculated for patients who experience more than a 10% change in body weight from baseline during the treatment period. Bevacizumab will be administered up to a maximum body weight of 135 kg.

Bevacizumab should be diluted in a bag containing a total of 100ml of 0.9% sodium chloride (and never in dextrose or glucose solutions), Bevacizumab is compatible with PVC (polyvinylchloride) or polyolefine infusion bags and set.

Bevacizumab should be administered as a continuous intravenous infusion via a rate regulating device. The total infusion time should always be 90, 60, or 30 minutes according to how many previous bevacizumab infusions have been received and how they were tolerated.

The first infusion should be administered over 90 minutes.

The second infusion can be administered over 60 minutes, if there were no infusion associated adverse events (e.g. fever/chills) with the first administration.

Third, and all subsequent cycles should be given over 30 minutes, if the 60 minute infusion was well tolerated
See Section 6.4.7 of this appendix for management of bevacizumab infusion related events.
When the infusion bag containing bevacizumab is empty, 50ml of 0.9% sodium chloride should be added to the IV bag, or an additional 50ml bag of 0.9% sodium chloride administered, and the infusion continued for a volume equal to that of the tubing to ensure complete delivery of the trial drug. The sodium chloride infusion is not included in the trial drug infusion time.

6.4.2 Guidance on Omitting Doses of Bevacizumab

Bevacizumab doses will not be reduced and missed doses of bevacizumab will not be made up.
If more than 2 consecutive doses of bevacizumab are missed due to adverse events, treatment with bevacizumab should be stopped and treatment continued with chemotherapy only if there is no contraindication to doing so. However, bevacizumab may be reintroduced if it was ceased following reduction in LVEF if recovery to the normal range is observed at the next cardiac monitoring scan. If there are any queries about restarting the bevacizumab, please contact the MRC CTU who can discuss the query with the Chief Investigator.

6.4.3 Pre-operative Chemotherapy: sECX+B

Please note that the sECX chemotherapy in both the control arm and the investigational arm are identical. Full details including the hydration and anti-emetic and dose modifications can be found in **Sections 6.2 and 6.3 of this appendix.**

3 cycles of epirubicin 50 mg/m^2 IV and cisplatin 60 mg/m^2 IV Day 1 and capecitabine *po* 1250mg/m^2/day.
The capecitabine is available as 150mg and 500mg tablets only and will be administered *po* in 2 divided doses continuously from Days 1 to 21 per cycle (see Table A3.1).
Capecitabine tablets should be administered morning and evening and swallowed with water within 30 minutes after a meal. If the total daily dose requires uneven distribution of tablets then the larger dose should be given in the evening.
Standard anti-diarrhoeal treatments (e.g. loperamide) may be used, whilst receiving capecitabine.
Each cycle is repeated every 21 days for 3 cycles, followed by surgery 5-6 weeks after completion of pre operative chemotherapy (last administration of capecitabine on cycle 3).

Please note as stated in Section 6.4.1 of this appendix all patients should be commenced on a proton pump inhibitor (PPI) from day 1 of the first cycle of treatment until 21 days after the last dose of bevacizumab. Patients who have a gastrectomy do not need to continue the PPI beyond surgery. (see Section 6.4.1 of this appendix for further details)

6.4.4 Problems Administering Capecitabine

For patients who have difficulty swallowing capecitabine, the tablets can be dissolved in water. Place capecitabine tablets in approximately 200ml of lukewarm water. By agitating the tablets for approximately 15 minutes, the tablets should dissolve. The tablets should be dissolved immediately before use and the solution swallowed immediately as there is no stability data for any form of suspension. The solution may also be administered through a naso-gastric or other enteral feeding tube. The solution has a very bitter taste and a fruit juice can be added to make the solution more palatable, but capecitabine should not be mixed with grapefruit juice.

If a patient vomits after taking a dose of capecitabine, that dose should not be taken again.

6.4.5 Intervals between Peri-operative Treatment and Surgery

Patients will have a 5-6 week break between pre-operative chemotherapy and surgery (that is 5-6 weeks from last administration of capecitabine and 8-9 weeks from last administration of bevacizumab in cycle 3). Post-operative treatment should *commence* 6-10 weeks after surgery. These intervals should be the same in both arms and are necessary to minimize the potential for increased peri-operative risk that may be related to chemotherapy or bevacizumab.

If at 10 weeks post surgery, the Principal Investigator's assessment is that a patient remains unfit to commence chemotherapy, then this should be reported to the Trial Manager at the MRC CTU. Patients on the experimental arm may be allowed to commence bevacizumab alone, after the Trial Manager has discussed the patient's situation with the Chief Investigator.

6.4.6 Bevacizumab Maintenance Treatment

After completion of post-operative sECX plus bevacizumab, patients in this arm will receive a further 6 doses of bevacizumab at 3 weekly intervals, over a total of 18 weeks. The first maintenance dose of bevacizumab should start in the week after the last capecitabine tablet was taken or 3 weeks after the last bevacizumab which was given as combination treatment with sECX. This will be administered at the same dose as peri-operative of **7.5 mg/kg IV** every 3 weeks.

If bevacizumab is omitted due to toxicity then this should be missed and not administered at a later date.

Please note that the patient should continue to receive PPI treatment until 21 days after the last dose of bevacizumab unless a total gastrectomy was performed at surgery (see Section 6.4.1 of this appendix for more details)

6.4.7 Bevacizumab Infusion-Related Events

Reactions to Bevacizumab are extremely rare and the suggested premedication management is not a proven way of managing this problem, local standard practice would also be an acceptable alternative.

In clinical studies, infusion reactions with the first dose of bevacizumab were uncommon (< 3%) and severe reactions occurred in 0.2% of patients.

If mild (grade 1 or 2) infusion-related reactions (e.g., fever, chills, headache, nausea) occur, pre-medications (e.g. paracetamol 1g *po* and chlorpheniramine 10mg IV) should be given with the next dose, but the infusion time may not be reduced for that subsequent infusion. If the next dose is well tolerated with pre-medication, the subsequent infusion time may be reduced by 30 +/- 10 minutes as long as pre-medication continues to be used. If infusion-related adverse events (AE) occur with the 60-minute infusion, all subsequent doses should be given over 90 +/- 15 minutes (with pre-medication). Similarly, if mild infusion-related AEs occur with the 30-minute infusion, all subsequent doses should be given over 60 +/- 10 minutes with pre-medications such as paracetamol 1g *po* and chlorpheniramine 10mg IV.

For patients with grade 3 infusion-related reactions, the bevacizumab infusion should be discussed with the ST03 Trial Manager who will refer the query to the Chief Investigator. Adequate information on re-challenge of bevacizumab is not available. At the Principal Investigators discretion, bevacizumab may be permanently discontinued or re-instituted with pre-medications and at a rate of 90 +/- 15 minutes. If the reaction occurred at the 90-minute rate, initially challenge at a slower infusion rate and gradually decrease to 90 minutes. In case of any doubt bevacizumab should be

permanently discontinued. When bevacizumab is re-instituted, the patient should be monitored for duration comparable to duration of reaction.

For patients with grade 4 infusion-related reactions, bevacizumab should be permanently discontinued.

6.4.8 Bevacizumab Allergic Reactions

Anaphylaxis: Anaphylaxis is defined as vascular collapse and shock (blood pressure <90 mm Hg that is unresponsive to IV fluids) believed to be allergic in origin, with or without antecedent respiratory distress and occurring within 30 minutes of initiation of bevacizumab infusion. Cutaneous manifestations include pruritus, urticaria, or angioedema. Anaphylaxis has not been observed in 1032 patients treated with bevacizumab in Genentech-sponsored trials.

Patients experiencing allergic reactions should be administered appropriate medical therapy and discontinue bevacizumab.

7. General Information on Adverse Events Associated with Bevacizumab

Several thousands of patients have been treated with bevacizumab either as a single agent or in combination with chemotherapy. The overall safety profile of bevacizumab, to date, is based on clinical trials in 20,339 patients with various advanced malignancies (metastatic carcinoma of the colon or rectum, adjuvant colon cancer, locally advanced or metastatic non-small cell lung cancer, locally recurrent or metastatic breast cancer, epithelial ovarian cancer and primary peritoneal cancer, metastatic renal cell carcinoma, pancreatic cancer and hormone-resistant prostate cancer, and malignant glioma), and on 3867 patients in two post-marketing studies of bevacizumab in mCRC and NSCLC. Full details are provided in the Investigator's Brochure.

The most common side effects across all clinical trials in patients receiving bevacizumab, with or without chemotherapy, were asthenia, diarrhoea, nausea, epistaxis, hypertension, proteinuria and pain.

The most serious side effects seen with bevacizumab were:

- Hypertension
- Proteinuria
- Arterial thrombosis
- Wound healing complications
- Gastrointestinal perforations
- Bleeding events
- Fistulae
- Venous thrombosis

Therefore the above events have been designated as **notable adverse events** in this trial and should be reported on an SAE form regardless of whether they fulfil the standard definitions of serious (given in Section 6). **Please see Section 17.4 of the main protocol for detailed instructions on reporting the ST03 notable events.**

Other toxicities include diarrhoea, nausea, pain, asthenia and epistaxis. **(See Appendix L Summary of product Characteristics).**

7.1 Specific toxicities requiring discontinuation of bevacizumab

The long half-life of bevacizumab (20 days) means that the discontinuation of bevacizumab in the case of adverse events is not expected to influence the short-term clinical evolution of the event and therefore, the management of adverse events is based on symptomatic treatment.

Any patient who develops any one of the following toxicities attributable to bevacizumab **should not** receive further bevacizumab:

- Any case of gastrointestinal perforation
- Any grade of arterial thromboembolic events (including transient ischaemic attack (TIA), cerebrovascular accident (CVA) and myocardial infarction (MI) or new diagnosis of ischaemic heart disease during study treatment)
- Grade 3/4 haemorrhagic events
- Grade 3/4 congestive heart failure or left ventricular dysfunction (symptomatic heart failure, LVEF <40%)
- Grade 4 hypertension (hypertensive crisis)
- Grade 4 proteinuria (nephrotic syndrome)
- Any grade of tracheo-oesophageal fistula or grade 4 fistula of any origin (for ≤ grade 3 fistula of non tracheo-oesophagel origin, recommencing bevacizumab after resolution must be discussed with the Chief Investigator via the Trial Manager.)

For the ST03 recommendations in the event of a Grade 4 Venous Thromboembolic Event please refer to section 7.2.5.

The events above require expedited reporting and discontinuation of bevacizumab, please see Section 17.4 of the main protocol for more details as to the requirements for reporting notable events within the ST03 trial.

If a patient develops a second episode of proteinuria, hypertension or venous thromboembolic event as a grade 3 toxicity then the principal investigator should not re-start bevacizumab without first discussing this with the ST03 Trial Manager who will discuss the patient's situation with the Chief Investigator.

7.2 Specific Severe Adverse Events of Bevacizumab and Recommendations for Treatment

7.2.1 Gastrointestinal Perforation

Evidence: In the Hurwitz study (11) when bevacizumab was combined with irinotecan and 5FU, 402 patients with advanced colorectal cancer received bevacizumab, approximately half of whom had had their primary bowel cancer in situ, 6 perforations were observed (1.5%) compared to none in the control arm. The First BEAT study reported a GI perforation rate of (4%, n=8) with unresected primary tumors: however only 3 of them occurred at the primary tumor site (27). In the BRiTE study GI perforation rate was 3% in patients with an intact primary versus 2% for resected primaries (28).

In the gastric cancer study reported by Shah *et al*. (16) one perforation and one near perforation were observed out of 26 patients assessable for toxicity. The former occurred in a patient who had stopped treatment due to progression of disease, one month after cessation of treatment, after their tumour progressed to full thickness and perforated. The latter occurred after 2 cycles of treatment at the site of disease. In the

AVAGAST study of advanced gastric cancer, GI perforations occurred in 2.3% (n=9) of patients in the bevacizumab containing arm, and 0.5% (n=1) in the placebo containing arm. These figures are commensurate with rates seen in colorectal cancer.

Data from the phase II safety component of ST03 demonstrates that the risk of gastrointestinal perforation is low both with ECX (2/101 patients, 2%) and with ECX+B (1/99 patients, 1%).

Overall, the investigators assess the risk of perforation as a real but small risk. Patients particularly at risk are those with intra-abdominal inflammation e.g. gastric ulcer, tumour necrosis or diverticulitis. Patients therefore need to be carefully monitored during treatment and made aware of this risk so that they know to seek medical attention if any suggestive symptoms occur. As patients entering this trial will all have endoscopies as part of pre-treatment assessment, patients with non-malignant ulceration will be excluded due to this possible risk. Gastrointestinal perforation is subject to expedited reporting as a notable event. **(see Section 17.4 of the main protocol).**

ST03 Recommendation: Any cases of gastrointestinal perforation should be managed aggressively, usually operatively, with the involvement of the gastrointestinal surgical team in the usual manner. Bevacizumab must be ceased indefinitely. The surgical team must be informed that the bevacizumab may potentially compromise wound healing.

7.2.2 Impaired Wound Healing

Evidence: Due to the anti-angiogenic properties of bevacizumab, there are concerns about its potential for impairing healing and increasing peri-operative complications. In the Hurwitz study (11) 173 out of the 402 patients treated with bevacizumab proceeded to surgery either due to emergencies unrelated to treatment administered, or because their disease had become resectable after tumour response. The incidence of bleeding and wound healing-impairment related events were both less than 2%, and no different from similar patients in the chemotherapy only arm. The investigators of this study recommend that to minimize the risk of complications, surgery should occur no sooner than 60 days after last bevacizumab administration and should not recommence till at least 28 days has elapsed after surgery. This is supported by a pharmacokinetic study which has shown the half-life of bevacizumab to be about 20 days (29), so that several half-lives would have elapsed during the above time span. Similarly low rates of wound healing complicatios were were seen in the adjuvant C-08 study in resected Stage II and III colon cancer, where wound healing complications were seen 1.7% of patients in the bevacizumab containing arm vs. 0.3% in the chemotherapy alone arm of the study (30). Additionally, of these wound healing complications more than half (14/23) were symptomatic abdominal wall hernias and only half of patients with a wound healing complication were required to stop bevacizumab therapy.

Wound infections were seen in 8% of patients treated with sECX and 6% of those treated with sECX+B during the phase II safety component of ST03 (≥Grade 3 were 4% for ECX and 1% for ECX+B respectively). Similarly, no increase in non-infectious wound complications were seen with the addition of bevacizumab (4% ECX vs. 2% ECX + B), nor was any difference seen in the rate of anastamotic leak between the arms at 4% in both. However a later analysis showed leak rates of 9% vs 24% in patients undergoing oesophagectomy/gastro-oesphagectomy after sECX vs sECX+B respectively.

ST03 Recommendation: As bevacizumab therapy may adversely affect wound healing it is recommended that at least 8 weeks elapse between the last bevacizumab injection and surgery. The Urgent Safety Amendment in October 2013 mandated that all patients with lower oesophageal and OGJ tumours entered into the sEXC+B arm (Arm B) have no further bevacizumab with pre-operative chemotherapy due to concerns over increased leak rates in the bevacizumab arm in this subset of patients as detailed above. Following surgery patients may resume treatment with bevacizumab provided that there are no other contraindications, chemotherapy and bevacizumab should not recommence until at least 6 weeks after surgery or until the wound is fully healed. If the wound is still not healed by the time the fourth cycle of chemotherapy is due, the principal investigator should contact the ST03 Trial Manager who will discuss the course of action to be taken with the chief investigator.

For patients requiring surgery whilst receiving bevacizumab treatment surgeons should be aware that there is an increased risk of impaired wound healing and careful consideration should be given to the benefits and risks of any potential operation. Whether the unplanned surgery is related either to the tumour (e.g. perforation or bleeding) or to an unrelated cause bevacizumab should be discontinued permanently once a decision to operate has been taken. For patients who undergo emergency surgery for a tumour related complication further surgery may be deemed appropriate after discussion by the local oesophago-gastric MDT. It is preferable that if a perforation at the tumour site has occurred surgery should be performed by a designated upper GI surgeon, if possible the surgeon who has already been involved with the patient's initial assessment.

7.2.3 Gastrointestinal Bleeding

Evidence: In phase I/II studies of bevacizumab (31) gastrointestinal bleeding was observed. On this basis, patients in the Hurwitz study (11) were monitored for this potential complication but there was no evidence of a difference between the arms. Epistaxis was a more commonly observed occurrence, but was easily managed by conventional treatment. No excess gastrointestinal bleeding was seen in the bevacizumab arm of the AVAGAST study, the rate of which was 2% in both arms (7).

GI haemorrhage was uncommon in the phase II safety component of ST03, with rates from 4% (ECX) to 1% (ECX+B).

ST03 Recommendation**:** If Grade 3/4 bleeding occurs appropriate treatment should be instituted and bevacizumab treatment will be discontinued permanently. An exception to this is blood transfusions that occur post-operatively without exceptional blood loss which will continue to be classified as Grade 3/4 haemorrhage and will continue to be reported as a notable event, however bevacizumab need not be discontinued in these circumstances.

7.2.4 Risk of Cardiotoxicity

Evidence: There are currently limited data to assess whether bevacizumab has cardiotoxic effects, particularly when used with other agents which are known to be cardiotoxic, for example the anthracyclines and fluoropyrimidines.

In a phase III study in patients with previously treated advanced breast cancer who were treated with either capecitabine or capecitabine plus bevacizumab, 2 (out of 215) and 7 (out of 229) patients in each arm respectively developed either grade 3 or 4 cardiotoxicity (cardiac failure or cardiomyopathy) (9). This difference was not statistically significant. Furthermore, the cardiotoxicity observed could have been due to reasons other than bevacizumab. All of these patients had received prior

anthracycline treatment (doxorubicin within the cumulative dose range of 240-360 mg/m^2), some would have received prior radiotherapy to the left chest wall, and approximately half of the patients with cardiotoxicity in each group had documented abnormal left ventricular ejection fractions prior to study entry.

More recently, the results of a phase II study in soft tissue sarcoma were reported in which anthracycline-naïve patients were treated with bevacizumab in combination with doxorubicin 75 mg/m^2 every 3 weeks (32). In this study, 6 patients out of the 17 treated had grade 2 or greater cardiotoxicity. Four of these had asymptomatic reductions in ejection fraction (grade 2; cumulative doxorubicin dosing of 75-300 mg/m^2), which were only detected due to protocol specified cardiac function testing after every 2 cycles of treatment. The remaining patients had grade 3 and 4 cardiotoxicity respectively (cumulative doxorubicin doses of 591 mg/m^2 and 420 mg/m^2). Bevacizumab has also been added to the doxorubicin containing R-CHOP regimen in non-Hodgkin's lymphoma in a phase II study. One grade 2 reduction in cardiac function has been detected in this 12 patient cohort since the initial reporting of the results of this study (33).

In contrast, a far greater number of patients have been treated with bevacizumab in various colorectal cancer studies, most of which have also included a fluoropyrimidine, with no cardiotoxicity observed. Overall, it is likely that there is a low risk of cardiotoxicity arising from the addition of bevacizumab to the ECX regimen, as in this study protocol. Although there are no data to suggest a dose relationship between bevacizumab dose and risk of cardiotoxicity, all three of the above mentioned studies used a higher dose of bevacizumab than this study (15 mg/m^2 every 3 weeks). In addition, the cardiotoxic potential of epirubicin is approximately half that of doxorubicin, so that the total dose of epirubicin of 300 mg/m^2 which patients might receive in this study is likely to be safe.

In the phase II safety component of ST03 additional cardiac monitoring of LVEF has been discontinued based on an IDMC review in June 2011. Based on the definition advised by the cardiologist for the IDMC reviews, a significant fall in LVEF was defined as a ≥10% decrease to <lower limit of normal (LLN) and 8/90 (8.9%) ECX and 13/85 (15.3%) ECX-B patients met the criteria. Of these, 2 ECX and 4 ECX-B patients had a LVEF <50% at the end of post-operative chemotherapy, all recovered to >50% 3 months later. One patient experienced CCF in the context of sepsis, MI and LV thrombus (LVEF 30% recovered to 50% 3 months later). No symptoms of CCF had been recorded in the remaining twenty patients with a documented significant asymptomatic fall in LVEF with median 22 months follow-up from randomisation.

ST03 Recommendation: Cardiotoxicity has been designated as a notable adverse event in this protocol in order to encourage reporting of any events, which might occur during the study. Due to the potential for increasing arterial thromboembolic risk, patients with unstable cardiac disease, myocardial infarction and cerebrovascular disease (transient ischaemic attacks and strokes) within the last 12 months will not be eligible. Bevacizumab should be permanently ceased in patients developing congestive cardiac failure, myocardial infarction or cerebrovascular disease. Patients diagnosed with new ischaemic heart disease during the study should also permanently cease bevacizumab.

7.2.5 Thrombosis/Embolism

Evidence: An analysis of 5 completed trials with a total of 1798 patients of bevacizumab and chemotherapy found the incidence of arterial thrombotic events (cerebrovascular accidents (strokes), transient ischaemic episodes, subarachnoid haemorrhages, myocardial infarctions and angina) to be 3.8% for patients receiving chemotherapy and bevacizumab compared to 1.7% for those receiving chemotherapy

alone. Mortality from these events was 0.8% compared to 0.4%. Patients > than 65 years and those with a previous history had an increased risk (34). However, a more recent meta-analysis of 6055 patients in 10 randomised clinical trials demonstrated no statistically significant increases in the unadjusted or exposure-adjusted incidences of all-grade VTEs for bevacizumab versus controls in the overall population or by tumor type (35).
Similarly, in AVAGAST there was no increase in VTE in the bevacizumab arm of the study compared to placebo/ Rates of arterial thrombosis were increased (2% vs 1%), although these remained at numerically low rate (7).

***ST03 Recommendation**:* Patients who develop the following grades of thrombosis /embolism must discontinue bevacizumab and the following action is recommended:

- Bevacizumab should be **permanently discontinued** in patients who develop any grade of arterial thromboembolic event (e.g. cerebrovascular accidents, transient ischaemic attacks, myocardial infarctions).
- Venous thromboembolic event – Grade 3 (DVT) or Grade 4 (symptomatic or asymptomatic Pulmonary Embolus (PE)) (first occurrence): stop bevacizumab upon diagnosis.

  Bevacizumab may be resumed after initiation of therapeutic-dose anticoagulant therapy as soon as all of the following criteria are met:

  (i) The patient should preferably be treated with low molecular weight heparin.

  (ii) The patient must not have had a Grade 3 or 4 haemorrhagic event since entering the study.

  (iii) Patient has been anti-coagulated and has become asymptomatic from a previous symptomatic PE.

Please refer to medications to be used with caution within Appendix S for guidance on the use of oral anticoagulants.**Please note that Grade 4 Venous Thromboembolic Event is considered as a notable event within this trial and should be reported on an SAE form (see Section 17.4 of the main protocol for more details)**

7.2.6 Hypertension

Evidence: Hypertension is the most common side effect seen in trials of chemotherapy and bevacizumab. The incidence of grade 3-4 hypertension may be up to 10-15% and 0.7% of patients have stopped bevacizumab due to hypertension (36). In the gastric cancer AVAGAST study the rate of ≥ grade 3 hypertension was 6% in patients receiving bevacizumab. Grade 3 hypertension is defined as an elevation of blood pressure that requires more than one drug or more intensive therapy than previously. Grade 4 toxicity is defined as hypertension with life-threatening consequences (e.g. hypertensive crisis).

The mechanism of bevacizumab-induced hypertension is not known but it has been suggested that it is related to decreased production of nitric oxide. VEGF inhibition results in decreased nitric oxide production, a known vasodilator. This could lead to vasoconstriction and increased blood pressure.

ST03 Recommendations: At this stage there is no information on the effect of bevacizumab in patients with uncontrolled hypertension at the time of initiating bevacizumab therapy. Patients with known hypertension are therefore only eligible if their hypertension is controlled, with or without the aid of hypertensive therapy, at the

time of screening/randomisation. Patients with uncontrolled hypertension (persistently >140/90mmHg) despite medical therapy will be ineligible for ST03.

Patients with a BP >140/90mmHg prior to randomisation should be commenced on an ACE inhibitor or other antihypertensive agent then BP re-checked a few days later, if BP is controlled to ≤140/90mmHg then the patient may be entered into the trial.

Patients should be monitored for the development or worsening of hypertension via frequent blood pressure measurement (at every visit). Blood pressure measurements should be taken after the patient has been in a resting position for ≥5 minutes. Repeated measurement of blood pressure for verification should be undertaken if the initial reading is above 140 mmHg systolic and/or 90 mmHg diastolic blood pressure. **(See Appendix E: Guidance on Hypertension)**

An angiotensin converting enzyme inhibitor (ACE inhibitor) should be used as first line to treat hypertension (e.g. perindopril starting dose 2mg daily) unless contraindicated. This should be titrated against blood pressure in consultation with the patient's general practitioner. Patients should have their renal function and electrolytes checked prior to ACE inhibitor initiation and 1 to 2 weeks after initiation or dose titration.

For patients who develop the following grades of hypertension the following action is recommended:

Grade 1 hypertension: Asymptomatic, transient (<24 hrs) increase by >20 mmHg (diastolic) or to > 140/90 mmHg if previously within normal limits.
Action: Re-check BP 1 hour later:

- If BP ≤140/90mmHg, bevacizumab can be given and no further action is indicated.
- If BP remains >140/90mmHg but is <150/100mmHg, bevacizumab may be administered but BP should be re-checked 48 hours later. If BP remains >140/90mmHg (Grade 2) antihypertensive therapy should be commenced.
- If BP ≥150/100mmHg omit bevacizumab and recheck BP 48 hours later. Commence antihypertensive therapy if BP remains >140/90mmHg (Grade 2)

Grade 2 hypertension: Recurrent or persistent (> 24 hr) increase by 20 mmHg (diastolic) or to > 140/90 mmHg if previously within normal limits.
Action: Anti-hypertensive therapy should be commenced. Once controlled to ≤140/90 mmHg, patients may continue bevacizumab therapy.

Grade 3 hypertension: Requiring more than one anti-hypertensive or more intensive therapy than previously.
Action: Bevacizumab should be withheld for persistent hypertension >140/90 mmHg and should be permanently discontinued if hypertension is not controlled.

Grade 4 hypertension: Life threatening consequence (e.g. hypertensive crisis).
Action: Occurrence of Grade 4 hypertension is a medical emergency and should lead to **permanent discontinuation** of bevacizumab.

7.2.7 Proteinuria

Evidence: Proteinuria is usually asymptomatic and the incidence in a population of 1132 patients with a number of different types of cancer was 23% (36). Patients with a history of hypertension may be at increased risk for the development of proteinuria when treated with bevacizumab. There is also evidence suggesting Grade 1 proteinuria may be dose-related to bevacizumab (11)

ST03 Recommendations: All patients require a urine dipstick for protein before entering the trial. 24 hour urine collection or spot morning urine protein-creatinine ratio (UPCR) are only required in patients with 2+ or more of protein on urine dipstick. From then on monitoring of proteinuria will be assessed by dipstick urinalysis up to 3 days prior to each bevacizumab dose.

- If <2+proteinuria bevacizumab can be administered as planned
- If 2+ or 3+ proteinuria follow algorithm in **Figure 7**
- If nephrotic syndrome, grade 4 proteinuria, occurs then bevacizumab should be permanently discontinued.

Please note that Grade 4 proteinuria is considered as a notable event within this trial and should be reported on an SAE form (see section 6.4 for more details)

Figure 7: Algorithm for Proteinuria (dipstick) ≥ 2+

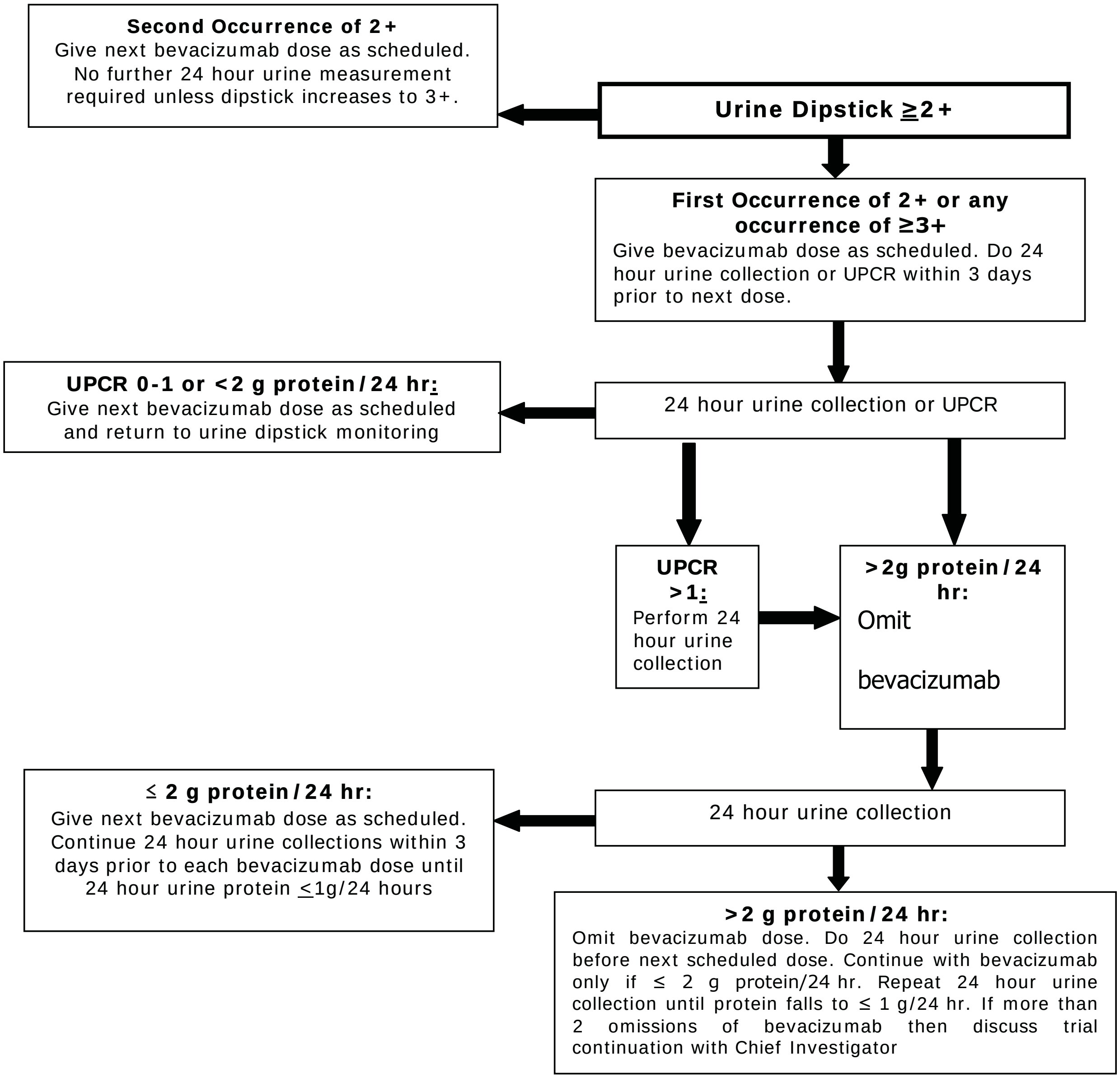

7.2.8 Fistula or intra-abdominal abscess

Evidence: Fistula formation is a recognised 'common' side effect of bevacizumab (occurring in more than 1 in 100 patients, but less than 1 in 10). These are most often gastrointestinal in origin, but may originate from other sites including the genitourinary system and rarely the trachea and/or oesophagus.

ST03 Recommendation: Any ST03 patient who develops any of the following must cease bevacizumab permanently:

A **grade 4 fistula** of any origin, irrespective of causality to bevacizumab
Any grade of fistula possibly, probably or definitely related to bevacizumab
Any grade of tracheosophageal fistula.
Any ST03 patient who develops any other fistula deemed by the Principal Investigator to be unrelated to bevacizumab, and to be related to surgery or the disease process itself, must have bevacizumab ceased at least until the fistula resolves completely (by either conservative management or surgery if related to surgery). At this point, if it is thought to be in the patient’s best interest to continue with bevacizumab, then this must be discussed with the ST03 Trial Manager who will refer the query to the Chief Investigator. Bevacizumab cannot be restarted without prior formal approval.
It is recognized that causality in these cases may be more difficult to assign with certainty, and in these cases bevacizumab will be ceased permanently.

Fistulae formations in patients up to 2 years from the start of treatment will be collected as notable events. Please refer to the notable events Section 6.4 for further details.

For Serious Adverse Event reporting guidelines please refer to Section 17 in the main protocol.

8. Assessments and Procedures required within the sECX+/-B Comparison

Summaries of the timing of assessments and CRF data return are given in Tables 8.1, 8.2 and 8.3 of this appendix.

For details on the pre-randomisation assessments, see Section 4.4 of this appendix Pre Randomisation Investigation and Screening Procedures.

8.1 Assessments Required Prior To Each Cycle of Treatment with sECX +/- Bevacizumab

Toxicity Assessment (Day 1, or up to 3 working days before)
Patients will be assessed for the worst toxicity (according to CTCAE v3.0 see appendix G) experienced in the preceding cycle and for suitability to proceed with the next cycle of treatment.

Blood Pressure Assessment
Should be performed at every visit. Blood pressure measurements should be taken after the patient has been in a resting position for >5 minutes. Repeated measurement of BP for verification should be undertaken if the initial reading is >140/90 mmHg. If BP is consistently >140/90mmHg treatment should be delayed or omitted as applicable while BP is controlled, please refer to the recommendations within section A4.2.6 (Also see **Appendix E: Guidance on Treating Hypertension and Figure 5:** Example of Management Plan for Treating Hypertension.)

Dipstick Urine Analysis
Any urine dipsticks abnormal for protein (2+ or more) should be followed up with a urine protein-creatinine ratio measurement or 24 hour urine collection for protein as described in Section 7.2.7 of this appendix.

Haematology and Serum Biochemistry (FBC and Nadir Blood Count)
Full blood count should be performed on first day of each cycle or up to 3 days before. Coagulation profile will not be repeated as a routine unless clinically indicated.

All patients must have a nadir blood count during cycle 1 treatment (at approximately day 10 of treatment) as described in Section 6.1.1 of this appendix.

Renal Function Assessment
An estimated creatinine clearance should be done on the first day of each cycle or up to 3 days before, if this is <60ml/min Section 6.3.6 of this appendix gives instructions for dose reductions in response to low creatinine clearances **(see Appendix I: Cockcroft and Gault Formula).**

During post-operative treatment and bevacizumab maintenance therapy, the same schedule of assessment as during pre-operative treatment applies.

8.2. Tumour Response Assessment Prior to Surgery

Following completion of pre-operative treatment the patients should be assessed for response, within 2 weeks of completing pre-operative treatment. This assessment should be carried out regardless of whether the patient continues on protocol treatment. If the patient stops treatment early then a Tumour Assessment Form (ST03/4) should also be completed at the time treatment stops. This assessment measures tumour response using the RECIST Version 1.0 guidelines (see Appendix F).

This tumour assessment should include:
Spiral/multi-slice CT, with oral contrast or water including chest, abdomen (pelvis optional). Maximum slice width 5mm. IV contrast/venous phase.

Other staging investigations, such as laparoscopy, EUS or PET scanning, should only be performed if clinically indicated, and according to local practice.

Patients should also be assessed for fitness for surgery and anaesthetic risk according to local practice.

Table 8.1 ST03 Trial Assessments

Required Trial Assessments	Pre-Randomisation	Before each cycle (pre- & post-operative)	Pre-surgery	Before each cycle of Maintenance Bevacizumab	Follow-up	Time Point
Clinical Examination (including BP)	Y	Y	Y	Y	Y	• Pre-randomisation
**Trans ST03 Blood sample collection**	Y					▪ Before patient starts chemotherapy
CT scan-abdomen and chest (neck pelvis optional)	Y		Y		As clinically indicated	▪ **See note 1**<br>▪ Within 2 weeks of completing last capecitabine tablet in pre-operative chemotherapy
EUS (as per local practice)	Y					According to local policy
Laparoscopy	Y					Within 4 weeks of randomisation
MRI and FDG PET scans (**only if centre is participating in MRI & FDG PET/CT sub-studies**)	Y (MRI & FDG PET) (only once eligibility to ST03 confirmed)	Y(only FDG PET scan)<br>- done between days 14-21 of 1st cycle of pre-operative chemotherapy	Y(only MRI scan)			• Baseline scan can be pre or post randomisation provided eligibility has been confirmed<br>• MRI Within 2 weeks of completing last capecitabine tablet in pre-operative chemotherapy<br>• Pre-op FDG PET as per local practice
ECHO or MUGA<br>Ejection fraction should be ≥50% or above centre's LLN for MUGA if this is <50%	Y					Within 6 weeks of randomisation<br>Further assessment if clinically indicated
ECG	Y					Within 4 weeks of randomisation
FBC: WBC >3 x 10^9/l<br>Platelets ≥100 x 10^9/l<br>Neutrophils ≥1.5 x 10^9/l<br>Nadir Blood Count day 10 cycle 1	Y	Platelets ≥75 x 10^9/l<br>Neutrophils ≥1 x 10^9/l<br>Nadir Blood Count (day 10 cycle 1)		Y	If necessary	▪ 1 week before randomisation<br>▪ Day 1 of each cycle or up to 3 days before<br>▪ Nadir blood count at day 10 cycle 1 (+/- 2 days)
Glomerular Filtration Rate (measured or estimated)	Y	Y				▪ 1 week before randomisation<br>▪ Day 1 of each cycle or up to 3 days before
Coagulation Profile (INR/APTT normal range)	Y					▪ Coagulation profile not to be repeated unless clinically indicated

**Required Trial Assessments**	**Pre-Randomisation**	**Before each cycle (pre- & post-operative)**	**Pre-surgery**	**Before each cycle of Maintenance Bevacizumab**	**Follow-up**	**Time Point**
Urine Analysis (Dip Stick)	Y	Y		Y		▪ Pre randomisation 24 hr urine collection or urinary protein-creatinine ratio required if dipstick shows ≥2+ of protein<br>▪ Then dipstick urinalysis
U and E serum creatinine	Y	Y				
Liver Function Tests ≤1.5xULN, x2.5 ALT/ AST, x3 ALP	Y	Y		Y		▪ 1 week before randomisation<br>▪ Day 1 of each cycle or up to 3 days before
PET/CT scan for centres not participating in FDG PET/CT sub-study	optional					**See note 1**
**Trans-ST03 collection of tumour samples**	Y*		Y*			***Biopsy and Resection blocksTo be sent to the Leeds Laboratory after surgery**
QoL Assessment	Y	End of Cycle 3 & 6	Y		Y	**See Table 11.2**
Pregnancy Test	Y					-ve pregnancy test result, within 7 days of starting treatment, if appropriate

Note 1: The last tumour staging investigation, which may include EUS, CT, laparoscopy or PET/CT should normally be within 28 days prior to randomisation.

8.3 Time Point for CRF Return Schedule

Table 8.2 Time Points for Completion of CRFs

Time	Form(s)
**Pre-Randomisation**	Randomisation Form (ST03/1)<br>(centres participating in the lapatinib feasibility study should use randomisation form ST03/1-HER for all patients)<br>Pre Treatment Screening Form (ST03/2)<br>Quality of Life (ST03/QL)
**Treatment Pre Surgery:**<br>Cycle 1, cycle 2, cycle 3<br><br>Cycle 3 Day 21	Treatment Pre –surgery Form (ST03/3)<br><br>Quality of Life (ST03/QL)
**Before Surgery**<br>End of Chemotherapy<br>Within 3 weeks before Surgery	Tumour Assessment (ST03/4)<br>Quality of Life (ST03/QL)
**After surgery**	Surgery Form (ST03/5)<br>Pathology Form (ST03/6)<br>Central Pathology Review Form (ST03-Path)<br>Trans-ST03 Tissue and Blood Collection (Trans-ST03)
**6 weeks Post Surgery Follow-Up**	Post-operative Report (ST03/7)
**Treatment Post-surgery:**<br>Cycle 4, cycle 5, cycle 6<br><br>Cycle 6, Day 21	Post Surgery Treatment (ST03/8)<br><br>Quality of Life (ST03/QL)
**9 weeks from the start of cycle 4**	End of Chemotherapy Form (ST03/9)<br>Follow up Assessment (ST03/11)
**Maintenance Bevacizumab doses 1-3**<br><br>**18 weeks follow up assessment from the start of cycle 4**	Bevacizumab Maintenance Treatment (ST03/10)<br><br>Follow-Up Assessment (ST03/11)<br>Quality of Life (ST03/QL)
**Maintenance Bevacizumab doses 4-6**<br><br>**27 weeks follow up assessment from the start of cycle 4**	Maintenance Bevacizumab (ST03/10)<br><br>Follow up Assessment (ST03/11)
**12 Months Post Surgery**	Follow up Form (ST03/11)<br>Quality of Life (ST03/QL)
**18 Months Post Surgery**	Follow up Form (ST03/11)<br>Quality of Life (ST03/QL)
**24 Months Post Surgery**	Follow up Form (ST03/11)<br>Quality of Life (ST03/QL)
**30 Months Post Surgery**	Follow up Form (ST03/11)
**36 Months Post Surgery**	Follow up Form (ST03/11)
**Annually thereafter**	Follow up Form (ST03/11)
**Death**	Death Form (ST03/12)
**Withdrawal From Trial**	Withdrawal Form (ST03/15)

Table 8.3 Intended CRF completion schedule according to treatment received during the trial

CRF type (number)	All protocol treatment received	Scenario 1†	Scenario 2†
**Randomisation (1) Pre-chemo screening (2)**	Pre-randomisation	Pre-randomisation	Pre-randomisation
**Pre-surgery chemo (3)**	End cycles 1-3	End of each cycle received	End of each cycle received
**Tumour assessment (4)**	After end cycle 3	After last pre-op cycle received	After last pre-op cycle received
**Surgery (5)**	Immediately after surgery	Immediately after surgery	At time of decision not to perform surgery
**Pathology (6)**	Immediately after surgery	Immediately after surgery	N/A
**Post-operative (7)**	6 wks post-op	6 wks post-op	N/A
**Post-surgery chemo (8)**	End cycles 4-6	N/A	N/A
**End of chemo (9)**	After end cycle 6	At time of decision not to give post-op chemo	At time of decision not to perform surgery
**Bevacizumab maintenance* (10)**	After each bevacizumab dose	N/A	N/A
**Follow-up (11)**	-	-	23 wks from start cycle 1
	9 wks from start cycle 4	32 wks from start cycle 1	32 wks from start cycle 1
	18 wks from start cycle 4	41 wks from start cycle 1	41 wks from start cycle 1
	27 wks from start cycle 4	50 wks from start cycle 1	50 wks from start cycle 1
	12 mths post-op	12 mths post-op	15 mths after start cycle 1
	18 mths post-op	18 mths post-op	21 mths after start cycle 1
	24 mths post-op	24 mths post-op	27 mths after start cycle 1
	30 mths post-op	30 mths post-op	33 mths after start cycle 1
	36 mths post-op	36 mths post-op	39 mths after start cycle 1
	Annually thereafter	Annually thereafter	Annually thereafter
**Death (12)**	As appropriate	As appropriate	As appropriate
**SAE (13)**	As appropriate	As appropriate	As appropriate
**QoL forms**	Pre-randomisation	Pre-randomisation	Pre-randomisation
	End cycle 3	9 weeks from start cycle 1	9 weeks from start cycle 1
	Within 3 weeks prior to surgery	Within 3 weeks prior to surgery	12 weeks from start cycle 1
	End cycle 6	32 wks from start cycle 1	32 wks from start cycle 1
	18 wks from start cycle 4	41 wks from start cycle 1	41 wks from start cycle 1
	12, 18 & 24 mths post-op	12, 18 & 24 mths post-op	15, 21 & 27 mths from start cycle 1

† **Scenario 1:** Patient receives some or all of the pre-operative chemotherapy and undergoes surgery but does not start post-operative chemotherapy

Scenario 2: Patient receives some or all of the pre-operative chemotherapy but does not undergo surgery (or subsequent post-operative chemotherapy)

* sECX+B patients only

In general, the dates given in the treatment schedule sent to you after each patient randomisation should be followed, even if patients do not complete protocol treatment. Please contact the trial office if you would like an updated treatment schedule sent to you for any patients that come off protocol treatment.

9. Statistical Considerations for the Bevaciuzmab Comparison

9.1 Method of Randomisation

Patients will be randomised centrally using a computerised algorithm developed and maintained by the MRC CTU. Randomisation will be performed using the method of minimisation over a number of clinically important stratification factors with an additional random element. To decrease determinability, the factors are not listed here.

9.2 Outcome Measures

9.2.1 Bevacizumab Comparison

Stage 1 (phase II)

Primary:

The primary outcome measure for Stage 1 was the safety of the investigational arm, in particular the rates of:

Gastric perforations at the site of the primary tumour
Cardiovascular complications, specifically congestive (i.e. symptomatic) cardiac failure, myocardial infarction and symptomatic (i.e. requiring treatment) arrhythmias
Perforations elsewhere in the intestine
Wound-healing related events
Gastrointestinal bleeding.

We intend to publish the results of the cardiac assessments, GI perforation rates and cardiovascular complication rates at the end of phase II (i.e. after 200 patients are expected to have completed pre-operative treatment). These data have been presented in abstract and poster form at the ASCO 2011 General Meeting.

Secondary:

The secondary outcome measures will be feasibility in terms of (i) patients acceptance of the randomisation as reflected in the rate of accrual and (ii) the proportion of patients that are able to complete the treatment.

Stage 2 (Phase III)

Primary:

The primary outcome for Stage 2 will be overall survival.

Secondary:

The secondary outcome measures are:

Response rates to pre-operative treatment
Surgical complete resection rates
Treatment-related morbidity
Disease-free survival
Quality of life
Cost-effectiveness

9.3 Sample Size

9.3.1 Bevacizumab Comparison
Stage I: safety and feasibility

In the first stage of the trial, patients will be randomised as in stage 2, but with formal monitoring of bevacizumab toxicity. Two formal interim reviews for the IDMC will take place when 50 then 100 sECX+B patients have completed their pre-operative treatment to look in particular at the incidence of tumour perforation, and cardiovascular events, but also wound healing complications and GI bleeding.

For the safety analyses, one-sided tests with a 10% significance level are appropriate as the interest is only in detecting unacceptably high event rates in the sECX-B arm. The tumour perforation rate in the control arm is expected to be negligible. In the Hurwitz study (8), the incidence of GI perforation in the context of response was 0.8 - 1.5%. The trial will be monitored to identify the likelihood of the tumour perforation rate exceeding 5-10% in the sECX-B arm. Using Fleming's design, if the true rate is ≤2%, 100 patients treated would give 90% power to exclude rates above 7%, at the 10% (1-sided) significance level. If the true rate is less than 1%, 100 patients would give similar power to exclude rates above 5%.

We anticipate at most 3-5% of patients on the sECX arm will experience the specific cardiovascular complications mentioned in section 13.2, and wish to exclude an absolute increase of 10% (i.e. to 13-15%) in the sECX+B arm. With 100 patients in each arm, an increase from 3% to 13% could be detected with 91% power, while an increase from 5% to 15% could be detected with 86% power.

To demonstrate the feasibility of accrual, we propose that in months 7-12 after the first patient has been recruited, 10 patients per month should be recruited and from month 13, 20 patients per month. Accrual rates during the first 6 months of the study are likely to be lower due to the time needed for centres to obtain local approvals. Formal interim analyses will be performed after 100 and 200 patients have been recruited and completed pre-operative treatment. 1 patient per month from 20 of the 34 NCRN networks should be feasible. Our sample size calculations allow for recruitment over 4 years at a rate of 250-300 per annum.

Stage 2: Efficacy

For the purposes of sample size calculation, the 5-year survival of the control arm has been estimated at 40%, which is slightly better than that observed in the CSC arm in MAGIC. This takes into consideration the possible impact that improvements in surgical technique, staging and supportive care may have had in recent years, but is also conservative, slightly increasing the number of patients required compared to more pessimistic estimates. A further 10% improvement in survival (corresponding to a HR of 0.76) would be consistent with the benefit observed by adding bevacizumab in other settings, for example the survival HR in the Hurwitz study (11) was 0.66.

To detect a hazard ratio of 0.76, equivalent to a 10% absolute increase in survival at 5 years from 40% to 50%, with 80% power (2-sided 5% significance level) requires 420 deaths to be observed. This would be achieved over a total trial period of approximately 5 years, by randomising 1100 patients over 3-4 years with a further 18-24 months follow-up prior to the primary analysis (which takes 6 months). The same number of events would be achieved by randomising ~900 patients over 5 years, and following up for 2, prior to the primary analysis. The maximum sample size will therefore be 1100 patients.

A further analysis is planned when 632 deaths have been observed, this would provide 80% power to detect a hazard ratio of 0.80 (equivalent to an 8% absolute survival difference at 5 years). The feasibility and timing of this analysis would depend on the total number of patients randomised in order to achieve the primary target of 420 deaths.

9.4 Interim Monitoring and Analyses

9.4.1 Bevacizumab Comparison

Formal interim analyses of the accumulating data will be performed after 100 and 200 patients have completed pre-operative treatment and subsequently at regular intervals (approximately annually) for review by an Independent Data Monitoring Committee (IDMC) (see also section 19). These analyses will be performed by the trial team at the MRC CTU. The IDMC will be asked to give advice on whether the accumulating data from the trial, together with results from any other relevant trials, justifies continuing recruitment of further patients or further follow-up. A decision to discontinue recruitment, in all patients or in selected subgroups will be made only if the result is likely to convince a broad range of clinicians including those entering patients into the trial and the general clinical community. If a decision is made to continue, the IDMC will advise on the frequency of future reviews of the data on the basis of accrual and event rates. The IDMC will make recommendations to the Trial Steering Committee **(TSC, see Section 30 of the main protocol)** as to whether the trial should continue in its present form. While the trial is ongoing the accumulating data will remain confidential.

9.5 Outline Analysis Plan

The principal analysis methods for the primary outcome measures are described below; a more detailed analysis plan will be prepared and made available as a separate document.

9.5.1 Bevaciuzmab Comparison

Stage I

Gastric perforation rates and cardiovascular complication rates will be presented as proportions, with exact 80% confidence intervals (to reflect the one-sided design with 10% significance level); the difference in proportions between arms will also be compared and presented with the corresponding 80% CI.

Stage II

Survival times from randomisation to death or censoring will be presented using Kaplan Meier curves and compared using the Logrank test. The treatment hazard ratio will be estimated from an unadjusted Cox proportional hazards regression model and presented with its corresponding 95% confidence interval.

Please refer to Section 16 onwards in the main body of the ST03 protocol for instruction on surgery, safety reporting procedures, pathology research, follow up and for further information on the ST03 trial.

Appendix T References

1. Okines AF, Langley RE, Thompson LC, et al: Bevacizumab with peri-operative epirubicin, cisplatin and capecitabine (ECX) in localised gastro-oesphageal adenocarcinoma: a safety report. Ann Oncol 24:702-9, 2013.

2. Hicklin DJ, Ellis LM: Role of the vascular endothelial growth factor pathway in tumor growth and angiogenesis. J Clin Oncol 23:1011-27, 2005

3. Kabbinavar F, Wong J, Ayala R, et al. The effect of antibody to vascular endothelial growth factor and cisplatin on the growth of lung tumours in nude mice. Proc. Am. Assoc. Cancer Res, 1995; 36:48

4. Yuan F, Chen Y, Dellian M, et al. Time dependent vascular regression and permeability changes in established human tumour xenografts induced by an anti-vascualar endothelial growth factor/vascular permeability factor antibody. Proc. Natl. Acad. Sci. USA 1996; 93: 14765-14770

5. Borgstrom P, Gold DP, Hillan N, et al. (La Jolla; San Francisco; San Diego, CA, USA). Importance of VEGF for breast cancer angiogenesis in vivo: implications from intravital microscopy of combination treatments with an Anti-VEGF neutralizing monoclonal antibody and doxorubicin. Anticancer Research. 1999; 19 (5B): 4203-4214

6. Von Minckwitz G, Eidtmann H, Rezai M, et al. Neoadjuvant chemotherapy and bevacizumab for HER2-negative breast cancer. N Engl J Med. 2012 Jan 26; 366 (4): 299-309

7. Ohtsu A, Shah MA et al. Bevacizumab in combination with chemotherapy as first line therapy in advanced gastric cancer: a randomised, double-blind, placebo-controlled phase III study. J Clin Oncol 2011; 29:3968-3976.

8. Perren T, Swart A-M, Pfisterer J et al. A phase 3 trial of bevacizumab in ovarian cancer. N Engl J Med. 2011 Dec 29; 365: 2484-2496

9. Miller KD, Chap LI, Holmes FA et al. Randomised phase III trial of capecitabine compared with bevacizumab plus capecitabine in patients with previously treated metastatic breast cancer. J Clin Oncol. 2005; 23: 792-799.

10. Miller K, Wang M, Gralow J et al. First-line bevacizumab and paclitaxel in patients with locally recurrent or metastatic breast cancer: a randomised phase III trial coordinated by the Eastern Cooperative Oncology Group (E2100) (Abstract 275) Eur J Cancer Suppl. 2005; 3 (77).

11. Hurwitz H, Fehrenbacher L, Novotny W, et al: Bevacizumab plus irinotecan, fluorouracil, and leucovorin for metastatic colorectal cancer. N Engl J Med 350:2335-42, 2004

12. Sandler A, gray R, Brahmer J. Randomised phase II/III trial of paclitaxel plus carboplatin with or without bevacizumab in patients with advanced non-squamous non-small cell lung cancer ECOG Trial E4599 Plenary session ASCO 2005 Orlando.

13. Reddy GK. The addition of bevacizumab to FOLFOX4 prolongs survival in relapsed colorectal cancer: interim data from the ECOG 3200 trial. Clin. Colorectal Cancer 2005 4 (5): 300-1.

14. Kabbinavar FF, Schulz J, McCleod M, et al: Addition of Bevacizumab to Bolus Fluorouracil and Leucovorin in First-Line Metastatic Colorectal Cancer: Results of a Randomized Phase II Trial. J Clin Oncol, 2005

15. Hochster HS, Welles L, Hart L: Bevacizumab (B) with oxaliplatin (O)-based chemotherapy in the first-line therapy of metastatic colorectal cancer (mCRC): Preliminary results of the randomized "TREE-2" trial. Program and abstracts of the 2005 Gastrointestinal Cancers Symposium; January 27-29, 2005; Hollywood, Florida

16. Shah MA, Ramanathan RK, Ilson DH, Levnor A, D'Adamo D, O'Reilly E, et al. Multicenter phase II study of irinotecan, cisplatin, and bevacizumab in patients with metastatic gastric or gastroesophageal junction adenocarcinoma. J Clin Oncol. 2006 Nov 20;24(33):5201-6.

17. Ajani JA, Baker J, Pisters PW, et al: CPT-11 plus cisplatin in patients with advanced, untreated gastric or gastroesophageal junction carcinoma: results of a phase II study. Cancer 94:641-6, 2002

18. Duff SE, Li C, Jeziorska M, et al: Vascular endothelial growth factors C and D and lymphangiogenesis in gastrointestinal tract malignancy. Br J Cancer 89:426-30, 2003

19. Burger RA, Brady MF, Bookman MA, et al. Incorporation of bevacizumab in the primary treatment of ovarian cancer. N Engl J Med 2011 Dec 29; 365: 2473-2483

20. Allegra CJ, Yothers G, O'Connell MJ, et al: Phase III trial assessing bevacizumab in stages II and III carcinoma of the colon: results of NSABP protocol C-08. J Clin Oncol 29:11-6, 2010

21. A. De Gramont EVC, J. Tabernero, M. J. Moore, D. Cunningham, F. Rivera, S. A. Im, M. Makrutzki, A. Shang, and P. M. Hoff AVANT: Results from a randomized, three-arm multinational phase III study to investigate bevacizumab with either XELOX or FOLFOX4 vs. FOLFOX4 alone as adjuvant treatment for colon cancer. Gastrointestinal Symposium; 2011; San Francisco; 2011. p. Abstract number 362.

22. Folkman J: Tumor angiogenesis: therapeutic implications. N Engl J Med 285:1182-6, 1971

23. Hanahan D, Weinberg RA: The hallmarks of cancer. Cell 100:57-70, 2000

24. Willett CG, Boucher Y, di Tomaso E, et al: Direct evidence that the VEGF-specific antibody bevacizumab has antivascular effects in human rectal cancer. Nat Med 10:145-7, 2004

25. Yang JC, Haworth L, Sherry RM, et al: A randomized trial of bevacizumab, an anti-vascular endothelial growth factor antibody, for metastatic renal cancer. N Engl J Med 349:427-34, 2003

26. REGARD Trial: Fuchs C S, Tomasek J, et al: Ramucirumab monotherapy for previously treated advanced gastric or gastro-oesophageal junction adenocarcinoma (REGARD): an international, randomised, multicentre, placebo-controlled, phase 3 trial. The Lancet 383, 9911, 31-39, 2014.

27. Van Cutsem E, Rivera F, Cunningham D: Safety and efficacy of first-line bevacizumab with folfox, xelox, folfiri and fluropyrimidines in metastatic colorectal cancer: the BEAT study. Ann Oncol 2009, 20; 1842-1847.

28. Kozloff M, Yood M, Grothey A: Clinical outcomes associated with bevacizumab-containing treatment of metastatic colorectal cancer: the BRiTE observational cohort study. The Oncologist 2009, 14; 862–870

29. Roche: Avastin Summary of Product Characteristics . 2005

30. Allegra C, Yothers G, O'Connell M: Initial safety report of NSABP C-08: a randomised phase III study of modified FOLFOX6 with or without bevacizumab for the adjuvant treatment of patients with stage II or III colon cancer. J Clin Oncol 2009, 27; 3385-3390.

31. Chen HX, Mooney M, Boron M et al. Phase II multicenter trial of bevacizumab plus fluorouracil and leucovorin in patients with advanced refractory colorectal cancer: an NCI Treatment Referral Center Trial TRC-0301. J Clin Oncol 2006; 24 3325-7.

32. D'Adamo DR, Anderson SE, Albritton K, et al. Phase II study of doxorubicin and bevacizumab for patients with metastatic soft-tissue sarcomas. J Clin Oncol 23: 7135-42, 2005.

33. Ganjoo KN, An CS, Robertson MJ, et al. Rituximab, bevacizumab, and CHOP (RA-CHOP) in untreated diffuse large B-cell lymphoma: safety, biomarker and pharmacokinetic analysis. Leukemia & Lymphoma 47: 998-1005; 2006

34. Skillings JR, Johnson DH, Miller K, et al: Arterial thromboembolic events in a protocol analysis of 5 randomised, controlled trials of bevacizumab with chemotherapy. J. Clin Cncol 2005; 23 (Suppl): 196s (abstract 3019)

35. Hurwitz HI, Saltz LB, Van Cutsem E et al: Venous thromboembolic events with chemotherapy plus bevacizumab: a pooled analysis of patients in randomized phase II and III studies. J Clin Oncol 2011 May 1;29:1757-64. Epub 2011 Mar 21.

36. Gordon MS, Cunningham D: Managing Patients Treated with Bevacizumab Combination Therapy. Oncology 2005; 69 (suppl 3): 25-33
