# Supplementary material for: Safety and Efficacy of the Addition of Lapatinib to Perioperative Chemotherapy for Resectable HER2-Positive Gastroesophageal Adenocarcinoma: A Randomized Phase 2 Clinical Trial
Source: JAMA Oncol. 2019 Jun 20;5(8):1181–7. doi: 10.1001/jamaoncol.2019.1179 (PMC6587151; doi:10.1001/jamaoncol.2019.1179)
Supplement: Supplement 2. — e Figure 1. HER2 Testing Flowchart eTable 1. Preoperative Toxicity eTable 2. Pathological Assessment eTable 3. Postoperative Complications eTable 4. Postoperative Toxicity [file jamaoncol-5-1181-s002.pdf]

Supplementary Online Content

Smyth EC, Rowley S, Cafferty FH, et al. Safety and efficacy of the addition of lapatinib to perioperative chemotherapy for resectable HER2-positive gastroesophageal adenocarcinoma: a randomized phase 2 clinical trial. *JAMA Oncol*. Published online June 20, 2019. doi:10.1001/jamaoncol.2019.1179

eFigure 1. HER2 Testing Flowchart

eTable 1. Preoperative Toxicity

eTable 2. Pathological Assessment

eTable 3. Postoperative Complications

eTable 4. Postoperative Toxicity

This supplementary material has been provided by the authors to give readers additional information about their work.

© 2019 Smyth EC et al. *JAMA Oncology.*

eFigure 1. HER2 Testing Flowchart

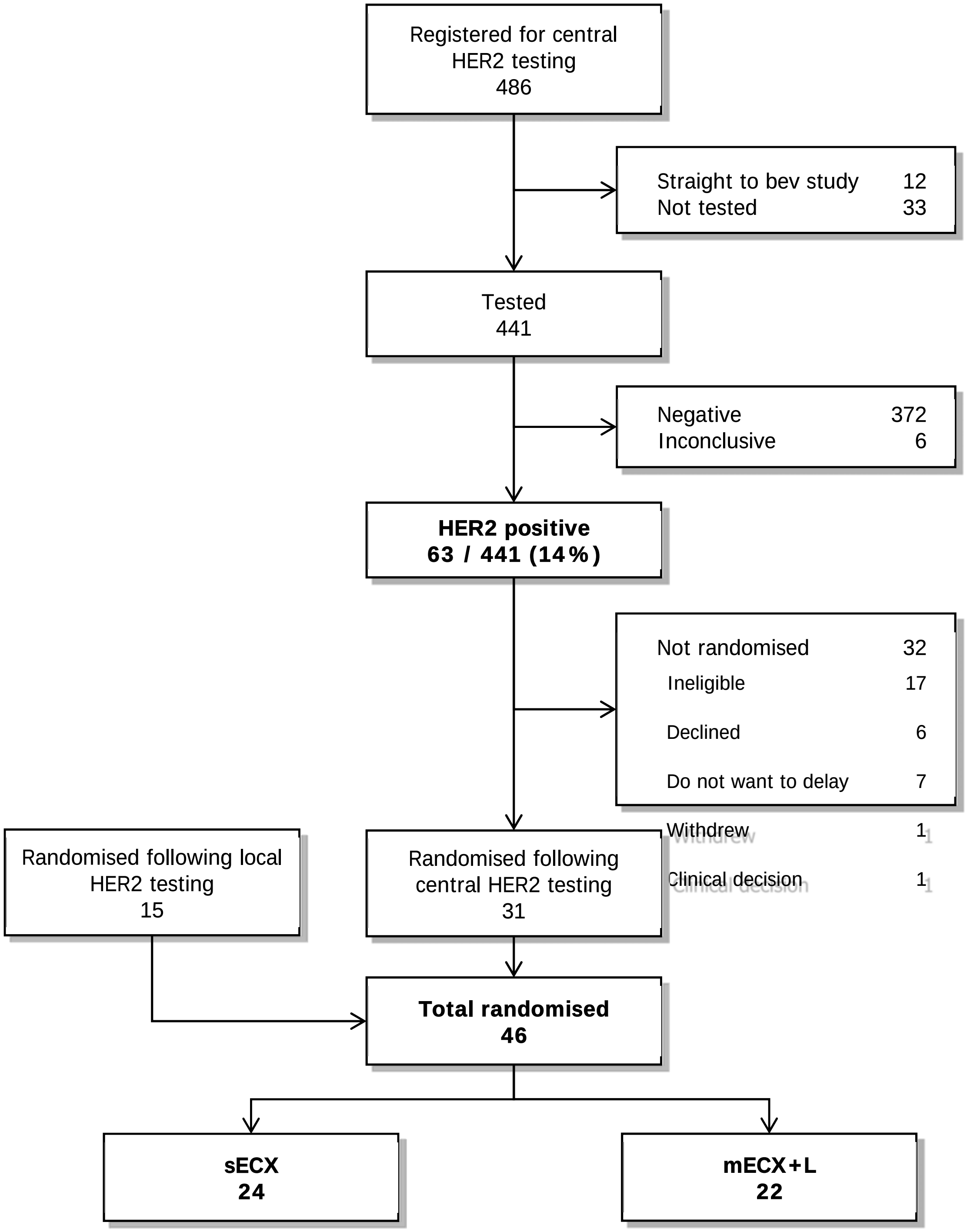

© 2019 Smyth EC et al. *JAMA Oncology.*

eTable 1. Preoperative Toxicity				
		**sECX**	**mECX+L**	**Total**
**Toxicity assessment performed**	No*	0	1	1
	Yes	24	19	43
**WORST COMMON TOXICITY CRITERIA TOXICITY GRADES**				
**Lethargy**	None	5<br>21%	4<br>21%	9<br>21%
	Grade 1-2	18<br>75%	13<br>68%	31<br>72%
	Grade 3-4	1<br>4%	2<br>11%	3<br>7%
**Nausea**	None	8<br>33%	5<br>26%	13<br>30%
	Grade 1-2	15<br>63%	14<br>74%	29<br>67%
	Grade 3-4	1<br>4%	0<br>0%	1<br>2%
**Diarrhoea**	None	14<br>58%	5<br>26%	19<br>44%
	Grade 1-2	10<br>42%	10<br>53%	20<br>47%
	Grade 3-4	0<br>0%	4<br>21%	4<br>9%
**Neutropenia**	None	11<br>46%	9<br>47%	20<br>47%
	Grade 1-2	8<br>33%	2<br>11%	10<br>23%
	Grade 3-4	5<br>21%	8<br>42%	13<br>30%
**Alopecia**	None	14<br>58%	8<br>42%	22<br>51%
	Grade 1-2	10<br>42%	11<br>58%	21<br>49%
**Stomatitis**	None	17<br>71%	8<br>42%	25<br>58%

© 2019 Smyth EC et al. *JAMA Oncology.*

	Grade 1-2	7<br>29%	11<br>58%	18<br>42%
**Vomiting**	None	17<br>71%	8<br>42%	25<br>58%
	Grade 1-2	7<br>29%	9<br>47%	16<br>37%
	Grade 3-4	0<br>0%	2<br>11%	2<br>5%
**Palmar plantar erythrodysesthesia**	None	17<br>71%	14<br>74%	31<br>72%
	Grade 1-2	7<br>29%	4<br>21%	11<br>26%
	Grade 3-4	0<br>0%	1<br>5%	1<br>2%
**Anorexia**	None	20<br>83%	14<br>74%	34<br>79%
	Grade 1-2	4<br>17%	3<br>16%	7<br>16%
	Grade 3-4	0<br>0%	2<br>11%	2<br>5%
**Peripheral neuropathy**	None	19<br>79%	16<br>84%	35<br>81%
	Grade 1-2	5<br>21%	3<br>16%	8<br>19%
**Thrombocytopenia**	None	23<br>96%	15<br>79%	38<br>88%
	Grade 1-2	1<br>4%	4<br>21%	5<br>12%
**Tinnitus**	None	23<br>96%	16<br>84%	39<br>91%
	Grade 1-2	1<br>4%	3<br>16%	4<br>9%
**Chest pain**	None	22<br>92%	18<br>95%	40<br>93%
	Grade 1-2	1<br>4%	0<br>0%	1<br>2%
	Grade 3-4	1<br>4%	1<br>5%	2<br>5%

© 2019 Smyth EC et al. *JAMA Oncology.*

**Hypertension**	None	22<br>92%	18<br>95%	40<br>93%
	Grade 1-2	1<br>4%	1<br>5%	2<br>5%
	Grade 3-4	1<br>4%	0<br>0%	1<br>2%
**Infection (normal neutrophil count)**	None	23<br>96%	17<br>89%	40<br>93%
	Grade 1-2	1<br>4%	1<br>5%	2<br>5%
	Grade 3-4	0<br>0%	1<br>5%	1<br>2%
**Infection with neutropenia**	None	22<br>92%	18<br>95%	40<br>93%
	Grade 1-2	2<br>8%	0<br>0%	2<br>5%
	Grade 3-4	0<br>0%	1<br>5%	1<br>2%
**Pulmonary embolism**	None	22<br>92%	18<br>95%	40<br>93%
	Grade 3-4	2<br>8%	1<br>5%	3<br>7%
**Renal toxicity**	None	21<br>88%	19<br>100%	40<br>93%
	Grade 1-2	3<br>13%	0<br>0%	3<br>7%
**Liver toxicity**	None	22<br>92%	19<br>100%	41<br>95%
	Grade 1-2	2<br>8%	0<br>0%	2<br>5%
**Arrhythmia**	None	24<br>100%	18<br>95%	42<br>98%
	Grade 1-2	0<br>0%	1<br>5%	1<br>2%
**Ototoxicity**	None	23<br>96%	19<br>100%	42<br>98%
	Grade 1-2	1<br>4%	0<br>0%	1<br>2%

© 2019 Smyth EC et al. *JAMA Oncology.*

**TOTAL**		**24**	**19**	**43**
* 1 patient withdrew on cycle 1, day 1 after being deemed to be inoperable, and the toxicity assessment was not performed.				

© 2019 Smyth EC et al. *JAMA Oncology.*

eTable 2. Pathological Assessment				
		**sECX**	**mECX+L**	**Total**
**Longest tumour diameter (mm)**	n	10	13	23
	Median (IQR)	35 (25 - 40)	22 (13 - 35)	29 (15 - 40)
	Range	12 – 280	0 – 700	0 – 700
**Lauren classification**	Intestinal type	9 56%	10 59%	19 58%
	Diffuse type	0 0%	1 6%	1 3%
	Unclassifiable	4 25%	1 6%	5 15%
	Unknown	3 19%	5 29%	8 24%
**Differentiation**	Well / moderately	12 75%	11 65%	23 70%
(by worst area)	Poorly	3 19%	4 24%	7 21%
	Unknown	1 6%	2 12%	3 9%
**Resection margin**	R0	11 69%	12 71%	23 70%
	R1	5 31%	3 18%	8 24%
	Circumferential	*2*	*2*	*4*
	Proximal	*2*	*0*	*2*
	Unknown	*1*	*1*	*2*
	Not specified	0 0%	2 12%	2 6%
**Lymphovascular invasion**	No	10 63%	8 47%	18 55%
	Yes	6 38%	8 47%	14 42%
	Unknown	0 0%	1 6%	1 3%
**Total number of lymph nodes**	n	16	17	33
	<15	2 13%	3 18%	5 15%
	15-24	7 44%	5 29%	12 36%
	25-34	3 19%	5 29%	8 24%
	35-44	3 19%	3 18%	6 18%
	45+	1 6%	1 6%	2 6%

© 2019 Smyth EC et al. *JAMA Oncology.*

**Number of positive lymph nodes**	n	16	17	33
	Median (IQR)	0 (0 - 4)	1 (0 - 3)	0 (0 - 3)
	Range	0 – 9	0 – 24	0 – 24
**Distant metastases**	No	15 94%	17 100%	32 97%
(other than lymph nodes)	Yes	1 6%	0 0%	1 3%
**Mandard tumour regression grade**	Grade 1	1 6%	3 18%	4 12%
	Grade 2	0 0%	0 0%	0 0%
	Grade 3	2 13%	0 0%	2 6%
	Grade 4	5 31%	4 24%	9 27%
	Grade 5	3 19%	5 29%	8 24%
	Unknown	5 31%	5 29%	10 30%
**TOTAL**		**16**	**17**	**33**

© 2019 Smyth EC et al. *JAMA Oncology.*

eTable 3. Postoperative Complications							
		**sECX**		**mECX+L**		**Total**	
n		17		17		34	
**All post-op complications**	None	9	53%	7	41%	16	47%
(maximum severity)	Non-life threatening	6	35%	8	47%	14	41%
	Life threatening	2	12%	2	12%	4	12%
**Revisional operations**	No	17	100%	15	88%	32	94%
	Yes	0	100%	2	12%	2	6%
**Life-threatening complications**							
Any life-threatening complication		2	12%	2	12%	4	12%
Respiratory failure		1	6%	2	12%	3	9%
Respiratory tract infection		1	6%	0	0%	1	3%
Empyema		1	6%	0	0%	1	3%
Intra-abdominal sepsis		0	0%	1	6%	1	3%
**All complications, regardless of severity***							
Any complication		8	47%	10	59%	18	53%
Respiratory tract infection		3	18%	2	12%	5	15%
Anastomotic leak†		3	18%	2	12%	5	15%
Wound healing complications		1	6%	3	18%	4	12%
Wound infection (superficial)		1	6%	3	18%	4	12%
Respiratory failure		2	12%	2	12%	4	12%

© 2019 Smyth EC et al. *JAMA Oncology.*

Cardiac complications	2	12%	0	0%	2	6%
Empyema	2	12%	0	0%	2	6%
MRSA	0	0%	1	6%	1	3%
Pulmonary embolism	1	6%	0	0%	1	3%
Intra-abdominal sepsis	0	0%	1	6%	1	3%
Pleural effusion requiring treatment	1	6%	0	0%	1	3%
Deep wound infection	0	0%	1	6%	1	3%
Other post-operative complications	1	6%	4	24%	5	15%

© 2019 Smyth EC et al. *JAMA Oncology.*

Table 4. Postoperative Toxicity				
		**sECX**	**mECX+L**	**Total**
**Toxicity assessment performed**	No	0	0	0
	Yes	8	10	18
**WORST COMMON TOXICITY CRITERIA TOXICITY GRADES**				
**Lethargy**	None	1 13%	0 0%	1 6%
	Grade 1-2	6 75%	10 100%	16 89%
	Grade 3-4	1 13%	0 0%	1 6%
**Diarrhoea**	None	4 50%	1 10%	5 28%
	Grade 1-2	4 50%	9 90%	13 72%
**Nausea**	None	3 38%	3 30%	6 33%
	Grade 1-2	5 63%	5 50%	10 56%
	Grade 3-4	0 0%	2 20%	2 11%
**Neutropenia**	None	5 63%	2 20%	7 39%
	Grade 1-2	2 25%	4 40%	6 33%
	Grade 3-4	1 13%	4 40%	5 28%
**Anorexia**	None	4 50%	4 40%	8 44%
	Grade 1-2	4 50%	5 50%	9 50%
	Grade 3-4	0 0%	1 10%	1 6%
**Peripheral neuropathy**	None	5 63%	5 50%	10 56%
	Grade 1-2	3 38%	5 50%	8 44%
**Vomiting**	None	5 63%	6 60%	11 61%
	Grade 1-2	3 38%	3 30%	6 33%
	Grade 3-4	0 0%	1 10%	1 6%
**Alopecia**	None	7 88%	4 40%	11 61%
	Grade 1-2	1 13%	6 60%	7 39%

© 2019 Smyth EC et al. *JAMA Oncology.*

**Tinnitus**	None	8 100%	5 50%	13 72%
	Grade 1-2	0 0%	5 50%	5 28%
**TOTAL**		**8**	**10**	**18**

© 2019 Smyth EC et al. *JAMA Oncology.*
