# Supplementary material for: Safety and Efficacy of the Addition of Lapatinib to Perioperative Chemotherapy for Resectable HER2-Positive Gastroesophageal Adenocarcinoma: A Randomized Phase 2 Clinical Trial
Source: JAMA Oncol. 2019 Jun 20;5(8):1181–7. doi: 10.1001/jamaoncol.2019.1179 (PMC6587151; doi:10.1001/jamaoncol.2019.1179)
Supplement: Supplement 3. — Data Sharing Statement [file jamaoncol-5-1181-s003.pdf]

# Data Sharing Statement

Smyth. Safety and Efficacy of the Addition of Lapatinib to Perioperative Chemotherapy for Resectable HER2-Positive Gastroesophageal Adenocarcinoma. *JAMA Oncol*. Published June 20, 2019. 10.1001/jamaoncol.2019.1179

**Data**
**Data available:** No

**Additional Information**
**Explanation for why data not available:** This will be considered following submission
